# A family of 512 reverse order laws for generalized inverses of a matrix product: A review

**DOI:** 10.1016/j.heliyon.2020.e04924

**Published:** 2020-09-28

**Authors:** Yongge Tian

**Affiliations:** College of Business and Economics, Shanghai Business School, Shanghai, China

**Keywords:** Mathematics, Matrix product, Orthogonal projector, Idempotent matrix, Generalized inverse, Reverse order law, Matrix-valued function, Linear statistical model, Ordinary least-squares estimator, Block matrix methodology, Matrix equation methodology, Matrix rank methodology

## Abstract

Reverse order laws for generalized inverses of matrix products are a classic object of study in the theory of generalized inverses. One of the well-known reverse order laws for a matrix product *AB* is ${\left(AB\right)}^{\left(i,\ldots ,j\right)}=B^{\left(s_{2},\ldots ,t_{2}\right)}A^{\left(s_{1},\ldots ,t_{1}\right)}$, where ${\left(\cdot \right)}^{\left(i,\ldots ,j\right)}$ denotes a {i,…,j}-generalized inverse of matrix. Because {i,…,j}-generalized inverse of a singular matrix is not unique, the relationships between both sides of the reverse order law can be divided into four situations for consideration. The aim of this paper is to give an overview of plenty of results concerning reverse order laws for {i,…,j}-generalized inverses of the product *AB*, from the development of background and preliminary tools to the collection of miscellaneous formulas and facts on the reverse order laws in one place with cogent introduction and references for further study. We begin with the introduction of a linear mixed model y=ABβ+Aγ+ϵ and the presentation of two least-squares methodologies for estimating the fixed parameter vector *β* in the model, and the description of connections between the two types of least-squares estimators and the reverse order laws for generalized inverses of *AB*. We then prepare various necessary matrix study tools, including a general theory on linear or nonlinear algebraic matrix identities, a group of expansion formulas for calculating ranks of block matrices, two groups of explicit formulas for calculating the maximum and minimum ranks of $B^{\left(s_{2},\ldots ,t_{2}\right)}A^{\left(s_{1},\ldots ,t_{1}\right)}$, as well as necessary and sufficient conditions for $B^{\left(s_{2},\ldots ,t_{2}\right)}A^{\left(s_{1},\ldots ,t_{1}\right)}$ to be invariant with respect to the choice of the generalized inverses, etc. Subsequently, we present a unified approach to the 512 matrix set inclusion problems associated with the above reverse order laws for the eight commonly-used types of generalized inverses of *A*, *B*, and *AB* by means of the definitions of generalized inverses, the block matrix methodology (BMM), the matrix equation methodology (MEM), and the matrix rank methodology (MRM).

## 1 Introduction

We begin with introducing the notation adopted in this paper. Let $R^{m\times n}$ and $C^{m\times n}$ denote the collections of all m×n real and complex matrices, respectively; $A^{T}$ and $A^{⁎}$ denote the transpose and the conjugate transpose, respectively; r(A) denote the rank of *A*, i.e., the maximum order of the invertible submatrix of *A*; $R(A)=\{Ax\;|\;x\in C^{m\times 1}\}$ and $N(A)=\{x\in C^{n\times 1}\;|\;Ax=0\}$ denote the range and the null space of a matrix $A\in C^{m\times n}$, respectively; $I_{m}$ denote the identity matrix of order *m*; and [A,B] denote a columnwise partitioned matrix consisting of two submatrices *A* and *B*. A matrix $A\in C^{m\times m}$ is said to be Hermitian if $A=A^{⁎}$; to be EP (or range Hermitian) if $R(A)=R(A^{⁎})$; to be idempotent if $A^{2}=A$; to be orthogonal projector if $A^{2}=A=A^{⁎}$. The Moore–Penrose inverse of $A\in C^{m\times n}$, denoted by $A^{†}$, is defined to be the unique matrix $X\in C^{n\times m}$ satisfying the four Penrose equations

(1.1) $$\left(1\right)\;\;AXA=A,\;\;\;\left(2\right)\;\;XAX=X,\;\;\;\left(3\right)\;\;{\left(AX\right)}^{⁎}=AX,\;\;\;\left(4\right)\;\;{\left(XA\right)}^{⁎}=XA.$$

A matrix *X* is called a {i,…,j}-generalized inverse of *A*, denoted by $A^{\left(i,\ldots ,j\right)}$, if it satisfies the *i*th, …,jth equations in (1.1). The collection of all {i,…,j}-generalized inverses of *A* is denoted by $\left\{A^{\left(i,\ldots ,j\right)}\right\}$. There are all 15 types of {i,…,j}-generalized inverses of *A* by definition, but people are mainly interested in the following eight situations that involve the equation AXA=A:

(1.2) $$A^{†},\;\;A^{\left(1,3,4\right)},\;\;A^{\left(1,2,4\right)},\;\;A^{\left(1,2,3\right)},\;\;A^{\left(1,4\right)},\;\;A^{\left(1,3\right)},\;\;A^{\left(1,2\right)},\;\;A^{\left(1\right)},$$

which are usually called the eight commonly-used types of generalized inverses of *A* in the literature (cf. [15], [17], [65]). The Moore–Penrose inverse of a matrix *A* was specially studied and recognized because $AA^{†}$, $A^{†}A$, $I_{m}-AA^{†}$, and $I_{n}-A^{†}A$ are four orthogonal projectors onto the ranges and kernels of *A* and $A^{⁎}$, respectively. Hence the Moore–Penrose inverse optimizes a number of interesting theoretical and computational properties associated with *A*, and can also be used to represent other generalized inverses by means of certain algebraic operations of *A* and $A^{†}$. In this paper, we denote by $P_{A}=AA^{†}$, $E_{A}=I_{m}-AA^{†}$, and $F_{A}=I_{n}-A^{†}A$ the three orthogonal projectors induced from *A*.

Theories of generalized inverses have been developed for several decades in the real and complex matrix algebras, operator algebras, rings, semi-groups, etc, and many kinds of generalized inverses have been defined for different purposes in theory and applications. Thus the researches concerning generalized inverses have deep roots in mathematics and have attracted sufficient attentions since 1950s. In comparison, the generalized inverses defined by the four Penrose equations of matrices have a wide range of results, facts, and formulas. Hence they are the most popular concepts and tools in the theory of generalized inverses to deal with singular matrices that occur in computational and applied mathematical problems. As always, it is a fundamental task to approach matrix expressions and equalities that involve generalized inverses of matrices from both theoretical and applied point of view. To emphasize the occurrence of generalized inverses in matrix calculations, we denote by

(1.3) $$f\left(A_{1}^{\left(i_{1},\ldots ,j_{1}\right)},A_{2}^{\left(i_{2},\ldots ,j_{2}\right)},\ldots ,A_{k}^{\left(i_{k},\ldots ,j_{k}\right)}\right)$$

a general matrix expression that involves certain algebraic operations of a family of generalized inverses $A_{1}^{\left(i_{1},\ldots ,j_{1}\right)}$, $A_{2}^{\left(i_{2},\ldots ,j_{2}\right)}$, …, $A_{k}^{\left(i_{k},\ldots ,j_{k}\right)}$, and denote by

(1.4) $$D_{f}=\left\{f\left(A_{1}^{\left(i_{1},\ldots ,j_{1}\right)},A_{2}^{\left(i_{2},\ldots ,j_{2}\right)},\ldots ,A_{k}^{\left(i_{k},\ldots ,j_{k}\right)}\right)\right\}$$

the domain of (1.3), namely, the collection of matrix values of the function with respect to all possible choices of the generalized inverses. As is known to all, one of the fundamental tasks in algebra is to establish and describe various algebraic equalities for operations of elements in the algebra. Since the theory of generalized inverses of matrices is nothing but a branch of matrix algebra, it is also a fundamental task to establish as many as possible matrix equalities for generalized inverses from theoretical and applied points of view. As usual, matrix equalities composed of generalized inverses can generally be written in the following form

(1.5) $$f\left(A_{1}^{\left(i_{1},\ldots ,j_{1}\right)},A_{2}^{\left(i_{2},\ldots ,j_{2}\right)},\ldots ,A_{k}^{\left(i_{k},\ldots ,j_{k}\right)}\right)=g\left(B_{1}^{\left(s_{1},\ldots ,t_{1}\right)},B_{2}^{\left(s_{2},\ldots ,t_{2}\right)},\ldots ,B_{l}^{\left(s_{l},\ldots ,t_{l}\right)}\right).$$

Because the matrix values of f(⋅) and g(⋅) are not necessarily unique, the relationships between both sides of (1.5) can be divided into the following four situations

(1.6) $$D_{f}\cap D_{g}\neq \emptyset ,\;\;\;D_{f}⊇D_{g},\;\;\;D_{f}\subseteq D_{g},\;\;\;D_{f}=D_{g}$$

by means of the domain notation defined in (1.4). Eqs. (1.5) and (1.6) contain a wide range of concrete topics in the theory of generalized inverses of matrices and applications. One of the most popular classes of (1.5) and (1.6) is concerned with various equalities composed of products of generalized inverses. To illustrate this class of matrix equalities, let *A* and *B* be two invertible matrices of the same size. Then the product *AB* is invertible as well and the following basic matrix equality

(1.7) $${\left(AB\right)}^{-1}=B^{-1}A^{-1}$$

always holds, which is usually called the two-term reverse order law (ROL) for the standard inverse of matrix product. A straightforward extension of (1.7) to multiple matrix products is given by ${\left(A_{1}A_{2}\cdots A_{k}\right)}^{-1}=A_{k}^{-1}\cdots A_{2}^{-1}A_{1}^{-1}$. We learned a complete story in linear algebra concerning these best known ROLs, and ubiquitously used them to compute the inverses of matrix products and to deal with various matrix expressions that involve inverse operations of products of matrices. If given matrix products are singular, people expect to write generalized inverses of the matrix products as certain reverse order products of generalized inverses of the given matrices. To take the most useful case, we assume that $A\in C^{m\times n}$ and $B\in C^{n\times p}$ are two given singular matrices. Then the product $AB\in C^{m\times p}$ is defined, yet it is usually singular as well. In this case, the three generalized inverses $A^{\left(s_{1},\ldots ,t_{1}\right)}$, $B^{\left(s_{2},\ldots ,t_{2}\right)}$, and ${\left(AB\right)}^{\left(i,\ldots ,j\right)}$ do exist, and people are interested in the relations among $A^{\left(s_{1},\ldots ,t_{1}\right)}$, $B^{\left(s_{2},\ldots ,t_{2}\right)}$, and ${\left(AB\right)}^{\left(i,\ldots ,j\right)}$. In the conventional formulations of theories of ROLs, the extensions of the standard forms of reverse order laws in (1.7) to generalized inverses of *AB* can be written as

(1.8) $${\left(AB\right)}^{\left(i,\ldots ,j\right)}=B^{\left(s_{2},\ldots ,t_{2}\right)}A^{\left(s_{1},\ldots ,t_{1}\right)},$$

which is obviously a special case of (1.5). Because the non-commutativity of matrix algebra, and also because $AA^{\left(s_{1},\ldots ,t_{1}\right)}\neq I_{m}$, $A^{\left(s_{1},\ldots ,t_{1}\right)}A\neq I_{n}$, $BB^{\left(s_{2},\ldots ,t_{2}\right)}\neq I_{n}$, and $B^{\left(s_{2},\ldots ,t_{2}\right)}B\neq I_{p}$ for two singular matrices *A* and *B*, the reverse order product $B^{\left(s_{2},\ldots ,t_{2}\right)}A^{\left(s_{1},\ldots ,t_{1}\right)}$ on the right-hand side of (1.8) does not necessarily satisfy the matrix equations defined for ${\left(AB\right)}^{\left(i,\ldots ,j\right)}$. In this case, we denote by $\left\{{\left(AB\right)}^{\left(i,\ldots ,j\right)}\right\}$ and $\left\{B^{\left(s_{2},\ldots ,t_{2}\right)}A^{\left(s_{1},\ldots ,t_{1}\right)}\right\}$ the collections of all possible choices of the matrices on both sides of (1.8). Therefore, it is natural to divide (1.8) into the following four reasonable relationships for the two matrix sets

(1.9) $$\{{\left(AB\right)}^{\left(i,\ldots ,j\right)}\}\cap \{B^{\left(s_{2},\ldots ,t_{2}\right)}A^{\left(s_{1},\ldots ,t_{1}\right)}\}\neq \{\emptyset \},$$

(1.10) $$\{{\left(AB\right)}^{\left(i,\ldots ,j\right)}\}⊇\{B^{\left(s_{2},\ldots ,t_{2}\right)}A^{\left(s_{1},\ldots ,t_{1}\right)}\},$$

(1.11) $$\{{\left(AB\right)}^{\left(i,\ldots ,j\right)}\}\subseteq \{B^{\left(s_{2},\ldots ,t_{2}\right)}A^{\left(s_{1},\ldots ,t_{1}\right)}\},$$

(1.12) $$\{{\left(AB\right)}^{\left(i,\ldots ,j\right)}\}=\{B^{\left(s_{2},\ldots ,t_{2}\right)}A^{\left(s_{1},\ldots ,t_{1}\right)}\}.$$

Each of (1.9)–(1.12) include $8^{3}=512$ situations for the eight commonly-used types of generalized inverses of *A*, *B*, and *AB*, respectively. Because generalized inverses of a matrix are defined to be common solutions of certain matrix equations, the ROL problems are in fact to describe connections among matrix expressions composed of solutions of several matrix equations, so that it is a general algebraic problem of describing these relations under various circumstances and assumptions.

Generally speaking, knowing how to characterize algebraic equalities and identities and to solve algebraic equations is a difficult problem that has no uniformly acceptable solutions, as such there are no general algebraic techniques that allow people to deal with complicated matrix-valued functions and equalities because of noncommutativity of matrix algebra. Notice that (1.9)–(1.12) do not necessarily hold for different choices of generalized inverses of the matrices, so that one wishes to find identifying conditions for (1.9)–(1.12) to hold under various assumptions. This is really a tremendous work because there are all 15 types of {i,…,j}-generalized inverse for a given matrix according to combinatoric choices of the four Penrose equations. Eqs. (1.9)–(1.12) and their extensions to multiple matrix products have been a classic objects of study in the theory of generalized inverses and applications, and have attracted considerable attention since 1960s. In fact, people have put a great deal of time and effort into the investigations of various ROLs for generalized inverses of matrix products, in particular, researches of (1.9)–(1.12) have already been flourished and diversified in contents since that beginning (cf. [1], [13], [14], [21], [24], [30], [33], [34], [44], [51], [52], [73], [74], [75], [113], [114], [115], [121], [122]). In spite of much effort devoted to the researches to ROLs problems in the past several decades, most parts of (1.9)–(1.12) remain unresolved. Given (1.9)–(1.12), a primary task is to establish necessary and sufficient conditions for the facts to hold (for the corresponding matrix equations to be solvable), but one can often be left in a confounding place of techniques, philosophies, and nuance when approaching so many different kinds of equalities. In fact, it is a common sense that knowing how to deal with a general matrix equality composed of multiple matrices is a difficult problem without uniformly acceptable solutions. No algebraist or algebraic technique can accurately tell people what to do with complicated matrix expressions and matrix equalities because the noncommutativity of real or complex matrix algebra. However, it has already been recognized since 1970s that a sufficient resolution of this kind of matrix equality problems can be established by means of matrix rank formulas and block matrix operations and representations, which can help people describe and prove many matrix equalities in a clear and concise way. In fact, these methodologies have been welcomed as most efficient and popular analytical techniques in matrix theory.

In the following, we give a specified introduction to the issues related to ranks of matrices. The rank of a matrix is defined to be the nonnegative integer that is the dimension of the row or column vector space of the matrix. This number can be calculated directly by transforming the matrix to certain row and/or column echelon forms. Besides, the rank of a matrix can be defined by some other manners. As one of the key indicators of a matrix, one can use the rank of a matrix to describe some basic properties and performances of the matrix under various assumptions, such as, the nullity, singularity, and nonsingularity of the matrix, numbers of singular values, etc. There are many simple and interesting properties concerning the rank of a matrix, one of the best-known basic facts is: A=0⇔r(A)=0. Also note the rule A=B⇔A−B=0 for any two matrices *A* and *B* of the same sizes, so that A=B⇔r(A−B)=0. Furthermore, assume that $S_{1}$ and $S_{2}$ are two sets consisting of matrices of the same size. Then it is straightforward to see from the above fact that the following two pairs of equivalent statements hold

$$S_{1}\cap S_{2}\neq \emptyset \Leftrightarrow \min_{A\in S_{1},\;B\in S_{2}}r(A-B)=0,S_{1}\subseteq S_{2}\Leftrightarrow \max_{A\in S_{1}}\min_{B\in S_{2}}r(A-B)=0.$$

Because of the simplicity of the concept of matrix rank, one can easily understand the meaning of the above assertions, and would like to take them as a useful way of characterizing connections of two matrices, as well as matrix equalities. To speak precisely, if certain analytical formulas for calculating the rank of A−B are established, people can obtain from the formulas some algebraic properties of the difference as described above, as well as necessary and sufficient conditions for A=B to hold. There were many concrete formulas for calculating ranks of matrices and their algebraic operations that were discovered in the long-lasting development of linear algebra, which are so fundamental and significant that people have conveniently used them to deal with numerous theoretical and computational problems in matrix theory. In other words, there do exist general ways of establishing nontrivial analytical formulas for calculating ranks of matrix expressions. Hence the matrix rank method described above has been recognized from theoretical and applied point of view.

In the past several decades, the present author has paid a great attention to the development of the block matrix methodology (BMM), the matrix equation methodology (MEM), and the matrix rank methodology (MRM), and has solved many fundamental problems in the theory of generalized inverses of matrices by means of the BMM, MEM and MRM. The corresponding work includes simplifying and deriving various complicated and nuanced matrix expressions and equalities, characterizing various ROLs for generalized inverses of matrix products, and establishing thousands of closed-form formulas for calculating ranks of block matrices, sums and differences of matrices, etc (cf. [78], [79], [81], [83], [84], [85], [86], [87], [88], [89], [90], [91], [93], [94], [95], [97], [98], [99], [100], [101], [102], [105], [106], [107], [109]). On the other hand, we have seen in the last two decades that various maximization and minimization problems concerning ranks of matrices have been proposed and studied in different disciplines of mathematics and applications (cf. [31], [48], [56], [59], [68], [124] among others). The past and present work shows that no methods in linear algebra and matrix theory, as described above, are more fundamental yet useful than the MRM in the study of algebraic matrix equality problems.

The main purpose of this study is to gather in a single document various known and novel formulas and facts on a family of 512 one-sided set inclusions associated with (1.10) for the eight commonly-used types of generalized inverses of *A*, *B*, and *AB* by means of the definitions of generalized inverses, the BMM, the MEM, and the MRM. This paper is organized as follows. In Section 2, we go through some basic facts of the ordinary least-squares estimators (OLSEs) for unknown parameters in a two-level linear regression model, and establish some analytical formulas for calculating the OLSEs, as well as explicit expressions of the expectations and the covariance matrices of the OLSEs by means of generalized inverses of the given vectors and matrices. We then describe a group of equivalent facts among several equalities of the OLSEs and ROLs for generalized inverses of the products of the given matrices in the model. The core work of this study is to present a classification analysis to a group of 512 one-sided matrix set inclusions associated with (1.10). To finish this task, we introduce in Section 3 various fundamental formulas of generalized inverses of matrices and their products, in Section 4 we provide various formulas for calculating ranks of matrices and their generalized inverses, and in Section 5 we present a group of results on linear and multilinear matrix identities that involve one or multiple variable matrices. Section 6 deals with the invariance properties of various matrix products that involve two matrices and their generalized inverses. Section 7 presents a family of known fundamental equalities for generalized inverses of two matrices of same size. The main results of Section 8 are a family of 126 known analytical formulas for calculating the maximum and minimum ranks of $B^{\left(s_{2},\ldots ,t_{2}\right)}A^{\left(s_{1},\ldots ,t_{1}\right)}$ and their variations with respect to the choice of the generalized inverses, as well as a variety of well-known equalities and facts concerning ranges and ranks of products of matrices and their generalized inverses. Sections 9, 10, and 11 are devoted to the establishments of necessary and sufficient conditions for (1.10) to hold, respectively. Section 12 concludes with a summary of the ROL problems addressed in this paper and mentions a list of challenging open problems concerning mixed ROLs for products of matrices. Throughout this paper, the author presents details of proofs of principle results, but omits the proofs of similar or parallel results and facts due to space limitation.

## 2 Relations between ROLs and least-squares estimators in linear statistical models

In this section, we exhibit some basic definitions and known results from least-squares estimation problems in linear statistical models that are related to ROLs. It is well known that parametric regression analysis is perhaps the most commonly employed tool in statistical data analysis and inference, while linear regression models belong to classic issues in statistical theory and are the common roots of many branches of current statistical theory. Although there has been a relatively systematical research about linear regression models and their applications in the past centuries, one can still propose many theoretical problems in this field and investigate these problems by way of various mathematical analysis tools. When using linear regression models to fit given data, unknown parameters in the models may not necessarily be assumed to be fixed, instead, to vary at more than one level, or to be given in nested forms. Multi-level hierarchical linear model is a feasible technique of fitting data that have a hierarchical structure. Because of the occurrence of parameters at more than one level, the inference of a multi-level hierarchical linear model involves various nested calculations of given matrices and vectors in the model. In fact, many problems in statistics and applications involve analyzing and manipulating this kind of data and models with nested structure (cf. [25], [26], [35], [49], [55], [63], [67], [76], [112], [120]). In this section, we start by introducing an example of two-level hierarchical linear model:

(2.1) $$M:\;y=A\alpha +\epsilon ,\;\;\alpha =B\beta +\gamma ,\;\;E(\epsilon )=0,\;E(\gamma )=0,\;\;Cov(\epsilon )=\sigma^{2}I_{n},\;\;Cov(\gamma )=\tau^{2}I_{p},\;Cov(\epsilon ,\;\gamma )=0,$$

where in the first-level model, $y\in R^{n\times 1}$ is a vector of observable response variables, $A\in R^{n\times p}$ is a known matrix of arbitrary rank, $\alpha \in R^{p\times 1}$ is a vector of unobservable random variables, $\epsilon \in R^{n\times 1}$ is a vector of randomly distributed error terms, $\sigma^{2}$ is an arbitrary positive scaling factor; in the second-level model, $B\in R^{p\times k}$ is a known matrix of arbitrary rank, $\beta \in R^{k\times 1}$ is a vector of fixed but unknown parameters, $\gamma \in R^{p\times 1}$ is a vector of unobservable random variables, $\tau^{2}$ is an arbitrary positive scaling factor. This kind of models have distinct names in statistical analysis according to their origination, such as, random-effect models, hierarchical models, nested models, etc. Substituting the second equation in (2.1) into the first equation leads to the following linear mixed model

(2.2) $$N:\;y=AB\beta +A\gamma +\epsilon ,\;\;E(y)=AB\beta ,\;\;Cov(y)=\sigma^{2}I_{n}+\tau^{2}AA^{T}.$$

It is well known that the most common technique used to estimate the unknown parameters in linear regression models is the method of least-squares. In fact, generalized inverses of matrices have a direct motivation that arises in statistical applications ranging from fitting observed data through the well-known least-squares method. For expositions of generalized inverse of matrices and applications to linear models, see e.g., [58], [63], [65].

Because of the two alternative model forms M and N in (2.1) and (2.2), there exist in fact two kinds of ordinary least-squares estimators (OLSEs) for the unknown parameter vector *β* in M and N, respectively. This fact prompts us to explore the connections between the two OLSEs from the mathematical and statistical point of view. Since there are two alternative forms in (2.1) and (2.2), respectively, we are able to employ two algebraic procedures to calculate the OLSEs of the unknown parameter vector *β* and the mean vector *ABβ* in (2.1) and (2.2), which are described as follows.

(I) The standard method is to
  (2.3) $$\mathrm{minimize}\;{\left(y-AB\beta \right)}^{T}(y-AB\beta )$$
  in the context of (2.2). We now show a primary usefulness of generalized inverses in statistics by decomposing the norm ${\left(y-AB\beta \right)}^{T}(y-AB\beta )$ in (2.3) as
  (2.4) $${\left(y-AB\beta \right)}^{T}(y-AB\beta )=y^{T}E_{AB}y+{\left(P_{AB}y-AB\beta \right)}^{T}(P_{AB}y-AB\beta ),$$
  where the Moore–Penrose inverse occurs naturally on the right-hand of the equality. Obviously, the two terms satisfy $y^{T}E_{AB}y⩾0$ and ${\left(P_{AB}y-AB\beta \right)}^{T}(P_{AB}y-AB\beta )⩾0$ (cf. [103], [104]). Hence it follows that
  $$\min_{\beta \in R^{p\times 1}}{\left(y-AB\beta \right)}^{T}(y-AB\beta )=y^{T}E_{AB}y+\min_{\beta \in R^{p\times 1}}{\left(P_{AB}y-AB\beta \right)}^{T}(P_{AB}y-AB\beta )=y^{T}E_{AB}y,$$
  which holds when *y* satisfies $P_{AB}y=AB\beta$. This equation is equivalent to the normal linear matrix equation ${\left(AB\right)}^{T}AB\beta ={\left(AB\right)}^{T}y$ by pre-multiplying ${\left(AB\right)}^{T}$, and is always consistent (cf. [36, p. 114] and [71, pp. 164–165]). Solving the equation gives the well-known OLSEs of *β* and *ABβ* under N:
  (2.5) $${\mathrm{OLSE}}_{N}(\beta )=\left({\left(AB\right)}^{†}+F_{AB}U\right)y={\left(AB\right)}^{\left(1,3\right)}y,$$
  (2.6) $${\mathrm{OLSE}}_{N}(AB\beta )=AB{\mathrm{OLSE}}_{N}(\beta )=AB{\left(AB\right)}^{\left(1,3\right)}y,$$
  where *U* is an arbitrary matrix. Furthermore, the expectations and the covariance matrices of ${\mathrm{OLSE}}_{N}(\beta )$ and ${\mathrm{OLSE}}_{N}(AB\beta )$ are given by
  (2.7) $$E\left({\mathrm{OLSE}}_{N}(\beta )\right)={\left(AB\right)}^{†}AB\beta ,$$
  (2.8) $$\mathrm{Cov}\left({\mathrm{OLSE}}_{N}(\beta )\right)={\left(AB\right)}^{†}(\sigma^{2}I_{n}+\tau^{2}AA^{T}){\left[{\left(AB\right)}^{†}\right]}^{T},$$
  (2.9) $$E\left({\mathrm{OLSE}}_{N}(AB\beta )\right)=AB\beta ,$$
  (2.10) $$\mathrm{Cov}\left({\mathrm{OLSE}}_{N}(AB\beta )\right)=AB{\left(AB\right)}^{†}(\sigma^{2}I_{n}+\tau^{2}AA^{T})AB{\left(AB\right)}^{†}.$$
(II) On the other hand, we may first solve the least-squares problem ${\left(y-A\alpha \right)}^{T}(y-A\alpha )=\min$ under (2.1) and obtain the OLSE of *α* as follows
  (2.11) $${\mathrm{OLSE}}_{M}(\alpha )=(A^{†}+F_{A}U_{1})y=A^{\left(1,3\right)}y,$$
  where $U_{1}$ is an arbitrary matrix. Substituting this formula into the second equation in (2.1) yields
  (2.12) $$A^{\left(1,3\right)}y=B\beta +\gamma .$$
  In this case, solving ${\left\|A^{\left(1,3\right)}y-B\beta \right\|}^{2}=\min$ under (2.12) leads to
  (2.13) $${\mathrm{OLSE}}_{M}(\beta )=(B^{†}+F_{B}U_{2})A^{\left(1,3\right)}y=B^{\left(1,3\right)}A^{\left(1,3\right)}y,$$
  (2.14) $${\mathrm{OLSE}}_{M}(AB\beta )=ABB^{\left(1,3\right)}A^{\left(1,3\right)}y,$$
  where $U_{2}$ is an arbitrary matrix. In the case of Moore–Penrose inverses, the expectations and the covariance matrices of these estimators are given by
  (2.15) $$E\left({\mathrm{OLSE}}_{M}(\beta )\right)=B^{†}A^{†}AB\beta ,$$
  (2.16) $$\mathrm{Cov}\left({\mathrm{OLSE}}_{M}(\beta )\right)=B^{†}A^{†}(\sigma^{2}I_{n}+\tau^{2}AA^{T}){\left(B^{†}A^{†}\right)}^{T},$$
  (2.17) $$E\left({\mathrm{OLSE}}_{M}(AB\beta )\right)=ABB^{†}A^{†}AB\beta ,$$
  (2.18) $$\mathrm{Cov}\left({\mathrm{OLSE}}_{M}(AB\beta )\right)=ABB^{†}A^{†}(\sigma^{2}I_{n}+\tau^{2}AA^{T}){\left(ABB^{†}A^{†}\right)}^{T}.$$

Note from (2.5)–(2.10) and (2.13)–(2.18) that the OLSEs under N and M are given in distinct formulas. Thus they have different statistical performances, and it would be of interest to describe the relationships between the OLSEs under N and M, in particular, it is necessary to establish identifying conditions for the following 6 equalities for the OLSEs and their expectations and covariances

(2.19) $${\mathrm{OLSE}}_{M}(\beta )={\mathrm{OLSE}}_{N}(\beta ),$$

(2.20) $$E\left({\mathrm{OLSE}}_{M}(\beta )\right)=E\left({\mathrm{OLSE}}_{N}(\beta )\right),$$

(2.21) $$\mathrm{Cov}\left({\mathrm{OLSE}}_{N}(\beta )\right)=\mathrm{Cov}\left({\mathrm{OLSE}}_{M}(\beta )\right),$$

and

(2.22) $${\mathrm{OLSE}}_{M}(AB\beta )={\mathrm{OLSE}}_{N}(AB\beta ),$$

(2.23) $$E\left({\mathrm{OLSE}}_{M}(AB\beta )\right)=E\left({\mathrm{OLSE}}_{N}(AB\beta )\right),$$

(2.24) $$\mathrm{Cov}\left({\mathrm{OLSE}}_{M}(AB\beta )\right)=\mathrm{Cov}\left({\mathrm{OLSE}}_{N}(AB\beta )\right)$$

to hold, respectively. To do so, we need to compare the coefficient matrices of *y* in (2.5) and (2.13), the expectations in (2.7), (2.9), (2.15), and (2.17) in order to examine the four equalities, and obtain the following facts.

Lemma 2.1 *Let the OLSEs of β and ABβ in* M *and* N *be as given in* (2.5)*,* (2.6)*,* (2.7)*,* (2.9)*,* (2.15)*, and* (2.17)*, respectively. Then the following 6 assertions hold*

(2.25) $${\mathrm{OLSE}}_{M}(\beta )={\mathrm{OLSE}}_{N}(\beta )\Leftrightarrow {\left(AB\right)}^{†}=B^{†}A^{†},$$

(2.26) $$E\left({\mathrm{OLSE}}_{M}(\beta )\right)=E\left({\mathrm{OLSE}}_{N}(\beta )\right)\Leftrightarrow {\left(AB\right)}^{†}AB=B^{†}A^{†}AB,$$

(2.27) $${\mathrm{OLSE}}_{M}(AB\beta )={\mathrm{OLSE}}_{N}(AB\beta )\Leftrightarrow AB{\left(AB\right)}^{†}=ABB^{†}A^{†},$$

(2.28) $$E\left({\mathrm{OLSE}}_{M}(AB\beta )\right)=E\left({\mathrm{OLSE}}_{N}(AB\beta )\right)\Leftrightarrow AB=ABB^{†}A^{†}AB,$$

*and*

(2.29) $$\mathrm{Cov}\left({\mathrm{OLSE}}_{M}(\beta )\right)=\mathrm{Cov}\left({\mathrm{OLSE}}_{N}(\beta )\right)\Leftrightarrow \left\{ \begin{array}{c} {\left(B^{T}(A^{T}A)B]\right)}^{†}=B^{†}{\left(A^{T}A\right)}^{†}{\left(B^{T}\right)}^{†}\;and\\ {\left(AB\right)}^{†}AA^{T}{\left({\left(AB\right)}^{†}\right)}^{T}=B^{†}A^{†}A{\left(B^{†}\right)}^{T}, \end{array}\right.$$

(2.30) $$\mathrm{Cov}\left({\mathrm{OLSE}}_{M}(AB\beta )\right)=\mathrm{Cov}\left({\mathrm{OLSE}}_{N}(AB\beta )\right)\Leftrightarrow \left\{ \begin{array}{c} AB{\left(AB\right)}^{†}=ABB^{†}A^{†}{\left(ABB^{†}A^{†}\right)}^{T}\;and\\ AB{\left(AB\right)}^{†}(AA^{T})AB{\left(AB\right)}^{†}=(ABB^{†})A^{†}A{\left(ABB^{†}\right)}^{T}. \end{array}\right.$$

The above derivations and discussions show some of the main applications of generalized inverses in statistical analysis, as well as a practical motivation of approaching the ROL problem in (1.8). Obviously, the matrix equality in (2.25) is the well-known ROL for the Moore–Penrose generalized inverses of the product *AB*, and the three matrix equalities in (2.26)–(2.28) are obtained by pre- and post-multiplying the equality in (2.25) with *AB*, respectively. The equivalent statements in (2.25)–(2.28) clearly show that ROLs and their variations can directly be used to describe and solve some fundamental statistical inference problems in statistical analysis of regression models, and therefore they can be taken as a remarkable motivation and valuable explanation for approaching various matrix equalities that involve generalized inverses. It is should be pointed out that the four matrix equalities in (2.25)–(2.28) do not necessarily hold for two general matrices *A* and *B*. Thus, it is imperative to establish necessary and sufficient conditions for the four matrix equalities in (2.25)–(2.28) to hold in order to interpret and use the four statistical statements in (2.25)–(2.28).

## 3 Fundamental formulas and facts concerning generalized inverses of matrices

In this section, we introduce a series of known formulas, results, and facts from the general theory of generalized inverses of matrices that we are going to use as background materials throughout this paper. In order to describe the relationships between the matrix sets in (1.9)–(1.12), we need to use a body of matrix analytic tools, including a brief introduction to the theory of generalized inverses of matrices, which will be utilized to establish and simplify various matrix equalities that involve generalized inverses. Note from the definitions of generalized inverses of a matrix that they are in fact defined to be (common) solutions of some matrix equations. Thus analytical expressions of generalized inverses of matrices can be written as certain matrix-valued functions with one or more variable matrices. In fact, analytical formulas of generalized inverses of matrices and their functions are important issues and tools in matrix analysis. For example, the basic formulas in the following lemma can be found, e.g., in [15], [17], [65].

Lemma 3.1 *Let* $A\in C^{m\times n}$*. Then the following results hold.*

(a) *The following equalities hold*
  (3.1) $${\left(A^{†}\right)}^{⁎}={\left(A^{⁎}\right)}^{†},\;\;\;\;{\left(A^{†}\right)}^{†}=A,$$
  (3.2) $$A^{†}=A^{⁎}{\left(AA^{⁎}\right)}^{†}={\left(A^{⁎}A\right)}^{†}A^{⁎}=A^{⁎}{\left(A^{⁎}AA^{⁎}\right)}^{†}A^{⁎},$$
  (3.3) $${\left(A^{⁎}\right)}^{†}A^{⁎}={\left(AA^{†}\right)}^{⁎}=AA^{†},\;\;A^{⁎}{\left(A^{⁎}\right)}^{†}={\left(A^{†}A\right)}^{⁎}=A^{†}A,$$
  (3.4) $${\left(AA^{⁎}\right)}^{†}={\left(A^{†}\right)}^{⁎}A^{†},\;\;{\left(A^{⁎}A\right)}^{†}=A^{†}{\left(A^{†}\right)}^{⁎},\;\;{\left(AA^{⁎}A\right)}^{†}=A^{†}{\left(A^{†}\right)}^{⁎}A^{†},$$
  (3.5) $$R(A)=R(AA^{⁎})=R(AA^{⁎}A)=R(AA^{†})=R[{\left(A^{†}\right)}^{⁎}],$$
  (3.6) $$R(A^{⁎})=R(A^{⁎}A)=R(A^{⁎}AA^{⁎})=R(A^{†})=R(A^{†}A),$$
  (3.7) $$r(A)=r(A^{⁎})=r(A^{†})=r(AA^{⁎})=r(A^{⁎}A)=r(AA^{†})=r(A^{†}A).$$
(b) *The general expressions of the seven commonly-used types of generalized inverses* $A^{\left(1,3,4\right)}$*,* $A^{\left(1,2,4\right)}$*,* $A^{\left(1,2,3\right)}$*,* $A^{\left(1,4\right)}$*,* $A^{\left(1,3\right)}$*,* $A^{\left(1,2\right)}$*, and* $A^{\left(1\right)}$ *of A can be written in the following 7 matrix-valued functions*
  (3.8) $$A^{\left(1,3,4\right)}=A^{†}+F_{A}UE_{A},$$
  (3.9) $$A^{\left(1,2,4\right)}=A^{†}+A^{†}AUE_{A},$$
  (3.10) $$A^{\left(1,2,3\right)}=A^{†}+F_{A}UAA^{†},$$
  (3.11) $$A^{\left(1,4\right)}=A^{†}+UE_{A},$$
  (3.12) $$A^{\left(1,3\right)}=A^{†}+F_{A}U,$$
  (3.13) $$A^{\left(1,2\right)}=(A^{†}+F_{A}U_{1})A(A^{†}+U_{2}E_{A}),$$
  (3.14) $$A^{\left(1\right)}=A^{†}+F_{A}U_{1}+U_{2}E_{A},$$
  *where* $U,\;U_{1},\;U_{2}\in C^{n\times m}$ *are arbitrary. In particular,*
  (3.15) $$A^{\left(1,3,4\right)}\;is\;unique\Leftrightarrow r(A)=m\;\text{or}\;r(A)=n,$$
  (3.16) $$A^{\left(1,2,4\right)}\;is\;unique\Leftrightarrow A=0\;\text{or}\;r(A)=m,$$
  (3.17) $$A^{\left(1,2,3\right)}\;is\;unique\Leftrightarrow A=0\;\text{or}\;r(A)=n,$$
  (3.18) $$A^{\left(1,4\right)}\;is\;unique\Leftrightarrow r(A)=m,$$
  (3.19) $$A^{\left(1,3\right)}\;is\;unique\Leftrightarrow r(A)=n,$$
  (3.20) $$A^{\left(1,2\right)}\;is\;unique\Leftrightarrow A=0\;\text{or}\;r(A)=m=n,$$
  (3.21) $$A^{\left(1\right)}\;is\;unique\Leftrightarrow r(A)=m=n,\;namely,\;A\;is\;nonsingular.$$
  *If* r(A)=m*, the general expressions of the last seven generalized inverses of A in* (1.2) *can be written as*
  (3.22) $$A^{\left(1,3,4\right)}=A^{\left(1,2,4\right)}=A^{\left(1,4\right)}=A^{†},$$
  (3.23) $$A^{\left(1,2,3\right)}=A^{\left(1,3\right)}=A^{\left(1,2\right)}=A^{\left(1\right)}=A^{†}+F_{A}V,$$
  *where* $V\in C^{n\times m}$ *is arbitrary. If* r(A)=n*, the general expressions of the last seven generalized inverses of A in* (1.2) *can be written as*
  (3.24) $$A^{\left(1,3,4\right)}=A^{\left(1,2,3\right)}=A^{\left(1,3\right)}=A^{†},$$
  (3.25) $$A^{\left(1,2,4\right)}=A^{\left(1,4\right)}=A^{\left(1,2\right)}=A^{\left(1\right)}=A^{†}+WE_{A},$$
  *where* $W\in C^{n\times m}$ *is arbitrary.*
(c) *The following set inclusions hold*
  (3.26) $$A^{†}\in \{A^{\left(1,3,4\right)}\}\subseteq \{A^{\left(1,4\right)}\}\subseteq \{A^{\left(1\right)}\},\;A^{†}\in \{A^{\left(1,3,4\right)}\}\subseteq \{A^{\left(1,3\right)}\}\subseteq \{A^{\left(1\right)}\},$$
  (3.27) $$A^{†}\in \{A^{\left(1,2,4\right)}\}\subseteq \{A^{\left(1,4\right)}\}\subseteq \{A^{\left(1\right)}\},\;A^{†}\in \{A^{\left(1,2,4\right)}\}\subseteq \{A^{\left(1,2\right)}\}\subseteq \{A^{\left(1\right)}\},$$
  (3.28) $$A^{†}\in \{A^{\left(1,2,3\right)}\}\subseteq \{A^{\left(1,3\right)}\}\subseteq \{A^{\left(1\right)}\},\;A^{†}\in \{A^{\left(1,2,3\right)}\}\subseteq \{A^{\left(1,2\right)}\}\subseteq \{A^{\left(1\right)}\},$$
  *and following matrix set equalities hold*
  (3.29) $$\{{\left(A^{\left(1,3,4\right)}\right)}^{⁎}\}=\{{\left(A^{⁎}\right)}^{\left(1,3,4\right)}\},\;\{{\left(A^{\left(1,2,4\right)}\right)}^{⁎}\}=\{{\left(A^{⁎}\right)}^{\left(1,2,3\right)}\},$$
  (3.30) $$\{{\left(A^{\left(1,2,3\right)}\right)}^{⁎}\}=\{{\left(A^{⁎}\right)}^{\left(1,2,4\right)}\},\;\{{\left(A^{\left(1,4\right)}\right)}^{⁎}\}=\{{\left(A^{⁎}\right)}^{\left(1,3\right)}\},$$
  (3.31) $$\{{\left(A^{\left(1,3\right)}\right)}^{⁎}\}=\{{\left(A^{⁎}\right)}^{\left(1,4\right)}\},\;\{{\left(A^{\left(1,2\right)}\right)}^{⁎}\}=\{{\left(A^{⁎}\right)}^{\left(1,2\right)}\},\;\;\;\;\;\;\;\;\{{\left(A^{\left(1\right)}\right)}^{⁎}\}=\{{\left(A^{⁎}\right)}^{\left(1\right)}\}.$$
(d) [86] *The following rank equalities hold*
  (3.32) $$\max_{A^{\left(1,3,4\right)}}r(A^{\left(1,3,4\right)})=\min\{m,\;n\},\;\min_{A^{\left(1,3,4\right)}}r(A^{\left(1,3,4\right)})=r(A),$$
  (3.33) $$\max_{A^{\left(1,2,4\right)}}r(A^{\left(1,2,4\right)})=r(A),\;\min_{A^{\left(1,2,4\right)}}r(A^{\left(1,2,4\right)})=r(A),$$
  (3.34) $$\max_{A^{\left(1,2,3\right)}}r(A^{\left(1,2,3\right)})=r(A),\;\min_{A^{\left(1,2,3\right)}}r(A^{\left(1,2,3\right)})=r(A),$$
  (3.35) $$\max_{A^{\left(1,4\right)}}r(A^{\left(1,4\right)})=\min\{m,\;n\},\;\min_{A^{\left(1,4\right)}}r(A^{\left(1,4\right)})=r(A),$$
  (3.36) $$\max_{A^{\left(1,3\right)}}r(A^{\left(1,3\right)})=\min\{m,\;n\},\;\min_{A^{\left(1,3\right)}}r(A^{\left(1,3\right)})=r(A),$$
  (3.37) $$\max_{A^{\left(1,2\right)}}r(A^{\left(1,2\right)})=r(A),\;\min_{A^{\left(1,2\right)}}r(A^{\left(1,2\right)})=r(A),$$
  (3.38) $$\max_{A^{\left(1\right)}}r(A^{\left(1\right)})=\min\{m,\;n\},\;\min_{A^{\left(1\right)}}r(A^{\left(1\right)})=r(A),$$
  *and the corresponding generalized inverse satisfying* (3.32)*–*(3.38) *can be constructed by means of the singular value decompositions of A.*
(e) *The following matrix equalities hold*
  (3.39) $$AA^{\left(1,3,4\right)}=AA^{\left(1,2,3\right)}=AA^{\left(1,3\right)}=AA^{†}\;\;\text{is unique},$$
  (3.40) $$AA^{\left(1,2,4\right)}=AA^{\left(1,4\right)}=AA^{\left(1,2\right)}=AA^{\left(1\right)}=AA^{†}+AUE_{A},$$
  (3.41) $$A^{\left(1,3,4\right)}A=A^{\left(1,2,4\right)}A=A^{\left(1,4\right)}A=A^{†}A\;\;\text{is unique},$$
  (3.42) $$A^{\left(1,2,3\right)}A=A^{\left(1,3\right)}A=A^{\left(1,2\right)}A=A^{\left(1\right)}A=A^{†}A+F_{A}UA,$$
  *where* $U\in C^{n\times m}$ *is arbitrary. In particular,*
  (3.43) $$AA^{\left(1,2,4\right)}=AA^{\left(1,4\right)}=AA^{\left(1,2\right)}=AA^{\left(1\right)}\;\text{is unique}\Leftrightarrow A=0\;\text{or}\;r(A)=m,$$
  (3.44) $$A^{\left(1,2,3\right)}A=A^{\left(1,3\right)}A=A^{\left(1,2\right)}A=A^{\left(1\right)}A\;\text{is unique}\Leftrightarrow A=0\;\text{or}\;r(A)=n.$$
  *Besides, the following matrix equalities hold*
  (3.45) $$A^{\left(1\right)}AA^{\left(1\right)}=A^{\left(1,2\right)},\;\;A^{\left(1\right)}AA^{\left(1,3\right)}=A^{\left(1,2,3\right)},\;\;A^{\left(1,4\right)}AA^{\left(1\right)}=A^{\left(1,2,4\right)},\;\;\;\;A^{\left(1,4\right)}AA^{\left(1,3\right)}=A^{†}.$$
(f) *The following set inclusions*
  (3.46) $$PA^{†}Q\in \{PA^{\left(1,3,4\right)}Q\}\subseteq \{PA^{\left(1,4\right)}Q\}\subseteq \{PA^{\left(1\right)}Q\},$$
  (3.47) $$PA^{†}Q\in \{PA^{\left(1,3,4\right)}Q\}\subseteq \{PA^{\left(1,3\right)}Q\}\subseteq \{PA^{\left(1\right)}Q\},$$
  (3.48) $$PA^{†}Q\in \{PA^{\left(1,2,4\right)}Q\}\subseteq \{PA^{\left(1,4\right)}Q\}\subseteq \{PA^{\left(1\right)}Q\},$$
  (3.49) $$PA^{†}Q\in \{PA^{\left(1,2,4\right)}Q\}\subseteq \{PA^{\left(1,2\right)}Q\}\subseteq \{PA^{\left(1\right)}Q\},$$
  (3.50) $$PA^{†}Q\in \{PA^{\left(1,2,3\right)}Q\}\subseteq \{PA^{\left(1,3\right)}Q\}\subseteq \{PA^{\left(1\right)}Q\},$$
  (3.51) $$PA^{†}Q\in \{PA^{\left(1,2,3\right)}Q\}\subseteq \{PA^{\left(1,2\right)}Q\}\subseteq \{PA^{\left(1\right)}Q\}$$
  *hold for any matrices P and Q.*
(g) $R(A)=N(I_{m}-AA^{\left(1\right)})$ *and* $N(A)=N(I_{m}-A^{\left(1\right)}A)$ *hold for all* $A^{\left(1\right)}$*.*

Lemma 3.2 **[85]**

*Let* $A\in C^{m\times n}$ *and* $G\in C^{n\times m}$*. Then*

(3.52) $$\min_{A^{\left(1\right)}}\;r(A^{\left(1\right)}-G)=r(A-AGA),$$

(3.53) $$\min_{A^{\left(1,2\right)}}r(A^{\left(1,2\right)}-G)=\max\{r(A-AGA),\;r(G)+r(A)-r(GA)-r(AG)\},$$

(3.54) $$\min_{A^{\left(1,3\right)}}r(A^{\left(1,3\right)}-G)=r(A^{⁎}AG-A^{⁎}),$$

(3.55) $$\min_{A^{\left(1,4\right)}}r(A^{\left(1,4\right)}-G)=r(GAA^{⁎}-A^{⁎}),$$

(3.56) $$\min_{A^{\left(1,2,3\right)}}r(A^{\left(1,2,3\right)}-G)=r(A^{⁎}AG-A^{⁎})+r\left[\begin{array}{c} A^{⁎}\\ G \end{array}\right]-r\left[\begin{array}{c} A^{⁎}\\ AG \end{array}\right],$$

(3.57) $$\min_{A^{\left(1,2,4\right)}}r(A^{\left(1,2,4\right)}-G)=r(GAA^{⁎}-A^{⁎})+r[A^{⁎},\;G]-r[A^{⁎},\;GA],$$

(3.58) $$\min_{A^{\left(1,3,4\right)}}r(A^{\left(1,3,4\right)}-G)=r(A^{⁎}AG-A^{⁎})+r(GAA^{⁎}-A^{⁎})-r(A-AGA),$$

(3.59) $$r(A^{†}-G)=r\left[\begin{array}{cc} A^{⁎}AA^{⁎} & A^{⁎}\\ A^{⁎} & G \end{array}\right]-r(A).$$

*If* $R(G)\subseteq R(A^{⁎})$*, then*

(3.60) $$r(A^{†}-G)=r(A^{⁎}-A^{⁎}AG).$$

*If* $R(G^{⁎})\subseteq R(A)$*, then*

(3.61) $$r(A^{†}-G)=r(A^{⁎}-GAA^{⁎}).$$

*If* $R(G)\subseteq R(A^{⁎})$ *and* $R(G^{⁎})\subseteq R(A)$*, then*

(3.62) $$r(A^{†}-G)=r(A-AGA).$$

*In particular,*

(3.63) $$G\in \{A^{\left(1\right)}\}\Leftrightarrow AGA=A,$$

(3.64) $$G\in \{A^{\left(1,2\right)}\}\Leftrightarrow AGA=A\;\text{and}\;r(G)=r(A),$$

(3.65) $$G\in \{A^{\left(1,3\right)}\}\Leftrightarrow AG=AA^{†}\Leftrightarrow A^{⁎}AG=A^{⁎},$$

(3.66) $$G\in \{A^{\left(1,4\right)}\}\Leftrightarrow GA=A^{†}A\Leftrightarrow GAA^{⁎}=A^{⁎},$$

(3.67) $$G\in \{A^{\left(1,2,3\right)}\}\Leftrightarrow A^{⁎}AG=A^{⁎}\;\text{and}\;r(G)=r(A)\Leftrightarrow A^{⁎}AG=A^{⁎}\;\text{and}\;GE_{A}=0,$$

(3.68) $$G\in \{A^{\left(1,2,4\right)}\}\Leftrightarrow GAA^{⁎}=A^{⁎}\;\text{and}\;r(G)=r(A)\Leftrightarrow GAA^{⁎}=A^{⁎}\;\text{and}\;F_{A}G=0,$$

(3.69) $$G\in \{A^{\left(1,3,4\right)}\}\Leftrightarrow A^{⁎}AG=A^{⁎}\;\text{and}\;GAA^{⁎}=A^{⁎},$$

(3.70) $$G=A^{†}\Leftrightarrow G\in \{A^{\left(1,3\right)}\},\;G\in \{A^{\left(1,4\right)}\},\;\text{and}\;r(G)=r(A)\Leftrightarrow AG=AA^{†},\;GA=A^{†}A,\;\text{and}\;r(G)=r(A)\Leftrightarrow A^{⁎}AG=A^{⁎},\;GAA^{⁎}=A^{⁎},\;\text{and}\;r(G)=r(A)\Leftrightarrow AG=AA^{†},\;GA=A^{†}A,\;GE_{A}=0,\;\text{and}\;F_{A}G=0.$$

In view of the facts in Lemma 3.2, we describe below how to use the MRM in the study of set inclusions for generalized inverses of matrices.

Lemma 3.3 *Let* $A\in C^{m\times n}$*, and* $f\left(A_{1}^{\left(i_{1},\ldots ,j_{1}\right)},A_{2}^{\left(i_{2},\ldots ,j_{2}\right)},\ldots ,A_{k}^{\left(i_{k},\ldots ,j_{k}\right)}\right)\in C^{n\times m}$ *be a matrix expression composed of* $A^{\left(i_{1},\ldots ,j_{1}\right)}$*,* $A_{2}^{\left(i_{2},\ldots ,j_{2}\right)}$*,* …*,* $A_{k}^{\left(i_{k},\ldots ,j_{k}\right)}$*. Then the following results hold.*

(a) $\{A^{\left(1\right)}\}⊇D_{f}$ *if and only if*
  $$\max_{A_{1}^{\left(i_{1},\ldots ,j_{1}\right)},\ldots ,A_{k}^{\left(i_{k},\ldots ,j_{k}\right)}}r(A-AfA)=0.$$
(b) $\{A^{\left(1,2\right)}\}⊇D_{f}$ *if and only if*
  $$\max_{A_{1}^{\left(i_{1},\ldots ,j_{1}\right)},\ldots ,A_{k}^{\left(i_{k},\ldots ,j_{k}\right)}}r(A-AfA)=0\;\;\text{and}\;\;\max_{A_{1}^{\left(i_{1},\ldots ,j_{1}\right)},\ldots ,A_{k}^{\left(i_{k},\ldots ,j_{k}\right)}}r(f)=r(A).$$
(c) $\{A^{\left(1,3\right)}\}⊇D_{f}$ *if and only if*
  $$\max_{A_{1}^{\left(i_{1},\ldots ,j_{1}\right)},\ldots ,A_{k}^{\left(i_{k},\ldots ,j_{k}\right)}}r(A^{⁎}-A^{⁎}Af)=0.$$
(d) $\{A^{\left(1,4\right)}\}⊇D_{f}$ *if and only if*
  $$\max_{A_{1}^{\left(i_{1},\ldots ,j_{1}\right)},\ldots ,A_{k}^{\left(i_{k},\ldots ,j_{k}\right)}}r(A^{⁎}-fAA^{⁎})=0.$$
(e) $\{A^{\left(1,2,3\right)}\}⊇D_{f}$ *if and only if*
  $$\max_{A_{1}^{\left(i_{1},\ldots ,j_{1}\right)},\ldots ,A_{k}^{\left(i_{k},\ldots ,j_{k}\right)}}r(A^{⁎}-A^{⁎}Af)=0\;\text{and}\;\;\max_{A_{1}^{\left(i_{1},\ldots ,j_{1}\right)},\ldots ,A_{k}^{\left(i_{k},\ldots ,j_{k}\right)}}r(f)=r(A).$$
(f) $\{A^{\left(1,2,4\right)}\}⊇D_{f}$ *if and only if*
  $$\max_{A_{1}^{\left(i_{1},\ldots ,j_{1}\right)},\ldots ,A_{k}^{\left(i_{k},\ldots ,j_{k}\right)}}r(A^{⁎}-fAA^{⁎})=0\;\;\text{and}\;\;\max_{A_{1}^{\left(i_{1},\ldots ,j_{1}\right)},\ldots ,A_{k}^{\left(i_{k},\ldots ,j_{k}\right)}}r(f)=r(A).$$
(g) $\{A^{\left(1,3,4\right)}\}⊇D_{f}$ ⇔ $\{A^{\left(1,3\right)}\}⊇D_{f}$ *and* $\{A^{\left(1,4\right)}\}⊇D_{f}$*.*
(h) $A^{†}=D_{f}$ ⇔ $\{A^{\left(1,2,3\right)}\}⊇D_{f}$ *and* $\{A^{\left(1,2,4\right)}\}⊇D_{f}$ ⇔ *f is invariant and* $A^{†}=f\left(A_{1}^{†},A_{2}^{†},\ldots ,A_{k}^{†}\right)$*.*

The assertions in Lemma 3.3 show strong requirements to establish various closed-formulas for calculating ranks of matrices and their generalized inverses, namely, if certain rank formulas associated with (1.9)–(1.11) are given, they can be used to derive necessary and sufficient conditions for (1.9)–(1.11) to hold, respectively.

Let $A\in C^{m\times n}$ and $B\in C^{n\times p}$. Then the following matrix expressions

(3.71) $${\left(AB\right)}^{\left(1,3,4\right)}={\left(AB\right)}^{†}+F_{AB}VE_{AB},$$

(3.72) $${\left(AB\right)}^{\left(1,2,4\right)}={\left(AB\right)}^{†}+{\left(AB\right)}^{†}(AB)VE_{AB},$$

(3.73) $${\left(AB\right)}^{\left(1,2,3\right)}={\left(AB\right)}^{†}+F_{AB}V(AB){\left(AB\right)}^{†},$$

(3.74) $${\left(AB\right)}^{\left(1,4\right)}={\left(AB\right)}^{†}+WE_{AB},$$

(3.75) $${\left(AB\right)}^{\left(1,3\right)}={\left(AB\right)}^{†}+F_{AB}V,$$

(3.76) $${\left(AB\right)}^{\left(1,2\right)}=\left({\left(AB\right)}^{†}+F_{AB}V\right)AB\left({\left(AB\right)}^{†}+WE_{AB}\right),$$

(3.77) $${\left(AB\right)}^{\left(1\right)}={\left(AB\right)}^{†}+F_{AB}V+WE_{AB},$$

and

(3.78) $$AB{\left(AB\right)}^{\left(1,3,4\right)}=AB{\left(AB\right)}^{\left(1,2,3\right)}=AB{\left(AB\right)}^{\left(1,3\right)}=AB{\left(AB\right)}^{†},$$

(3.79) $$AB{\left(AB\right)}^{\left(1,2,4\right)}=AB{\left(AB\right)}^{\left(1,4\right)}=AB{\left(AB\right)}^{\left(1,2\right)}=AB{\left(AB\right)}^{\left(1\right)}=AB{\left(AB\right)}^{†}+ABVE_{AB},$$

(3.80) $${\left(AB\right)}^{\left(1,3,4\right)}AB={\left(AB\right)}^{\left(1,2,4\right)}AB={\left(AB\right)}^{\left(1,4\right)}AB={\left(AB\right)}^{†}AB,$$

(3.81) $${\left(AB\right)}^{\left(1,2,3\right)}AB={\left(AB\right)}^{\left(1,3\right)}AB={\left(AB\right)}^{\left(1,2\right)}AB={\left(AB\right)}^{\left(1\right)}AB={\left(AB\right)}^{†}AB+F_{AB}VAB,$$

(3.82) $$B{\left(AB\right)}^{\left(1,3,4\right)}A=B{\left(AB\right)}^{†}A+BF_{AB}VE_{AB}A,$$

(3.83) $$B{\left(AB\right)}^{\left(1,2,4\right)}A=B{\left(AB\right)}^{†}A+B{\left(AB\right)}^{†}(AB)VE_{AB}A,$$

(3.84) $$B{\left(AB\right)}^{\left(1,2,3\right)}A=B{\left(AB\right)}^{†}A+BF_{AB}V(AB){\left(AB\right)}^{†}A,$$

(3.85) $$B{\left(AB\right)}^{\left(1,4\right)}A=B{\left(AB\right)}^{†}A+BWE_{AB}A,$$

(3.86) $$B{\left(AB\right)}^{\left(1,3\right)}A=B{\left(AB\right)}^{†}A+BF_{AB}VA,$$

(3.87) $$B{\left(AB\right)}^{\left(1,2\right)}A=\left(B{\left(AB\right)}^{†}+BF_{AB}V\right)AB\left({\left(AB\right)}^{†}A+WE_{AB}A\right),$$

(3.88) $$B{\left(AB\right)}^{\left(1\right)}A=B{\left(AB\right)}^{†}A+BF_{AB}VB+AWE_{AB}A$$

hold by (3.8)–(3.14), where the two matrices *V* and *W* are arbitrary.

It is natural to see from the ROL in (1.8) that the starting point of the investigation of the ROL is to list out algebraic properties of the product $B^{\left(s_{2},\ldots ,t_{2}\right)}A^{\left(s_{1},\ldots ,t_{1}\right)}$ that are necessary to identify the ROL. On the other hand, a conventional technique in the study of matrix expressions that involve generalized inverses is representing the expressions in certain marginal matrix-valued functions that involve variable matrices. Because generalized inverses of a matrix are linear or multilinear matrix-valued functions (MVFs), the products $B^{\left(s_{2},\ldots ,t_{2}\right)}A^{\left(s_{1},\ldots ,t_{1}\right)}$ can be rewritten as 63 distinct linear or multilinear MVFs for the eight commonly-used types of generalized inverses of *A* and *B*, respectively, except the unique case $B^{†}A^{†}$ for the Moore–Penrose inverses. For convenience of discussion, we present all the 63 MVFs in the following list:

(3.89) $$B^{†}A^{\left(1,3,4\right)}=B^{†}A^{†}+B^{†}F_{A}UE_{A},$$

(3.90) $$B^{†}A^{\left(1,2,4\right)}=B^{†}A^{†}+B^{†}A^{†}AUE_{A},$$

(3.91) $$B^{†}A^{\left(1,2,3\right)}=B^{†}A^{†}+B^{†}F_{A}UAA^{†},$$

(3.92) $$B^{†}A^{\left(1,4\right)}=B^{†}A^{†}+B^{†}UE_{A},$$

(3.93) $$B^{†}A^{\left(1,3\right)}=B^{†}A^{†}+B^{†}F_{A}U,$$

(3.94) $$B^{†}A^{\left(1,2\right)}=(B^{†}A^{†}A+B^{†}F_{A}U_{1}A)(A^{†}+A^{†}AU_{2}E_{A}),$$

(3.95) $$B^{†}A^{\left(1\right)}=B^{†}A^{†}+B^{†}F_{A}U_{1}+B^{†}U_{2}E_{A},$$

(3.96) $$B^{\left(1,3,4\right)}A^{†}=B^{†}A^{†}+F_{B}VE_{B}A^{†},$$

(3.97) $$B^{\left(1,3,4\right)}A^{\left(1,3,4\right)}=(B^{†}+F_{B}VE_{B})(A^{†}+F_{A}UE_{A}),$$

(3.98) $$B^{\left(1,3,4\right)}A^{\left(1,2,4\right)}=(B^{†}+F_{B}VE_{B})(A^{†}+A^{†}AUE_{A}),$$

(3.99) $$B^{\left(1,3,4\right)}A^{\left(1,2,3\right)}=(B^{†}+F_{B}VE_{B})(A^{†}+F_{A}UAA^{†}),$$

(3.100) $$B^{\left(1,3,4\right)}A^{\left(1,4\right)}=(B^{†}+F_{B}VE_{B})(A^{†}+UE_{A}),$$

(3.101) $$B^{\left(1,3,4\right)}A^{\left(1,3\right)}=(B^{†}+F_{B}VE_{B})(A^{†}+F_{A}U),$$

(3.102) $$B^{\left(1,3,4\right)}A^{\left(1,2\right)}=(B^{†}+F_{B}VE_{B})(A^{†}A+F_{A}U_{1}A)(A^{†}+A^{†}AU_{2}E_{A}),$$

(3.103) $$B^{\left(1,3,4\right)}A^{\left(1\right)}=(B^{†}+F_{B}VE_{B})(A^{†}+F_{A}U_{1}+U_{2}E_{A}),$$

(3.104) $$B^{\left(1,2,4\right)}A^{†}=B^{†}A^{†}+B^{†}BVE_{B}A^{†},$$

(3.105) $$B^{\left(1,2,4\right)}A^{\left(1,3,4\right)}=(B^{†}+B^{†}BVE_{B})(A^{†}+F_{A}UE_{A}),$$

(3.106) $$B^{\left(1,2,4\right)}A^{\left(1,2,4\right)}=(B^{†}+B^{†}BVE_{B})(A^{†}+A^{†}AUE_{A}),$$

(3.107) $$B^{\left(1,2,4\right)}A^{\left(1,2,3\right)}=(B^{†}+B^{†}BVE_{B})(A^{†}+F_{A}UAA^{†}),$$

(3.108) $$B^{\left(1,2,4\right)}A^{\left(1,4\right)}=(B^{†}+B^{†}BVE_{B})(A^{†}+UE_{A}),$$

(3.109) $$B^{\left(1,2,4\right)}A^{\left(1,3\right)}=(B^{†}+B^{†}BVE_{B})(A^{†}+F_{A}U),$$

(3.110) $$B^{\left(1,2,4\right)}A^{\left(1,2\right)}=(B^{†}+B^{†}BVE_{B})(A^{†}A+F_{A}U_{1}A)(A^{†}+A^{†}AU_{2}E_{A}),$$

(3.111) $$B^{\left(1,2,4\right)}A^{\left(1\right)}=(B^{†}+B^{†}BVE_{B})(A^{†}+F_{A}U_{1}+U_{2}E_{A}),$$

(3.112) $$B^{\left(1,2,3\right)}A^{†}=B^{†}A^{†}+F_{B}VBB^{†}A^{†},$$

(3.113) $$B^{\left(1,2,3\right)}A^{\left(1,3,4\right)}=(B^{†}+F_{B}VBB^{†})(A^{†}+F_{A}UE_{A}),$$

(3.114) $$B^{\left(1,2,3\right)}A^{\left(1,2,4\right)}=(B^{†}+F_{B}VBB^{†})(A^{†}+A^{†}AUE_{A}),$$

(3.115) $$B^{\left(1,2,3\right)}A^{\left(1,2,3\right)}=(B^{†}+F_{B}VBB^{†})(A^{†}+F_{A}UAA^{†}),$$

(3.116) $$B^{\left(1,2,3\right)}A^{\left(1,4\right)}=(B^{†}+F_{B}VBB^{†})(A^{†}+UE_{A}),$$

(3.117) $$B^{\left(1,2,3\right)}A^{\left(1,3\right)}=(B^{†}+F_{B}VBB^{†})(A^{†}+F_{A}U),$$

(3.118) $$B^{\left(1,2,3\right)}A^{\left(1,2\right)}=(B^{†}+F_{B}VBB^{†})(A^{†}A+F_{A}U_{1}A)(A^{†}+A^{†}AU_{2}E_{A}),$$

(3.119) $$B^{\left(1,2,3\right)}A^{\left(1\right)}=(B^{†}+F_{B}VBB^{†})(A^{†}+F_{A}U_{1}+U_{2}E_{A}),$$

(3.120) $$B^{\left(1,4\right)}A^{†}=B^{†}A^{†}+VE_{B}A^{†},$$

(3.121) $$B^{\left(1,4\right)}A^{\left(1,3,4\right)}=(B^{†}+VE_{B})(A^{†}+F_{A}UE_{A}),$$

(3.122) $$B^{\left(1,4\right)}A^{\left(1,2,4\right)}=(B^{†}+VE_{B})(A^{†}+A^{†}AUE_{A}),$$

(3.123) $$B^{\left(1,4\right)}A^{\left(1,2,3\right)}=(B^{†}+VE_{B})(A^{†}+F_{A}UAA^{†}),$$

(3.124) $$B^{\left(1,4\right)}A^{\left(1,4\right)}=(B^{†}+VE_{B})(A^{†}+UE_{A}),$$

(3.125) $$B^{\left(1,4\right)}A^{\left(1,3\right)}=(B^{†}+VE_{B})(A^{†}+F_{A}U),$$

(3.126) $$B^{\left(1,4\right)}A^{\left(1,2\right)}=(B^{†}+VE_{B})(A^{†}A+F_{A}U_{1}A)(A^{†}+A^{†}AU_{2}E_{A}),$$

(3.127) $$B^{\left(1,4\right)}A^{\left(1\right)}=(B^{†}+VE_{B})(A^{†}+F_{A}U_{1}+U_{2}E_{A}),$$

(3.128) $$B^{\left(1,3\right)}A^{†}=B^{†}A^{†}+F_{B}VA^{†},$$

(3.129) $$B^{\left(1,3\right)}A^{\left(1,3,4\right)}=(B^{†}+F_{B}V)(A^{†}+F_{A}UE_{A}),$$

(3.130) $$B^{\left(1,3\right)}A^{\left(1,2,4\right)}=(B^{†}+F_{B}V)(A^{†}+A^{†}AUE_{A}),$$

(3.131) $$B^{\left(1,3\right)}A^{\left(1,2,3\right)}=(B^{†}+F_{B}V)(A^{†}+F_{A}UAA^{†}),$$

(3.132) $$B^{\left(1,3\right)}A^{\left(1,4\right)}=(B^{†}+F_{B}V)(A^{†}+UE_{A}),$$

(3.133) $$B^{\left(1,3\right)}A^{\left(1,3\right)}=(B^{†}+F_{B}V)(A^{†}+F_{A}U),$$

(3.134) $$B^{\left(1,3\right)}A^{\left(1,2\right)}=(B^{†}+F_{B}V)(A^{†}A+F_{A}U_{1}A)(A^{†}+A^{†}AU_{2}E_{A}),$$

(3.135) $$B^{\left(1,3\right)}A^{\left(1\right)}=(B^{†}+F_{B}V)(A^{†}+F_{A}U_{1}+U_{2}E_{A}),$$

(3.136) $$B^{\left(1,2\right)}A^{†}=(B^{†}+F_{B}V_{1}BB^{†})(BB^{†}A^{†}+BV_{2}E_{B}A^{†}),$$

(3.137) $$B^{\left(1,2\right)}A^{\left(1,3,4\right)}=(B^{†}+F_{B}V_{1}BB^{†})(BB^{†}+BV_{2}E_{B})(A^{†}+F_{A}UE_{A}),$$

(3.138) $$B^{\left(1,2\right)}A^{\left(1,2,4\right)}=(B^{†}+F_{B}V_{1}BB^{†})(BB^{†}+BV_{2}E_{B})(A^{†}+A^{†}AUE_{A}),$$

(3.139) $$B^{\left(1,2\right)}A^{\left(1,2,3\right)}=(B^{†}+F_{B}V_{1}BB^{†})(BB^{†}+BV_{2}E_{B})(A^{†}+F_{A}UAA^{†}),$$

(3.140) $$B^{\left(1,2\right)}A^{\left(1,4\right)}=(B^{†}+F_{B}V_{1}BB^{†})(BB^{†}+BV_{2}E_{B})(A^{†}+UE_{A}),$$

(3.141) $$B^{\left(1,2\right)}A^{\left(1,3\right)}=(B^{†}+F_{B}V_{1}BB^{†})(BB^{†}+BV_{2}E_{B})(A^{†}+F_{A}U),$$

(3.142) $$B^{\left(1,2\right)}A^{\left(1,2\right)}=(B^{†}+F_{B}V_{1}BB^{†})(BB^{†}+BV_{2}E_{B})(A^{†}A+F_{A}U_{1}A)(A^{†}+A^{†}AU_{2}E_{A}),$$

(3.143) $$B^{\left(1,2\right)}A^{\left(1\right)}=(B^{†}+F_{B}V_{1}BB^{†})(BB^{†}+BV_{2}E_{B})(A^{†}+F_{A}U_{1}+U_{2}E_{A}),$$

(3.144) $$B^{\left(1\right)}A^{†}=B^{†}A^{†}+F_{B}V_{1}A^{†}+V_{2}E_{B}A^{†},$$

(3.145) $$B^{\left(1\right)}A^{\left(1,3,4\right)}=(B^{†}+F_{B}V_{1}+V_{2}E_{B})(A^{†}+F_{A}UE_{A}),$$

(3.146) $$B^{\left(1\right)}A^{\left(1,2,4\right)}=(B^{†}+F_{B}V_{1}+V_{2}E_{B})(A^{†}+A^{†}AUE_{A}),$$

(3.147) $$B^{\left(1\right)}A^{\left(1,2,3\right)}=(B^{†}+F_{B}V_{1}+V_{2}E_{B})(A^{†}+F_{A}UAA^{†}),$$

(3.148) $$B^{\left(1\right)}A^{\left(1,4\right)}=(B^{†}+F_{B}V_{1}+V_{2}E_{B})(A^{†}+UE_{A}),$$

(3.149) $$B^{\left(1\right)}A^{\left(1,3\right)}=(B^{†}+F_{B}V_{1}+V_{2}E_{B})(A^{†}+F_{A}U),$$

(3.150) $$B^{\left(1\right)}A^{\left(1,2\right)}=(B^{†}+F_{B}V_{1}+V_{2}E_{B})(A^{†}A+F_{A}U_{1}A)(A^{†}+A^{†}AU_{2}E_{A}),$$

(3.151) $$B^{\left(1\right)}A^{\left(1\right)}=(B^{†}+F_{B}V_{1}+V_{2}E_{B})(A^{†}+F_{A}U_{1}+U_{2}E_{A}),$$

where *V*, $V_{1}$, $V_{2}$, *U*, $U_{1}$, $U_{2}$ are arbitrary matrices of appropriate sizes. Correspondingly, the 64 products $ABB^{\left(s_{2},\ldots ,t_{2}\right)}A^{\left(s_{1},\ldots ,t_{1}\right)}$ for the eight commonly-used types of generalized inverses of *A* and *B* except $ABB^{†}A^{†}$ are divided into the following groups

(3.152) $$ABB^{†}A^{\left(1,3,4\right)}=ABB^{†}A^{†}+ABB^{†}F_{A}UE_{A},$$

(3.153) $$ABB^{†}A^{\left(1,2,4\right)}=ABB^{†}A^{†}+ABB^{†}A^{†}AUE_{A},$$

(3.154) $$ABB^{†}A^{\left(1,2,3\right)}=ABB^{†}A^{†}+ABB^{†}F_{A}UAA^{†},$$

(3.155) $$ABB^{†}A^{\left(1,4\right)}=ABB^{†}A^{†}+ABB^{†}UE_{A},$$

(3.156) $$ABB^{†}A^{\left(1,3\right)}=ABB^{†}A^{†}+ABB^{†}F_{A}U,$$

(3.157) $$ABB^{†}A^{\left(1,2\right)}=ABB^{†}(A^{†}+F_{A}U_{1})A(A^{†}+U_{2}E_{A}),$$

(3.158) $$ABB^{†}A^{\left(1\right)}=ABB^{†}(A^{†}+F_{A}U_{1}+U_{2}E_{A}),$$

(3.159) $$ABB^{\left(1\right)}A^{†}=ABB^{†}A^{†}+ABVE_{B}A^{†},$$

(3.160) $$ABB^{\left(1\right)}A^{\left(1,3,4\right)}=AB(B^{†}+VE_{B})(A^{†}+F_{A}UE_{A}),$$

(3.161) $$ABB^{\left(1\right)}A^{\left(1,2,4\right)}=AB(B^{†}+VE_{B})(A^{†}+A^{†}AUE_{A}),$$

(3.162) $$ABB^{\left(1\right)}A^{\left(1,2,3\right)}=AB(B^{†}+VE_{B})(A^{†}+F_{A}UAA^{†}),$$

(3.163) $$ABB^{\left(1\right)}A^{\left(1,4\right)}=AB(B^{†}+VE_{B})(A^{†}+UE_{A}),$$

(3.164) $$ABB^{\left(1\right)}A^{\left(1,3\right)}=AB(B^{†}+VE_{B})(A^{†}+F_{A}U),$$

(3.165) $$ABB^{\left(1\right)}A^{\left(1,2\right)}=AB(B^{†}+VE_{B})(A^{†}+F_{A}U_{1})A(A^{†}+U_{2}E_{A}),$$

(3.166) $$ABB^{\left(1\right)}A^{\left(1\right)}=AB(B^{†}+VE_{B})(A^{†}+F_{A}U_{1}+U_{2}E_{A}),$$

where $V,\;U,\;U_{1}$, and $U_{2}$ are arbitrary matrices; the 64 products $B^{\left(s_{2},\ldots ,t_{2}\right)}A^{\left(s_{1},\ldots ,t_{1}\right)}AB$ except $B^{†}A^{†}AB$ are distinctively classified as the following groups

(3.167) $$B^{\left(1,3,4\right)}A^{†}AB=B^{†}A^{†}AB+F_{B}VE_{B}A^{†}AB,$$

(3.168) $$B^{\left(1,2,4\right)}A^{†}AB=B^{†}A^{†}AB+B^{†}BVE_{B}A^{†}AB,$$

(3.169) $$B^{\left(1,2,3\right)}A^{†}AB=B^{†}A^{†}AB+F_{B}VBB^{†}A^{†}AB,$$

(3.170) $$B^{\left(1,4\right)}A^{†}AB=B^{†}A^{†}AB+VE_{B}A^{†}AB,$$

(3.171) $$B^{\left(1,3\right)}A^{†}AB=B^{†}A^{†}AB+F_{B}VA^{†}AB,$$

(3.172) $$B^{\left(1,2\right)}A^{†}AB=(B^{†}+F_{B}V_{1})B(B^{†}+V_{2}E_{B})A^{†}AB,$$

(3.173) $$B^{\left(1\right)}A^{†}AB=(B^{†}+F_{B}V_{1}+V_{2}E_{B})A^{†}AB,$$

(3.174) $$B^{†}A^{\left(1\right)}AB=B^{†}(A^{†}+F_{A}U)AB,$$

(3.175) $$B^{\left(1,3,4\right)}A^{\left(1\right)}AB=(B^{†}+F_{B}VE_{B})(A^{†}+F_{A}U)AB,$$

(3.176) $$B^{\left(1,2,4\right)}A^{\left(1\right)}AB=(B^{†}+B^{†}BVE_{B})(A^{†}+F_{A}U)AB,$$

(3.177) $$B^{\left(1,2,3\right)}A^{\left(1\right)}AB=(B^{†}+F_{B}VBB^{†})(A^{†}+F_{A}U)AB,$$

(3.178) $$B^{\left(1,4\right)}A^{\left(1\right)}AB=(B^{†}+VE_{B})(A^{†}+F_{A}U)AB,$$

(3.179) $$B^{\left(1,3\right)}A^{\left(1\right)}AB=(B^{†}+F_{B}V)(A^{†}+F_{A}U)AB,$$

(3.180) $$B^{\left(1,2\right)}A^{\left(1\right)}AB=(B^{†}+F_{B}V_{1})B(B^{†}+V_{2}E_{B})(A^{†}+F_{A}U)AB,$$

(3.181) $$B^{\left(1\right)}A^{\left(1\right)}AB=(B^{†}+F_{B}V_{1}+V_{2}E_{B})(A^{†}+F_{A}U)AB,$$

where $U,\;V,\;V_{1}$, and $V_{2}$ are variable matrices; the 64 products $ABB^{\left(s_{2},\ldots ,t_{2}\right)}A^{\left(s_{1},\ldots ,t_{1}\right)}AB$ except $ABB^{†}A^{†}AB$ for the eight commonly-used types of generalized inverses of *A* and *B* are distinctively divided into the following three groups:

(3.182) $$ABB^{†}A^{\left(1\right)}AB=ABB^{†}A^{†}AB+ABB^{†}F_{A}UAB,$$

(3.183) $$ABB^{\left(1\right)}A^{†}AB=ABB^{†}A^{†}AB+ABVE_{B}A^{†}AB,$$

(3.184) $$ABB^{\left(1\right)}A^{\left(1\right)}AB=(ABB^{†}+ABVE_{B})(A^{†}AB+F_{A}UAB),$$

where *U* and *V* are variable matrices. It is helpful to display all the matrix products together, so that we can approach the right-hand sides of these matrix-valued functions uniformly.

## 4 Miscellaneous formulas for calculating ranks of matrices and their generalized inverses

One of the most significant developments of matrix theory in past several decades has been the establishment of many analytical formulas for calculating ranks of matrix expressions that involve variable matrices and generalized inverses of matrices. The salient values of these rank formulas have been demonstrated in solving various complicated algebraic and computational problems in matrix analysis and applications. In this section, we present a collection of known and new formulas, results, and facts concerning ranks of matrices and their generalized inverses, which are solid basis of the MRM and BMM, and are indispensable to make the paper self-contained when establishing and simplifying various matrix expressions and equalities associated with ROL problems.

Lemma 4.1 **[90]**

*Let* $A\in C^{m\times n}$ *and* $B\in C^{n\times p}$*, and integer* k⩾1*.*

(a) *If* $R(A^{⁎}AB)\subseteq R(B)$*, then*
  (4.1) $$R({\left(A^{⁎}A\right)}^{k}B)\subseteq R(B)\;\text{and}\;R({\left(AA^{⁎}\right)}^{k}AB)=R(AB).$$
(b) *If* $R(BB^{⁎}A^{⁎})\subseteq R(A^{⁎})$*, then*
  (4.2) $$R({\left(BB^{⁎}\right)}^{k}A^{⁎})\subseteq R(A^{⁎})\;\text{and}\;R({\left(B^{⁎}B\right)}^{k}{\left(AB\right)}^{⁎})=R({\left(AB\right)}^{⁎}).$$

Lemma 4.2 *Let* $P_{1}\in C^{m\times p_{1}}$*,* $P_{2}\in C^{m\times p_{2}}$*,* $Q_{1}\in C^{m\times q_{1}}$*, and* $Q_{2}\in C^{m\times q_{2}}$*. Then*

(4.3) $$R(P_{1})=R(Q_{1})\;\text{and}\;R(P_{2})=R(Q_{2})\Rightarrow r[P_{1},\;P_{2}]=r[Q_{1},\;Q_{2}].$$

Lemma 4.3 **[57]**

*Let* $A\in C^{m\times n},\;B\in C^{m\times k}$*,* $C\in C^{l\times n}$*, and* $D\in C^{l\times k}$*. Then*

(4.4) $$r[A,\;B]=r(A)+r(E_{A}B)=r(B)+r(E_{B}A),$$

(4.5) $$r\left[\begin{array}{c} A\\ C \end{array}\right]=r(A)+r(CF_{A})=r(C)+r(AF_{C}),$$

(4.6) $$r\left[\begin{array}{cc} A & B\\ C & 0 \end{array}\right]=r(B)+r(C)+r(E_{B}AF_{C}),$$

(4.7) $$r\left[\begin{array}{cc} A & B\\ C & D \end{array}\right]=r(A)+r\left[\begin{array}{cc} 0 & E_{A}B\\ CF_{A} & D-CA^{†}B \end{array}\right].$$

*In particular, if* R(B)⊆R(A) *and* $R(C^{⁎})\subseteq R(A^{⁎})$*, then*

(4.8) $$r\left[\begin{array}{cc} A & B\\ C & D \end{array}\right]=r(A)+r(D-CA^{†}B).$$

*Furthermore, the following results hold.*

(a) $r[A,\;B]=r(A)\Leftrightarrow R(B)\subseteq R(A)\Leftrightarrow AA^{†}B=B\Leftrightarrow E_{A}B=0$*.*
(b) $r\left[\begin{array}{c} A\\ C \end{array}\right]=r(A)\Leftrightarrow R(C^{⁎})\subseteq R(A^{⁎})\Leftrightarrow CA^{†}A=C\Leftrightarrow CF_{A}=0$*.*
(c) $r[A,\;B]=r(A)+r(B)\Leftrightarrow R(A)\cap R(B)=\{0\}\Leftrightarrow R({\left(E_{A}B\right)}^{⁎})=R(B^{⁎})\Leftrightarrow R({\left(E_{B}A\right)}^{⁎})=R(A^{⁎})$*.*
(d) $r\left[\begin{array}{c} A\\ C \end{array}\right]=r(A)+r(C)\Leftrightarrow R(A^{⁎})\cap R(C^{⁎})=\{0\}\Leftrightarrow R(CF_{A})=R(C)\Leftrightarrow R(AF_{C})=R(A)$*.*
(e) $r\left[\begin{array}{cc} A & B\\ C & D \end{array}\right]=r(A)$ ⇔ R(B)⊆R(A)*,* $R(C^{⁎})\subseteq R(A^{⁎})$*, and* $CA^{†}B=D$*.*
(f) r(A+B)=r(A)+r(B)⇔R(A)∩R(B)={0} *and* $R(A^{⁎})\cap R(B^{⁎})=\{0\}$ *for* $A,\;B\in C^{m\times n}$*.*

Lemma 4.4 **[96]**

*Let* $A\in C^{m\times n}$ *and* $B\in C^{m\times k}$*, and denote* $P_{A}=AA^{†}$ *and* $P_{B}=BB^{†}$*. Then the following range equality*

(4.9) $$R(A)\cap R(B)=R(P_{A}P_{B})\cap R(P_{B}P_{A})$$

*holds. Consequently, the following rank equalities*

(4.10) r[A,B]=r(A)+r(B)−dim⁡(R(A)∩R(B)),

(4.11) $$r[A,\;B]=r(A)+r(B)-\dim\left(R(P_{A}P_{B})\cap R(P_{B}P_{A})\right),$$

(4.12) $$r[A,\;B]=r(A)+r(B)-r(P_{A}P_{B})-r(P_{B}P_{A})+r[P_{A}P_{B},\;P_{B}P_{A}]$$

*hold. In particular, the following results hold.*

(a) r[A,B]=r(A)+r(B) ⇔ $r[P_{A}P_{B},\;P_{B}P_{A}]=r(P_{A}P_{B})+r(P_{B}P_{A})$ ⇔ R(A)∩R(B)={0} ⇔ $R(P_{A}P_{B})\cap R(P_{B}P_{A})=\{0\}$*.*
(b) $r[A,\;B]=r(A)+r(B)-r(P_{A}P_{B})$ ⇔ $r[P_{A}P_{B},\;P_{B}P_{A}]=r(P_{A}P_{B})=r(P_{B}P_{A})$ ⇔ $R(P_{A}P_{B})=R(P_{B}P_{A})$ ⇔ $P_{A}P_{B}=P_{B}P_{A}$*.*
(c) $r[A,\;B]=r[P_{A}P_{B},\;P_{B}P_{A}]$ ⇔ $r(A^{⁎}B)=r(A)=r(B)$*.*

Lemma 4.5 **[78], [79]**

*Let* $A\in C^{m\times n},\;B\in C^{m\times k}$*,* $C\in C^{l\times n}$*, and* $D\in C^{l\times k}$*. Then*

(4.13) $$r(D-CA^{†}B)=r\left[\begin{array}{cc} A^{⁎}AA^{⁎} & A^{⁎}B\\ CA^{⁎} & D \end{array}\right]-r(A).$$

(4.14) $$r(D-CAA^{†}B)=r\left[\begin{array}{cc} A^{⁎}A & A^{⁎}B\\ CA & D \end{array}\right]-r(A),$$

(4.15) $$r(D-CA^{†}AB)=r\left[\begin{array}{cc} AA^{⁎} & AB\\ CA^{⁎} & D \end{array}\right]-r(A),$$

(4.16) $$r(A^{⁎}-A^{†})=r(AA^{⁎}A-A).$$

Lemma 4.6 **[57]**

*Let* $A\in C^{m\times n}$ *and* $B\in C^{n\times p}$*. Then*

(4.17) $$r(AB)=r(A)+r(B)-n+r((I_{n}-BB^{\left(1\right)})(I_{n}-A^{\left(1\right)}A))$$

*holds for all* $A^{\left(1\right)}$ *and* $B^{\left(1\right)}$*. In particular, the following inequalities*

(4.18) $$\max\{0,\;r(A)+r(B)-n\}\leq r(A)+r(B)-r[A^{⁎},\;B]\leq r(AB)\leq \min\{r(A),\;r(B)\}$$

*hold, and the following two groups of equivalent facts hold*

(4.19) $$r(AB)=r(A)+r(B)-n\Leftrightarrow (I_{n}-BB^{\left(1\right)})(I_{n}-A^{\left(1\right)}A)=0,$$

(4.20) r(AB)=n⇔r(A)=r(B)=n.

The two formulas in the following lemma are best known in elementary linear algebra.

Lemma 4.7 *Let* $A\in C^{m\times m}$*. Then*

(4.21) $$r(A-A^{2})=r(I_{m}-A)+r(A)-m,$$

(4.22) $$r(A-A^{3})=r(I_{m}+A)+r(I_{m}-A)+r(A)-2m.$$

Lemma 4.8 **[80]**

*Let* $A\in C^{m\times n}$ *and assume that* $X_{1},\;X_{2}\in \{A^{\left(2\right)}\}$*. Then*

(4.23) $$r(X_{1}-X_{2})=r\left[\begin{array}{c} X_{1}\\ X_{2} \end{array}\right]+r[X_{1},\;X_{2}]-r(X_{1})-r(X_{2}).$$

Lemma 4.9 **[96], [97], [107]**

*Let* $P,\;Q\in C^{m\times m}$ *be a pair of orthogonal projectors. Then*

(4.24) r(P+Q)=r[P,Q],

(4.25) r(P−Q)=2r[P,Q]−r(P)−r(Q),

(4.26) $$r(PQ-QP)=r(P-Q)+r(P+Q-I_{m})-m,$$

(4.27) r(PQ−QP)=2r[P,Q]+2r(PQ)−2r(P)−2r(Q),

(4.28) r(PQ−QP)=2r[PQ,QP]−2r(PQ).

Lemma 4.10 **[107]**

*Let* $A\in C^{m\times n}$ *be given, and let* $P\in C^{m\times m}$ *and* $Q\in C^{n\times n}$ *be a pair of idempotent matrices.*

(4.29) $$r(PA-AQ)=r\left[\begin{array}{c} PA\\ Q \end{array}\right]+r[AQ,\;P]-r(P)-r(Q).$$

Lemma 4.11 **[82], [85]**

*Let* $A\in C^{m\times n}$*,* $B\in C^{m\times k}$*,* $C\in C^{l\times n}$*, and* $D\in C^{l\times k}$ *be given. Then*

(4.30) $$\max_{A^{\left(1\right)}\in \{A^{\left(1\right)}\}}r(D-CA^{\left(1\right)}B)=\min\left\{r[C,\;D],\;\;r\left[\begin{array}{c} B\\ D \end{array}\right],\;\;r\left[\begin{array}{cc} A & B\\ C & D \end{array}\right]-r(A)\right\}.$$

*Hence*

(4.31) $$CA^{\left(1\right)}B=D\;\text{for all }A^{\left(1\right)}\Leftrightarrow [C,\;D]=0\;\text{or}\;\left[\begin{array}{c} B\\ D \end{array}\right]=0\;\text{or}\left[\begin{array}{cc} A & B\\ C & D \end{array}\right]=r(A).$$

Lemma 4.12 *Let* $A\in C^{m\times n}$ *and* $B\in C^{n\times p}$*, and denote*

$$t_{1}=\min\{r(A)+r(B),\;\;n\},\;\;t_{2}=r[A^{⁎},\;B],\;\;t_{3}=r[A^{⁎}AB,\;B],\;\;t_{4}=r[AA^{⁎}AB,\;AB],t_{5}=r(A)+r[A^{⁎}AB,\;B]-r[A^{⁎},\;B],\;\;t_{6}=r(AB),\;\;t_{7}=r(A)+r(B)-r[A^{⁎},\;B],\;\;t_{8}=\max\{0,\;\;r(A)+r(B)-n\}.$$

*Then the following inequalities hold*

(4.32) $$t_{1}\geq t_{2}\geq t_{3}\geq t_{4}\geq t_{5}\geq t_{6}\geq t_{7}\geq t_{8}.$$

Proof The first inequality in (4.32) follows from the two well-known inequalities $r[A^{⁎},\;B]\leq r(A)+r(B)$ and $r[A^{⁎},\;B]\leq n$. The second inequality in (4.32) follows directly the matrix product $[A^{⁎}AB,\;B]=[A^{⁎},\;B]\left[\begin{array}{cc} AB & 0\\ 0 & I_{p} \end{array}\right]$. The third inequality in (4.32) follows directly the matrix product $\left[AA^{⁎}AB,\;AB]=A[A^{⁎}AB,\;B\right]$. Furthermore, rewrite $\left[AA^{⁎}AB,\;AB\right]$ as a triple matrix product $[AA^{⁎}AB,\;AB]=A[A^{⁎}A,\;B]\left[\begin{array}{cc} B & 0\\ 0 & I_{p} \end{array}\right]$, and applying the well-known Frobenius' inequality r(XYZ)≥r(XY)+r(YZ)−r(Y) to this triple product yields

$$r[AA^{⁎}AB,\;AB]\geq r(A[A^{⁎}A,\;B])+r\left([A^{⁎}A,\;B]\left[\begin{array}{cc} B & 0\\ 0 & I_{p} \end{array}\right]\right)-r[A^{⁎}A,\;B]=r(A)+r[A^{⁎}AB,\;B]-r[A^{⁎},\;B],$$

establishing the fourth inequality in (4.32). Applying the Frobenius' inequality to the product $[A^{⁎}AB,\;B]=[A^{⁎},\;I_{n}]\left[\begin{array}{cc} A & 0\\ 0 & B \end{array}\right]\left[\begin{array}{cc} B & 0\\ 0 & I_{p} \end{array}\right]$ yields

$$r[A^{⁎}AB,\;B]\geq r\left([A^{⁎},\;I_{n}]\left[\begin{array}{cc} A & 0\\ 0 & B \end{array}\right]\right)+r\left(\left[\begin{array}{cc} A & 0\\ 0 & B \end{array}\right]\left[\begin{array}{cc} B & 0\\ 0 & I_{p} \end{array}\right]\right)-r\left[\begin{array}{cc} A & 0\\ 0 & B \end{array}\right]=r[A^{⁎},\;B]+r(AB)-r(A),$$

establishing the fifth inequality in (4.32). Applying (4.4) and inequality r(X−Y)≥r(X)−r(Y) to $\left[A^{⁎},\;B\right]$ yields

$$r[A^{⁎},\;B]=r(A^{⁎})+r(B-A^{†}AB)\geq r(A)+r(B)-r(A^{†}AB)=r(A)+r(B)-r(AB),$$

thus establishing the sixth inequality in (4.32). The last inequality in (4.32) is equivalent to the first inequality. □

Lemma 4.13 *Let* $A\in C^{m\times n},\;B\in C^{n\times p}$*, and denote*

$$J=\left[\begin{array}{cc} AB{\left(AB\right)}^{⁎}AB & \;ABB^{⁎}B\\ AA^{⁎}AB & AB \end{array}\right]=\left[\begin{array}{c} ABB^{⁎}\\ A \end{array}\right][A^{⁎}AB,\;B],s_{1}=r\left[\begin{array}{c} ABB^{⁎}\\ A \end{array}\right]+r[A^{⁎}AB,\;B]-r[A^{⁎},\;B],s_{2}=r[A^{⁎},\;B]+2r(AB)-r(A)-r(B),s_{3}=r\left[\begin{array}{c} ABB^{⁎}B\\ AB \end{array}\right]+r[AA^{⁎}AB,\;AB]-r(AB),s_{4}=r\left[\begin{array}{c} ABB^{⁎}\\ A \end{array}\right]+r[A^{⁎}AB,\;B]+r(A)+r(B)-2r[A^{⁎},\;B]-r(AB).$$

*Then*

(4.33) $$r(J)\geq s_{1}\geq s_{2}\geq r(AB),$$

(4.34) $$r(J)\geq s_{3}\geq s_{4}\geq r(AB).$$

Proof Follows from Lemma 4.12. □

Lemma 4.14 **[95]**

*Let* M *and* N *be a pair of linear subspaces of* $C^{m}$*, and let* $P_{M}$ *and* $P_{N}$ *be the orthogonal projectors onto* M *and* N*, respectively. Then the following two dimension formulas*

$$\dim[(M+N)\cap (M+N^{⊥})\cap (M^{⊥}+N)\cap (M^{⊥}+N^{⊥})]=2r(P_{M}P_{N})-2\dim(M\cap N),\dim[(M\cap N)⊕(M\cap N^{⊥})⊕(M^{⊥}\cap N)⊕(M^{⊥}\cap N^{⊥})]=m-2r(P_{M}P_{N})+2\dim(M\cap N)$$

*hold. In particular, the following statements are equivalent:*

(a) $\left(M+N)\cap (M+N^{⊥})\cap (M^{⊥}+N)\cap (M^{⊥}+N^{⊥})=\{0\right\}$*.*
(b) $(M\cap N)⊕(M\cap N^{⊥})⊕(M^{⊥}\cap N)⊕(M^{⊥}\cap N^{⊥})=C^{m}$*.*
(c) $r(P_{M}P_{N})=\dim(M\cap N)$ *and/or* $r(P_{N}P_{M})=\dim(N\cap M)$*.*
(d) $P_{M}P_{N}=P_{M\cap N}$ *and/or* $P_{N}P_{M}=P_{N\cap M}$*.*
(e) $P_{M}P_{N}=P_{N}P_{M}$*.*

## 5 Characterizations of linear and multilinear matrix identities

Matrix equations occupy a central place in the development of matrix theory. Because generalized inverses of a matrix are defined to be solutions of some/all of the four Penrose equations, (1.8) can be regarded as a nonlinear matrix equation of the form X=YZ subject to the restrictions $X\in \{{\left(AB\right)}^{\left(i,\ldots ,j\right)}\}$, $Y\in \{B^{\left(s_{2},\ldots ,t_{2}\right)}\}$, and $Z\in \{A^{\left(s_{1},\ldots ,t_{1}\right)}\}$. For a general matrix equation $f(X_{1},\ldots ,X_{k})=0$, it is a fundamental and challenging problem to establish necessary and sufficient conditions for the equality to hold for all the variable matrices $X_{1},\ldots ,X_{k}$ due to the noncommutativity of matrix algebra. For the two simplest matrix equations AX=0 and AXB=0, it is old news that AX=0 holds for all *X* if and only if A=0; AXB=0 holds for all *X* if and only if A=0 or B=0 (cf. [6]). As matrix equations are given in general forms that involve two or more unknown matrices, the derivations and representations of identifying conditions become increasingly difficult for the matrix equations to always hold for all variable matrices in them. In a recent paper [45], Jiang and Tian reconsidered the uniqueness (invariance property) of some general linear and multilinear matrix identities and provided a readily comprehensible procedure of establishing various types of linear and multilinear matrix identities that involve separated variable matrices by means of the block matrix method. In this section, we present a group of known results concerning linear and multilinear matrix identities, which we shall use in the characterization of the invariance properties of products $B^{\left(s_{2},\ldots ,t_{2}\right)}A^{\left(s_{1},\ldots ,t_{1}\right)}$ with respect to the choices of $A^{\left(s_{1},\ldots ,t_{1}\right)}$ and $B^{\left(s_{2},\ldots ,t_{2}\right)}$.

Lemma 5.1 **[62]**

*Let*

(5.1) BX=A

*be a given linear matrix equation, where* $A\in C^{m\times n}$ *and* $B\in C^{m\times p}$ *are known matrices, and* $X\in C^{p\times n}$ *is an unknown matrix. Then*

(5.2) $$\mathrm{(5.1)}\text{ is solvable for }X\Leftrightarrow R(A)\subseteq R(B)\Leftrightarrow BB^{†}A=A\Leftrightarrow E_{B}A=0.$$

*In this situation, the general solution of* (5.1) *can be written in the following parametric form*

(5.3) $$X=B^{†}A+F_{B}V,$$

*where* $V\in C^{p\times n}$ *is arbitrary. In particular,* (5.1) *holds for all* $X\in C^{p\times n}$ *if and only if* A=0 *and* B=0*, or equivalently,* [A,B]=0*.*

Lemma 5.2 **[62]**

*Let*

(5.4) BXC=A

*be a given linear matrix equation, where* $A\in C^{m\times n}$*,* $B\in C^{m\times p}$*, and* $C\in C^{q\times n}$ *are known matrices. Then the following statements are equivalent:*

(a) *Eq.* (5.4) *is solvable for* $X\in C^{p\times q}$*.*
(b) R(A)⊆R(B) *and* $R(A^{⁎})\subseteq R(C^{⁎})$*.*
(c) r[A,B]=r(B) *and* $r\left[\begin{array}{c} A\\ C \end{array}\right]=r(C)$*.*
(d) $BB^{†}A=A$ *and* $AC^{†}C=A$*.*
(e) $BB^{†}AC^{†}C=A$*.*

*In this situation, the general solution of* (5.4) *can be written in the following parametric form*

(5.5) $$X=B^{†}AC^{†}+F_{B}V+WE_{C},$$

*where* $V,\;W\in C^{p\times q}$ *are arbitrary. In particular,* (5.4) *holds for all* $X\in C^{p\times q}$ *if and only if*

(5.6) $$[A,\;B]=0\;\;\text{or}\;\;\left[\begin{array}{c} A\\ C \end{array}\right]=0.$$

The constructions of the two block matrices in (5.6) are helpful to describe more general linear and multilinear matrix equalities, which will be demonstrated in the following several lemmas.

Lemma 5.3 **[45]**

*Let*

(5.7) $$B_{1}X_{1}C_{1}+B_{2}X_{2}C_{2}=A$$

*be a given linear matrix equation, where* $A\in C^{m\times n}$*,* $B_{1}\in C^{m\times p_{1}}$*,* $B_{2}\in C^{m\times p_{2}}$*,* $C_{1}\in C^{q_{1}\times n}$*, and* $C_{2}\in C^{q_{2}\times n}$ *are known matrices. Then the following results hold.*

(a) *Eq.* (5.7) *holds for all* $X_{1}\in C^{p_{1}\times q_{1}}$ *and* $X_{2}\in C^{p_{2}\times q_{2}}$ *if and only if the 5 given matrices satisfy one of the following 4 block matrix equalities:*
  $$\left(i\right)\;\;[A,\;B_{1},\;B_{2}]=0,\;\;\;\left(\mathrm{ii}\right)\;\;\left[\begin{array}{cc} A & B_{1}\\ C_{2} & 0 \end{array}\right]=0,\;\;\;\left(\mathrm{iii}\right)\;\;\left[\begin{array}{cc} A\; & B_{2}\\ C_{1} & 0 \end{array}\right]=0,\;\;\;\left(\mathrm{iv}\right)\;\left[\begin{array}{c} A\\ C_{1}\\ C_{2} \end{array}\right]=0.$$
(b) *Under the assumptions that* $R(B_{1})\subseteq R(B_{2})$ *and* $R(C_{1}^{⁎})⊇R(C_{2}^{⁎})$*,* (5.7) *holds for all matrices* $X_{1}$ *and* $X_{2}$ *if and only if one of the following 3 block matrix equalities holds*
  $$\left(i\right)\;[A,\;B_{2}]=0,\;\;\;\left(\mathrm{ii}\right)\;\left[\begin{array}{c} A\\ C_{1} \end{array}\right]=0,\;\;\;\left(\mathrm{iii}\right)\;\left[\begin{array}{cc} A & B_{1}\\ C_{2} & 0 \end{array}\right]=0.$$

Several multilinear versions of matrix identities were also established in [45] as follows.

Lemma 5.4 *Let*

(5.8) $$(A_{1}+B_{1}X_{1}C_{1})(A_{2}+B_{2}X_{2}C_{2})=A$$

*be a given multilinear matrix equation, where* $A\in C^{m\times n}$*,* $A_{1}\in C^{m\times s}$*,* $B_{1}\in C^{m\times p_{1}}$*,* $C_{1}\in C^{q_{1}\times s}$*,* $A_{2}\in C^{s\times n}$*,* $B_{2}\in C^{s\times p_{2}}$*, and* $C_{2}\in C^{q_{2}\times n}$ *are known matrices. Then the following results hold.*

(a) *Eq.* (5.8) *holds for all* $X_{1}\in C^{p_{1}\times q_{1}}$ *and* $X_{2}\in C^{p_{2}\times q_{2}}$ *if and only if one of the following 4 block matrix equalities holds:*
  $$\left(i\right)\;[A_{1}A_{2}-A,\;A_{1}B_{2},\;B_{1}]=0,\;\;\left(\mathrm{ii}\right)\;\left[\begin{array}{cc} A_{1}A_{2}-A & B_{1}\\ C_{2} & 0 \end{array}\right]=0,\;\;\left(\mathrm{iii}\right)\;\left[\begin{array}{cc} A_{1}A_{2}-A & A_{1}B_{2}\\ C_{1}A_{2} & C_{1}B_{2} \end{array}\right]=0,\;\;\left(\mathrm{iv}\right)\;\left[\begin{array}{c} A_{1}A_{2}-A\\ C_{1}A_{2}\\ C_{2} \end{array}\right]=0.$$
(b) *Under the assumption* $R(A_{1})\subseteq R(B_{1})$ *and* $R(A_{2})\subseteq R(B_{2})$*,* (5.8) *holds for all* $X_{1}\in C^{p_{1}\times q_{1}}$ *and* $X_{2}\in C^{p_{2}\times q_{2}}$ *if and only if one of the following 3 block matrix equalities holds*
  $$\left(i\right)\;\;[A,\;\;B_{1}]=0,\;\;\;\;\left(\mathrm{ii}\right)\;\;\left[\begin{array}{cc} A & A_{1}B_{2}\\ 0 & C_{1}B_{2} \end{array}\right]=0,\;\;\;\;\left(\mathrm{iii}\right)\;\;\left[\begin{array}{c} A_{1}A_{2}-A\\ C_{1}A_{2}\\ C_{2} \end{array}\right]=0.$$
(c) *Under the assumption* $R(A_{1}^{⁎})\subseteq R(C_{1}^{⁎})$ *and* $R(A_{2}^{⁎})\subseteq R(C_{2}^{⁎})$*,* (5.8) *holds for all* $X_{1}\in C^{p_{1}\times q_{1}}$ *and* $X_{2}\in C^{p_{2}\times q_{2}}$ *if and only if one of the following 3 block matrix equalities holds*
  $$\left(i\right)\;[A_{1}A_{2}-A,\;A_{1}B_{2},\;B_{1}]=0,\;\;\;\;\left(\mathrm{ii}\right)\;\left[\begin{array}{cc} A & 0\\ C_{1}A_{2} & C_{1}B_{2} \end{array}\right]=0,\;\;\;\;\left(\mathrm{iii}\right)\;\left[\begin{array}{c} A\\ C_{2} \end{array}\right]=0.$$
(d) *Under the assumption* $R(A_{1})\subseteq R(B_{1})$ *and* $R(A_{2}^{⁎})\subseteq R(C_{2}^{⁎})$*,* (5.8) *holds for all* $X_{1}\in C^{p_{1}\times q_{1}}$ *and* $X_{2}\in C^{p_{2}\times q_{2}}$ *if and only if one of the following 3 block matrix equalities holds*
  $$\left(i\right)\;\;[A,\;B_{1}]=0,\;\;\;\;\left(\mathrm{ii}\right)\;\;\left[\begin{array}{c} A\\ C_{2} \end{array}\right]=0,\;\;\;\;\left(\mathrm{iii}\right)\;\;\left[\begin{array}{cc} A_{1}A_{2}-A & A_{1}B_{2}\\ C_{1}A_{2} & C_{1}B_{2} \end{array}\right]=0.$$
(e) *Under the assumption* $R(A_{1}^{⁎})\subseteq R(C_{1}^{⁎})$ *and* $R(A_{2})\subseteq R(B_{2})$*,* (5.8) *holds for all* $X_{1}\in C^{p_{1}\times q_{1}}$ *and* $X_{2}\in C^{p_{2}\times q_{2}}$ *if and only if one of the following 4 block matrix equalities holds*
  $$\left(i\right)\;[A,\;A_{1}B_{2},\;B_{1}]=0,\;\;\left(\mathrm{ii}\right)\;\left[\begin{array}{cc} A_{1}A_{2}-A & B_{1}\\ C_{2} & 0 \end{array}\right]=0,\;\;\left(\mathrm{iii}\right)\;\left[\begin{array}{cc} A & 0\\ 0 & C_{1}B_{2} \end{array}\right]=0,\;\;\left(\mathrm{iv}\right)\;\left[\begin{array}{c} A\\ C_{1}A_{2}\\ C_{2} \end{array}\right]=0.$$

Lemma 5.5 *Let*

(5.9) $$(A_{1}+B_{1}X_{1}C_{1})(A_{2}+B_{2}X_{2}C_{2})(A_{3}+B_{3}X_{3}C_{3})=A$$

*be a given multilinear matrix equation, where A,* $A_{i}$*,* $B_{i}$*, and* $C_{i}$ *are known matrices of appropriate sizes,* i=1,2,3*. Then the following results hold.*

(a) *Eq.* (5.9) *holds for all* $X_{1}$*,* $X_{2}$*, and* $X_{3}$ *if and only if one of the following 8 block matrix equalities holds*
  $$\begin{array}{cccc} \left(i\right)\; & [A_{1}A_{2}A_{3}-A,\;A_{1}A_{2}B_{3},\;A_{1}B_{2},\;B_{1}]=0,\; & \left(\mathrm{ii}\right)\; & \left[\begin{array}{ccc} A_{1}A_{2}A_{3}-A & A_{1}A_{2}B_{3} & A_{1}B_{2}\\ C_{1}A_{2}A_{3} & C_{1}A_{2}B_{3} & C_{1}B_{2} \end{array}\right]=0,\\ \left(\mathrm{iii}\right)\; & \left[\begin{array}{ccc} A_{1}A_{2}A_{3}-A & A_{1}A_{2}B_{3} & B_{1}\\ C_{2}A_{3} & C_{2}B_{3} & 0 \end{array}\right]=0,\; & \left(\mathrm{iv}\right)\; & \left[\begin{array}{ccc} A_{1}A_{2}A_{3}-A & A_{1}B_{2} & B_{1}\\ C_{3} & 0 & 0 \end{array}\right]=0,\\ \left(v\right)\; & \left[\begin{array}{cc} A_{1}A_{2}A_{3}-A & A_{1}A_{2}B_{3}\\ C_{1}A_{2}A_{3} & C_{1}A_{2}B_{3}\\ C_{2}A_{3} & C_{2}B_{3} \end{array}\right]=0,\; & \left(\mathrm{vi}\right)\; & \left[\begin{array}{cc} A_{1}A_{2}A_{3}-A & A_{1}B_{2}\\ C_{1}A_{2}A_{3} & C_{1}B_{2}\\ C_{3} & 0 \end{array}\right]=0,\\ \left(\mathrm{vii}\right)\; & \left[\begin{array}{cc} A_{1}A_{2}A_{3}-A & B_{1}\\ C_{2}A_{3} & 0\\ C_{3} & 0 \end{array}\right]=0,\; & \left(\mathrm{viii}\right)\; & \left[\begin{array}{c} A_{1}A_{2}A_{3}-A\\ C_{1}A_{2}A_{3}\\ C_{2}A_{3}\\ C_{3} \end{array}\right]=0. \end{array}$$
(b) *Under the assumption* $R(A_{1})\subseteq R(B_{1})$ *and* $R(A_{3})\subseteq R(B_{3})$*,* (5.9) *holds for all* $X_{1}$*,* $X_{2}$*, and* $X_{3}$ *if and only if one of the following 5 block matrix equalities holds*
  $$\begin{array}{cc} \left(i\right)\; & [A,\;B_{1}]=0,\;\;\left(\mathrm{ii}\right)\;\;\left[\begin{array}{ccc} A & A_{1}A_{2}B_{3} & A_{1}B_{2}\\ 0 & C_{1}A_{2}B_{3} & C_{1}B_{2} \end{array}\right]=0,\;\;\left(\mathrm{iii}\right)\;\left[\begin{array}{cc} A & A_{1}A_{2}B_{3}\\ 0 & C_{1}A_{2}B_{3}\\ 0 & C_{2}B_{3} \end{array}\right]=0,\\ \left(\mathrm{iv}\right)\; & \left[\begin{array}{cc} A_{1}A_{2}A_{3}-A & A_{1}B_{2}\\ C_{1}A_{2}A_{3} & C_{1}B_{2}\\ C_{3} & 0 \end{array}\right]=0,\;\;\;\left(v\right)\;\left[\begin{array}{c} A_{1}A_{2}A_{3}-A\\ C_{1}A_{2}A_{3}\\ C_{2}A_{3}\\ C_{3} \end{array}\right]=0. \end{array}$$
(c) *Under the assumption* $R(A_{1}^{⁎})\subseteq R(C_{1}^{⁎})$ *and* $R(A_{3}^{⁎})\subseteq R(C_{3}^{⁎})$*,* (5.9) *holds for all* $X_{1}$*,* $X_{2}$*, and* $X_{3}$ *if and only if one of the following 5 block matrix equalities holds*
  $$\begin{array}{cccc} \left(i\right)\; & [A_{1}A_{2}A_{3}-A,\;A_{1}A_{2}B_{3},\;A_{1}B_{2},\;B_{1}]=0,\; & \left(\mathrm{ii}\right)\; & \left[\begin{array}{ccc} A & 0 & 0\\ C_{1}A_{2}A_{3} & C_{1}A_{2}B_{3} & C_{1}B_{2} \end{array}\right]=0,\\ \left(\mathrm{iii}\right)\; & \left[\begin{array}{ccc} A_{1}A_{2}A_{3}-A & A_{1}A_{2}B_{3} & B_{1}\\ C_{2}A_{3} & C_{2}B_{3} & 0 \end{array}\right]=0,\; & \left(\mathrm{iv}\right)\; & \left[\begin{array}{cc} A & 0\\ C_{1}A_{2}A_{3} & C_{1}A_{2}B_{3}\\ C_{2}A_{3} & C_{2}B_{3} \end{array}\right]=0,\;\;\;\;\left(v\right)\;\left[\begin{array}{c} A\\ C_{3} \end{array}\right]=0. \end{array}$$
(d) *Under the assumption* $R(A_{1})\subseteq R(B_{1})$ *and* $R(A_{3}^{⁎})\subseteq R(C_{3}^{⁎})$*,* (5.9) *holds for all* $X_{1}$*,* $X_{2}$*, and* $X_{3}$ *if and only if one of the following 4 block matrix equalities holds*
  $$\left(i\right)\;[A,\;B_{1}]=0,\;\;\left(\mathrm{ii}\right)\;\left[\begin{array}{c} A\\ C_{3} \end{array}\right]=0,\;\;\left(\mathrm{iii}\right)\;\left[\begin{array}{ccc} A_{1}A_{2}A_{3}-A & A_{1}A_{2}B_{3} & A_{1}B_{2}\\ C_{1}A_{2}A_{3} & C_{1}A_{2}B_{3} & C_{1}B_{2} \end{array}\right]=0,\;\;\left(\mathrm{iv}\right)\;\left[\begin{array}{cc} A_{1}A_{2}A_{3}-A & A_{1}A_{2}B_{3}\\ C_{1}A_{2}A_{3} & C_{1}A_{2}B_{3}\\ C_{2}A_{3} & C_{2}B_{3} \end{array}\right]=0.$$
(e) *Under the assumption* $R(A_{1}^{⁎})\subseteq R(C_{1}^{⁎})$ *and* $R(A_{3})\subseteq R(B_{3})$*,* (5.9) *holds for all matrices* $X_{1}$*,* $X_{2}$*, and* $X_{3}$ *if and only if one of the following 8 block matrix equalities holds*
  $$\begin{array}{cccc} \left(i\right)\; & [A,\;A_{1}A_{2}B_{3},\;A_{1}B_{2},\;B_{1}]=0,\; & \left(\mathrm{ii}\right)\; & \left[\begin{array}{ccc} A & 0 & 0\\ 0 & C_{1}A_{2}B_{3} & C_{1}B_{2} \end{array}\right]=0,\\ \left(\mathrm{iii}\right)\; & \left[\begin{array}{ccc} A & A_{1}A_{2}B_{3} & B_{1}\\ 0 & C_{2}B_{3} & 0 \end{array}\right]=0,\; & \left(\mathrm{iv}\right)\; & \left[\begin{array}{ccc} A_{1}A_{2}A_{3}-A & A_{1}B_{2} & B_{1}\\ C_{3} & 0 & 0 \end{array}\right]=0,\\ \left(v\right)\; & \left[\begin{array}{cc} A & 0\\ 0 & C_{1}A_{2}B_{3}\\ 0 & C_{2}B_{3} \end{array}\right]=0,\; & \left(\mathrm{vi}\right)\; & \left[\begin{array}{cc} A & 0\\ C_{1}A_{2}A_{3} & C_{1}B_{2}\\ C_{3} & 0 \end{array}\right]=0,\\ \left(\mathrm{vii}\right)\; & \left[\begin{array}{cc} A_{1}A_{2}A_{3}-A & B_{1}\\ C_{2}A_{3} & 0\\ C_{3} & 0 \end{array}\right]=0,\; & \left(\mathrm{viii}\right)\; & \left[\begin{array}{c} A\\ C_{1}A_{2}A_{3}\\ C_{2}A_{3}\\ C_{3} \end{array}\right]=0. \end{array}$$

Lemma 5.6 *Let*

(5.10) $$(A_{1}+B_{1}X_{1}C_{1})(A_{2}+B_{2}X_{2}+Y_{2}C_{2})(A_{3}+B_{3}X_{3}C_{3})=A$$

*be a given multilinear matrix equation, where A,* $A_{i}$*,* $B_{i}$*, and* $C_{i}$ *are known matrices of appropriate sizes,* i=1,2,3*. Then* (5.10) *holds for all matrices* $X_{1}$*,* $X_{2}$*,* $X_{3}$*, and* $Y_{2}$ *if and only if one of the following 8 block matrix equalities holds*

$$\begin{array}{cc} \left(i\right)\; & [A,\;A_{1},\;B_{1}]=0,\;\;\;\left(\mathrm{ii}\right)\;\;\left[\begin{array}{cc} A & A_{1}\\ 0 & C_{1} \end{array}\right]=0,\;\;\;\left(\mathrm{iii}\right)\;\;\left[\begin{array}{cc} A & 0\\ A_{3} & B_{3} \end{array}\right]=0,\;\;\;\left(\mathrm{iv}\right)\;\;\left[\begin{array}{c} A\\ A_{3}\\ C_{3} \end{array}\right]=0,\\ \left(v\right)\; & \left[\begin{array}{cccc} A_{1}A_{2}A_{3}-A & A_{1}A_{2}B_{3} & A_{1}B_{2} & B_{1}\\ C_{2}A_{3} & C_{2}B_{3} & 0 & 0 \end{array}\right]=0,\;\;\;\left(\mathrm{vi}\right)\;\left[\begin{array}{ccc} A_{1}A_{2}A_{3}-A & A_{1}B_{2} & B_{1}\\ C_{2}A_{3} & 0 & 0\\ C_{3} & 0 & 0 \end{array}\right]=0,\\ \left(\mathrm{vii}\right)\; & \left[\begin{array}{ccc} A_{1}A_{2}A_{3}-A & A_{1}A_{2}B_{3} & A_{1}B_{2}\\ C_{1}A_{2}A_{3} & C_{1}A_{2}B_{3} & C_{1}B_{2}\\ C_{2}A_{3} & C_{2}B_{3} & 0 \end{array}\right]=0,\;\;\;\;\;\;\;\;\left(\mathrm{viii}\right)\;\left[\begin{array}{cc} A_{1}A_{2}A_{3}-A & A_{1}B_{2}\\ C_{1}A_{2}A_{3} & C_{1}B_{2}\\ C_{2}A_{3} & 0\\ C_{3} & 0 \end{array}\right]=0. \end{array}$$

*Under the assumption* $R(A_{1})\subseteq R(B_{1})$ *and* $R(A_{3}^{⁎})\subseteq R(C_{3}^{⁎})$*,* (5.10) *holds for all matrices* $X_{1}$*,* $X_{2}$*,* $X_{3}$*, and* $Y_{2}$ *if and only if one of the following 5 block matrix equalities holds*

$$\left(i\right)\;[A,\;B_{1}]=0,\;\;\left(\mathrm{ii}\right)\;\left[\begin{array}{c} A\\ C_{3} \end{array}\right]=0,\;\;\left(\mathrm{iii}\right)\;\left[\begin{array}{cc} A & A_{1}\\ 0 & C_{1} \end{array}\right]=0,\;\;\left(\mathrm{iv}\right)\;\left[\begin{array}{cc} A & 0\\ A_{3} & B_{3} \end{array}\right]=0,\;\;\left(v\right)\;\left[\begin{array}{ccc} A_{1}A_{2}A_{3}-A & A_{1}A_{2}B_{3} & A_{1}B_{2}\\ C_{1}A_{2}A_{3} & C_{1}A_{2}B_{3} & C_{1}B_{2}\\ C_{2}A_{3} & C_{2}B_{3} & 0 \end{array}\right]=0.$$

Lemma 5.7 *Let*

(5.11) $$(A_{1}+B_{1}X_{1}C_{1}+D_{1}Y_{1}E_{1})(A_{2}+B_{2}X_{2}C_{2}+D_{2}Y_{2}E_{2})=A$$

*be a given multilinear matrix equation, where A,* $A_{i}$*,* $B_{i}$*,* $C_{i}$*,* $D_{i}$*, and* $E_{i}$ *are known matrices of appropriate sizes,* i=1,2*. Then the following results hold.*

(a) *Eq.* (5.11) *holds for all matrices* $X_{1}$*,* $X_{2}$*,* $Y_{1}$*, and* $Y_{2}$ *if and only if one of the following 16 block matrix equalities holds*
  $$\begin{array}{cccc} \left(i\right)\; & [A_{1}A_{2}-A,\;A_{1}B_{2},\;A_{1}D_{2},\;B_{1},\;D_{1}]=0,\;\;\; & \left(\mathrm{ii}\right)\; & \left[\begin{array}{cccc} A_{1}A_{2}-A & A_{1}B_{2} & A_{1}D_{2} & B_{1}\\ E_{1}A_{2} & E_{1}B_{2} & E_{1}D_{2} & 0 \end{array}\right]=0,\\ \left(\mathrm{iii}\right)\; & \left[\begin{array}{cccc} A_{1}A_{2}-A & A_{1}B_{2} & A_{1}D_{2} & D_{1}\\ C_{1}A_{2} & C_{1}B_{2} & C_{1}D_{2} & 0 \end{array}\right]=0,\; & \left(\mathrm{iv}\right)\; & \left[\begin{array}{cccc} A_{1}A_{2}-A & A_{1}B_{2} & B_{1} & D_{1}\\ E_{2} & 0 & 0 & 0 \end{array}\right]=0,\\ \left(v\right)\; & \left[\begin{array}{cccc} A_{1}A_{2}-A & A_{1}D_{2} & B_{1} & D_{1}\\ C_{2} & 0 & 0 & 0 \end{array}\right]=0,\; & \left(\mathrm{vi}\right)\; & \left[\begin{array}{ccc} A_{1}A_{2}-A & A_{1}B_{2} & A_{1}D_{2}\\ C_{1}A_{2} & C_{1}B_{2} & C_{1}D_{2}\\ E_{1}A_{2} & E_{1}B_{2} & E_{1}D_{2} \end{array}\right]=0,\\ \left(\mathrm{vii}\right)\; & \left[\begin{array}{ccc} A_{1}A_{2}-A & A_{1}B_{2} & B_{1}\\ E_{1}A_{2} & E_{1}B_{2} & 0\\ E_{2} & 0 & 0 \end{array}\right]=0,\; & \left(\mathrm{viii}\right)\; & \left[\begin{array}{ccc} A_{1}A_{2}-A & A_{1}B_{2} & D_{1}\\ C_{1}A_{2} & C_{1}B_{2} & 0\\ E_{2} & 0 & 0 \end{array}\right]=0,\\ \left(\mathrm{ix}\right)\; & \left[\begin{array}{ccc} A_{1}A_{2}-A & A_{1}D_{2} & B_{1}\\ E_{1}A_{2} & E_{1}D_{2} & 0\\ C_{2} & 0 & 0 \end{array}\right]=0,\; & \left(x\right)\; & \left[\begin{array}{ccc} A_{1}A_{2}-A & A_{1}D_{2} & D_{1}\\ C_{1}A_{2} & C_{1}D_{2} & 0\\ C_{2} & 0 & 0 \end{array}\right]=0,\\ \left(\mathrm{xi}\right)\; & \left[\begin{array}{ccc} A_{1}A_{2}-A & B_{1} & D_{1}\\ C_{2} & 0 & 0\\ E_{2} & 0 & 0 \end{array}\right]=0,\; & \left(\mathrm{xii}\right)\; & \left[\begin{array}{cc} A_{1}A_{2}-A & A_{1}B_{2}\\ C_{1}A_{2} & C_{1}B_{2}\\ E_{1}A_{2} & E_{1}B_{2}\\ E_{2} & 0 \end{array}\right]=0,\\ \left(\mathrm{xiii}\right)\; & \left[\begin{array}{cc} A_{1}A_{2}-A & A_{1}D_{2}\\ C_{1}A_{2} & C_{1}D_{2}\\ E_{1}A_{2} & E_{1}D_{2}\\ C_{2} & 0 \end{array}\right]=0,\; & \left(\mathrm{xiv}\right)\; & \left[\begin{array}{cc} A_{1}A_{2}-A & B_{1}\\ E_{1}A_{2} & 0\\ C_{2} & 0\\ E_{2} & 0 \end{array}\right]=0,\\ \left(\mathrm{xv}\right)\; & \left[\begin{array}{cc} A_{1}A_{2}-A & D_{1}\\ C_{1}A_{2} & 0\\ C_{2} & 0\\ E_{2} & 0 \end{array}\right]=0,\; & \left(\mathrm{xvi}\right)\; & \left[\begin{array}{c} A_{1}A_{2}-A\\ C_{1}A_{2}\\ E_{1}A_{2}\\ C_{2}\\ E_{2} \end{array}\right]=0. \end{array}$$
(b) *The matrix equation*
  (5.12) $$(A_{1}+B_{1}X_{1}+Y_{1}E_{1})(A_{2}+B_{2}X_{2}+Y_{2}E_{2})=A$$
  *holds for all matrices* $X_{1}$*,* $X_{2}$*,* $Y_{1}$*, and* $Y_{2}$ *if and only if one of the following 3 block matrix equalities holds*
  $$\left(i\right)\;\;\left[\begin{array}{ccc} A & A_{1} & B_{1}\\ 0 & E_{1} & 0 \end{array}\right]=0,\;\;\;\left(\mathrm{ii}\right)\;\;\left[\begin{array}{cc} A & 0\\ A_{2} & B_{2}\\ E_{2} & 0 \end{array}\right]=0,\;\;\;\left(\mathrm{iii}\right)\;\;\left[\begin{array}{ccc} A_{1}A_{2}-A & A_{1}B_{2} & B_{1}\\ E_{1}A_{2} & E_{1}B_{2} & 0\\ E_{2} & 0 & 0 \end{array}\right]=0.$$
(c) *The matrix equation*
  (5.13) $$(A_{1}+B_{1}X_{1}C_{1})(A_{2}+B_{2}X_{2}C_{2}+D_{2}Y_{2}E_{2})=A$$
  *holds for all matrices* $X_{1}$*,* $X_{2}$*, and* $Y_{2}$ *if and only if one of the following 8 block matrix equalities holds:*
  $$\begin{array}{cccc} \left(i\right)\; & [A_{1}A_{2}-A,\;A_{1}B_{2},\;A_{1}D_{2},\;B_{1}]=0,\; & \left(\mathrm{ii}\right)\; & \left[\begin{array}{ccc} A_{1}A_{2}-A & A_{1}D_{2} & B_{1}\\ C_{2} & 0 & 0 \end{array}\right]=0,\\ \left(\mathrm{iii}\right)\; & \left[\begin{array}{ccc} A_{1}A_{2}-A & A_{1}B_{2} & B_{1}\\ E_{2} & 0 & 0 \end{array}\right]=0,\; & \left(\mathrm{iv}\right)\; & \left[\begin{array}{ccc} A_{1}A_{2}-A & A_{1}B_{2} & A_{1}D_{2}\\ C_{1}A_{2} & C_{1}B_{2} & C_{1}D_{2} \end{array}\right]=0,\\ \left(v\right)\; & \left[\begin{array}{cc} A_{1}A_{2}-A & B_{1}\\ C_{2} & 0\\ E_{2} & 0 \end{array}\right]=0,\; & \left(\mathrm{vi}\right)\; & \left[\begin{array}{cc} A_{1}A_{2}-A & A_{1}D_{2}\\ C_{1}A_{2} & C_{1}D_{2}\\ C_{2} & 0 \end{array}\right]=0,\\ \left(\mathrm{vii}\right)\; & \left[\begin{array}{cc} A_{1}A_{2}-A & A_{1}B_{2}\\ C_{1}A_{2} & C_{1}B_{2}\\ E_{2} & 0 \end{array}\right]=0,\; & \left(\mathrm{viii}\right)\; & \left[\begin{array}{c} A_{1}A_{2}-A\\ C_{1}A_{2}\\ C_{2}\\ E_{2} \end{array}\right]=0. \end{array}$$
(d) *The matrix equation*
  (5.14) $$(A_{1}+B_{1}X_{1}C_{1}+D_{1}Y_{1}E_{1})(A_{2}+B_{2}X_{2}C_{2})=A$$
  *holds for all matrices* $X_{1}$*,* $Y_{1}$*, and* $X_{2}$ *if and only if one of the following 8 block matrix equalities holds:*
  $$\begin{array}{cccc} \left(i\right)\; & [A_{1}A_{2}-A,\;A_{1}B_{2},\;B_{1},\;D_{1}]=0,\; & \left(\mathrm{ii}\right)\; & \left[\begin{array}{ccc} A_{1}A_{2}-A & B_{1} & D_{1}\\ C_{2} & 0 & 0 \end{array}\right]=0,\\ \left(\mathrm{iii}\right)\; & \left[\begin{array}{ccc} A_{1}A_{2}-A & A_{1}B_{2} & B_{1}\\ E_{1}A_{2} & E_{1}B_{2} & 0 \end{array}\right]=0,\; & \left(\mathrm{iv}\right)\; & \left[\begin{array}{ccc} A_{1}A_{2}-A & A_{1}B_{2} & D_{1}\\ C_{1}A_{2} & C_{1}B_{2} & 0 \end{array}\right]=0,\\ \left(v\right)\; & \left[\begin{array}{cc} A_{1}A_{2}-A & B_{1}\\ E_{1}A_{2} & 0\\ C_{2} & 0 \end{array}\right]=0,\; & \left(\mathrm{vi}\right)\; & \left[\begin{array}{cc} A_{1}A_{2}-A & D_{1}\\ C_{1}A_{2} & 0\\ C_{2} & 0 \end{array}\right]=0,\\ \left(\mathrm{vii}\right)\; & \left[\begin{array}{cc} A_{1}A_{2}-A & A_{1}B_{2}\\ C_{1}A_{2} & C_{1}B_{2}\\ E_{1}A_{2} & E_{1}B_{2} \end{array}\right]=0,\; & \left(\mathrm{viii}\right)\; & \left[\begin{array}{c} A_{1}A_{2}-A\\ C_{1}A_{2}\\ E_{1}A_{2}\\ C_{2} \end{array}\right]=0. \end{array}$$
(e) *The bilinear matrix equation*
  (5.15) $$M_{1}(A_{1}+B_{1}X_{1}+Y_{1}C_{1})M_{2}(A_{2}+B_{2}X_{2}+Y_{2}C_{2})M_{3}=M$$
  *holds for all matrices* $X_{1}$*,* $X_{2}$*,* $Y_{1}$*, and* $Y_{2}$ *if and only if one of the following 6 block matrix equalities holds*
  $$\begin{array}{cc} \left(i\right)\; & [M,\;M_{1}]=0,\;\;\;\;\;\;\;\;\left(\mathrm{ii}\right)\;\;\left[\begin{array}{c} M\\ M_{3} \end{array}\right]=0,\;\;\;\;\;\;\;\;\left(\mathrm{iii}\right)\;\;\left[\begin{array}{cc} M & 0\\ 0 & M_{2} \end{array}\right]=0,\\ \left(\mathrm{iv}\right)\; & \left[\begin{array}{ccc} M & M_{1}A_{1}M_{2} & M_{1}B_{1}\\ 0 & C_{1}M_{2} & 0 \end{array}\right]=0,\;\;\left(v\right)\;\left[\begin{array}{cc} M & 0\\ M_{2}A_{2}M_{3} & M_{2}B_{2}\\ C_{2}M_{3} & 0 \end{array}\right]=0,\;\;\left(\mathrm{vi}\right)\;\;\left[\begin{array}{ccc} M_{1}A_{1}M_{2}A_{2}M_{3}-M & M_{1}A_{1}M_{2}B_{2} & M_{1}B_{1}\\ C_{1}M_{2}A_{2}M_{3} & C_{1}M_{2}B_{2} & 0\\ C_{2}M_{3} & 0 & 0 \end{array}\right]=0. \end{array}$$
(f) *The matrix equation*
  (5.16) $$(A_{1}+B_{1}X_{1}C_{1})(A_{2}+B_{2}X_{2}+Y_{2}E_{2})=A$$
  *holds for all matrices* $X_{1}$*,* $X_{2}$*, and* $Y_{2}$ *if and only if one of the following 4 block matrix equalities holds:*
  $$\left(i\right)\;[A,\;A_{1},\;B_{1}]=0,\;\;\;\left(\mathrm{ii}\right)\;\left[\begin{array}{ccc} A_{1}A_{2}-A & A_{1}B_{2} & B_{1}\\ E_{2} & 0 & 0 \end{array}\right]=0,\;\;\;\left(\mathrm{iii}\right)\;\left[\begin{array}{cc} A & A_{1}\\ 0 & C_{1} \end{array}\right]=0,\;\;\;\left(\mathrm{iv}\right)\;\left[\begin{array}{cc} A_{1}A_{2}-A & A_{1}B_{2}\\ C_{1}A_{2} & C_{1}B_{2}\\ E_{2} & 0 \end{array}\right]=0.$$
(g) *The matrix equation*
  (5.17) $$(A_{1}+B_{1}X_{1}+Y_{1}E_{1})(A_{2}+B_{2}X_{2}C_{2})=A$$
  *holds for all matrices* $X_{1}$*,* $Y_{1}$*, and* $X_{2}$ *if and only if one of the following 4 block matrix equalities holds:*
  $$\left(i\right)\;\left[\begin{array}{ccc} A_{1}A_{2}-A & A_{1}B_{2} & B_{1}\\ E_{1}A_{2} & E_{1}B_{2} & 0 \end{array}\right]=0,\;\;\;\left(\mathrm{ii}\right)\;\left[\begin{array}{cc} A_{1}A_{2}-A & B_{1}\\ E_{1}A_{2} & 0\\ C_{2} & 0 \end{array}\right]=0,\;\;\;\left(\mathrm{iii}\right)\;\left[\begin{array}{cc} A & 0\\ A_{2} & B_{2} \end{array}\right]=0,\;\;\;\left(\mathrm{iv}\right)\;\left[\begin{array}{c} A\\ A_{2}\\ C_{2} \end{array}\right]=0.$$

Lemma 5.8 *Let*

(5.18) $$(A_{1}+B_{1}X_{1}C_{1})(A_{2}+B_{2}X_{2}C_{2})(A_{3}+B_{3}X_{3}C_{3})(A_{4}+B_{4}X_{4}C_{4})=A$$

*be a given multilinear matrix equation, where A,* $A_{i}$*,* $B_{i}$*, and* $C_{i}$ *are known matrices of appropriate sizes,* i=1,2,3,4*. Then the following results hold.*

(a) *Eq.* (5.18) *holds for all matrices* $X_{1}$*,* $X_{2}$*,* $X_{3}$*, and* $X_{4}$ *if and only if one of the following 16 block matrix equalities holds*
  $$\begin{array}{cccc} \left(i\right)\; & [A_{1}A_{2}A_{3}A_{4}-A,\;A_{1}A_{2}A_{3}B_{4},\;A_{1}A_{2}B_{3},\;A_{1}B_{2},\;B_{1}]=0,\; & \left(\mathrm{ii}\right)\; & \left[\begin{array}{cccc} A_{1}A_{2}A_{3}A_{4}-A & A_{1}A_{2}B_{3} & A_{1}B_{2} & B_{1}\\ C_{4} & 0 & 0 & 0 \end{array}\right]=0,\\ \left(\mathrm{iii}\right)\; & \left[\begin{array}{cccc} A_{1}A_{2}A_{3}A_{4}-A & A_{1}A_{2}A_{3}B_{4} & A_{1}B_{2} & B_{1}\\ C_{3}A_{4} & C_{3}B_{4} & 0 & 0 \end{array}\right]=0,\; & \left(\mathrm{iv}\right)\; & \left[\begin{array}{cccc} A_{1}A_{2}A_{3}A_{4}-A & A_{1}A_{2}A_{3}B_{4} & A_{1}A_{2}B_{3} & B_{1}\\ C_{2}A_{3}A_{4} & C_{2}A_{3}B_{4} & C_{2}B_{3} & 0 \end{array}\right]=0,\\ \left(v\right)\; & \left[\begin{array}{cccc} A_{1}A_{2}A_{3}A_{4}-A & A_{1}A_{2}A_{3}B_{4} & A_{1}A_{2}B_{3} & A_{1}B_{2}\\ C_{1}A_{2}A_{3}A_{4} & C_{1}A_{2}A_{3}B_{4} & C_{1}A_{2}B_{3} & C_{1}B_{2} \end{array}\right]=0,\; & \left(\mathrm{vi}\right)\; & \left[\begin{array}{ccc} A_{1}A_{2}A_{3}A_{4}-A & A_{1}B_{2} & B_{1}\\ C_{3}A_{4} & 0 & 0\\ C_{4} & 0 & 0 \end{array}\right]=0,\\ \left(\mathrm{vii}\right)\; & \left[\begin{array}{ccc} A_{1}A_{2}A_{3}A_{4}-A & A_{1}A_{2}B_{3} & B_{1}\\ C_{2}A_{3}A_{4} & C_{2}B_{3} & 0\\ C_{4} & 0 & 0 \end{array}\right]=0,\; & \left(\mathrm{viii}\right)\; & \left[\begin{array}{ccc} A_{1}A_{2}A_{3}A_{4}-A & A_{1}A_{2}B_{3} & A_{1}B_{2}\\ C_{1}A_{2}A_{3}A_{4} & C_{1}A_{2}B_{3} & C_{1}B_{2}\\ C_{4} & 0 & 0 \end{array}\right]=0,\\ \left(\mathrm{ix}\right)\; & \left[\begin{array}{ccc} A_{1}A_{2}A_{3}A_{4}-A & A_{1}A_{2}A_{3}B_{4} & B_{1}\\ C_{2}A_{3}A_{4} & C_{2}A_{3}B_{4} & 0\\ C_{3}A_{4} & C_{3}B_{4} & 0 \end{array}\right]=0,\; & \left(x\right)\; & \left[\begin{array}{ccc} A_{1}A_{2}A_{3}A_{4}-A & A_{1}A_{2}A_{3}B_{4} & A_{1}B_{2}\\ C_{1}A_{2}A_{3}A_{4} & C_{1}A_{2}A_{3}B_{4} & C_{1}B_{2}\\ C_{3}A_{4} & C_{3}B_{4} & 0 \end{array}\right]=0,\\ \left(\mathrm{xi}\right)\; & \left[\begin{array}{ccc} A_{1}A_{2}A_{3}A_{4}-A & A_{1}A_{2}A_{3}B_{4} & A_{1}A_{2}B_{3}\\ C_{1}A_{2}A_{3}A_{4} & C_{1}A_{2}A_{3}B_{4} & C_{1}A_{2}B_{3}\\ C_{2}A_{3}A_{4} & C_{2}A_{3}B_{4} & C_{2}B_{3} \end{array}\right]=0,\; & \left(\mathrm{xii}\right)\; & \left[\begin{array}{cc} A_{1}A_{2}A_{3}A_{4}-A & B_{1}\\ C_{2}A_{3}A_{4} & 0\\ C_{3}A_{4} & 0\\ C_{4} & 0 \end{array}\right]=0,\\ \left(\mathrm{xiii}\right)\; & \left[\begin{array}{cc} A_{1}A_{2}A_{3}A_{4}-A & A_{1}B_{2}\\ C_{1}A_{2}A_{3}A_{4} & C_{1}B_{2}\\ C_{3}A_{4} & 0\\ C_{4} & 0 \end{array}\right]=0,\; & \left(\mathrm{xiv}\right)\; & \left[\begin{array}{cc} A_{1}A_{2}A_{3}A_{4}-A & A_{1}A_{2}B_{3}\\ C_{1}A_{2}A_{3}A_{4} & C_{1}A_{2}B_{3}\\ C_{2}A_{3}A_{4} & C_{2}B_{3}\\ C_{4} & 0 \end{array}\right]=0,\\ \left(\mathrm{xv}\right)\; & \left[\begin{array}{cc} A_{1}A_{2}A_{3}A_{4}-A & A_{1}A_{2}A_{3}B_{4}\\ C_{1}A_{2}A_{3}A_{4} & C_{1}A_{2}A_{3}B_{4}\\ C_{2}A_{3}A_{4} & C_{2}A_{3}B_{4}\\ C_{3}A_{4} & C_{3}B_{4} \end{array}\right]=0,\; & \left(\mathrm{xvi}\right)\; & \left[\begin{array}{c} A_{1}A_{2}A_{3}A_{4}-A\\ C_{1}A_{2}A_{3}A_{4}\\ C_{2}A_{3}A_{4}\\ C_{3}A_{4}\\ C_{4} \end{array}\right]=0. \end{array}$$
(b) *Under the assumptions* $R(A_{1})\subseteq R(B_{1})$ *and* $R(A_{4}^{⁎})\subseteq R(C_{4}^{⁎})$*,* (5.18) *holds for all matrices* $X_{1}$*,* $X_{2}$*,* $X_{3}$*, and* $X_{4}$ *if and only if one of the following 6 block matrix equalities holds*
  $$\begin{array}{cc} \left(i\right)\; & [A,\;B_{1}]=0,\;\;\;\left(\mathrm{ii}\right)\;\left[\begin{array}{c} A\\ C_{4} \end{array}\right]=0,\;\;\;\left(\mathrm{iii}\right)\;\left[\begin{array}{cccc} A_{1}A_{2}A_{3}A_{4}-A & A_{1}A_{2}A_{3}B_{4} & A_{1}A_{2}B_{3} & A_{1}B_{2}\\ C_{1}A_{2}A_{3}A_{4} & C_{1}A_{2}A_{3}B_{4} & C_{1}A_{2}B_{3} & C_{1}B_{2} \end{array}\right]=0,\\ \left(\mathrm{iv}\right)\; & \left[\begin{array}{ccc} A_{1}A_{2}A_{3}A_{4}-A & A_{1}A_{2}A_{3}B_{4} & A_{1}B_{2}\\ C_{1}A_{2}A_{3}A_{4} & C_{1}A_{2}A_{3}B_{4} & C_{1}B_{2}\\ C_{3}A_{4} & C_{3}B_{4} & 0 \end{array}\right]=0,\;\;\;\left(v\right)\;\left[\begin{array}{ccc} A_{1}A_{2}A_{3}A_{4}-A & A_{1}A_{2}A_{3}B_{4} & A_{1}A_{2}B_{3}\\ C_{1}A_{2}A_{3}A_{4} & C_{1}A_{2}A_{3}B_{4} & C_{1}A_{2}B_{3}\\ C_{2}A_{3}A_{4} & C_{2}A_{3}B_{4} & C_{2}B_{3} \end{array}\right]=0,\;\;\;\left(\mathrm{vi}\right)\;\left[\begin{array}{cc} A_{1}A_{2}A_{3}A_{4}-A & A_{1}A_{2}A_{3}B_{4}\\ C_{1}A_{2}A_{3}A_{4} & C_{1}A_{2}A_{3}B_{4}\\ C_{2}A_{3}A_{4} & C_{2}A_{3}B_{4}\\ C_{3}A_{4} & C_{3}B_{4} \end{array}\right]=0. \end{array}$$
(c) *Under the assumption* $R(A_{1})\subseteq R(B_{1})$*,* $R(A_{2}^{⁎})\subseteq R(C_{2}^{⁎})$*,* $R(A_{3})\subseteq R(B_{3})$*, and* $R(A_{4}^{⁎})\subseteq R(C_{4}^{⁎})$*,* (5.18) *holds for all matrices* $X_{1}$*,* $X_{2}$*,* $X_{3}$*, and* $X_{4}$ *if and only if one of the following 6 block matrix equalities holds*
  $$\begin{array}{cc} \left(i\right)\; & [A,\;B_{1}]=0,\;\;\;\;\left(\mathrm{ii}\right)\left[\begin{array}{c} A\\ C_{4} \end{array}\right]=0,\;\;\;\;\left(\mathrm{iii}\right)\;\left[\begin{array}{cc} A & 0\\ 0 & C_{2}B_{3} \end{array}\right]=0,\;\;\;\left(\mathrm{iv}\right)\;\left[\begin{array}{ccc} A & A_{1}A_{2}B_{3} & A_{1}B_{2}\\ 0 & C_{1}A_{2}B_{3} & C_{1}B_{2} \end{array}\right]=0,\\ \left(v\right)\; & \left[\begin{array}{cc} A & 0\\ C_{2}A_{3}A_{4} & C_{2}A_{3}B_{4}\\ C_{3}A_{4} & C_{3}B_{4} \end{array}\right]=0,\;\;\;\;\;\;\;\;\;\;\;\;\;\;\;\;\;\;\;\left(\mathrm{vi}\right)\;\left[\begin{array}{ccc} A_{1}A_{2}A_{3}A_{4}-A & A_{1}A_{2}A_{3}B_{4} & A_{1}B_{2}\\ C_{1}A_{2}A_{3}A_{4} & C_{1}A_{2}A_{3}B_{4} & C_{1}B_{2}\\ C_{3}A_{4} & C_{3}B_{4} & 0 \end{array}\right]=0. \end{array}$$
(d) *The multilinear matrix equation*
  (5.19) $$M_{1}(A_{1}+B_{1}X_{1})M_{2}(A_{2}+X_{2}C_{2})M_{3}(A_{3}+B_{3}X_{3})M_{4}(A_{4}+X_{4}C_{4})M_{5}=A$$
  *holds for all variable matrices* $X_{1}$*,* $X_{2}$*,* $X_{3}$*, and* $X_{4}$ *if and only if one of the following 8 block matrix equalities holds*
  $$\begin{array}{cc} \left(i\right)\; & [A,\;M_{1}A_{1}M_{2},\;M_{1}B_{1}]=0,\;\;\;\left(\mathrm{ii}\right)\;\left[\begin{array}{c} A\\ M_{4}A_{4}M_{5}\\ C_{4}M_{5} \end{array}\right]=0,\;\;\;\left(\mathrm{iii}\right)\;\left[\begin{array}{cc} A & 0\\ 0 & M_{2} \end{array}\right]=0,\;\;\;\left(\mathrm{iv}\right)\;\left[\begin{array}{cc} A & 0\\ 0 & M_{4} \end{array}\right]=0,\\ \left(v\right)\; & \left[\begin{array}{cccc} A & M_{1}A_{1}M_{2}A_{2}M_{3}A_{3}M_{4} & M_{1}A_{1}M_{2}A_{2}M_{3}B_{3} & M_{1}B_{1}\\ 0 & C_{2}M_{3}A_{3}M_{4} & C_{2}M_{3}B_{3} & 0 \end{array}\right]=0,\;\;\;\left(\mathrm{vi}\right)\;\left[\begin{array}{cc} A & 0\\ M_{2}A_{2}M_{3}A_{3}M_{4}A_{4}M_{5} & M_{2}A_{2}M_{3}B_{3}\\ C_{2}M_{3}A_{3}M_{4}A_{4}M_{5} & C_{2}M_{3}B_{3}\\ C_{4}M_{5} & 0 \end{array}\right]=0,\\ \left(\mathrm{vii}\right)\; & \left[\begin{array}{ccc} A & 0 & 0\\ 0 & M_{2}A_{2}M_{3}A_{3}M_{4} & M_{2}A_{2}M_{3}B_{3}\\ 0 & C_{2}M_{3}A_{3}M_{4} & C_{2}M_{3}B_{3} \end{array}\right]=0,\;\;\;\left(\mathrm{viii}\right)\;\left[\begin{array}{ccc} M_{1}A_{1}M_{2}A_{2}M_{3}A_{3}M_{4}A_{4}M_{5}-A & M_{1}A_{1}M_{2}A_{2}M_{3}B_{3} & M_{1}B_{1}\\ C_{2}M_{3}A_{3}M_{4}A_{4}M_{5} & C_{2}M_{3}B_{3} & 0\\ C_{4}M_{5} & 0 & 0 \end{array}\right]=0. \end{array}$$

With a brief summary, we can say that Lemma 5.2, Lemma 5.8 demonstrate how to characterize linear or multilinear matrix identities by means of a group of block matrix equalities composed of the known matrices in the identities. These matrix equalities are easy to verify and simplify when the matrices in the identities are given with concrete expressions. In particular, if some or all the given matrices in (5.7)–(5.19) are known to be non-null, then some or most of the block matrix equalities in Lemma 5.2, Lemma 5.8 can be removed.

## 6 Invariance property of matrix products that involve two generalized inverses

The hundreds of matrix-valued functions in (3.89)–(3.181) show in a striking manner what are the difficulties in the classification and characterization of the reverse order law problems formulated in (1.8)–(1.12). In other words, we need to make considerable extra efforts from the very beginning in order to obtain a complete set of solutions to all these reverse order law problems.

For a matrix expression $f\left(A_{1}^{\left(i_{1},\ldots ,j_{1}\right)},\;A_{2}^{\left(i_{2},\ldots ,j_{2}\right)},\ldots ,A_{k}^{\left(i_{k},\ldots ,j_{k}\right)}\right)$ that involves generalized inverses, one of the most fundamental fact that people like to know is concerned with the invariance property of the matrix expression with respect to the choice of the generalized inverses. The invariance property of this matrix expression can be formulated as an equivalent fact that $f\left(A_{1}^{\left(i_{1},\ldots ,j_{1}\right)},\;A_{2}^{\left(i_{2},\ldots ,j_{2}\right)},\ldots ,A_{k}^{\left(i_{k},\ldots ,j_{k}\right)}\right)=A$ always holds for all the generalized inverses in the equality. Especially, people are interested in the uniqueness of matrix products (cf. [6], [38], [40], [42], [45], [53], [63], [66], [108], [118]) for expositions and some previous results. It can be seen from Lemma 3.3(h) that the invariance property (uniqueness) of the product $B^{\left(s_{2},\ldots ,t_{2}\right)}A^{\left(s_{1},\ldots ,t_{1}\right)}$ occurs in the study of ${\left(AB\right)}^{†}=B^{\left(s_{2},\ldots ,t_{2}\right)}A^{\left(s_{1},\ldots ,t_{1}\right)}$. Hence it is necessary to know conditions under which the product $B^{\left(s_{2},\ldots ,t_{2}\right)}A^{\left(s_{1},\ldots ,t_{1}\right)}$ is invariant with respect to choice of $A^{\left(s_{1},\ldots ,t_{1}\right)}$ and $B^{\left(s_{2},\ldots ,t_{2}\right)}$. Because $B^{†}A^{†}$ is unique for any two given matrices *A* and *B*, it can readily be seen from the definition of the Moore–Penrose inverse that the product $B^{\left(s_{2},\ldots ,t_{2}\right)}A^{\left(s_{1},\ldots ,t_{1}\right)}$ is invariant with respect to the choice of the eight commonly-used types of generalized inverses $A^{\left(s_{1},\ldots ,t_{1}\right)}$ and $B^{\left(s_{2},\ldots ,t_{2}\right)}$ if and only if the equality

(6.1) $$B^{\left(s_{2},\ldots ,t_{2}\right)}A^{\left(s_{1},\ldots ,t_{1}\right)}=B^{†}A^{†}$$

holds for all $A^{\left(s_{1},\ldots ,t_{1}\right)}$ and $B^{\left(s_{2},\ldots ,t_{2}\right)}$. Substituting the 63 matrix-valued functions in (3.89)–(3.151) into (6.1), respectively, will result in 63 linear or multilinear matrix equations. In this section, we only present the detailed results concerning the invariance property of 63 matrix equations in (6.1). The complete characterizations of these invariance property problems can be formulated by means of Lemma 5.2, Lemma 5.8, but the details are rather technical and tedious, and therefore are omitted here due to space limitation.

Theorem 6.1 *Let* $A\in C^{m\times n}$ *and* $B\in C^{n\times p}$ *be given. Then the following results hold.*

〈1〉 $B^{†}A^{\left(1,3,4\right)}$ *is invariant* ⇔ r(A)=m *or* $R(A^{⁎})⊇R(B)$*.*
〈2〉 $B^{†}A^{\left(1,2,4\right)}$ *is invariant* ⇔ AB=0 *or* r(A)=m*.*
〈3〉 $B^{†}A^{\left(1,2,3\right)}$ *is invariant* ⇔ A=0 *or* $R(A^{⁎})⊇R(B)$*.*
〈4〉 $B^{†}A^{\left(1,4\right)}$ *is invariant* ⇔ r(A)=m *or* B=0*.*
〈5〉 $B^{†}A^{\left(1,3\right)}$ *is invariant* ⇔ $R(A^{⁎})⊇R(B)$*.*
〈6〉 $B^{†}A^{\left(1,2\right)}$ *is invariant* ⇔ A=0 *or* B=0 *or* {r(A)=m *and* $R(A^{⁎})⊇R(B)\}$*.*
〈7〉 $B^{†}A^{\left(1\right)}$ *is invariant* ⇔ B=0 *or* {r(A)=m *and* $R(A^{⁎})⊇R(B)\}$*.*
〈8〉 $B^{\left(1,3,4\right)}A^{†}$ *is invariant* ⇔ r(B)=p *or* $R(A^{⁎})\subseteq R(B)$*.*
〈9〉 $B^{\left(1,3,4\right)}A^{\left(1,3,4\right)}$ *is invariant* ⇔ r(A)=r(B)=n *or* {r(A)=m *and* r(B)=p} *or* {r(A)=m *and* $R(A^{⁎})\subseteq R(B)\}$ *or* {r(B)=p *and* $R(A^{⁎})⊇R(B)\}$*.*
〈10〉 $B^{\left(1,3,4\right)}A^{\left(1,2,4\right)}$ *is invariant* ⇔ {AB=0 *and* r(B)=p} *or* {r(A)=m *and* r(B)=p} *or* {AB=0 *and* $R(A^{⁎})\subseteq R(B)\}$ *or* {r(A)=m *and* $R(A^{⁎})\subseteq R(B)\}$*.*
〈11〉 $B^{\left(1,3,4\right)}A^{\left(1,2,3\right)}$ *is invariant* ⇔ A=0 *or* {r(B)=p *and* $R(A^{⁎})⊇R(B)\}$ *or* r(A)=r(B)=n*.*
〈12〉 $B^{\left(1,3,4\right)}A^{\left(1,4\right)}$ *is invariant* ⇔ {r(A)=m *and* r(B)=p} *or* {r(A)=m *and* $R(A^{⁎})\subseteq R(B)\}$*.*
〈13〉 $B^{\left(1,3,4\right)}A^{\left(1,3\right)}$ *is invariant* ⇔ r(A)=r(B)=n *or* {r(B)=p *and* $R(A^{⁎})⊇R(B)\}$*.*
〈14〉 $B^{\left(1,3,4\right)}A^{\left(1,2\right)}$ *is invariant* ⇔ A=0 *or* r(A)=r(B)=m=n *or* {AB=0,r(A)=m,andr(B)=p}*.*
〈15〉 $B^{\left(1,3,4\right)}A^{\left(1\right)}$ *is invariant* ⇔ r(A)=r(B)=m=n *or* {r(A)=m*,* r(B)=p*, and* $R(A^{⁎})⊇R(B)\}$*.*
〈16〉 $B^{\left(1,2,4\right)}A^{†}$ *is invariant* ⇔ B=0 *or* $R(A^{⁎})\subseteq R(B)$*.*
〈17〉 $B^{\left(1,2,4\right)}A^{\left(1,3,4\right)}$ *is invariant* ⇔ B=0 *or* {r(A)=m *and* $R(A^{⁎})\subseteq R(B)\}$ *or* r(A)=r(B)=n*.*
〈18〉 $B^{\left(1,2,4\right)}A^{\left(1,2,4\right)}$ *is invariant* ⇔ A=0 *or* B=0 *or* {r(A)=m *and* $R(A^{⁎})\subseteq R(B)\}$*.*
〈19〉 $B^{\left(1,2,4\right)}A^{\left(1,2,3\right)}$ *is invariant* ⇔ A=0 *or* B=0 *or* r(A)=r(B)=n*.*
〈20〉 $B^{\left(1,2,4\right)}A^{\left(1,4\right)}$ *is invariant* ⇔ B=0 *or* {r(A)=m *and* $R(A^{⁎})\subseteq R(B)\}$*.*
〈21〉 $B^{\left(1,2,4\right)}A^{\left(1,3\right)}$ *is invariant* ⇔ B=0 *or* r(A)=r(B)=n*.*
〈22〉 $B^{\left(1,2,4\right)}A^{\left(1,2\right)}$ *is invariant* ⇔ A=0 *or* B=0 *or* r(A)=r(B)=m=n*.*
〈23〉 $B^{\left(1,2,4\right)}A^{\left(1\right)}$ *is invariant* ⇔ B=0 *or* r(A)=r(B)=m=n*.*
〈24〉 $B^{\left(1,2,3\right)}A^{†}$ *is invariant* ⇔ A=0 *or* B=0 *or* (B)=p*.*
〈25〉 $B^{\left(1,2,3\right)}A^{\left(1,3,4\right)}$ *is invariant* ⇔ {AB=0 *and* r(A)=m} *or* {r(A)=m *and* r(B)=p} *or* {AB=0 *and* $R(A^{⁎})⊇R(B)\}$ *or* {r(B)=p *and* $R(A^{⁎})⊇R(B)\}$*.*
〈26〉 $B^{\left(1,2,3\right)}A^{\left(1,2,4\right)}$ *is invariant* ⇔ AB=0 *or* {r(A)=m *and* r(B)=p}*.*
〈27〉 $B^{\left(1,2,3\right)}A^{\left(1,2,3\right)}$ *is invariant* ⇔ A=0 *or* B=0 *or* {r(B)=p *and* $R(A^{⁎})⊇R(B)\}$*.*
〈28〉 $B^{\left(1,2,3\right)}A^{\left(1,4\right)}$ *is invariant* ⇔ B=0 *or* {AB=0 *and* r(A)=m} *or* {r(A)=m *and* r(B)=p}*.*
〈29〉 $B^{\left(1,2,3\right)}A^{\left(1,3\right)}$ *is invariant* ⇔ B=0 *or* {r(B)=p *and* $R(A^{⁎})⊇R(B)\}$*.*
〈30〉 $B^{\left(1,2,3\right)}A^{\left(1,2\right)}$ *is invariant* ⇔ A=0 *or* B=0 *or* {r(A)=m*,* r(B)=p*, and* $R(A^{⁎})⊇R(B)\}$*.*
〈31〉 $B^{\left(1,2,3\right)}A^{\left(1\right)}$ *is invariant* ⇔ B=0 *or* {r(A)=m*,* r(B)=p*, and* $R(A^{⁎})⊇R(B)\}$*.*
〈32〉 $B^{\left(1,4\right)}A^{†}$ *is invariant* ⇔ $R(A^{⁎})\subseteq R(B)$*.*
〈33〉 $B^{\left(1,4\right)}A^{\left(1,3,4\right)}$ *is invariant* ⇔ r(A)=r(B)=n *or* {r(A)=m *and* $R(A^{⁎})\subseteq R(B)\}$*.*
〈34〉 $B^{\left(1,4\right)}A^{\left(1,2,4\right)}$ *is invariant* ⇔ A=0 *or* {r(A)=m *and* $R(A^{⁎})\subseteq R(B)\}$*.*
〈35〉 $B^{\left(1,4\right)}A^{\left(1,2,3\right)}$ *is invariant* ⇔ A=0 *or* r(A)=r(B)=n*.*
〈36〉 $B^{\left(1,4\right)}A^{\left(1,4\right)}$ *is invariant* ⇔ r(A)=m *and* $R(A^{⁎})\subseteq R(B)$*.*
〈37〉 $B^{\left(1,4\right)}A^{\left(1,3\right)}$ *is invariant* ⇔ r(A)=r(B)=n*.*
〈38〉 $B^{\left(1,4\right)}A^{\left(1,2\right)}$ *is invariant* ⇔ A=0 *or* r(A)=r(B)=m=n*.*
〈39〉 $B^{\left(1,4\right)}A^{\left(1\right)}$ *is invariant* ⇔ r(A)=r(B)=m=n*.*
〈40〉 $B^{\left(1,3\right)}A^{†}$ *is invariant* ⇔ r(B)=p*.*
〈41〉 $B^{\left(1,3\right)}A^{\left(1,3,4\right)}$ *is invariant* ⇔ {r(A)=m *and* r(B)=p} *or* {r(B)=p *and* $R(A^{⁎})⊇R(B)\}$*.*
〈42〉 $B^{\left(1,3\right)}A^{\left(1,2,4\right)}$ *is invariant* ⇔ A=0 *or* {AB=0 *and* r(B)=p} *or* {r(A)=m *and* r(B)=p}*.*
〈43〉 $B^{\left(1,3\right)}A^{\left(1,2,3\right)}$ *is invariant* ⇔ A=0 *or* {r(B)=p *and* $R(A^{⁎})⊇R(B)\}$*.*
〈44〉 $B^{\left(1,3\right)}A^{\left(1,4\right)}$ *is invariant* ⇔ r(A)=m *and* r(B)=p*.*
〈45〉 $B^{\left(1,3\right)}A^{\left(1,3\right)}$ *is invariant* ⇔ r(B)=p *and* $R(A^{⁎})⊇R(B)$*.*
〈46〉 $B^{\left(1,3\right)}A^{\left(1,2\right)}$ *is invariant* ⇔ A=0 *or* {r(A)=m*,* r(B)=p*, and* $R(A^{⁎})⊇R(B)\}$*.*
〈47〉 $B^{\left(1,3\right)}A^{\left(1\right)}$ *is invariant* ⇔ r(A)=m*,* r(B)=p*, and* $R(A^{⁎})⊇R(B)$*.*
〈48〉 $B^{\left(1,2\right)}A^{†}$ *is invariant* ⇔ A=0 *or* B=0 *or* {r(B)=p *and* $R(A^{⁎})\subseteq R(B)\}$*.*
〈49〉 $B^{\left(1,2\right)}A^{\left(1,3,4\right)}$ *is invariant* ⇔ B=0 *or* r(A)=r(B)=n=p *or* {AB=0,r(A)=m,andr(B)=p}*.*
〈50〉 $B^{\left(1,2\right)}A^{\left(1,2,4\right)}$ *is invariant* ⇔ A=0 *or* B=0 *or* {r(A)=m*,* r(B)=p*, and* $R(A^{⁎})\subseteq R(B)\}$*.*
〈51〉 $B^{\left(1,2\right)}A^{\left(1,2,3\right)}$ *is invariant* ⇔ A=0 *or* B=0 *or* r(A)=r(B)=n=p*.*
〈52〉 $B^{\left(1,2\right)}A^{\left(1,4\right)}$ *is invariant* ⇔ B=0 *or* {r(A)=m*,* r(B)=p*, and* $R(A^{⁎})\subseteq R(B)\}$*.*
〈53〉 $B^{\left(1,2\right)}A^{\left(1,3\right)}$ *is invariant* ⇔ B=0 *or* r(A)=r(B)=n=p*.*
〈54〉 $B^{\left(1,2\right)}A^{\left(1,2\right)}$ *is invariant* ⇔ A=0 *or* B=0 *or* r(A)=r(B)=m=n=p*.*
〈55〉 $B^{\left(1,2\right)}A^{\left(1\right)}$ *is invariant* ⇔ B=0 *or* r(A)=r(B)=m=n=p*.*
〈56〉 $B^{\left(1\right)}A^{†}$ *is invariant* ⇔ r(B)=p *and* $R(A^{⁎})\subseteq R(B)$*.*
〈57〉 $B^{\left(1\right)}A^{\left(1,3,4\right)}$ *is invariant* ⇔ r(A)=r(B)=n=p *or* {r(A)=m*,* r(B)=p*, and* $R(A^{⁎})\subseteq R(B)\}$*.*
〈58〉 $B^{\left(1\right)}A^{\left(1,2,4\right)}$ *is invariant* ⇔ A=0 *or* {r(A)=m*,* r(B)=p*, and* $R(A^{⁎})\subseteq R(B)\}$*.*
〈59〉 $B^{\left(1\right)}A^{\left(1,2,3\right)}$ *is invariant* ⇔ r(A)=r(B)=n=p*.*
〈60〉 $B^{\left(1\right)}A^{\left(1,4\right)}$ *is invariant* ⇔ r(A)=m*,* r(B)=p*, and* $R(A^{⁎})\subseteq R(B)$*.*
〈61〉 $B^{\left(1\right)}A^{\left(1,3\right)}$ *is invariant* ⇔ r(A)=r(B)=n=p*.*
〈62〉 $B^{\left(1\right)}A^{\left(1,2\right)}$ *is invariant* ⇔ A=0 *or* r(A)=r(B)=m=n=p*.*
〈63〉 $B^{\left(1\right)}A^{\left(1\right)}$ *is invariant* ⇔ r(A)=r(B)=m=n=p*.*

Proof We only give the proofs of Results 〈1〉, 〈54〉, and 〈63〉 as representatives. The product $B^{†}A^{\left(1,3,4\right)}$ in (3.89) is invariant iff $B^{†}A^{\left(1,3,4\right)}\equiv B^{†}A^{†}$, which is equivalent to $B^{†}F_{A}UE_{A}\equiv 0$, namely, $B^{†}F_{A}=0$ or $E_{A}=0$ by Lemma 5.1. These two equalities are further equivalent to the facts: $R(A^{⁎})⊇R(B)$ or r(A)=m by Lemma 4.3(a) and (b), establishing 〈1〉.

It follows from (3.142) that $B^{\left(1,2\right)}A^{\left(1,2\right)}\equiv B^{†}A^{†}$ can be rewritten as

$$(B^{†}+F_{B}V_{1})B(B^{†}+V_{2}E_{B})(A^{†}+F_{A}U_{1})A(A^{†}+U_{2}E_{A})\equiv B^{†}A^{†}.$$

By Lemma 5.8(d), the identity holds if and only if one of the following eight equalities hold

$$\begin{array}{c} \left(i\right)\;\;[B^{†}A^{†},\;B^{†}B,\;F_{B}]=0,\;\;\;\left(\mathrm{ii}\right)\left[\begin{array}{cc} B^{†}A^{†} & 0\\ 0 & B \end{array}\right]=0,\;\;\left(\mathrm{iii}\right)\;\;\left[\begin{array}{cc} B^{†}A^{†} & 0\\ 0 & A \end{array}\right]=0,\;\;\;\left(\mathrm{iv}\right)\;\;\left[\begin{array}{c} B^{†}A^{†}\\ AA^{†}\\ E_{A} \end{array}\right]=0,\\ \left(v\right)\;\;\left[\begin{array}{cccc} B^{†}A^{†} & B^{†}BB^{†}A^{†}A & B^{†}BB^{†}F_{A} & F_{B}\\ 0 & E_{B}A^{†}A & E_{B}F_{A} & 0 \end{array}\right]=0,\;\;\;\left(\mathrm{vi}\right)\;\;\left[\begin{array}{cc} B^{†}A^{†} & 0\\ BB^{†}A^{†}AA^{†} & BB^{†}F_{A}\\ E_{B}A^{†}AA^{†} & E_{B}F_{A}\\ E_{A} & 0 \end{array}\right]=0,\\ \left(\mathrm{vii}\right)\;\;\left[\begin{array}{ccc} B^{†}A^{†} & 0 & 0\\ 0 & BB^{†}A^{†}A & BB^{†}F_{A}\\ 0 & E_{B}A^{†}A & E_{B}F_{A} \end{array}\right]=0,\;\;\;\left(\mathrm{viii}\right)\;\;\left[\begin{array}{ccc} B^{†}BB^{†}A^{†}AA^{†}-B^{†}A^{†} & B^{†}BB^{†}F_{A} & F_{B}\\ E_{B}A^{†}AA^{†} & E_{B}F_{A} & 0\\ E_{A} & 0 & 0 \end{array}\right]=0, \end{array}$$

where (i) and (iv) are invalid because $B^{†}B+F_{B}=I_{p}$ and $AA^{†}+E_{A}=I_{m}$; (ii) is reduced to B=0; (iii) is reduced to A=0; (v) is a special case of (iii); (vi) is a special case of (iii); (vii) is a special case of (ii) and (iii); (viii) is reduced to $E_{A}=0$, $F_{A}=0$, $E_{B}=0$, and $F_{B}=0$, namely, A=0 or B=0 or r(A)=r(B)=m=n=p, thus establishing 〈54〉.

By (3.151), the identity $B^{\left(1\right)}A^{\left(1\right)}\equiv B^{†}A^{†}$ can be rewritten as

$$(B^{†}+F_{B}V_{1}+V_{2}E_{B})(A^{†}+F_{A}U_{1}+U_{2}E_{A})\equiv B^{†}A^{†}.$$

By Lemma 5.7(b), the identity holds if and only if

$$\left(i\right)\;\left[\begin{array}{ccc} B^{†}A^{†} & B^{†} & F_{B}\\ 0 & E_{B} & 0 \end{array}\right]=0,\;\left(\mathrm{ii}\right)\;\left[\begin{array}{cc} B^{†}A^{†} & 0\\ A^{†} & F_{A}\\ E_{A} & 0 \end{array}\right]=0,\;\left(\mathrm{iii}\right)\;\left[\begin{array}{ccc} B^{†}A^{†}-B^{†}A^{†} & B^{†}F_{A} & F_{B}\\ E_{B}A^{†} & E_{B}F_{A} & 0\\ E_{A} & 0 & 0 \end{array}\right]=0,$$

where (i) and (ii) are invalid because $B^{†}B+F_{B}=I_{p}$ and $AA^{†}+E_{A}=I_{m}$, and (iii) is reduced to $BB^{†}=I_{n}$, $B^{†}B=I_{p}$, $AA^{†}=I_{m}$, and $A^{†}A=I_{n}$, thus establishing 〈63〉. The remaining results can be shown by a similar way with rather technical and tedious details, and therefore we omit the proofs due to space limitation. □

The following results can be shown by a similar way.

Theorem 6.2 *Let* $A\in C^{m\times n}$ *and* $B\in C^{n\times p}$ *be given, and denote* $N=[A^{⁎},\;B]$*. Then the following results hold.*

〈1〉 *Eq.* (3.152) *is invariant* ⇔ r(A)=m *or* r(AB)=r(A)+r(B)−r(N)*.*
〈2〉 *Eq.* (3.153) *is invariant* ⇔ AB=0 *or* r(A)=m*.*
〈3〉 *Eq.* (3.154) *is invariant* ⇔ r(AB)=r(A)+r(B)−r(N)*.*
〈4〉 *Eq.* (3.155) *is invariant* ⇔ AB=0 *or* r(A)=m*.*
〈5〉 *Eq.* (3.156) *is invariant* ⇔ r(AB)=r(A)+r(B)−r(N)*.*
〈6〉 *Eq.* (3.157) *is invariant* ⇔ r(A)=m *or* r(AB)=r(A)+r(B)−r(N)*.*
〈7〉 *Eq.* (3.158) *is invariant* ⇔ r(A)=m *or* r(AB)=r(A)+r(B)−r(N)*.*
〈8〉 *Eq.* (3.159) *is invariant* ⇔ AB=0 *or* $R(A^{⁎})\subseteq R(B)$*.*
〈9〉 *Eq.* (3.160) *is invariant* ⇔ AB=0 *or* {r(AB)=n *or* r(A)=m *and* $R(A^{⁎})\subseteq R(B)\}$*.*
〈10〉 *Eq.* (3.161) *is invariant* ⇔ AB=0 *or* {r(A)=m *and* $R(A^{⁎})\subseteq R(B)\}$*.*
〈11〉 *Eq.* (3.162) *is invariant* ⇔ r(A)=r(B)=n*.*
〈12〉 *Eq.* (3.163) *is invariant* ⇔ AB=0 *or* {r(A)=m *and* $R(A^{⁎})\subseteq R(B)\}$*.*
〈13〉 *Eq.* (3.164) *is invariant* ⇔ AB=0 *or* r(A)=r(B)=n*.*
〈14〉 *Eq.* (3.165) *is invariant* ⇔ AB=0 *or* r(A)=r(B)=m=n*.*
〈15〉 *Eq.* (3.166) *is invariant* ⇔ AB=0 *or* r(A)=r(B)=m=n*.*
〈16〉 *Eq.* (3.167) *is invariant* ⇔ r(B)=p *or* r(AB)=r(A)+r(B)−r(N)*.*
〈17〉 *Eq.* (3.168) *is invariant* ⇔ r(N)=r(A)+r(B)−r(AB)*.*
〈18〉 *Eq.* (3.169) *is invariant* ⇔ AB=0 *or* r(B)=p*.*
〈19〉 *Eq.* (3.170) *is invariant* ⇔ r(AB)=r(A)+r(B)−r(N)*.*
〈20〉 *Eq.* (3.171) *is invariant* ⇔ AB=0 *or* r(B)=p*.*
〈21〉 *Eq.* (3.172) *is invariant* ⇔ r(B)=p *or* r(AB)=r(A)+r(B)−r(N)*.*
〈22〉 *Eq.* (3.173) *is invariant* ⇔ r(B)=p *or* r(AB)=r(A)+r(B)−r(N)*.*
〈23〉 *Eq.* (3.174) *is invariant* ⇔ AB=0 *or* $R(A^{⁎})⊇R(B)$*.*
〈24〉 *Eq.* (3.175) *is invariant* ⇔ AB=0 *or* r(A)=r(B)=n *or* {r(B)=p *and* $R(A^{⁎})⊇R(B)\}$*.*
〈25〉 *Eq.* (3.176) *is invariant* ⇔ AB=0 *or* r(A)=r(B)=n*.*
〈26〉 *Eq.* (3.177) *is invariant* ⇔ AB=0 *or* {r(B)=p *and* $R(A^{⁎})⊇R(B)\}$*.*
〈27〉 *Eq.* (3.178) *is invariant* ⇔ AB=0 *or* r(A)=r(B)=n*.*
〈28〉 *Eq.* (3.179) *is invariant* ⇔ AB=0 *or* {r(B)=p *and* $R(A^{⁎})⊇R(B)\}$*.*
〈29〉 *Eq.* (3.180) *is invariant* ⇔ AB=0 *or* r(A)=r(B)=n=p*.*
〈30〉 *Eq.* (3.181) *is invariant* ⇔ AB=0 *or* r(A)=r(B)=n=p*.*
〈31〉 *Eq.* (3.182) *is invariant* ⇔ r(AB)=r(A)+r(B)−r(N)*.*
〈32〉 *Eq.* (3.183) *is invariant* ⇔ r(AB)=r(A)+r(B)−r(N)*.*
〈33〉 *Eq.* (3.184) *is invariant* ⇔ AB=0 *or* r(AB)=r(A)+r(B)−n*.*

Besides, invariance properties of product of generalized inverses can also considered in other algebraic systems, see [46] for a similar work concerning linear and multilinear functional identities in a prime ring and their applications in the characterization of invariance properties of products of generalized inverses of two elements.

## 7 Fundamental equalities between generalized inverses of two matrices

The simplest matrix equality for generalized inverses in (1.5) is given by $A^{\left(i,\ldots ,j\right)}=B^{\left(s,\ldots ,t\right)}$. Necessary and sufficient conditions for these equalities to hold are currently being worked out by the present author in [100] as follows.

Lemma 7.1 *Let* $A,\;B\in C^{m\times n}$ *be given. Then the following 24 results hold.*

〈1〉 $A^{†}=B^{†}\Leftrightarrow A=B$*.*
〈2〉 $A^{†}\in \{B^{\left(1,3,4\right)}\}$ ⇔ $B^{⁎}A=B^{⁎}B$ *and* $AB^{⁎}=BB^{⁎}$*.*
〈3〉 $A^{†}\in \{B^{\left(1,2,4\right)}\}$ ⇔ $R(A^{⁎})=R(B^{⁎})$ *and* $A^{⁎}A=A^{⁎}B$*.*
〈4〉 $A^{†}\in \{B^{\left(1,4\right)}\}$ ⇔ $R(A^{⁎})⊇R(B^{⁎})$ *and* $A^{⁎}AB^{⁎}=A^{⁎}BB^{⁎}$*.*
〈5〉 $A^{†}\in \{B^{\left(1,2\right)}\}$ ⇔ r(A)=r(B) *and* $A^{⁎}AA^{⁎}=A^{⁎}BA^{⁎}$*.*
〈6〉 $A^{†}\in \{B^{\left(1\right)}\}$ ⇔ $r(A^{⁎}AA^{⁎}-A^{⁎}BA^{⁎})=r(A)-r(B)$*.*
〈7〉 $\{A^{\left(1,3,4\right)}\}\cap \{B^{\left(1,3,4\right)}\}\neq \emptyset$ ⇔ $R\left[\begin{array}{c} A\\ B \end{array}\right]\subseteq R\left[\begin{array}{c} AA^{⁎}\\ BB^{⁎} \end{array}\right]$*,* $R\left[\begin{array}{c} A^{⁎}\\ B^{⁎} \end{array}\right]\subseteq R\left[\begin{array}{c} A^{⁎}A\\ B^{⁎}B \end{array}\right]$*,* $A^{⁎}AB^{⁎}=A^{⁎}BB^{⁎}$*, and* $B^{⁎}AA^{⁎}=B^{⁎}BA^{⁎}$*.*
〈8〉 $\{A^{\left(1,3,4\right)}\}\cap \{B^{\left(1,2,4\right)}\}\neq \emptyset$ ⇔ $R\left[\begin{array}{c} A\\ B \end{array}\right]\subseteq R\left[\begin{array}{c} AA^{⁎}\\ BB^{⁎} \end{array}\right]$ *and* $A^{⁎}A=A^{⁎}B$*.*
〈9〉 $\{A^{\left(1,3,4\right)}\}\cap \{B^{\left(1,4\right)}\}\neq \emptyset$ ⇔ $R\left[\begin{array}{c} A\\ B \end{array}\right]\subseteq R\left[\begin{array}{c} AA^{⁎}\\ BB^{⁎} \end{array}\right]$ *and* $A^{⁎}AB^{⁎}=A^{⁎}BB^{⁎}$*.*
〈10〉 $\{A^{\left(1,3,4\right)}\}\cap \{B^{\left(1,2\right)}\}\neq \emptyset$ ⇔ $R\left[\begin{array}{c} A^{⁎}B\\ B \end{array}\right]\subseteq R\left[\begin{array}{c} A^{⁎}A\\ B \end{array}\right]$*,* $R\left[\begin{array}{c} AB^{⁎}\\ B^{⁎} \end{array}\right]\subseteq R\left[\begin{array}{c} AA^{⁎}\\ B^{⁎} \end{array}\right]$*, and* $A^{⁎}AA^{⁎}=A^{⁎}BA^{⁎}$*.*
〈11〉 $\{A^{\left(1,3,4\right)}\}\cap \{B^{\left(1\right)}\}\neq \emptyset$ ⇔ $R\left[\begin{array}{c} A^{⁎}B\\ B \end{array}\right]\subseteq R\left[\begin{array}{c} A^{⁎}A\\ B \end{array}\right]$ *and* $R\left[\begin{array}{c} AB^{⁎}\\ B^{⁎} \end{array}\right]\subseteq R\left[\begin{array}{c} AA^{⁎}\\ B^{⁎} \end{array}\right]$*.*
〈12〉 $\{A^{\left(1,2,4\right)}\}\cap \{B^{\left(1,2,4\right)}\}\neq \emptyset$ ⇔ $R(A^{⁎})=R(B^{⁎})$ *and* $R\left[\begin{array}{c} A\\ B \end{array}\right]\subseteq R\left[\begin{array}{c} AA^{⁎}\\ BB^{⁎} \end{array}\right]$*.*
〈13〉 $\{A^{\left(1,2,4\right)}\}\cap \{B^{\left(1,2,3\right)}\}\neq \emptyset$ ⇔ $r(AB^{⁎})=r(B^{⁎}A)=r(A)=r(B)$ *and* $B^{⁎}AA^{⁎}=B^{⁎}BA^{⁎}$*.*
〈14〉 $\{A^{\left(1,2,4\right)}\}\cap \{B^{\left(1,4\right)}\}\neq \emptyset$ ⇔ $R(A^{⁎})⊇R(B^{⁎})$ *and* $R\left[\begin{array}{c} A\\ B \end{array}\right]\subseteq R\left[\begin{array}{c} AA^{⁎}\\ BB^{⁎} \end{array}\right]$*.*
〈15〉 $\{A^{\left(1,2,4\right)}\}\cap \{B^{\left(1,3\right)}\}\neq \emptyset$ ⇔ $R(BA^{⁎})=R(B)$ *and* $B^{⁎}AA^{⁎}=B^{⁎}BA^{⁎}$*.*
〈16〉 $\{A^{\left(1,2,4\right)}\}\cap \{B^{\left(1,2\right)}\}\neq \emptyset$ ⇔ $R(AB^{⁎})=R(A)$*,* $R(BA^{⁎})=R(B)$*, and* $R\left[\begin{array}{c} AB^{⁎}\\ B^{⁎} \end{array}\right]\subseteq R\left[\begin{array}{c} AA^{⁎}\\ B^{⁎} \end{array}\right]$*.*
〈17〉 $\{A^{\left(1,2,4\right)}\}\cap \{B^{\left(1\right)}\}\neq \emptyset$ ⇔ $R(BA^{⁎})=R(B)$ *and* $R\left[\begin{array}{c} AB^{⁎}\\ B^{⁎} \end{array}\right]\subseteq R\left[\begin{array}{c} AA^{⁎}\\ B^{⁎} \end{array}\right]$*.*
〈18〉 $\{A^{\left(1,4\right)}\}\cap \{B^{\left(1,4\right)}\}\neq \emptyset$ ⇔ $R\left[\begin{array}{c} A\\ B \end{array}\right]\subseteq R\left[\begin{array}{c} AA^{⁎}\\ BB^{⁎} \end{array}\right]$*.*
〈19〉 $\{A^{\left(1,4\right)}\}\cap \{B^{\left(1,3\right)}\}\neq \emptyset$ ⇔ $B^{⁎}AA^{⁎}=B^{⁎}BA^{⁎}$*.*
〈20〉 $\{A^{\left(1,4\right)}\}\cap \{B^{\left(1,2\right)}\}\neq \emptyset$ ⇔ $R(AB^{⁎})=R(A)$ *and* $R\left[\begin{array}{c} AB^{⁎}\\ B^{⁎} \end{array}\right]\subseteq R\left[\begin{array}{c} AA^{⁎}\\ B^{⁎} \end{array}\right]$*.*
〈21〉 $\{A^{\left(1,4\right)}\}\cap \{B^{\left(1\right)}\}\neq \emptyset$ ⇔ $R\left[\begin{array}{c} AB^{⁎}\\ B^{⁎} \end{array}\right]\subseteq R\left[\begin{array}{c} AA^{⁎}\\ B^{⁎} \end{array}\right]$*.*
〈22〉 $\{A^{\left(1,2\right)}\}\cap \{B^{\left(1,2\right)}\}\neq \emptyset$ ⇔ $r(A-B)=r\left[\begin{array}{c} A\\ B \end{array}\right]+r[A,\;B]-r(A)-r(B)$ *and* r(A)=r(B)*.*
〈23〉 $\{A^{\left(1,2\right)}\}\cap \{B^{\left(1\right)}\}\neq \emptyset$ ⇔ $r(A-B)=r\left[\begin{array}{c} A\\ B \end{array}\right]+r[A,\;B]-r(A)-r(B)$ *and* r(A)≥r(B)*.*
〈24〉 $\{A^{\left(1\right)}\}\cap \{B^{\left(1\right)}\}\neq \emptyset$ ⇔ $r(A-B)=r\left[\begin{array}{c} A\\ B \end{array}\right]+r[A,\;B]-r(A)-r(B)$*.*

Lemma 7.2 *Let* $A,\;B\in C^{m\times n}$ *be given. Then the following results hold.*

〈1a〉 $\left\{A^{\left(1,3,4\right)}\}\subseteq \{B^{\left(1,3,4\right)}\right\}$ ⇔ $B^{⁎}A=B^{⁎}B$ *and* $AB^{⁎}=BB^{⁎}$*.*
〈1b〉 $\left\{A^{\left(1,3,4\right)}\}⊇\{B^{\left(1,3,4\right)}\right\}$ ⇔ $A^{⁎}A=A^{⁎}B$ *and* $AA^{⁎}=BA^{⁎}$*.*
〈1c〉 $\left\{A^{\left(1,3,4\right)}\}=\{B^{\left(1,3,4\right)}\right\}$ ⇔ A=B*.*
〈2a〉 $\left\{A^{\left(1,3,4\right)}\}\subseteq \{B^{\left(1,2,4\right)}\right\}$ ⇔ {r(A)=m *and* A=B} *or* {r(A)=r(B)=n *and* $A^{⁎}A=A^{⁎}B\}$*.*
〈2b〉 $\left\{A^{\left(1,3,4\right)}\}⊇\{B^{\left(1,2,4\right)}\right\}$ ⇔ A=0 *or {*r(B)=m*,* $A^{⁎}A=A^{⁎}B$*, and* $AA^{⁎}=BA^{⁎}\}$*.*
〈2c〉 $\left\{A^{\left(1,3,4\right)}\}=\{B^{\left(1,2,4\right)}\right\}$ ⇔ r(A)=m *and* A=B*.*
〈3a〉 $\left\{A^{\left(1,3,4\right)}\}\subseteq \{B^{\left(1,4\right)}\right\}$ ⇔ $\left\{R(A^{⁎})⊇R(B^{⁎}\right)$ *and* $AB^{⁎}=BB^{⁎}\}$ *or* {r(A)=n *and* $A^{⁎}AB^{⁎}=A^{⁎}BB^{⁎}\}$*.*
〈3b〉 $\left\{A^{\left(1,3,4\right)}\}⊇\{B^{\left(1,4\right)}\right\}$ ⇔ A=0 *or {*r(B)=m*,* $A^{⁎}A=A^{⁎}B$*, and* $AA^{⁎}=BA^{⁎}\}$*.*
〈3c〉 $\left\{A^{\left(1,3,4\right)}\}=\{B^{\left(1,4\right)}\right\}$ ⇔ A=B=0 *or* {r(A)=m *and* A=B}*.*
〈4a〉 $\left\{A^{\left(1,3,4\right)}\}\subseteq \{B^{\left(1,2\right)}\right\}$ ⇔ {r(A)=r(B)=m *and* $AA^{⁎}=BA^{⁎}\}$ *or* {r(A)=r(B)=n *and* $A^{⁎}A=A^{⁎}B\}$*.*
〈4b〉 $\left\{A^{\left(1,3,4\right)}\}⊇\{B^{\left(1,2\right)}\right\}$ ⇔ A=0 *or {*r(B)=m=n*,* $A^{⁎}A=A^{⁎}B$*, and* $AA^{⁎}=BA^{⁎}\}$*.*
〈4c〉 $\left\{A^{\left(1,3,4\right)}\}=\{B^{\left(1,2\right)}\right\}$ ⇔ r(A)=m=n *and* A=B*.*
〈5a〉 $\left\{A^{\left(1,3,4\right)}\}\subseteq \{B^{\left(1\right)}\right\}$ ⇔ $R\left[\begin{array}{c} AA^{⁎}\\ BA^{⁎} \end{array}\right]⊇R\left[\begin{array}{c} B\\ B \end{array}\right]$ *or* $R\left[\begin{array}{c} A^{⁎}A\\ B^{⁎}A \end{array}\right]⊇R\left[\begin{array}{c} B^{⁎}\\ B^{⁎} \end{array}\right]$*.*
〈5b〉 $\left\{A^{\left(1,3,4\right)}\}⊇\{B^{\left(1\right)}\right\}$ ⇔ A=0 *or {*r(B)=m=n*,* $A^{⁎}A=A^{⁎}B$*, and* $AA^{⁎}=BA^{⁎}\}$*.*
〈5c〉 $\left\{A^{\left(1,3,4\right)}\}=\{B^{\left(1\right)}\right\}$ ⇔ A=B=0 *or* {r(A)=m=n *and* A=B}*.*
〈6〉 $\left\{A^{\left(1,2,4\right)}\}\subseteq \{B^{\left(1,2,4\right)}\right\}$ ⇔ $\left\{A^{\left(1,2,4\right)}\}⊇\{B^{\left(1,2,4\right)}\right\}$ ⇔ $\left\{A^{\left(1,2,4\right)}\}=\{B^{\left(1,2,4\right)}\right\}$ ⇔ A=B*.*
〈7a〉 $\left\{A^{\left(1,2,4\right)}\}\subseteq \{B^{\left(1,2,3\right)}\right\}$ ⇔ A=B=0 *or* {r(A)=r(B)=m *and* $AA^{⁎}=BA^{⁎}\}$*.*
〈7b〉 $\left\{A^{\left(1,2,4\right)}\}⊇\{B^{\left(1,2,3\right)}\right\}$ ⇔ A=B=0 *or* {r(A)=r(B)=n *and* $B^{⁎}A=B^{⁎}B\}$*.*
〈7c〉 $\left\{A^{\left(1,2,4\right)}\}=\{B^{\left(1,2,3\right)}\right\}$ ⇔ A=B=0 *or* {r(A)=m=n *and* A=B}*.*
〈8a〉 $\left\{A^{\left(1,2,4\right)}\}\subseteq \{B^{\left(1,4\right)}\right\}$ ⇔ $R(A^{⁎})⊇R(B^{⁎})$ *and* $AB^{⁎}=BB^{⁎}$*.*
〈8b〉 $\left\{A^{\left(1,2,4\right)}\}⊇\{B^{\left(1,4\right)}\right\}$ ⇔ r(A)=min⁡{m,n} *and* A=B*.*
〈8c〉 $\left\{A^{\left(1,2,4\right)}\}=\{B^{\left(1,4\right)}\right\}$ ⇔ r(A)=min⁡{m,n} *and* A=B*.*
〈9a〉 $\left\{A^{\left(1,2,4\right)}\}\subseteq \{B^{\left(1,3\right)}\right\}$ ⇔ B=0 *or* {r(A)=m *and* $B^{⁎}AA^{⁎}=B^{⁎}BA^{⁎}\}$*.*
〈9b〉 $\left\{A^{\left(1,2,4\right)}\}⊇\{B^{\left(1,3\right)}\right\}$ ⇔ r(A)=r(B)=n *and* $B^{⁎}A=B^{⁎}B$*.*
〈9c〉 $\left\{A^{\left(1,2,4\right)}\}=\{B^{\left(1,3\right)}\right\}$ ⇔ r(A)=m=n *and* A=B*.*
〈10a〉 $\left\{A^{\left(1,2,4\right)}\}\subseteq \{B^{\left(1,2\right)}\right\}$ ⇔ R(A)=R(B) *and* $AA^{⁎}=BA^{⁎}$*.*
〈10b〉 $\left\{A^{\left(1,2,4\right)}\}⊇\{B^{\left(1,2\right)}\right\}$ ⇔ A=B=0 *or* {r(A)=n *and* A=B}*.*
〈10c〉 $\left\{A^{\left(1,2,4\right)}\}=\{B^{\left(1,2\right)}\right\}$ ⇔ A=B=0 *or* {r(A)=n *and* A=B}*.*
〈11a〉 $\left\{A^{\left(1,2,4\right)}\}\subseteq \{B^{\left(1\right)}\right\}$ ⇔ $R\left[\begin{array}{c} AA^{⁎}\\ BA^{⁎} \end{array}\right]⊇R\left[\begin{array}{c} B\\ B \end{array}\right]$*.*
〈11b〉 $\left\{A^{\left(1,2,4\right)}\}⊇\{B^{\left(1\right)}\right\}$ ⇔ r(A)=n *and* A=B*.*
〈11c〉 $\left\{A^{\left(1,2,4\right)}\}=\{B^{\left(1\right)}\right\}$ ⇔ r(A)=n *and* A=B*.*
〈12a〉 $\left\{A^{\left(1,4\right)}\}\subseteq \{B^{\left(1,4\right)}\right\}$ ⇔ $R(A^{⁎})⊇R(B^{⁎})$ *and* $AB^{⁎}=BB^{⁎}$*.*
〈12b〉 $\left\{A^{\left(1,4\right)}\}⊇\{B^{\left(1,4\right)}\right\}$ ⇔ $R(A^{⁎})\subseteq R(B^{⁎})$ *and* $AA^{⁎}=BA^{⁎}$*.*
〈12c〉 $\left\{A^{\left(1,4\right)}\}=\{B^{\left(1,4\right)}\right\}$ ⇔ A=B*.*
〈13a〉 $\left\{A^{\left(1,4\right)}\}\subseteq \{B^{\left(1,3\right)}\right\}$ ⇔ B=0 *or* {r(A)=m *and* $B^{⁎}AA^{⁎}=B^{⁎}BA^{⁎}\}$*.*
〈13b〉 $\left\{A^{\left(1,4\right)}\}⊇\{B^{\left(1,3\right)}\right\}$ ⇔ A=0 *or* {r(B)=n *and* $B^{⁎}AA^{⁎}=B^{⁎}BA^{⁎}\}$*.*
〈13c〉 $\left\{A^{\left(1,4\right)}\}=\{B^{\left(1,3\right)}\right\}$ ⇔ A=B=0 *or* {r(A)=m*,* r(B)=n*, and* $B^{⁎}AA^{⁎}=B^{⁎}BA^{⁎}\}$*.*
〈14a〉 $\left\{A^{\left(1,4\right)}\}\subseteq \{B^{\left(1,2\right)}\right\}$ ⇔ r(A)=r(B)=min⁡{m,n} *and* $AA^{⁎}=BA^{⁎}$*.*
〈14b〉 $\left\{A^{\left(1,4\right)}\}⊇\{B^{\left(1,2\right)}\right\}$ ⇔ A=0 *or {*r(B)=n *and* $AA^{⁎}=BA^{⁎}\}$*.*
〈14c〉 $\left\{A^{\left(1,4\right)}\}=\{B^{\left(1,2\right)}\right\}$ ⇔ r(A)=r(B)=n *and* A=B*.*
〈15a〉 $\left\{A^{\left(1,4\right)}\}\subseteq \{B^{\left(1\right)}\right\}$ ⇔ $R\left[\begin{array}{c} AA^{⁎}\\ BA^{⁎} \end{array}\right]⊇R\left[\begin{array}{c} B\\ B \end{array}\right]$*.*
〈15b〉 $\left\{A^{\left(1,4\right)}\}⊇\{B^{\left(1\right)}\right\}$ ⇔ A=0 *or* {r(B)=n *and* $AA^{⁎}=BA^{⁎}\}$*.*
〈15c〉 $\left\{A^{\left(1,4\right)}\}=\{B^{\left(1\right)}\right\}$ ⇔ A=B=0 *or* {r(A)=n *and* A=B}*.*
〈16〉 $\left\{A^{\left(1,2\right)}\}\subseteq \{B^{\left(1,2\right)}\right\}$ ⇔ $\left\{A^{\left(1,2\right)}\}⊇\{B^{\left(1,2\right)}\right\}$ ⇔ $\left\{A^{\left(1,2\right)}\}=\{B^{\left(1,2\right)}\right\}$ ⇔ A=B*.*
〈17a〉 $\left\{A^{\left(1,2\right)}\}\subseteq \{B^{\left(1\right)}\right\}$ ⇔ r(A−B)=r(A)−r(B)*.*
〈17b〉 $\left\{A^{\left(1,2\right)}\}⊇\{B^{\left(1\right)}\right\}$ ⇔ r(A)=min⁡{m,n} *and* A=B*.*
〈17c〉 $\left\{A^{\left(1,2\right)}\}=\{B^{\left(1\right)}\right\}$ ⇔ r(A)=min⁡{m,n} *and* A=B*.*
〈18a〉 $\left\{A^{\left(1\right)}\}\subseteq \{B^{\left(1\right)}\right\}$ ⇔ r(A−B)=r(A)−r(B)*.*
〈18b〉 $\left\{A^{\left(1\right)}\}⊇\{B^{\left(1\right)}\right\}$ ⇔ r(B−A)=r(B)−r(A)*.*
〈18c〉 $\left\{A^{\left(1\right)}\}=\{B^{\left(1\right)}\right\}$ ⇔ A=B*.*

The results in Lemma 7.1, Lemma 7.2 can be simplified further for specified matrices *A* and *B*. For example, applying Lemma 7.1, Lemma 7.2 to two orthogonal projectors of the same size yields the following consequences.

Corollary 7.3 *Let* $A,\;B\in C^{m\times m}$ *be two orthogonal projectors. Then the following 23 results hold.*

〈1〉 $A\in \{B^{\left(1,3,4\right)}\}$ ⇔ R(A)⊇R(B)*.*
〈2〉 $A\in \{B^{\left(1,2,4\right)}\}$ ⇔ A=B*.*
〈3〉 $A\in \{B^{\left(1,4\right)}\}$ ⇔ R(A)⊇R(B)*.*
〈4〉 $A\in \{B^{\left(1,2\right)}\}$ ⇔ r(A)=r(B) *and* A=ABA*.*
〈5〉 $A\in \{B^{\left(1\right)}\}$ ⇔ r(A−ABA)=r(A)−r(B)*.*
〈6〉 $\{A^{\left(1,3,4\right)}\}\cap \{B^{\left(1,3,4\right)}\}\neq \emptyset$ *holds.*
〈7〉 $\{A^{\left(1,3,4\right)}\}\cap \{B^{\left(1,2,4\right)}\}\neq \emptyset$ ⇔ R(A)⊆R(B)*.*
〈8〉 $\{A^{\left(1,3,4\right)}\}\cap \{B^{\left(1,4\right)}\}\neq \emptyset$ *holds.*
〈9〉 $\{A^{\left(1,3,4\right)}\}\cap \{B^{\left(1,2\right)}\}\neq \emptyset$ ⇔ A=ABA*.*
〈10〉 $\{A^{\left(1,3,4\right)}\}\cap \{B^{\left(1\right)}\}\neq \emptyset$ *holds.*
〈11〉 $\{A^{\left(1,2,4\right)}\}\cap \{B^{\left(1,2,4\right)}\}\neq \emptyset$ ⇔ A=B*.*
〈12〉 $\{A^{\left(1,2,4\right)}\}\cap \{B^{\left(1,2,3\right)}\}\neq \emptyset$ ⇔ r(AB)=r(BA)=r(A)=r(B)*.*
〈13〉 $\{A^{\left(1,2,4\right)}\}\cap \{B^{\left(1,4\right)}\}\neq \emptyset$ ⇔ R(A)⊇R(B)*.*
〈14〉 $\{A^{\left(1,2,4\right)}\}\cap \{B^{\left(1,3\right)}\}\neq \emptyset$ ⇔ R(BA)=R(B)*.*
〈15〉 $\{A^{\left(1,2,4\right)}\}\cap \{B^{\left(1,2\right)}\}\neq \emptyset$ ⇔ R(AB)=R(A) *and* R(BA)=R(B)*.*
〈16〉 $\{A^{\left(1,2,4\right)}\}\cap \{B^{\left(1\right)}\}\neq \emptyset$ ⇔ R(BA)=R(B)*.*
〈17〉 $\{A^{\left(1,4\right)}\}\cap \{B^{\left(1,4\right)}\}\neq \emptyset$ *holds.*
〈18〉 $\{A^{\left(1,4\right)}\}\cap \{B^{\left(1,3\right)}\}\neq \emptyset$ *holds.*
〈19〉 $\{A^{\left(1,4\right)}\}\cap \{B^{\left(1,2\right)}\}\neq \emptyset$ ⇔ R(AB)=R(A)*.*
〈20〉 $\{A^{\left(1,4\right)}\}\cap \{B^{\left(1\right)}\}\neq \emptyset$ *holds.*
〈21〉 $\{A^{\left(1,2\right)}\}\cap \{B^{\left(1,2\right)}\}\neq \emptyset$ ⇔ r(A−B)=2r[A,B]−2r(A) *and* r(A)=r(B)*.*
〈22〉 $\{A^{\left(1,2\right)}\}\cap \{B^{\left(1\right)}\}\neq \emptyset$ ⇔ r(A−B)=2r[A,B]−r(A)−r(B) *and* r(A)≥r(B)*.*
〈23〉 $\{A^{\left(1\right)}\}\cap \{B^{\left(1\right)}\}\neq \emptyset$ ⇔ r(A−B)=2r[A,B]−r(A)−r(B)*.*

Corollary 7.4 *Let* $A,\;B\in C^{m\times m}$ *be two orthogonal projectors. Then the following results hold.*

〈1a〉 $\left\{A^{\left(1,3,4\right)}\}\subseteq \{B^{\left(1,3,4\right)}\right\}$ ⇔ R(A)⊇R(B)*.*
〈1b〉 $\left\{A^{\left(1,3,4\right)}\}⊇\{B^{\left(1,3,4\right)}\right\}$ ⇔ R(A)⊆R(B)*.*
〈1c〉 $\left\{A^{\left(1,3,4\right)}\}=\{B^{\left(1,3,4\right)}\right\}$ ⇔ A=B*.*
〈2a〉 $\left\{A^{\left(1,3,4\right)}\}\subseteq \{B^{\left(1,2,4\right)}\right\}$ ⇔ $A=B=I_{m}$*.*
〈2b〉 $\left\{A^{\left(1,3,4\right)}\}⊇\{B^{\left(1,2,4\right)}\right\}$ ⇔ A=0 *or* $B=I_{m}$*.*
〈2c〉 $\left\{A^{\left(1,3,4\right)}\}=\{B^{\left(1,2,4\right)}\right\}$ ⇔ $A=B=I_{m}$*.*
〈3a〉 $\left\{A^{\left(1,3,4\right)}\}\subseteq \{B^{\left(1,4\right)}\right\}$ ⇔ R(A)⊇R(B)*.*
〈3b〉 $\left\{A^{\left(1,3,4\right)}\}⊇\{B^{\left(1,4\right)}\right\}$ ⇔ A=0 *or* $B=I_{m}$*.*
〈3c〉 $\left\{A^{\left(1,3,4\right)}\}=\{B^{\left(1,4\right)}\right\}$ ⇔ A=B=0 *or* $A=B=I_{m}$*.*
〈4a〉 $\left\{A^{\left(1,3,4\right)}\}⊇\{B^{\left(1,2\right)}\right\}$ ⇔ A=0 *or* $B=I_{m}$*.*
〈4b〉 $\left\{A^{\left(1,3,4\right)}\}\subseteq \{B^{\left(1,2\right)}\right\}$ ⇔ $\left\{A^{\left(1,3,4\right)}\}=\{B^{\left(1,2\right)}\right\}$ ⇔ $A=B=I_{m}$*.*
〈5a〉 $\left\{A^{\left(1,3,4\right)}\}\subseteq \{B^{\left(1\right)}\right\}$ ⇔ R(A)⊇R(B)*.*
〈5b〉 $\left\{A^{\left(1,3,4\right)}\}⊇\{B^{\left(1\right)}\right\}$ ⇔ A=0 *or* $B=I_{m}$*.*
〈5c〉 $\left\{A^{\left(1,3,4\right)}\}=\{B^{\left(1\right)}\right\}$ ⇔ A=B=0 *or* $A=B=I_{m}$*.*
〈6〉 $\left\{A^{\left(1,2,4\right)}\}\subseteq \{B^{\left(1,2,4\right)}\right\}$ ⇔ $\left\{A^{\left(1,2,4\right)}\}⊇\{B^{\left(1,2,4\right)}\right\}$ ⇔ $\left\{A^{\left(1,2,4\right)}\}=\{B^{\left(1,2,4\right)}\right\}$ ⇔ A=B*.*
〈7〉 $\left\{A^{\left(1,2,4\right)}\}\subseteq \{B^{\left(1,2,3\right)}\right\}$ ⇔ $\left\{A^{\left(1,2,4\right)}\}⊇\{B^{\left(1,2,3\right)}\right\}$ ⇔ $\left\{A^{\left(1,2,4\right)}\}=\{B^{\left(1,2,3\right)}\right\}$ ⇔ A=B=0 *or* $A=B=I_{m}$*.*
〈8a〉 $\left\{A^{\left(1,2,4\right)}\}\subseteq \{B^{\left(1,4\right)}\right\}$ ⇔ R(A)⊇R(B)*.*
〈8b〉 $\left\{A^{\left(1,2,4\right)}\}⊇\{B^{\left(1,4\right)}\right\}$ ⇔ $\left\{A^{\left(1,2,4\right)}\}=\{B^{\left(1,4\right)}\right\}$ ⇔ $A=B=I_{m}$*.*
〈9a〉 $\left\{A^{\left(1,2,4\right)}\}\subseteq \{B^{\left(1,3\right)}\right\}$ ⇔ B=0 *or* $A=I_{m}$*.*
〈9b〉 $\left\{A^{\left(1,2,4\right)}\}⊇\{B^{\left(1,3\right)}\right\}$ ⇔ $\left\{A^{\left(1,2,4\right)}\}=\{B^{\left(1,3\right)}\right\}$ ⇔ $A=B=I_{m}$*.*
〈10a〉 $\left\{A^{\left(1,2,4\right)}\}\subseteq \{B^{\left(1,2\right)}\right\}$ ⇔ A=B*.*
〈10b〉 $\left\{A^{\left(1,2,4\right)}\}⊇\{B^{\left(1,2\right)}\right\}$ ⇔ $\left\{A^{\left(1,2,4\right)}\}=\{B^{\left(1,2\right)}\right\}$ ⇔ A=B=0 *or* $A=B=I_{m}$*.*
〈11a〉 $\left\{A^{\left(1,2,4\right)}\}\subseteq \{B^{\left(1\right)}\right\}$ ⇔ R(A)⊇R(B)*.*
〈11b〉 $\left\{A^{\left(1,2,4\right)}\}⊇\{B^{\left(1\right)}\right\}$ ⇔ $\left\{A^{\left(1,2,4\right)}\}=\{B^{\left(1\right)}\right\}$ ⇔ $A=B=I_{m}$*.*
〈12a〉 $\left\{A^{\left(1,4\right)}\}\subseteq \{B^{\left(1,4\right)}\right\}$ ⇔ R(A)⊇R(B)*.*
〈12b〉 $\left\{A^{\left(1,4\right)}\}⊇\{B^{\left(1,4\right)}\right\}$ ⇔ R(A)⊆R(B)*.*
〈12c〉 $\left\{A^{\left(1,4\right)}\}=\{B^{\left(1,4\right)}\right\}$ ⇔ A=B*.*
〈13a〉 $\left\{A^{\left(1,4\right)}\}\subseteq \{B^{\left(1,3\right)}\right\}$ ⇔ B=0 *or* $A=I_{m}$*.*
〈13b〉 $\left\{A^{\left(1,4\right)}\}⊇\{B^{\left(1,3\right)}\right\}$ ⇔ A=0 *or* $B=I_{m}$*.*
〈13c〉 $\left\{A^{\left(1,4\right)}\}=\{B^{\left(1,3\right)}\right\}$ ⇔ A=B=0 *or* $A=B=I_{m}$*.*
〈14a〉 $\left\{A^{\left(1,4\right)}\}⊇\{B^{\left(1,2\right)}\right\}$ ⇔ A=0 *or* $B=I_{m}$*.*
〈14b〉 $\left\{A^{\left(1,4\right)}\}\subseteq \{B^{\left(1,2\right)}\right\}$ ⇔ $\left\{A^{\left(1,4\right)}\}=\{B^{\left(1,2\right)}\right\}$ ⇔ $A=B=I_{m}$*.*
〈15a〉 $\left\{A^{\left(1,4\right)}\}\subseteq \{B^{\left(1\right)}\right\}$ ⇔ R(A)⊇R(B)*.*
〈15b〉 $\left\{A^{\left(1,4\right)}\}⊇\{B^{\left(1\right)}\right\}$ ⇔ A=0 *or* $B=I_{m}$*.*
〈15c〉 $\left\{A^{\left(1,4\right)}\}=\{B^{\left(1\right)}\right\}$ ⇔ A=B=0 *or* $A=B=I_{m}$*.*
〈16〉 $\left\{A^{\left(1,2\right)}\}\subseteq \{B^{\left(1,2\right)}\right\}$ ⇔ $\left\{A^{\left(1,2\right)}\}⊇\{B^{\left(1,2\right)}\right\}$ ⇔ $\left\{A^{\left(1,2\right)}\}=\{B^{\left(1,2\right)}\right\}$ ⇔ A=B*.*
〈17a〉 $\left\{A^{\left(1,2\right)}\}\subseteq \{B^{\left(1\right)}\right\}$ ⇔ r(A−B)=r(A)−r(B)*.*
〈17b〉 $\left\{A^{\left(1,2\right)}\}⊇\{B^{\left(1\right)}\right\}$ ⇔ $\left\{A^{\left(1,2\right)}\}=\{B^{\left(1\right)}\right\}$ ⇔ $A=B=I_{m}$*.*
〈18a〉 $\left\{A^{\left(1\right)}\}\subseteq \{B^{\left(1\right)}\right\}$ ⇔ r(A−B)=r(A)−r(B)*.*
〈18b〉 $\left\{A^{\left(1\right)}\}⊇\{B^{\left(1\right)}\right\}$ ⇔ r(B−A)=r(B)−r(A)*.*
〈18c〉 $\left\{A^{\left(1\right)}\}=\{B^{\left(1\right)}\right\}$ ⇔ A=B*.*

Corollary 7.5 *Let* $A\in C^{m\times n}$ *be given. Then the following results hold.*

〈1a〉 $\left\{A^{\left(1,3,4\right)}\}\subseteq \{A^{\left(1,2,4\right)}\right\}$ ⇔ r(A)=m *or* r(A)=n*.*
〈1b〉 $\left\{A^{\left(1,3,4\right)}\}⊇\{A^{\left(1,2,4\right)}\right\}$ ⇔ A=0 *or* r(A)=m*.*
〈1c〉 $\left\{A^{\left(1,3,4\right)}\}=\{A^{\left(1,2,4\right)}\right\}$ ⇔ r(A)=m*.*
〈2a〉 $\left\{A^{\left(1,3,4\right)}\}\subseteq \{A^{\left(1,2,3\right)}\right\}$ ⇔ r(A)=m *or* r(A)=n*.*
〈2b〉 $\left\{A^{\left(1,3,4\right)}\}⊇\{A^{\left(1,2,3\right)}\right\}$ ⇔ A=0 *or* r(A)=n*.*
〈2c〉 $\left\{A^{\left(1,3,4\right)}\}=\{A^{\left(1,2,3\right)}\right\}$ ⇔ r(A)=n*.*
〈3〉 $\left\{A^{\left(1,3,4\right)}\}⊇\{A^{\left(1,4\right)}\right\}$ ⇔ $\left\{A^{\left(1,3,4\right)}\}=\{A^{\left(1,4\right)}\right\}$ ⇔ A=0 *or* r(A)=m*.*
〈4〉 $\left\{A^{\left(1,3,4\right)}\}⊇\{A^{\left(1,3\right)}\right\}$ ⇔ $\left\{A^{\left(1,3,4\right)}\}=\{A^{\left(1,3\right)}\right\}$ ⇔ A=0 *or* r(A)=n*.*
〈5a〉 $\left\{A^{\left(1,3,4\right)}\}\subseteq \{A^{\left(1,2\right)}\right\}$ ⇔ r(A)=m *or* r(A)=n*.*
〈5b〉 $\left\{A^{\left(1,3,4\right)}\}⊇\{A^{\left(1,2\right)}\right\}$ ⇔ A=0 *or* r(A)=m=n*.*
〈5c〉 $\left\{A^{\left(1,3,4\right)}\}=\{A^{\left(1,2\right)}\right\}$ ⇔ r(A)=m=n*.*
〈6a〉 $\left\{A^{\left(1,3,4\right)}\}⊇\{A^{\left(1\right)}\right\}$ ⇔ A=0 *or* r(A)=m=n*.*
〈6b〉 $\left\{A^{\left(1,3,4\right)}\}=\{A^{\left(1\right)}\right\}$ ⇔ A=0 *or* r(A)=m=n*.*
〈7a〉 $\left\{A^{\left(1,2,4\right)}\}\subseteq \{A^{\left(1,2,3\right)}\right\}$ ⇔ A=0 *or* r(A)=m*.*
〈7b〉 $\left\{A^{\left(1,2,4\right)}\}⊇\{A^{\left(1,2,3\right)}\right\}$ ⇔ A=0 *or* r(A)=n*.*
〈7c〉 $\left\{A^{\left(1,2,4\right)}\}=\{A^{\left(1,2,3\right)}\right\}$ ⇔ A=0 *or* r(A)=m=n*.*
〈8〉 $\left\{A^{\left(1,2,4\right)}\}⊇\{A^{\left(1,4\right)}\right\}$ ⇔ $\left\{A^{\left(1,2,4\right)}\}=\{A^{\left(1,4\right)}\right\}$ ⇔ r(A)=m *or* r(A)=n*.*
〈9a〉 $\left\{A^{\left(1,2,4\right)}\}\subseteq \{A^{\left(1,3\right)}\right\}$ ⇔ A=0 *or* r(A)=m*.*
〈9b〉 $\left\{A^{\left(1,2,4\right)}\}⊇\{A^{\left(1,3\right)}\right\}$ ⇔ r(A)=n*.*
〈9c〉 $\left\{A^{\left(1,2,4\right)}\}=\{A^{\left(1,3\right)}\right\}$ ⇔ r(A)=m=n*.*
〈10〉 $\left\{A^{\left(1,2,4\right)}\}⊇\{A^{\left(1,2\right)}\right\}$ ⇔ $\left\{A^{\left(1,2,4\right)}\}=\{A^{\left(1,2\right)}\right\}$ ⇔ A=0 *or* r(A)=n*.*
〈11〉 $\left\{A^{\left(1,2,4\right)}\}⊇\{A^{\left(1\right)}\right\}$ ⇔ $\left\{A^{\left(1,2,4\right)}\}=\{A^{\left(1\right)}\right\}$ ⇔ r(A)=n*.*
〈12a〉 $\left\{A^{\left(1,2,3\right)}\}\subseteq \{A^{\left(1,4\right)}\right\}$ ⇔ A=0 *or* r(A)=n*.*
〈12b〉 $\left\{A^{\left(1,2,3\right)}\}⊇\{A^{\left(1,4\right)}\right\}$ ⇔ r(A)=m*.*
〈12c〉 $\left\{A^{\left(1,2,3\right)}\}=\{A^{\left(1,4\right)}\right\}$ ⇔ r(A)=m=n*.*
〈13〉 $\left\{A^{\left(1,2,3\right)}\}⊇\{A^{\left(1,3\right)}\right\}$ ⇔ $\left\{A^{\left(1,2,3\right)}\}=\{A^{\left(1,3\right)}\right\}$ ⇔ r(A)=m *or* r(A)=n*.*
〈14〉 $\left\{A^{\left(1,2,3\right)}\}⊇\{A^{\left(1,2\right)}\right\}$ ⇔ $\left\{A^{\left(1,2,3\right)}\}=\{A^{\left(1,2\right)}\right\}$ ⇔ A=0 *or* r(A)=m*.*
〈15〉 $\left\{A^{\left(1,2,3\right)}\}⊇\{A^{\left(1\right)}\right\}$ ⇔ $\left\{A^{\left(1,2,3\right)}\}=\{A^{\left(1\right)}\right\}$ ⇔ r(A)=m*.*
〈16a〉 $\left\{A^{\left(1,4\right)}\}\subseteq \{A^{\left(1,3\right)}\right\}$ ⇔ A=0 *or* r(A)=m*.*
〈16b〉 $\left\{A^{\left(1,4\right)}\}⊇\{A^{\left(1,3\right)}\right\}$ ⇔ A=0 *or* r(A)=n*.*
〈16c〉 $\left\{A^{\left(1,4\right)}\}=\{A^{\left(1,3\right)}\right\}$ ⇔ A=0 *or* r(A)=m=n*.*
〈17a〉 $\left\{A^{\left(1,4\right)}\}\subseteq \{A^{\left(1,2\right)}\right\}$ ⇔ r(A)=m *or* r(A)=n*.*
〈17b〉 $\left\{A^{\left(1,4\right)}\}⊇\{A^{\left(1,2\right)}\right\}$ ⇔ A=0 *or* r(A)=n*.*
〈17c〉 $\left\{A^{\left(1,4\right)}\}=\{A^{\left(1,2\right)}\right\}$ ⇔ r(A)=n*.*
〈18〉 $\left\{A^{\left(1,4\right)}\}⊇\{A^{\left(1\right)}\right\}$ ⇔ $\left\{A^{\left(1,4\right)}\}=\{A^{\left(1\right)}\right\}$ ⇔ A=0 *or* r(A)=n*.*
〈19a〉 $\left\{A^{\left(1,3\right)}\}\subseteq \{A^{\left(1,2\right)}\right\}$ ⇔ r(A)=m *or* r(A)=n*.*
〈19b〉 $\left\{A^{\left(1,3\right)}\}⊇\{A^{\left(1,2\right)}\right\}$ ⇔ A=0 *or* r(A)=m*.*
〈19c〉 $\left\{A^{\left(1,3\right)}\}=\{A^{\left(1,2\right)}\right\}$ ⇔ r(A)=m*.*
〈20〉 $\left\{A^{\left(1,3\right)}\}⊇\{A^{\left(1\right)}\right\}$ ⇔ $\left\{A^{\left(1,3\right)}\}=\{A^{\left(1\right)}\right\}$ ⇔ A=0 *or* r(A)=m*.*
〈21〉 $\left\{A^{\left(1,2\right)}\}⊇\{A^{\left(1\right)}\right\}$ ⇔ $\left\{A^{\left(1,2\right)}\}=\{A^{\left(1\right)}\right\}$ ⇔ r(A)=m *or* r(A)=n*.*

The preceding results show that much work is involved in the establishments of the simplest matrix equalities for two generalized inverses. Hence the approaches on general matrix equalities for generalized inverses will become quite complicated for operations of more matrices and generalized inverses.

## 8 Ranks of matrix expressions composed of two generalized inverses

In order to characterize the set inclusions in (1.10), we first need to know some fundamental properties of the products of generalized inverses. In this section, we present a variety of known results concerning the ranks and uniqueness of the product $B^{\left(s_{2},\ldots ,t_{2}\right)}A^{\left(s_{1},\ldots ,t_{1}\right)}$ and their variations, which will be used to characterize (1.8) under various assumptions.

It is obvious that (1.8) includes a large variety of situations with symmetric pattern. Hence the following lemma can help us characterize many set inclusion problems by symmetry.

Lemma 8.1 *Let* $A\in C^{m\times n}$ *and* $B\in C^{n\times p}$ *be given. Then the following 64 matrix set identities*

(8.1) $$\left\{{\left(B^{\left(s_{2},\ldots ,t_{2}\right)}A^{\left(s_{1},\ldots ,t_{1}\right)}\right)}^{⁎}\right\}=\left\{{\left(A^{⁎}\right)}^{\left(i_{1},\ldots ,j_{1}\right)}{\left(B^{⁎}\right)}^{\left(i_{2},\ldots ,j_{2}\right)}\right\}$$

*hold according to Lemma* 3.1(c) *for the eight commonly-used types of generalized inverses of A and B.*

Lemma 8.2 **[79]**

*Let* $A\in C^{m\times n}$ *and* $B\in C^{n\times p}$ *be given. Then the product* $B^{†}A^{†}$ *can be written as*

(8.2) $$B^{†}A^{†}=-[B^{⁎},\;0]{\left[\begin{array}{cc} 0 & A^{⁎}AA^{⁎}\\ B^{⁎}BB^{⁎}\; & \;B^{⁎}A^{⁎} \end{array}\right]}^{†}\left[\begin{array}{c} A^{⁎}\\ 0 \end{array}\right]\overset{△}{=}-PJ^{†}Q,$$

*where the block matrices P, J, and Q satisfy* r(J)=r(A)+r(B)*,* R(Q)⊆R(J)*, and* $R(P^{⁎})\subseteq R(J^{⁎})$*.*

Note that the rank of the product $B^{\left(s_{2},\ldots ,t_{2}\right)}A^{\left(s_{1},\ldots ,t_{1}\right)}$ may vary with respect to the choice of $A^{\left(s_{1},\ldots ,t_{1}\right)}$ and $B^{\left(s_{2},\ldots ,t_{2}\right)}$ (the variable matrices in the analytical expressions of $A^{\left(s_{1},\ldots ,t_{1}\right)}$ and $B^{\left(s_{2},\ldots ,t_{2}\right)}$). Also note from Lemma 3.2 that the ranks of the 64 products $B^{\left(s_{2},\ldots ,t_{2}\right)}A^{\left(s_{1},\ldots ,t_{1}\right)}$ are involved in the set inclusions for the {1,2}-, {1,2,3}-, and {1,2,4}-generalized inverses of *AB*. Thus it is imperative to determine the maximum and minimum ranks of $B^{\left(s_{2},\ldots ,t_{2}\right)}A^{\left(s_{1},\ldots ,t_{1}\right)}$ with respect to the choice of the generalized inverses. In the past several decades, a great achievement in linear algebra is the sufficient development of the matrix rank theory. Thousands of analytical formulas for calculating (maximum and minimum) ranks of matrix expressions have been established, and numerous consequences and applications of these matrix rank formulas have been obtained. In recent two papers [93], [94], the present author provided a comprehensive study of the rank problems of matrix expressions composed of a pair of matrices and their generalized inverses, including the following analytical formulas for calculating the maximum and minimum ranks of $B^{\left(s_{2},\ldots ,t_{2}\right)}A^{\left(s_{1},\ldots ,t_{1}\right)}$ with respect to the choice of $A^{\left(s_{1},\ldots ,t_{1}\right)}$ and $B^{\left(s_{2},\ldots ,t_{2}\right)}$.

Lemma 8.3 **[93]**

*Let* $A\in C^{m\times n}$ *and* $B\in C^{n\times p}$ *be given, and denote*

$$M=AB,\;\;N=[A^{⁎},\;B],\;\;t=m+p+r(M)-r(A)-r(B),\;\;t_{1}=m+r(M)-r(A),\;\;t_{2}=p+r(M)-r(B).$$

*Also use* $u(B^{\left(s_{2},\ldots ,t_{2}\right)}A^{\left(s_{1},\ldots ,t_{1}\right)})$ *and* $v(B^{\left(s_{2},\ldots ,t_{2}\right)}A^{\left(s_{1},\ldots ,t_{1}\right)})$ *to denote the maximum and minimum ranks of the product* $B^{\left(s_{2},\ldots ,t_{2}\right)}A^{\left(s_{1},\ldots ,t_{1}\right)}$ *with respect to the choice of* $A^{\left(s_{1},\ldots ,t_{1}\right)}$ *and* $B^{\left(s_{2},\ldots ,t_{2}\right)}$*, respectively. Then*

$$u(B^{†}A^{\left(1,3,4\right)})=\min\{r(B),\;t_{1}\},\;v(B^{†}A^{\left(1,3,4\right)})=r(M),u(B^{†}A^{\left(1,2,4\right)})=r(M),\;v(B^{†}A^{\left(1,2,4\right)})=r(M),u(B^{†}A^{\left(1,2,3\right)})=\min\{r(A),\;r(B)\},\;v(B^{†}A^{\left(1,2,3\right)})=r(A)+r(B)-r(N),u(B^{†}A^{\left(1,4\right)})=\min\{r(B),\;t_{1}\},\;v(B^{†}A^{\left(1,4\right)})=r(M),u(B^{†}A^{\left(1,3\right)})=\min\{m,\;r(B)\},\;v(B^{†}A^{\left(1,3\right)})=r(A)+r(B)-r(N),u(B^{†}A^{\left(1,2\right)})=\min\{r(A),\;r(B)\},\;v(B^{†}A^{\left(1,2\right)})=r(A)+r(B)-r(N),u(B^{†}A^{\left(1\right)})=\min\{m,\;r(B)\},\;v(B^{†}A^{\left(1\right)})=r(A)+r(B)-r(N),u(B^{\left(1,3,4\right)}A^{†})=\min\{r(A),\;t_{2}\},\;v(B^{\left(1,3,4\right)}A^{†})=r(M),u(B^{\left(1,3,4\right)}A^{\left(1,3,4\right)})=\min\{m,\;n,\;p,\;t\},\;v(B^{\left(1,3,4\right)}A^{\left(1,3,4\right)})=r(M),u(B^{\left(1,3,4\right)}A^{\left(1,2,4\right)})=\min\{r(A),\;t_{2}\},\;v(B^{\left(1,3,4\right)}A^{\left(1,2,4\right)})=r(M),u(B^{\left(1,3,4\right)}A^{\left(1,2,3\right)})=\min\{p,\;r(A)\},\;v(B^{\left(1,3,4\right)}A^{\left(1,2,3\right)})=r(A)+r(B)-r(N),u(B^{\left(1,3,4\right)}A^{\left(1,4\right)})=\min\{m,\;n,\;p,\;t\},\;v(B^{\left(1,3,4\right)}A^{\left(1,4\right)})=r(M),u(B^{\left(1,3,4\right)}A^{\left(1,3\right)})=\min\{m,\;n,\;p\},\;v(B^{\left(1,3,4\right)}A^{\left(1,3\right)})=r(A)+r(B)-r(N),u(B^{\left(1,3,4\right)}A^{\left(1,2\right)})=\min\{p,\;r(A)\},\;v(B^{\left(1,3,4\right)}A^{\left(1,2\right)})=r(A)+r(B)-r(N),u(B^{\left(1,3,4\right)}A^{\left(1\right)})=\min\{m,\;n,\;p\},\;v(B^{\left(1,3,4\right)}A^{\left(1\right)})=r(A)+r(B)-r(N),u(B^{\left(1,2,4\right)}A^{†})=\min\{r(A),\;r(B)\},\;v(B^{\left(1,2,4\right)}A^{†})=r(A)+r(B)-r(N),u(B^{\left(1,2,4\right)}A^{\left(1,3,4\right)})=\min\{m,\;r(B)\},\;v(B^{\left(1,2,4\right)}A^{\left(1,3,4\right)})=r(A)+r(B)-r(N),u(B^{\left(1,2,4\right)}A^{\left(1,2,4\right)})=\min\{r(A),\;r(B)\},\;v(B^{\left(1,2,4\right)}A^{\left(1,2,4\right)})=r(A)+r(B)-r(N),u(B^{\left(1,2,4\right)}A^{\left(1,2,3\right)})=\min\{r(A),\;r(B)\},\;v(B^{\left(1,2,4\right)}A^{\left(1,2,3\right)})=\max\{0,\;r(A)+r(B)-n\},u(B^{\left(1,2,4\right)}A^{\left(1,4\right)})=\min\{m,\;r(B)\},\;v(B^{\left(1,2,4\right)}A^{\left(1,4\right)})=r(A)+r(B)-r(N),u(B^{\left(1,2,4\right)}A^{\left(1,3\right)})=\min\{m,\;r(B)\},\;v(B^{\left(1,2,4\right)}A^{\left(1,3\right)})=\max\{0,\;r(A)+r(B)-n\},u(B^{\left(1,2,4\right)}A^{\left(1,2\right)})=\min\{r(A),\;r(B)\},\;v(B^{\left(1,2,4\right)}A^{\left(1,2\right)})=\max\{0,\;r(A)+r(B)-n\},u(B^{\left(1,2,4\right)}A^{\left(1\right)})=\min\{m,\;r(B)\},\;v(B^{\left(1,2,4\right)}A^{\left(1\right)})=\max\{0,\;r(A)+r(B)-n\},$$

$$u(B^{\left(1,2,3\right)}A^{†})=r(M),\;v(B^{\left(1,2,3\right)}A^{†})=r(M),\;\;u(B^{\left(1,2,3\right)}A^{\left(1,3,4\right)})=\min\{r(B),\;t_{1}\},\;v(B^{\left(1,2,3\right)}A^{\left(1,3,4\right)})=r(M),\;\;u(B^{\left(1,2,3\right)}A^{\left(1,2,4\right)})=r(M),\;v(B^{\left(1,2,3\right)}A^{\left(1,2,4\right)})=r(M),\;\;u(B^{\left(1,2,3\right)}A^{\left(1,2,3\right)})=\min\{r(A),\;r(B)\},\;v(B^{\left(1,2,3\right)}A^{\left(1,2,3\right)})=r(A)+r(B)-r(N),\;\;u(B^{\left(1,2,3\right)}A^{\left(1,4\right)})=\min\{r(B),\;t_{1}\},\;v(B^{\left(1,2,3\right)}A^{\left(1,4\right)})=r(M),\;\;u(B^{\left(1,2,3\right)}A^{\left(1,3\right)})=\min\{m,\;r(B)\},\;v(B^{\left(1,2,3\right)}A^{\left(1,3\right)})=r(A)+r(B)-r(N),\;\;u(B^{\left(1,2,3\right)}A^{\left(1,2\right)})=\min\{r(A),\;r(B)\},\;v(B^{\left(1,2,3\right)}A^{\left(1,2\right)})=r(A)+r(B)-r(N),\;\;u(B^{\left(1,2,3\right)}A^{\left(1\right)})=\min\{m,\;r(B)\},\;v(B^{\left(1,2,3\right)}A^{\left(1\right)})=r(A)+r(B)-r(N),\;\;u(B^{\left(1,4\right)}A^{†})=\min\{p,\;r(A)\},\;v(B^{\left(1,4\right)}A^{†})=r(A)+r(B)-r(N),\;\;u(B^{\left(1,4\right)}A^{\left(1,3,4\right)})=\min\{m,\;n,\;p\},\;v(B^{\left(1,4\right)}A^{\left(1,3,4\right)})=r(A)+r(B)-r(N),\;\;u(B^{\left(1,4\right)}A^{\left(1,2,4\right)})=\min\{p,\;r(A)\},\;v(B^{\left(1,4\right)}A^{\left(1,2,4\right)})=r(A)+r(B)-r(N),\;\;u(B^{\left(1,4\right)}A^{\left(1,2,3\right)})=\min\{p,\;r(A)\},\;v(B^{\left(1,4\right)}A^{\left(1,2,3\right)})=\max\{0,\;r(A)+r(B)-n\},\;\;u(B^{\left(1,4\right)}A^{\left(1,4\right)})=\min\{m,\;n,\;p\},\;v(B^{\left(1,4\right)}A^{\left(1,4\right)})=r(A)+r(B)-r(N),\;\;u(B^{\left(1,4\right)}A^{\left(1,3\right)})=\min\{m,\;n,\;p\},\;v(B^{\left(1,4\right)}A^{\left(1,3\right)})=\max\{0,\;r(A)+r(B)-n\},\;\;u(B^{\left(1,4\right)}A^{\left(1,2\right)})=\min\{p,\;r(A)\},\;v(B^{\left(1,4\right)}A^{\left(1,2\right)})=\max\{0,\;r(A)+r(B)-n\},\;\;u(B^{\left(1,4\right)}A^{\left(1\right)})=\min\{m,\;n,\;p\},\;v(B^{\left(1,4\right)}A^{\left(1\right)})=\max\{0,\;r(A)+r(B)-n\},\;\;u(B^{\left(1,3\right)}A^{†})=\min\{r(A),\;t_{2}\},\;v(B^{\left(1,3\right)}A^{†})=r(M),\;\;u(B^{\left(1,3\right)}A^{\left(1,3,4\right)})=\min\{m,\;n,\;p,\;t\},\;v(B^{\left(1,3\right)}A^{\left(1,3,4\right)})=r(M),\;\;u(B^{\left(1,3\right)}A^{\left(1,2,4\right)})=\min\{r(A),\;t_{2}\},\;v(B^{\left(1,3\right)}A^{\left(1,2,4\right)})=r(M),\;\;u(B^{\left(1,3\right)}A^{\left(1,2,3\right)})=\min\{p,\;r(A)\},\;v(B^{\left(1,3\right)}A^{\left(1,2,3\right)})=r(A)+r(B)-r(N),\;\;u(B^{\left(1,3\right)}A^{\left(1,4\right)})=\min\{m,\;n,\;p,\;t\},\;v(B^{\left(1,3\right)}A^{\left(1,4\right)})=r(M),\;\;u(B^{\left(1,3\right)}A^{\left(1,3\right)})=\min\{m,\;n,\;p\},\;v(B^{\left(1,3\right)}A^{\left(1,3\right)})=r(A)+r(B)-r(N),\;\;u(B^{\left(1,3\right)}A^{\left(1,2\right)})=\min\{p,\;r(A)\},\;v(B^{\left(1,3\right)}A^{\left(1,2\right)})=r(A)+r(B)-r(N),\;\;u(B^{\left(1,3\right)}A^{\left(1\right)})=\min\{m,\;n,\;p\},\;v(B^{\left(1,3\right)}A^{\left(1\right)})=r(A)+r(B)-r(N),\;\;u(B^{\left(1,2\right)}A^{†})=\min\{r(A),\;r(B)\},\;v(B^{\left(1,2\right)}A^{†})=r(A)+r(B)-r(N),\;\;u(B^{\left(1,2\right)}A^{\left(1,3,4\right)})=\min\{m,\;r(B)\},\;v(B^{\left(1,2\right)}A^{\left(1,3,4\right)})=r(A)+r(B)-r(N),\;\;u(B^{\left(1,2\right)}A^{\left(1,2,4\right)})=\min\{r(A),\;r(B)\},\;v(B^{\left(1,2\right)}A^{\left(1,2,4\right)})=r(A)+r(B)-r(N),\;\;u(B^{\left(1,2\right)}A^{\left(1,2,3\right)})=\min\{r(A),\;r(B)\},\;v(B^{\left(1,2\right)}A^{\left(1,2,3\right)})=\max\{0,\;r(A)+r(B)-n\},\;\;u(B^{\left(1,2\right)}A^{\left(1,4\right)})=\min\{m,\;r(B)\},\;v(B^{\left(1,2\right)}A^{\left(1,4\right)})=r(A)+r(B)-r(N),\;\;u(B^{\left(1,2\right)}A^{\left(1,3\right)})=\min\{m,\;r(B)\},\;v(B^{\left(1,2\right)}A^{\left(1,3\right)})=\max\{0,\;r(A)+r(B)-n\},\;\;u(B^{\left(1,2\right)}A^{\left(1,2\right)})=\min\{r(A),\;r(B)\},\;v(B^{\left(1,2\right)}A^{\left(1,2\right)})=\max\{0,\;r(A)+r(B)-n\},\;\;u(B^{\left(1,2\right)}A^{\left(1\right)})=\min\{m,\;r(B)\},\;v(B^{\left(1,2\right)}A^{\left(1\right)})=\max\{0,\;r(A)+r(B)-n\},\;\;u(B^{\left(1\right)}A^{†})=\min\{p,\;r(A)\},\;v(B^{\left(1\right)}A^{†})=r(A)+r(B)-r(N),\;\;u(B^{\left(1\right)}A^{\left(1,3,4\right)})=\min\{m,\;n,\;p\},\;v(B^{\left(1\right)}A^{\left(1,3,4\right)})=r(A)+r(B)-r(N),\;\;u(B^{\left(1\right)}A^{\left(1,2,4\right)})=\min\{p,\;r(A)\},\;v(B^{\left(1\right)}A^{\left(1,2,4\right)})=r(A)+r(B)-r(N),\;\;u(B^{\left(1\right)}A^{\left(1,2,3\right)})=\min\{p,\;r(A)\},\;v(B^{\left(1\right)}A^{\left(1,2,3\right)})=\max\{0,\;r(A)+r(B)-n\},\;\;u(B^{\left(1\right)}A^{\left(1,4\right)})=\min\{m,\;n,\;p\},\;v(B^{\left(1\right)}A^{\left(1,4\right)})=r(A)+r(B)-r(N),\;\;u(B^{\left(1\right)}A^{\left(1,3\right)})=\min\{m,\;n,\;p\},\;v(B^{\left(1\right)}A^{\left(1,3\right)})=\max\{0,\;r(A)+r(B)-n\},\;\;u(B^{\left(1\right)}A^{\left(1,2\right)})=\min\{p,\;r(A)\},\;v(B^{\left(1\right)}A^{\left(1,2\right)})=\max\{0,\;r(A)+r(B)-n\},\;\;u(B^{\left(1\right)}A^{\left(1\right)})=\min\{m,\;n,\;p\},\;v(B^{\left(1\right)}A^{\left(1\right)})=\max\{0,\;r(A)+r(B)-n\}.$$

Notice that the rank formulas in Lemma 8.3 are all given in simple and analytical forms. Thus, they can be utilized to describe algebraic performance of the products of generalized inverses in many situations, including the rank invariance of $B^{\left(s_{2},\ldots ,t_{2}\right)}A^{\left(s_{1},\ldots ,t_{1}\right)}$, which will be used in Theorem 9.3 below.

Lemma 8.4 **[94]**

*Let* $A\in C^{m\times n}$ *and* $B\in C^{n\times p}$ *be given, and denote* $N=[A^{⁎},\;B]$*. Then the following results hold.*

(a) *The following two rank equalities always hold for all the generalized inverses*
  $$r(BB^{†}A^{†}A)=r(BB^{\left(1,3\right)}A^{\left(1,4\right)}A)=r(AB).$$
(b) *The following 4 maximum rank formulas hold*
  $$u(BB^{†}A^{\left(1\right)}A)=u(BB^{\left(1\right)}A^{†}A)=u(BB^{\left(1\right)}A^{\left(1\right)}A)=\min\{r(A),\;\;r(B)\}.$$
(c) *The following 2 minimum rank formulas hold*
  $$v(BB^{†}A^{\left(1\right)}A)=v(BB^{\left(1\right)}A^{†}A)=r(A)+r(B)-r(N);$$
  *the following minimum rank formula holds*
  $$v(BB^{\left(1\right)}A^{\left(1\right)}A)=\max\{0,\;\;r(A)+r(B)-n\}.$$
(d) *The following 2 formulas hold for all the generalized inverses*
  $$r(I_{n}-BB^{†}A^{†}A)=r(I_{n}-BB^{\left(1,3\right)}A^{\left(1,4\right)}A)=r(N)-r(A)-r(B)+n.$$
(e) *The following 2 maximum rank formulas hold*
  $$u(I_{n}-BB^{†}A^{\left(1\right)}A)=u(I_{n}-BB^{\left(1\right)}A^{†}A)=r(N)-r(A)-r(B)+n;$$
  *the following maximum rank formula holds*
  $$u(I_{n}-BB^{\left(1\right)}A^{\left(1\right)}A)=\min\{n,\;\;2n-r(A)-r(B)\}.$$
(f) *The following 3 minimum rank formulas hold*
  $$v(I_{n}-BB^{†}A^{\left(1\right)}A)=v(I_{n}-BB^{\left(1\right)}A^{†}A)=v(I_{n}-BB^{\left(1\right)}A^{\left(1\right)}A)=n-r(AB).$$
(g) *The following rank equality holds*
  $$r(A^{\left(s_{1},\ldots ,t_{1}\right)}ABB^{\left(s_{2},\ldots ,t_{2}\right)})=r(AB),$$
  *for all the eight types of commonly-used* $A^{\left(s_{1},\ldots ,t_{1}\right)}$ *and* $B^{\left(s_{2},\ldots ,t_{2}\right)}$*.*
(h) *The following rank equality holds*
  $$r(I_{n}-A^{†}ABB^{†})=r(N)-r(A)-r(B)+n.$$
(i) *The following 2 maximum rank formulas hold*
  $$u(I_{n}-A^{\left(1\right)}ABB^{†})=u(I_{n}-A^{†}ABB^{\left(1\right)})=r(N)-r(A)-r(B)+n;$$
  *the following maximum rank formula holds*
  $$u(I_{n}-A^{\left(1\right)}ABB^{\left(1\right)})=\min\left\{n,\;\;2n-r(A)-r(B)\right\}.$$
(j) *The following 3 minimum rank formulas hold*
  $$v(I_{n}-A^{\left(1\right)}ABB^{†})=v(I_{n}-A^{†}ABB^{\left(1\right)})=v(I_{n}-A^{\left(1\right)}ABB^{\left(1\right)})=n-r(AB).$$
(k) *The following rank equality holds*
  $$u(ABB^{\left(s_{2},\ldots ,t_{2}\right)}A^{\left(s_{1},\ldots ,t_{1}\right)})=r(AB)$$
  *for all the eight commonly-used types of generalized inverses* $A^{\left(s_{1},\ldots ,t_{1}\right)}$ *and* $B^{\left(s_{2},\ldots ,t_{2}\right)}$*.*
(l) *The following 3 minimum rank formulas hold*
  $$v(ABB^{†}A^{\left(1,3,4\right)})=v(ABB^{†}A^{\left(1,2,4\right)})=v(ABB^{†}A^{\left(1,4\right)})=r(AB);$$
  *the following 8 minimum rank formulas hold*
  $$v(ABB^{\left(1\right)}A^{†})=v(ABB^{\left(1\right)}A^{\left(1,3,4\right)})=v(ABB^{\left(1\right)}A^{\left(1,2,4\right)})=v(ABB^{\left(1\right)}A^{\left(1,4\right)})=v(ABB^{†}A^{\left(1,2,3\right)})=v(ABB^{†}A^{\left(1,3\right)})=v(ABB^{†}A^{\left(1,2\right)})=v(ABB^{†}A^{\left(1\right)})=r(A)+r(B)-r(N);$$
  *the following 4 minimum rank formulas hold*
  $$v(ABB^{\left(1\right)}A^{\left(1,2,3\right)})=v(ABB^{\left(1\right)}A^{\left(1,3\right)})=v(ABB^{\left(1\right)}A^{\left(1,2\right)})=v(ABB^{\left(1\right)}A^{\left(1\right)})=\max\{0,\;r(A)+r(B)-n\}.$$
(m) *The following 4 rank equalities hold for all the generalized inverses*
  $$r(I_{m}-ABB^{†}A^{†})=r(I_{m}-ABB^{†}A^{\left(1,3,4\right)})=r(I_{m}-ABB^{†}A^{\left(1,2,4\right)})=r(I_{m}-ABB^{†}A^{\left(1,4\right)})=r(N)-r(A)-r(B)+m.$$
(n) *The following 8 maximum rank formulas hold*
  $$u(I_{m}-ABB^{†}A^{\left(1,2,3\right)})=u(I_{m}-ABB^{†}A^{\left(1,3\right)})=u(I_{m}-ABB^{†}A^{\left(1,2\right)})=u(I_{m}-ABB^{†}A^{\left(1\right)})=u(I_{m}-ABB^{\left(1\right)}A^{†})=u(I_{m}-ABB^{\left(1\right)}A^{\left(1,3,4\right)})=u(I_{m}-ABB^{\left(1\right)}A^{\left(1,2,4\right)})=u(I_{m}-ABB^{\left(1\right)}A^{\left(1,4\right)})=r(N)-r(A)-r(B)+m;$$
  *the following 4 maximum rank formulas hold*
  $$u(I_{m}-ABB^{\left(1\right)}A^{\left(1,2,3\right)})=u(I_{m}-ABB^{\left(1\right)}A^{\left(1,3\right)})=u(I_{m}-ABB^{\left(1\right)}A^{\left(1,2\right)})=u(I_{m}-ABB^{\left(1\right)}A^{\left(1\right)})=\min\{m,\;\;m+n-r(A)-r(B)\}.$$
(o) *The following 12 minimum rank formulas hold*
  $$v(I_{m}-ABB^{†}A^{\left(1,2,3\right)})=v(I_{m}-ABB^{†}A^{\left(1,3\right)})=v(I_{m}-ABB^{†}A^{\left(1,2\right)})=v(I_{m}-ABB^{†}A^{\left(1\right)})=v(I_{m}-ABB^{\left(1\right)}A^{†})=v(I_{m}-ABB^{\left(1\right)}A^{\left(1,3,4\right)})=v(I_{m}-ABB^{\left(1\right)}A^{\left(1,2,4\right)})=v(I_{m}-ABB^{\left(1\right)}A^{\left(1,4\right)})=v(I_{m}-ABB^{\left(1\right)}A^{\left(1,2,3\right)})=v(I_{m}-ABB^{\left(1\right)}A^{\left(1,3\right)})=v(I_{m}-ABB^{\left(1\right)}A^{\left(1,2\right)})=v(I_{m}-ABB^{\left(1\right)}A^{\left(1\right)})=m-r(AB).$$
(p) *The following rank equality holds*
  $$u(B^{\left(s_{2},\ldots ,t_{2}\right)}A^{\left(s_{1},\ldots ,t_{1}\right)}AB)=r(AB)$$
  *for all the eight commonly-used types of generalized inverses* $A^{\left(s_{1},\ldots ,t_{1}\right)}$ *and* $B^{\left(s_{2},\ldots ,t_{2}\right)}$*.*
(q) *The following 4 minimum rank formulas hold*
  $$v(B^{\left(1,3,4\right)}A^{†}AB)=v(B^{\left(1,2,3\right)}A^{†}AB)=v(B^{\left(1,3\right)}A^{†}AB)=r(AB);$$
  *the following 8 minimum rank formulas hold*
  $$v(B^{†}A^{\left(1\right)}AB)=v(B^{\left(1,3,4\right)}A^{\left(1\right)}AB)=v(B^{\left(1,2,3\right)}A^{\left(1\right)}AB)=v(B^{\left(1,3\right)}A^{\left(1\right)}AB)=v(B^{\left(1,2,4\right)}A^{†}AB)=v(B^{\left(1,4\right)}A^{†}AB)=v(B^{\left(1,2\right)}A^{†}AB)=v(B^{\left(1\right)}A^{†}AB)=r(A)+r(B)-r(N);$$
  *the following 4 minimum rank formulas hold*
  $$v(B^{\left(1,2,4\right)}A^{\left(1\right)}AB)=v(B^{\left(1,4\right)}A^{\left(1\right)}AB)=v(B^{\left(1,2\right)}A^{\left(1\right)}AB)=v(B^{\left(1\right)}A^{\left(1\right)}AB)=\max\{0,\;\;r(A)+r(B)-n\}.$$
(r) *The following 4 rank equalities hold for all the generalized inverses*
  $$r(I_{p}-B^{†}A^{†}AB)=r(I_{p}-B^{\left(1,3,4\right)}A^{†}AB)=r(I_{p}-B^{\left(1,2,3\right)}A^{†}AB)=r(I_{p}-B^{\left(1,3\right)}A^{†}AB)=r(N)-r(A)-r(B)+p.$$
(s) *The following 8 maximum rank formulas hold*
  $$u(I_{p}-B^{\left(1,2,4\right)}A^{†}AB)=u(I_{p}-B^{\left(1,4\right)}A^{†}AB)=u(I_{p}-B^{\left(1,2\right)}A^{†}AB)=u(I_{p}-B^{\left(1\right)}A^{†}AB)=u(I_{p}-B^{†}A^{\left(1\right)}AB)=u(I_{p}-B^{\left(1,3,4\right)}A^{\left(1\right)}AB)=u(I_{p}-B^{\left(1,2,3\right)}A^{\left(1\right)}AB)=u(I_{p}-B^{\left(1,3\right)}A^{\left(1\right)}AB)=r(N)-r(A)-r(B)+p;$$
  *the following 4 maximum rank formulas hold*
  $$u(I_{p}-B^{\left(1,2,4\right)}A^{\left(1\right)}AB)=u(I_{p}-B^{\left(1,4\right)}A^{\left(1\right)}AB)=u(I_{p}-B^{\left(1,2\right)}A^{\left(1\right)}AB)=u(I_{p}-B^{\left(1\right)}A^{\left(1\right)}AB)=\min\{p,\;\;n+p-r(A)-r(B)\}.$$
(t) *The following 12 minimum rank formulas hold*
  $$v(I_{p}-B^{\left(1,2,4\right)}A^{†}AB)=v(I_{p}-B^{\left(1,4\right)}A^{†}AB)=v(I_{p}-B^{\left(1,2\right)}A^{†}AB)=v(I_{p}-B^{\left(1\right)}A^{†}AB)=v(I_{p}-B^{†}A^{\left(1\right)}AB)=v(I_{p}-B^{\left(1,3,4\right)}A^{\left(1\right)}AB)=v(I_{p}-B^{\left(1,2,3\right)}A^{\left(1\right)}AB)=v(I_{p}-B^{\left(1,3\right)}A^{\left(1\right)}AB)=v(I_{p}-B^{\left(1,2,4\right)}A^{\left(1\right)}AB)=v(I_{p}-B^{\left(1,4\right)}A^{\left(1\right)}AB)=v(I_{p}-B^{\left(1,2\right)}A^{\left(1\right)}AB)=v(I_{p}-B^{\left(1\right)}A^{\left(1\right)}AB)=p-r(AB).$$
(u) *The following maximum rank equality holds*
  $$u(ABB^{\left(s_{2},\ldots ,t_{2}\right)}A^{\left(s_{1},\ldots ,t_{1}\right)}AB)=r(AB)$$
  *for all the eight commonly-used types of generalized inverses* $A^{\left(s_{1},\ldots ,t_{1}\right)}$ *and* $B^{\left(s_{2},\ldots ,t_{2}\right)}$*.*
(v) *The following 2 minimum rank formulas hold*
  $$v(BB^{†}A^{\left(1\right)}AB)=v(ABB^{\left(1\right)}A^{†}AB)=r(A)+r(B)-r(N);$$
  *the following minimum rank formula holds*
  $$v(ABB^{\left(1\right)}A^{\left(1\right)}AB)=\max\{0,\;\;r(A)+r(B)-n\}.$$

Theorem 8.5 *Let* $A\in C^{m\times n}$ *and* $B\in C^{n\times p}$*, and denote* M=AB*,* $H=ABB^{†}A^{†}AB$*, and* $N=[A^{⁎},\;B]$*. The following rank formulas hold*

(8.3) $$r(M-MB^{†}A^{†}M)=r(AE_{B}F_{A}B)=r(N)+r(AB)-r(A)-r(B),$$

(8.4) $$\max_{A^{\left(1\right)}}r(M-MB^{†}A^{\left(1\right)}M)=r(N)+r(M)-r(A)-r(B),$$

(8.5) $$\max_{A^{\left(1\right)}}r(H-MB^{†}A^{\left(1\right)}M)=r(N)+r(M)-r(A)-r(B),$$

(8.6) $$\max_{B^{\left(1\right)}}r(M-MB^{\left(1\right)}A^{†}M)=r(N)+r(M)-r(A)-r(B),$$

(8.7) $$\max_{B^{\left(1\right)}}r(H-MB^{\left(1\right)}A^{†}M)=r(N)+r(M)-r(A)-r(B),$$

(8.8) $$\max_{A^{\left(1\right)},\;B^{\left(1\right)}}r(M-MB^{\left(1\right)}A^{\left(1\right)}M)=\min\{r(M),\;\;r(M)-r(A)-r(B)+n\},$$

(8.9) $$\max_{A^{\left(1\right)},\;B^{\left(1\right)}}r(H-MB^{\left(1\right)}A^{\left(1\right)}M)=\min\{r(M),\;r(N)+2r(M)-2r(A)-2r(B)+n\}.$$

Proof It is easy to verify that $AE_{B}F_{A}B=A(I_{n}-BB^{†}-A^{†}A+BB^{†}A^{†}A)B=MB^{†}A^{†}M-M$. The last rank formula in (8.3) was established in [7]. Subsequently, we give a direct proof of the last rank formula in (8.3). Applying (4.8) and (8.2) to $M-MB^{†}A^{†}M$ gives

$$r(M-MB^{†}A^{†}M)=r(AB+ABPJ^{†}QAB)=r\left[\begin{array}{ccc} B^{⁎}A^{⁎} & B^{⁎}BB^{⁎} & 0\\ A^{⁎}AA^{⁎} & 0 & A^{⁎}AB\\ 0 & ABB^{⁎} & -AB \end{array}\right]-r(A)-r(B)=r\left[\begin{array}{ccc} B^{⁎}A^{⁎} & B^{⁎}B & 0\\ AA^{⁎} & 0 & AB\\ 0 & AB & -AB \end{array}\right]-r(A)-r(B)=r\left[\begin{array}{ccc} B^{⁎}A^{⁎} & B^{⁎}B & 0\\ AA^{⁎} & AB & 0\\ 0 & 0 & -AB \end{array}\right]-r(A)-r(B)=r({\left[A^{⁎},\;B\right]}^{⁎}[A^{⁎},\;B])+r(AB)-r(A)-r(B)=r(N)+r(AB)-r(A)-r(B),$$

thus establishing the last two rank formulas in (8.3). By (4.30),

$$\max_{A^{\left(1\right)}}r(M-MB^{†}A^{\left(1\right)}M)=\min\left\{r[M,\;MB^{†}],\;\;r\left[\begin{array}{c} M\\ M \end{array}\right],\;\;r\left[\begin{array}{cc} A & M\\ MB^{†} & M \end{array}\right]-r(A)\right\}=r\left[\begin{array}{c} A\\ ABB^{†} \end{array}\right]-r(A)=r\left[\begin{array}{c} A-ABB^{†}\\ ABB^{†} \end{array}\right]-r(A)=r(A-ABB^{†})+r(AB)-r(A)=r(N)+r(AB)-r(A)-r(B),\max_{A^{\left(1\right)}}r(H-MB^{†}A^{\left(1\right)}M)=\min\left\{r[H,\;MB^{†}],\;\;r\left[\begin{array}{c} H\\ M \end{array}\right],\;\;r\left[\begin{array}{cc} A & M\\ MB^{†} & H \end{array}\right]-r(A)\right\}=\min\left\{r(M),\;\;r\left[\begin{array}{cc} A & M\\ MB^{†}-MB^{†}A^{†}A & 0 \end{array}\right]-r(A)\right\}=r[A,\;M]+r(MB^{†}-MB^{†}A^{†}A)-r(A)=r\left[\begin{array}{c} MB^{†}\\ A \end{array}\right]-r(A)=r\left[\begin{array}{c} ABB^{†}\\ A-ABB^{†} \end{array}\right]-r(A)=r(ABB^{†})+r(A-ABB^{†})-r(A)=r(N)+r(AB)-r(A)-r(B),$$

as required for (8.4) and (8.5). Eqs. (8.6)–(8.9) can be established by a similar way. □

Finally, we present two groups of simple or well-known equalities and facts concerning ranges and ranks of matrices, which are the fundamental tricks of the MRM and will be used in the derivations and simplifications of calculations of ranks of algebraic operations of matrices and their generalized inverses in the sequel.

Lemma 8.6 *Let* $A\in C^{m\times n}$ *and* $B\in C^{m\times p}$*,* $P\in C^{p\times m}$*,* $M\in C^{n\times n}$*. Then the following results*

(8.10) R(A)⊆R(B)andr(A)=r(B)⇒R(A)=R(B),

(8.11) R(A)⊆R(B)⇒R(PA)⊆R(PB),

(8.12) R(A)=R(B)⇒R(PA)=R(PB),

(8.13) $$R(A)=R(AA^{⁎})=R(AA^{⁎}A)=R(AA^{†})=R\left({\left(A^{†}\right)}^{⁎}\right),$$

(8.14) $$R(A^{⁎})=R(A^{⁎}A)=R(A^{⁎}AA^{⁎})=R(A^{†})=R(A^{†}A),$$

(8.15) $$R(AB^{†}B)=R(AB^{†})=R(AB^{⁎}B)=R(AB^{⁎}),$$

(8.16) $$R(ABB^{⁎}A^{⁎})=R(ABB^{⁎})=R(AB),$$

(8.17) $$R(B^{⁎}A^{⁎}AB)=R(B^{⁎}A^{⁎}A)=R(B^{⁎}A^{⁎}),$$

(8.18) $$R(ABB^{†}A^{†}AB)=R(ABB^{†}A^{†})=R(AB),$$

(8.19) $$R(B^{†}A^{†}ABB^{†}A^{†})=R(B^{†}A^{†}AB)=R(B^{†}A^{†})=R(B^{†}A^{⁎}),$$

(8.20) $$R(A)=R(B)\Leftrightarrow r[A,\;B]=r(A)=r(B)\Leftrightarrow AA^{†}=BB^{†}$$

*hold, and the following rank equalities hold*

(8.21) $$r(AA^{⁎}ABB^{⁎}B)=r(A^{⁎}ABB^{⁎})=r(ABB^{⁎}A^{⁎})=r(B^{⁎}A^{⁎}AB)=r(AB),$$

(8.22) $$r\left({\left(A^{⁎}A\right)}^{1/2}{\left(BB^{⁎}\right)}^{1/2}\right)=r\left({\left(BB^{⁎}\right)}^{1/2}{\left(A^{⁎}A\right)}^{1/2}\right)=r(AB),$$

(8.23) $$r(B^{†}A^{†})=r(B^{⁎}A^{†})=r(B^{†}A^{⁎})=r(AB),$$

(8.24) $$r\left({\left(A^{†}\right)}^{⁎}{\left(B^{†}\right)}^{⁎}\right)=r\left({\left(A^{†}\right)}^{⁎}B\right)=r\left(A{\left(B^{†}\right)}^{⁎}\right)=r(AB),$$

(8.25) $$r(BB^{†}A^{†}A)=r(BB^{†}A^{⁎}A)=r(BB^{⁎}A^{†}A)=r(AB),$$

(8.26) $$r(A^{†}ABB^{†})=r(A^{†}ABB^{⁎})=r(A^{⁎}ABB^{†})=r(AB),$$

(8.27) $$r(ABB^{†}A^{†})=r(ABB^{†}A^{⁎})=r(ABB^{⁎}A^{†})=r(AB),$$

(8.28) $$r(B^{†}A^{†}AB)=r(B^{†}A^{⁎}AB)=r(B^{⁎}A^{†}AB)=r(AB),$$

(8.29) $$r(ABB^{†}A^{†}AB)=r(AB^{†}A^{⁎}AB)=r(ABB^{⁎}A^{†}AB)=r(AB),$$

(8.30) $$r\left({\left(BB^{⁎}\right)}^{†}{\left(A^{⁎}A\right)}^{†})\right)=r\left({\left(BB^{⁎}\right)}^{†}(A^{⁎}A)\right)=r\left(BB^{⁎}){\left(A^{⁎}A\right)}^{†})\right)=r(AB),$$

(8.31) $$r\left(B^{†}{\left(A^{⁎}A\right)}^{†}\right)=r(B^{†}A^{⁎}A)=r\left(B^{⁎}{\left(A^{⁎}A\right)}^{†})\right)=r(AB),$$

(8.32) $$r\left({\left(BB^{⁎}\right)}^{†}A^{†}\right)=r\left({\left(BB^{⁎}\right)}^{†}A^{⁎}\right)=r(BB^{⁎}A^{†})=r(AB),$$

(8.33) $$r(B^{†}MA^{†})=r(B^{⁎}MA^{†})=r(B^{†}MA^{⁎})=r(B^{⁎}MA^{⁎}),$$

(8.34) $$r(BB^{†}MA^{†}A)=r(BB^{⁎}MA^{†})=r(BB^{†}MA^{⁎}A)=r(BB^{⁎}MA^{⁎}A)=r(B^{⁎}MA^{⁎}).$$

## 9 Set inclusions for {1}-, {1,2}-, {1,3}-, and {1,4}-generalized inverses of *AB*

After sufficient preparations of the background materials and tools in the preceding sections, we are now at the point to address the 512 set inclusion problems that are formulated in (1.10). This section focuses on the four groups of set inclusions for $\left\{{\left(AB\right)}^{\left(1\right)}\right\}$, $\left\{{\left(AB\right)}^{\left(1,2\right)}\right\}$, $\left\{{\left(AB\right)}^{\left(1,3\right)}\right\}$, $\left\{{\left(AB\right)}^{\left(1,4\right)}\right\}$ in (1.10). We first mention that several cases of the set inclusions in (1.10) were considered in the literature, such as,

$$\;\;\;\;\;\{{\left(AB\right)}^{\left(1\right)}\}⊇\{B^{1)}A^{\left(1\right)}\},\;\;\;\{{\left(AB\right)}^{\left(1,2\right)}\}⊇\{B^{1,2)}A^{\left(1,2\right)}\},\;\;\;\{{\left(AB\right)}^{\left(1,3\right)}\}⊇\{B^{1,3)}A^{\left(1,3\right)}\},\{{\left(AB\right)}^{\left(1,4\right)}\}⊇\{B^{1,4)}A^{\left(1,4\right)}\},\;\;\;\{{\left(AB\right)}^{\left(1,2,3\right)}\}⊇\{B^{1,2,3)}A^{\left(1,2,3\right)}\},\;\;\{{\left(AB\right)}^{\left(1,2,4\right)}\}⊇\{B^{1,2,4)}A^{\left(1,2,4\right)}\}$$

(cf. [20], [21], [23], [28], [50], [52], [54], [64], [77], [81], [92], [114], [116], [117], [119], [123], [126]). Despite the fact that ROLs have been around for a long time, there are still many open problems regarding ROLs under various assumptions.

For the first matrix set $\left\{{\left(AB\right)}^{\left(1\right)}\right\}$, it can be seen from (3.63) that

(9.1) $$\{{\left(AB\right)}^{\left(1\right)}\}⊇\{B^{\left(s_{2},\ldots ,t_{2}\right)}A^{\left(s_{1},\ldots ,t_{1}\right)}\}\Leftrightarrow ABB^{\left(s_{2},\ldots ,t_{2}\right)}A^{\left(s_{1},\ldots ,t_{1}\right)}AB=AB$$

hold for the eight commonly-used types of generalized inverses $A^{\left(s_{1},\ldots ,t_{1}\right)}$ and $B^{\left(s_{2},\ldots ,t_{2}\right)}$, respectively. In this situation, by substitution of (3.182)–(3.184) into (9.1), the equalities in (9.1) are converted to three linear or multilinear matrix equations with some unknown matrices for the eight commonly-used types of generalized inverses of *A* and *B*. The first group of results in this section describe various equivalent statements for the 64 ROLs in (9.1) to hold, which correspond in turn to the statistical fact in (2.28).

Theorem 9.1 *Let* $A\in C^{m\times n}$ *and* $B\in C^{n\times p}$*. Then the following 165 statements are equivalent:*

$$\;\begin{array}{cccc} \langle 1\rangle \; & \{{\left(AB\right)}^{\left(1\right)}\}⊇\{B^{†}A^{\left(1\right)}\}.\; & \;\langle 2\rangle \; & \{{\left(AB\right)}^{\left(1\right)}\}⊇\{B^{†}A^{\left(1,2\right)}\}.\\ \langle 3\rangle \; & \{{\left(AB\right)}^{\left(1\right)}\}⊇\{B^{†}A^{\left(1,3\right)}\}.\; & \langle 4\rangle \; & \{{\left(AB\right)}^{\left(1\right)}\}⊇\{B^{†}A^{\left(1,4\right)}\}.\\ \langle 5\rangle \; & \{{\left(AB\right)}^{\left(1\right)}\}⊇\{B^{†}A^{\left(1,2,3\right)}\}.\; & \langle 6\rangle \; & \{{\left(AB\right)}^{\left(1\right)}\}⊇\{B^{†}A^{\left(1,2,4\right)}\}.\\ \langle 7\rangle \; & \{{\left(AB\right)}^{\left(1\right)}\}⊇\{B^{†}A^{\left(1,3,4\right)}\}.\; & \langle 8\rangle \; & \{{\left(AB\right)}^{\left(1\right)}\}⊇\{B^{\left(1,3,4\right)}A^{\left(1\right)}\}.\\ \langle 9\rangle \; & \{{\left(AB\right)}^{\left(1\right)}\}⊇\{B^{\left(1,3,4\right)}A^{\left(1,2\right)}\}.\; & \langle 10\rangle \; & \{{\left(AB\right)}^{\left(1\right)}\}⊇\{B^{\left(1,3,4\right)}A^{\left(1,3\right)}\}.\\ \langle 11\rangle \; & \{{\left(AB\right)}^{\left(1\right)}\}⊇\{B^{\left(1,3,4\right)}A^{\left(1,4\right)}\}.\; & \langle 12\rangle \; & \{{\left(AB\right)}^{\left(1\right)}\}⊇\{B^{\left(1,3,4\right)}A^{\left(1,2,3\right)}\}.\\ \langle 13\rangle \; & \{{\left(AB\right)}^{\left(1\right)}\}⊇\{B^{\left(1,3,4\right)}A^{\left(1,2,4\right)}\}.\; & \langle 14\rangle \; & \{{\left(AB\right)}^{\left(1\right)}\}⊇\{B^{\left(1,3,4\right)}A^{\left(1,3,4\right)}\}.\\ \langle 15\rangle \; & \{{\left(AB\right)}^{\left(1\right)}\}⊇\{B^{\left(1,3,4\right)}A^{†}\}.\; & \langle 16\rangle \; & \left\{{\left(AB\right)}^{\left(1\right)}\}⊇\{B^{\left(1,2,4\right)}A^{\left(1,4\right)}\right\}\\ \langle 17\rangle \; & \{{\left(AB\right)}^{\left(1\right)}\}⊇\{B^{\left(1,2,4\right)}A^{\left(1,2,4\right)}\}.\; & \langle 18\rangle \; & \{{\left(AB\right)}^{\left(1\right)}\}⊇\{B^{\left(1,2,4\right)}A^{\left(1,3,4\right)}\}.\\ \langle 19\rangle \; & \{{\left(AB\right)}^{\left(1\right)}\}⊇\{B^{\left(1,2,4\right)}A^{†}\}.\; & \langle 20\rangle \; & \{{\left(AB\right)}^{\left(1\right)}\}⊇\{B^{\left(1,2,3\right)}A^{\left(1\right)}\}.\\ \langle 21\rangle \; & \{{\left(AB\right)}^{\left(1\right)}\}⊇\{B^{\left(1,2,3\right)}A^{\left(1,2\right)}\}.\; & \langle 22\rangle \; & \{{\left(AB\right)}^{\left(1\right)}\}⊇\{B^{\left(1,2,3\right)}A^{\left(1,3\right)}\}.\\ \langle 23\rangle \; & \{{\left(AB\right)}^{\left(1\right)}\}⊇\{B^{\left(1,2,3\right)}A^{\left(1,4\right)}\}.\; & \langle 24\rangle \; & \{{\left(AB\right)}^{\left(1\right)}\}⊇\{B^{\left(1,2,3\right)}A^{\left(1,2,3\right)}\}.\\ \langle 25\rangle \; & \{{\left(AB\right)}^{\left(1\right)}\}⊇\{B^{\left(1,2,3\right)}A^{\left(1,2,4\right)}\}.\; & \langle 26\rangle \; & \{{\left(AB\right)}^{\left(1\right)}\}⊇\{B^{\left(1,2,3\right)}A^{\left(1,3,4\right)}\}.\\ \langle 27\rangle \; & \{{\left(AB\right)}^{\left(1\right)}\}⊇\{B^{\left(1,2,3\right)}A^{†}\}.\; & \langle 28\rangle \; & \{{\left(AB\right)}^{\left(1\right)}\}⊇\{B^{\left(1,4\right)}A^{\left(1,4\right)}\}.\\ \langle 29\rangle \; & \{{\left(AB\right)}^{\left(1\right)}\}⊇\{B^{\left(1,4\right)}A^{\left(1,2,4\right)}\}.\; & \langle 30\rangle \; & \{{\left(AB\right)}^{\left(1\right)}\}⊇\{B^{\left(1,4\right)}A^{\left(1,3,4\right)}\}.\\ \langle 31\rangle \; & \{{\left(AB\right)}^{\left(1\right)}\}⊇\{B^{\left(1,4\right)}A^{†}\}.\; & \langle 32\rangle \; & \{{\left(AB\right)}^{\left(1\right)}\}⊇\{B^{\left(1,3\right)}A^{\left(1\right)}\}.\\ \langle 33\rangle \; & \{{\left(AB\right)}^{\left(1\right)}\}⊇\{B^{\left(1,3\right)}A^{\left(1,2\right)}\}.\; & \langle 34\rangle \; & \{{\left(AB\right)}^{\left(1\right)}\}⊇\{B^{\left(1,3\right)}A^{\left(1,3\right)}\}.\\ \langle 35\rangle \; & \{{\left(AB\right)}^{\left(1\right)}\}⊇\{B^{\left(1,3\right)}A^{\left(1,4\right)}\}.\; & \langle 36\rangle \; & \{{\left(AB\right)}^{\left(1\right)}\}⊇\{B^{\left(1,3\right)}A^{\left(1,2,3\right)}\}.\\ \langle 37\rangle \; & \{{\left(AB\right)}^{\left(1\right)}\}⊇\{B^{\left(1,3\right)}A^{\left(1,2,4\right)}\}.\; & \langle 38\rangle \; & \{{\left(AB\right)}^{\left(1\right)}\}⊇\{B^{\left(1,3\right)}A^{\left(1,3,4\right)}\}.\\ \langle 39\rangle \; & \{{\left(AB\right)}^{\left(1\right)}\}⊇\{B^{\left(1,3\right)}A^{†}\}.\; & \langle 40\rangle \; & \{{\left(AB\right)}^{\left(1\right)}\}⊇\{B^{\left(1,2\right)}A^{\left(1,4\right)}\}.\\ \langle 41\rangle \; & \{{\left(AB\right)}^{\left(1\right)}\}⊇\{B^{\left(1,2\right)}A^{\left(1,2,4\right)}\}.\; & \langle 42\rangle \; & \{{\left(AB\right)}^{\left(1\right)}\}⊇\{B^{\left(1,2\right)}A^{\left(1,3,4\right)}\}.\\ \langle 43\rangle \; & \{{\left(AB\right)}^{\left(1\right)}\}⊇\{B^{\left(1,2\right)}A^{†}\}.\; & \langle 44\rangle \; & \{{\left(AB\right)}^{\left(1\right)}\}⊇\{B^{\left(1\right)}A^{\left(1,4\right)}\}.\\ \langle 45\rangle \; & \{{\left(AB\right)}^{\left(1\right)}\}⊇\{B^{\left(1\right)}A^{\left(1,2,4\right)}\}.\; & \langle 46\rangle \; & \{{\left(AB\right)}^{\left(1\right)}\}⊇\{B^{\left(1\right)}A^{\left(1,3,4\right)}\}.\\ \langle 47\rangle \; & \{{\left(AB\right)}^{\left(1\right)}\}⊇\{B^{\left(1\right)}A^{†}\}.\; & \langle 48\rangle \; & \{{\left(AB\right)}^{\left(1\right)}\}∋B^{†}A^{†}.\\ \langle 49\rangle \; & \{AB{\left(AB\right)}^{\left(1\right)}\}⊇\{ABB^{†}A^{\left(1\right)}\}.\; & \langle 50\rangle \; & \{AB{\left(AB\right)}^{\left(1\right)}\}⊇\{ABB^{†}A^{\left(1,2\right)}\}.\\ \langle 51\rangle \; & \{AB{\left(AB\right)}^{\left(1\right)}\}⊇\{ABB^{†}A^{\left(1,3\right)}\}.\; & \langle 52\rangle \; & \{AB{\left(AB\right)}^{\left(1\right)}\}⊇\{ABB^{†}A^{\left(1,4\right)}\}.\\ \langle 53\rangle \; & \{AB{\left(AB\right)}^{\left(1\right)}\}⊇\{ABB^{†}A^{\left(1,2,3\right)}\}.\; & \langle 54\rangle \; & \{AB{\left(AB\right)}^{\left(1\right)}\}⊇\{ABB^{†}A^{\left(1,2,4\right)}\}.\\ \langle 55\rangle \; & \{AB{\left(AB\right)}^{\left(1\right)}\}⊇\{ABB^{†}A^{\left(1,3,4\right)}\}.\; & \langle 56\rangle \; & \{AB{\left(AB\right)}^{\left(1\right)}\}∋ABB^{†}A^{†}.\\ \langle 57\rangle \; & \{AB{\left(AB\right)}^{\left(1\right)}\}⊇\{ABB^{\left(1\right)}A^{\left(1,4\right)}\}.\; & \langle 58\rangle \; & \{AB{\left(AB\right)}^{\left(1\right)}\}⊇\{ABB^{\left(1\right)}A^{\left(1,2,4\right)}\}.\\ \langle 59\rangle \; & \{AB{\left(AB\right)}^{\left(1\right)}\}⊇\{ABB^{\left(1\right)}A^{\left(1,3,4\right)}\}.\; & \langle 60\rangle \; & \{AB{\left(AB\right)}^{\left(1\right)}\}⊇\{ABB^{\left(1\right)}A^{†}\}.\\ \langle 61\rangle \; & \{{\left(AB\right)}^{\left(1\right)}AB\}⊇\{B^{\left(1\right)}A^{†}AB\}.\; & \langle 62\rangle \; & \{{\left(AB\right)}^{\left(1\right)}AB\}⊇\{B^{\left(1,2\right)}A^{†}AB\}.\\ \langle 63\rangle \; & \{{\left(AB\right)}^{\left(1\right)}AB\}⊇\{B^{\left(1,3\right)}A^{†}AB\}.\; & \langle 64\rangle \; & \{{\left(AB\right)}^{\left(1\right)}AB\}⊇\{B^{\left(1,4\right)}A^{†}AB\}.\\ \langle 65\rangle \; & \{{\left(AB\right)}^{\left(1\right)}AB\}⊇\{B^{\left(1,2,3\right)}A^{†}AB\}.\; & \langle 66\rangle \; & \{{\left(AB\right)}^{\left(1\right)}AB\}⊇\{B^{\left(1,2,4\right)}A^{†}AB\}.\\ \langle 67\rangle \; & \{{\left(AB\right)}^{\left(1\right)}AB\}⊇\{B^{\left(1,3,4\right)}A^{†}AB\}.\; & \langle 68\rangle \; & \{{\left(AB\right)}^{\left(1\right)}AB\}∋B^{†}A^{†}AB.\\ \langle 69\rangle \; & \{{\left(AB\right)}^{\left(1\right)}AB\}⊇\{B^{\left(1,3\right)}A^{\left(1\right)}AB\}.\; & \langle 70\rangle \; & \{{\left(AB\right)}^{\left(1\right)}AB\}⊇\{B^{\left(1,4\right)}A^{\left(1\right)}AB\}.\\ \langle 71\rangle \; & \{{\left(AB\right)}^{\left(1\right)}AB\}⊇\{B^{\left(1,2,3\right)}A^{\left(1\right)}AB\}.\; & \langle 72\rangle \; & \{{\left(AB\right)}^{\left(1\right)}AB\}⊇\{B^{\left(1,3,4\right)}A^{\left(1\right)}AB\}.\\ \langle 73\rangle \; & \{B{\left(AB\right)}^{\left(1\right)}A\}⊇\{BB^{\left(1\right)}A^{\left(1\right)}A\}.\; & \langle 74\rangle \; & \{B{\left(AB\right)}^{\left(1\right)}A\}⊇\{BB^{†}A^{\left(1\right)}A\}.\\ \langle 75\rangle \; & \{B{\left(AB\right)}^{\left(1\right)}A\}⊇\{BB^{\left(1\right)}A^{†}A\}.\; & \langle 76\rangle \; & \{B{\left(AB\right)}^{\left(1\right)}A\}∋BB^{†}A^{†}A. \end{array}$$

〈77〉 $\left\{{\left({\left(A^{†}\right)}^{⁎}B\right)}^{\left(1\right)}\right\}∋B^{†}A^{⁎}$ *and/or* $\left\{{\left(A{\left(B^{†}\right)}^{⁎}\right)}^{\left(1\right)}\right\}∋B^{⁎}A^{†}$*.*
〈78〉 $\left\{{\left(A^{⁎}AB\right)}^{\left(1\right)}\right\}∋B^{†}{\left(A^{⁎}A\right)}^{†}$ *and/or* $\left\{{\left(ABB^{⁎}\right)}^{\left(1\right)}\right\}∋{\left(BB^{⁎}\right)}^{†}A^{†}$*.*
〈79〉 $\left\{{\left(A^{⁎}ABB^{⁎}\right)}^{\left(1\right)}\right\}∋{\left(BB^{⁎}\right)}^{†}{\left(A^{⁎}A\right)}^{†}$ *and/or* $\left\{{\left(BB^{⁎}A^{⁎}A\right)}^{\left(1\right)}\right\}∋{\left(A^{⁎}A\right)}^{†}{\left(BB^{⁎}\right)}^{†}$*.*
〈80〉 $\left\{{\left({\left(A^{⁎}A\right)}^{1/2}{\left(BB^{⁎}\right)}^{1/2}\right)}^{\left(1\right)}\right\}∋{\left({\left(BB^{⁎}\right)}^{1/2}\right)}^{†}{\left({\left(A^{⁎}A\right)}^{1/2}\right)}^{†}$ *and/or* $\left\{{\left({\left(BB^{⁎}\right)}^{1/2}{\left(A^{⁎}A\right)}^{1/2}\right)}^{\left(1\right)}\right\}∋{\left({\left(A^{⁎}A\right)}^{1/2}\right)}^{†}{\left({\left(BB^{⁎}\right)}^{1/2}\right)}^{†}$*.*
〈81〉 $\left\{{\left(AA^{⁎}ABB^{⁎}B\right)}^{\left(1\right)}\right\}∋{\left(BB^{⁎}B\right)}^{†}{\left(AA^{⁎}A\right)}^{†}$*.*
〈82〉 $\left\{{\left(A^{†}AB\right)}^{\left(1\right)}\right\}∋B^{†}A^{†}A$ *and/or* $\left\{{\left(ABB^{†}\right)}^{\left(1\right)}\right\}∋BB^{†}A^{†}$*.*
〈83〉 $\left\{{\left(A^{†}ABB^{†}\right)}^{\left(1\right)}\right\}∋BB^{†}A^{†}A$ *and/or* $\left\{{\left(BB^{†}A^{†}A\right)}^{\left(1\right)}\right\}∋A^{†}ABB^{†}$*.*
〈84〉 $\left\{{\left(F_{A}BB^{†}\right)}^{\left(1\right)}\right\}∋BB^{†}F_{A}$ *and/or* $\left\{{\left(BB^{†}F_{A}\right)}^{\left(1\right)}\right\}∋F_{A}BB^{†}$*.*
〈85〉 $\left\{{\left(A^{†}AE_{B}\right)}^{\left(1\right)}\right\}∋E_{B}A^{†}A$ *and/or* $\left\{{\left(E_{B}A^{†}A\right)}^{\left(1\right)}\right\}∋A^{†}AE_{B}$*.*
〈86〉 $\left\{{\left(F_{A}E_{B}\right)}^{\left(1\right)}\right\}\in E_{B}F_{A}$ *and/or* $\left\{{\left(E_{B}F_{A}\right)}^{\left(1\right)}\right\}∋F_{A}E_{B}$*.*
〈87〉 $ABB^{†}A^{†}AB=AB$ *and/or* $AE_{B}F_{A}B=0$*.*
〈88〉 $B^{†}A^{†}ABB^{†}A^{†}=B^{†}A^{†}$ *and/or* $B^{†}F_{A}E_{B}A^{†}=0$*.*
〈89〉 $ABB^{†}F_{A}=0$ *and/or* $E_{B}A^{†}AB=0$*.*
〈90〉 $AE_{B}F_{A}=0$ *and/or* $E_{B}F_{A}B=0$*.*
〈91〉 *One/all of the four facts:* $A^{†}ABB^{†}=BB^{†}A^{†}A$*,* $F_{A}BB^{†}=BB^{†}F_{A}$*,* $A^{†}AE_{B}=E_{B}A^{†}A$*,* $F_{A}E_{B}=E_{B}F_{A}$*.*
〈92〉 ${\left(A^{†}ABB^{†}\right)}^{2}=A^{†}ABB^{†}$ *and/or* ${\left(BB^{†}A^{†}A\right)}^{2}=BB^{†}A^{†}A$*.*
〈93〉 ${\left(ABB^{†}A^{†}\right)}^{2}=ABB^{†}A^{†}$ *and/or* ${\left(B^{†}A^{†}AB\right)}^{2}=B^{†}A^{†}AB$*.*
〈94〉 ${\left(F_{A}BB^{†}\right)}^{2}=F_{A}BB^{†}$ *and/or* ${\left(BB^{†}F_{A}\right)}^{2}=BB^{†}F_{A}$*.*
〈95〉 ${\left(A^{†}AE_{B}\right)}^{2}=A^{†}AE_{B}$ *and/or* ${\left(E_{B}A^{†}A\right)}^{2}=E_{B}A^{†}A$*.*
〈96〉 ${\left(F_{A}E_{B}\right)}^{2}=F_{A}E_{B}$ *and/or* ${\left(E_{B}F_{A}\right)}^{2}=E_{B}F_{A}$*.*
〈97〉 ${\left(AE_{B}A^{†}\right)}^{2}=AE_{B}A^{†}$ *and/or* ${\left(B^{†}F_{A}B\right)}^{2}=B^{†}F_{A}B$*.*
〈98〉 ${\left(A^{†}ABB^{†}\right)}^{†}=BB^{†}A^{†}A$ *and/or* ${\left(BB^{†}A^{†}A\right)}^{†}=A^{†}ABB^{†}$*.*
〈99〉 ${\left(BB^{†}F_{A}\right)}^{†}=F_{A}BB^{†}$ *and/or* ${\left(F_{A}BB^{†}\right)}^{†}=BB^{†}F_{A}$*.*
〈100〉 ${\left(A^{†}AE_{B}\right)}^{†}=E_{B}A^{†}A$ *and/or* ${\left(E_{B}A^{†}A\right)}^{†}=A^{†}AE_{B}$*.*
〈101〉 ${\left(F_{A}E_{B}\right)}^{†}=E_{B}F_{A}$ *and/or* ${\left(E_{B}F_{A}\right)}^{†}=F_{A}E_{B}$*.*
〈102〉 ${\left(A^{†}A-BB^{†}\right)}^{†}=A^{†}A-BB^{†}$ *and/or* ${\left(BB^{†}-A^{†}A\right)}^{†}=BB^{†}-A^{†}A$*.*
〈103〉 ${\left(I_{n}-A^{†}A-BB^{†}\right)}^{†}=I_{n}-A^{†}A-BB^{†}$*.*
〈104〉 ${\left(I_{m}-ABB^{†}A^{†}\right)}^{†}=I_{m}-ABB^{†}A^{†}$ *and/or* ${\left(I_{p}-B^{†}A^{†}AB\right)}^{†}=I_{p}-B^{†}A^{†}AB$*.*
〈105〉 ${\left(I_{n}-A^{†}ABB^{†}\right)}^{†}=I_{n}-A^{†}ABB^{†}$ *and/or* ${\left(I_{n}-BB^{†}A^{†}A\right)}^{†}=I_{n}-BB^{†}A^{†}A$*.*
〈106〉 ${\left(I_{m}-AE_{B}A^{†}\right)}^{†}=I_{m}-AE_{B}A^{†}$ *and/or* ${\left(I_{p}-B^{†}F_{A}B\right)}^{†}=I_{p}-B^{†}F_{A}B$*.*
〈107〉 ${\left(I_{n}-F_{A}BB^{†}\right)}^{†}=I_{n}-F_{A}BB^{†}$ *and/or* ${\left(I_{n}-BB^{†}F_{A}\right)}^{†}=I_{n}-BB^{†}F_{A}$*.*
〈108〉 ${\left(I_{n}-A^{†}AE_{B}\right)}^{†}=I_{n}-A^{†}AE_{B}$ *and/or* ${\left(I_{n}-E_{B}A^{†}A\right)}^{†}=I_{n}-E_{B}A^{†}A$*.*
〈109〉 ${\left(I_{n}-F_{A}E_{B}\right)}^{†}=I_{n}-F_{A}E_{B}$ *and/or* ${\left(I_{n}-E_{B}F_{A}\right)}^{†}=I_{n}-E_{B}F_{A}$*.*
〈110〉 $[A^{⁎},\;B]{\left[A^{⁎},\;B\right]}^{†}=A^{†}A+BB^{†}-A^{†}ABB^{†}$ *and/or* $[A^{⁎},\;B]{\left[A^{⁎},\;B\right]}^{†}=A^{†}A+BB^{†}-BB^{†}A^{†}A$*.*
〈111〉 $[F_{A},\;B]{\left[F_{A},\;B\right]}^{†}=F_{A}+BB^{†}-F_{A}BB^{†}$ *and/or* $[F_{A},\;B]{\left[F_{A},\;B\right]}^{†}=F_{A}+BB^{†}-BB^{†}F_{A}$*.*
〈112〉 $[A^{⁎},\;E_{B}]{\left[A^{⁎},\;E_{B}\right]}^{†}=A^{†}A+E_{B}-A^{†}AE_{B}$ *and/or* $[A^{⁎},\;E_{B}]{\left[A^{⁎},\;E_{B}\right]}^{†}=A^{†}A+E_{B}-E_{B}A^{†}A$*.*
〈113〉 $[F_{A},\;E_{B}]{\left[F_{A},\;E_{B}\right]}^{†}=F_{A}+E_{B}-F_{A}E_{B}$ *and/or* $[F_{A},\;E_{B}]{\left[F_{A},\;E_{B}\right]}^{†}=F_{A}+E_{B}-E_{B}F_{A}$*.*
〈114〉 $ABB^{†}A^{\left(1\right)}AB$ *is invariant with respect to the choice of* $A^{\left(1\right)}$*, i.e.,* $ABB^{†}A^{\left(1\right)}AB=ABB^{†}A^{†}AB$ *holds for all* $A^{\left(1\right)}$*.*
〈115〉 $ABB^{\left(1\right)}A^{†}AB$ *is invariant with respect to the choice of* $B^{\left(1\right)}$*, i.e.,* $ABB^{\left(1\right)}A^{†}AB=ABB^{†}A^{†}AB$ *holds for all* $B^{\left(1\right)}$*.*
〈116〉 $r[A^{⁎},\;B]=r(A)+r(B)-r(AB)$*.*
〈117〉 $r[F_{A},\;B]=r(F_{A})+r(B)-r(F_{A}B)$*.*
〈118〉 $r[A,\;E_{B}]=r(A)+r(E_{B})-r(AE_{B})$*.*
〈119〉 $r[F_{A},\;E_{B}]=r(F_{A})+r(E_{B})-r(F_{A}E_{B})$*.*
〈120〉 $r(I_{n}-A^{†}ABB^{†})=n-r(A^{†}ABB^{†})$ *and/or* $r(I_{n}-BB^{†}A^{†}A)=n-r(BB^{†}A^{†}A)$*.*
〈121〉 $r(I_{m}-ABB^{†}A^{†})=m-r(ABB^{†}A^{†})$ *and/or* $r(I_{p}-B^{†}A^{†}AB)=p-r(B^{†}A^{†}AB)$*.*
〈122〉 $r(I_{n}-F_{A}BB^{†})=n-r(F_{A}BB^{†})$ *and/or* $r(I_{n}-BB^{†}F_{A})=n-r(BB^{†}F_{A})$*.*
〈123〉 $r(I_{n}-A^{†}AE_{B})=n-r(A^{†}AE_{B})$ *and/or* $r(I_{n}-E_{B}A^{†}A)=n-r(E_{B}A^{†}A)$*.*
〈124〉 $r(I_{n}-F_{A}E_{B})=n-r(F_{A}E_{B})$ *and/or* $r(I_{n}-E_{B}F_{A})=n-r(E_{B}F_{A})$*.*
〈125〉 $r(I_{m}-AE_{B}A^{†})=m-r(AE_{B}A^{†})$ *and/or* $r(I_{p}-B^{†}F_{A}B)=p-r(B^{†}F_{A}B)$*.*
〈126〉 $r(A^{†}A-A^{†}ABB^{†})=r(A^{†}A)-r(A^{†}ABB^{†})$ *and/or* $r(BB^{†}-A^{†}ABB^{†})=r(BB^{†})-r(A^{†}ABB^{†})$*.*
〈127〉 $r(A^{†}A-A^{†}AE_{B})=r(A^{†}A)-r(A^{†}AE_{B})$ *and/or* $r(BB^{†}-F_{A}BB^{†})=r(BB^{†})-r(F_{A}BB^{†})$*.*
〈128〉 $r(F_{A}-F_{A}BB^{†})=r(F_{A})-r(F_{A}BB^{†})$ *and/or* $r(E_{B}-A^{†}AE_{B})=r(E_{B})-r(A^{†}AE_{B})$*.*
〈129〉 $r(F_{A}-F_{A}E_{B})=r(F_{A})-r(F_{A}E_{B})$ *and* $r(E_{B}-F_{A}E_{B})=r(E_{B})-r(F_{A}E_{B})$*.*
〈130〉 $R(I_{n}-A^{†}ABB^{†})\cap R(A^{†}ABB^{†})=\{0\}$ *and/or* $R(I_{n}-BB^{†}A^{†}A)\cap R(BB^{†}A^{†}A)=\{0\}$*.*
〈131〉 $R(I_{n}-F_{A}BB^{†})\cap R(F_{A}BB^{†})=\{0\}$ *and/or* $R(I_{n}-BB^{†}F_{A})\cap R(BB^{†}F_{A})=\{0\}$*.*
〈132〉 $R(I_{n}-A^{†}AE_{B})\cap R(A^{†}AE_{B})=\{0\}$ *and/or* $R(I_{n}-E_{B}A^{†}A)\cap R(E_{B}A^{†}A)=\{0\}$*.*
〈133〉 $R(I_{n}-F_{A}E_{B})\cap R(F_{A}E_{B})=\{0\}$ *and/or* $R(I_{n}-E_{B}F_{A})\cap R(E_{B}F_{A})=\{0\}$*.*
〈134〉 $R(I_{m}-ABB^{†}A^{†})\cap R(ABB^{†}A^{†})=\{0\}$ *and/or* $R\left({\left(I_{m}-ABB^{†}A^{†}\right)}^{⁎}\right)\cap R\left({\left(ABB^{†}A^{†}\right)}^{⁎}\right)=\{0\}$*.*
〈135〉 $R(I_{p}-B^{†}A^{†}AB)\cap R(B^{†}A^{†}AB)=\{0\}$ *and/or* $R\left({\left(I_{p}-B^{†}A^{†}AB\right)}^{⁎}\right)\cap R\left({\left(B^{†}A^{†}AB\right)}^{⁎}\right)=\{0\}$*.*
〈136〉 $R(I_{m}-AE_{B}A^{†})\cap R(AE_{B}A^{†})=\{0\}$ *and/or* $R\left({\left(I_{m}-AE_{B}A^{†}\right)}^{⁎}\right)\cap R\left({\left(AE_{B}A^{†}\right)}^{⁎}\right)=\{0\}$*.*
〈137〉 $R(I_{p}-B^{†}F_{A}B)\cap R(B^{†}F_{A}B)=\{0\}$ *and/or* $R\left({\left(I_{p}-B^{†}F_{A}B\right)}^{⁎}\right)\cap R\left({\left(B^{†}F_{A}B\right)}^{⁎}\right)=\{0\}$*.*
〈138〉 $C^{n}=R(I_{n}-A^{†}ABB^{†})⊕R(A^{†}ABB^{†})$ *and/or* $C^{n}=R(I_{n}-BB^{†}A^{†}A)⊕R(BB^{†}A^{†}A)$*.*
〈139〉 $C^{n}=R(I_{n}-F_{A}BB^{†})⊕R(F_{A}BB^{†})$ *and/or* $C^{n}=R(I_{n}-BB^{†}F_{A})⊕R(BB^{†}F_{A})$*.*
〈140〉 $C^{n}=R(I_{n}-A^{†}AE_{B})⊕R(A^{†}AE_{B})$ *and/or* $C^{n}=R(I_{n}-E_{B}A^{†}A)⊕R(E_{B}A^{†}A)$*.*
〈141〉 $C^{n}=R(I_{n}-F_{A}E_{B})⊕R(F_{A}E_{B})$ *and/or* $C^{n}=R(I_{n}-E_{B}F_{A})⊕R(E_{B}F_{A})$*.*
〈142〉 $C^{m}=R(I_{m}-ABB^{†}A^{†})⊕R(ABB^{†}A^{†})$ *and/or* $C^{m}=R\left({\left(I_{m}-ABB^{†}A^{†}\right)}^{⁎}\right)⊕R\left({\left(ABB^{†}A^{†}\right)}^{⁎}\right)$*.*
〈143〉 $C^{p}=R(I_{p}-B^{†}A^{†}AB)⊕R(B^{†}A^{†}AB)$ *and/or* $C^{p}=R\left({\left(I_{p}-B^{†}A^{†}AB\right)}^{⁎}\right)⊕R\left({\left(B^{†}A^{†}AB\right)}^{⁎}\right)=\{0\}$*.*
〈144〉 $C^{m}=R(I_{m}-AE_{B}A^{†})⊕R(AE_{B}A^{†})$ *and/or* $C^{m}=R\left({\left(I_{m}-AE_{B}A^{†}\right)}^{⁎}\right)⊕R\left({\left(AE_{B}A^{†}\right)}^{⁎}\right)$*.*
〈145〉 $C^{p}=R(I_{p}-B^{†}F_{A}B)⊕R(B^{†}F_{A}B)$ *and/or* $C^{p}=R\left({\left(I_{p}-B^{†}F_{A}B\right)}^{⁎}\right)⊕R\left({\left(B^{†}F_{A}B\right)}^{⁎}\right)$*.*
〈146〉 *One/all of the four facts:* $\dim\left(R(A^{⁎})\cap R(B)\right)=r(AB)$*,* $\dim\left(R(F_{A})\cap R(B)\right)=r(F_{A}B)$*,* $\dim\left(R(A^{⁎})\cap R(E_{B})\right)=r(AE_{B})$*,* $\dim\left(R(F_{A})\cap R(E_{B})\right)=r(F_{A}E_{B})$*.*
〈147〉 $R(BB^{†}A^{†}A)\subseteq R(A^{†}A)$ *and/or* $R(A^{†}ABB^{†})\subseteq R(BB^{†})$*.*
〈148〉 $R(BB^{†}F_{A})\subseteq R(F_{A})$ *and/or* $R(F_{A}BB^{†})\subseteq R(BB^{†})$*.*
〈149〉 $R(E_{B}A^{†}A)\subseteq R(A^{†}A)$ *and/or* $R(A^{†}AE_{B})\subseteq R(E_{B})$*.*
〈150〉 $R(E_{B}F_{A})\subseteq R(F_{A})$ *and/or* $R(F_{A}E_{B})\subseteq R(E_{B})$*.*
〈151〉 $R(A^{†}ABB^{†})=R(A^{†}A)\cap R(BB^{†})$ *and/or* $R(BB^{†}A^{†}A)=R(A^{†}A)\cap R(BB^{†})$*.*
〈152〉 $R(F_{A}BB^{†})=R(F_{A})\cap R(BB^{†})$ *and/or* $R(BB^{†}F_{A})=R(F_{A})\cap R(BB^{†})$*.*
〈153〉 $R(A^{†}AE_{B})=R(A^{†}A)\cap R(E_{B})$ *and/or* $R(E_{B}A^{†}A)=R(A^{†}A)\cap R(E_{B})$*.*
〈154〉 $R(F_{A}E_{B})=R(F_{A})\cap R(E_{B})$ *and/or* $R(E_{B}F_{A})=R(F_{A})\cap R(E_{B})$*.*
〈155〉 *One/all of the four facts:* $R(A^{†}ABB^{†})=R(BB^{†}A^{†}A)$*,* $R(A^{†}AE_{B})=R(E_{B}A^{†}A)$*,* $R(F_{A}BB^{†})=R(BB^{†}F_{A})$*,* $R(F_{A}E_{B})=R(E_{B}F_{A})$*.*
〈156〉 *One/all of the four facts:* $R(AB)\cap R(AE_{B})=\{0\}$*,* $R(F_{A}B)\cap R(F_{A}E_{B})=\{0\}$*,* $R(B^{⁎}A^{⁎})\cap R(B^{⁎}F_{A})=\{0\}$*,* $R(E_{B}A^{⁎})\cap R(E_{B}F_{A})=\{0\}$*.*
〈157〉 *One/all of the four facts:* $R(A)=R(AB)⊕R(AE_{B})$*,* $N(A)=R(F_{A}B)⊕R(F_{A}E_{B})$*,* $R(B^{⁎})=R(B^{⁎}A^{⁎})⊕R(B^{⁎}F_{A})$*,* $N(B^{⁎})=R(E_{B}A^{⁎})⊕R(E_{B}F_{A})$*.*
〈158〉 $R(A^{†}A-BB^{†})\cap R(A^{†}A+BB^{†}-I_{n})=\{0\}$*.*
〈159〉 $\left(R(A^{⁎})+R(B)\right)\cap \left(R(A^{⁎})+R{\left(B\right)}^{⊥}\right)\cap \left(R{\left(A^{⁎}\right)}^{⊥}+R(B)\right)\cap \left(R{\left(A^{⁎}\right)}^{⊥}+R{\left(B\right)}^{⊥}\right)=\{0\}$*.*
〈160〉 $\left(R(A^{⁎})\cap R(B)\right)⊕\left(R(A^{⁎})\cap R{\left(B\right)}^{⊥}\right)⊕\left(R{\left(A^{⁎}\right)}^{⊥}\cap R(B)\right)⊕\left(R{\left(A^{⁎}\right)}^{⊥}\cap R{\left(B\right)}^{⊥}\right)=C^{n}$*.*
〈161〉 $P_{R(A^{⁎})\cap R(B)}=A^{†}ABB^{†}$ *and/or* $P_{R(B)\cap R(A^{⁎})}=BB^{†}A^{†}A$*.*
〈162〉 *The matrix equations* $A^{⁎}X=BB^{†}A^{⁎}$ *and/or* $BY=A^{†}AB$ *are solvable.*
〈163〉 *The matrix equations* $F_{A}X=BB^{†}F_{A}$ *and/or* $BY=F_{A}B$ *are solvable.*
〈164〉 *The matrix equations* $A^{⁎}X=E_{B}A^{⁎}$ *and/or* $E_{B}Y=A^{†}AE_{B}$ *are solvable.*
〈165〉 *The matrix equations* $F_{A}X=E_{B}F_{A}$ *and/or* $E_{B}Y=F_{A}E_{B}$ *are solvable.*

Proof Setting all sides of (8.3)–(8.6) equal to zero leads to the equivalences of 〈1〉–〈48〉, 〈87〉, and 〈116〉.

Setting all sides of (8.5) and (8.7) equal to zero leads to the equivalences of 〈114〉, 〈115〉, and 〈116〉.

By (4.4),

(9.2) $$r(F_{A})=n-r(A),\;\;r(F_{A}B)=r[A^{⁎},\;B]-r(A^{⁎}),$$

(9.3) $$r(E_{B})=n-r(B),\;\;r(AE_{B})=r[A^{⁎},\;B]-r(B),$$

(9.4) $$r[F_{A},\;B]=r(F_{A})+r(A^{†}AB)=n-r(A)+r(AB),$$

(9.5) $$r[A^{⁎},\;E_{B}]=r(ABB^{†})+r(E_{B})=n-r(B)+r(AB),$$

(9.6) $$r[F_{A},\;E_{B}]=r(F_{A})+r(A^{†}AE_{B})=n-r(A)-r(B)-r[A^{⁎},\;B],$$

(9.7) $$r(F_{A}E_{B})=r[A^{⁎},\;E_{B}]-r(A^{⁎})=n-r(A)-r(B)+r(AB).$$

Substituting (9.2)–(9.7) into the three rank equalities in 〈117〉–〈119〉 and simplifying lead to the equivalences of 〈116〉–〈119〉.

Applying (4.13) to $I_{n}-A^{†}ABB^{†}$, $I_{m}-ABB^{†}A^{†}$, and $I_{p}-B^{†}A^{†}AB$, and simplifying by (4.4) and (4.5) lead to

(9.8) $$r(I_{n}-A^{†}ABB^{†})=r(I_{n}-BB^{†}A^{†}A)=r\left[\begin{array}{cc} AA^{⁎} & ABB^{†}\\ A^{⁎} & I_{n} \end{array}\right]-r(A)=r\left[\begin{array}{cc} AA^{⁎}-ABB^{†}A^{⁎} & 0\\ 0 & I_{n} \end{array}\right]-r(A)=n+r(AA^{⁎}-ABB^{†}A^{⁎})-r(A)=n+r\left[\begin{array}{cc} B^{⁎}B & B^{⁎}A^{⁎}\\ AB & AA^{⁎} \end{array}\right]-r(A)-r(B)=n+r({\left[A,\;B^{⁎}\right]}^{⁎}[A,\;B^{⁎}])-r(A)-r(B)=n+r(N)-r(A)-r(B),$$

(9.9) $$r(I_{m}-ABB^{†}A^{†})=r\left[\begin{array}{cc} B^{⁎}B & B^{⁎}A^{†}\\ AB & I_{m} \end{array}\right]-r(B)=r\left[\begin{array}{cc} B^{⁎}B-B^{⁎}A^{†}AB & 0\\ 0 & I_{m} \end{array}\right]-r(B)=r(B^{⁎}F_{A}B)-r(B)+m=r(F_{A}B)-r(B)+m=r(N)-r(A)-r(B)+m,$$

(9.10) $$r(I_{p}-B^{†}A^{†}AB)=r\left[\begin{array}{cc} AA^{⁎} & AB\\ B^{†}A^{⁎} & I_{p} \end{array}\right]-r(A)=r\left[\begin{array}{cc} AA^{⁎}-ABB^{†}A^{⁎} & 0\\ 0 & I_{p} \end{array}\right]-r(A)=r(AE_{B}A^{⁎})-r(A)+p=r(E_{B}A^{⁎})-r(A)+p=r(N)-r(A)-r(B)+p.$$

Combining (9.8)–(9.10) yields

(9.11) $$r(I_{n}-A^{†}ABB^{†})-n+r(A^{†}ABB^{†})=r(I_{n}-BB^{†}A^{†}A)-n+r(BB^{†}A^{†}A)=r(N)-r(A)-r(B)+r(AB),$$

(9.12) $$r(I_{m}-ABB^{†}A^{†})-m+r(ABB^{†}A^{†})=r(N)-r(A)-r(B)+r(AB),$$

(9.13) $$r(I_{p}-B^{†}A^{†}AB)-p+r(B^{†}A^{†}AB)=r(N)-r(A)-r(B)+r(AB).$$

Setting all sides of (9.11)–(9.13) equal to zero leads to the equivalences of 〈120〉, 〈121〉, and 〈116〉. Replacing $A^{†}A$ with $F_{A}$ and $BB^{†}$ with $E_{B}$ respectively in 〈120〉 and 〈121〉 leads to the equivalences of 〈122〉–〈125〉 with 〈116〉–〈118〉.

Applying (8.3) to $B^{†}F_{A}E_{B}A^{†}=B^{†}A^{†}-B^{†}A^{†}ABB^{†}A^{†}$ and simplifying by Lemma 8.6 yield

(9.14) $$r(B^{†}F_{A}E_{B}A^{†})=r(B^{†}A^{†}-B^{†}A^{†}ABB^{†}A^{†})=r[{\left(B^{†}\right)}^{⁎},\;A^{†}]+r(B^{†}A^{†})-r(B^{†})-r(A^{†})=r(N)+r(AB)-r(B)-r(A).$$

Setting both sides of (9.14) equal to zero leads to the equivalence of 〈88〉 and 〈116〉.

Replacing *A* and *B* in (9.14) with ${\left(A^{†}\right)}^{⁎}$, $A^{⁎}A$, ${\left(A^{⁎}A\right)}^{1/2}$, $AA^{⁎}A$, $A^{†}A$, ${\left(B^{†}\right)}^{⁎}$, $BB^{⁎}$, ${\left(BB^{⁎}\right)}^{1/2}$, $BB^{⁎}B$, and $BB^{†}$, respectively, and simplifying by Lemma 8.6 lead to the following rank formulas

$$r\left({\left(A^{†}\right)}^{⁎}B-{\left(A^{†}\right)}^{⁎}BB^{†}A^{⁎}{\left(A^{†}\right)}^{⁎}B\right)=r\left(A{\left(B^{†}\right)}^{⁎}-A{\left(B^{†}\right)}^{⁎}B^{⁎}A^{†}A{\left(B^{†}\right)}^{⁎}\right)=r\left((A^{⁎}A)B-(A^{⁎}A)BB^{†}{\left(A^{⁎}A\right)}^{†}(A^{⁎}A)B\right)=r\left(A(BB^{⁎})-A(BB^{⁎}){\left(BB^{⁎}\right)}^{†}A^{†}A(BB^{⁎})\right)=r\left((A^{⁎}A)(BB^{⁎})-(A^{⁎}A)(BB^{⁎}){\left(BB^{⁎}\right)}^{†}{\left(A^{⁎}A\right)}^{†}(A^{⁎}A)(BB^{⁎})\right)=r\left({\left(A^{⁎}A\right)}^{1/2}{\left(BB^{⁎}\right)}^{1/2}-{\left(A^{⁎}A\right)}^{1/2}{\left(BB^{⁎}\right)}^{1/2}{\left({\left(BB^{⁎}\right)}^{1/2}\right)}^{†}{\left({\left(A^{⁎}A\right)}^{1/2}\right)}^{†}{\left(A^{⁎}A\right)}^{1/2}{\left(BB^{⁎}\right)}^{1/2}\right)=r\left((AA^{⁎}A)(BB^{⁎}B)-(AA^{⁎}A)(BB^{⁎}B){\left(BB^{⁎}B\right)}^{†}{\left(AA^{⁎}A\right)}^{†}(AA^{⁎}A)(BB^{⁎}B)\right)$$

(9.15) $$=r\left(A^{†}AB-(A^{†}AB)B^{†}A^{†}A(A^{†}AB)\right)=r\left(ABB^{†}-(ABB^{†})BB^{†}A^{†}(ABB^{†})\right)=r\left(A^{†}ABB^{†}-(A^{†}ABB^{†})BB^{†}A^{†}A(A^{†}ABB^{†})\right)=r(N)+r(AB)-r(A)-r(B).$$

Setting all sides of (9.15) equal to zero leads to the equivalences of 〈77〉–〈83〉 and 〈116〉.

Replacing *A* and *B* in (9.14) with $AA^{†}$, $F_{A}$, $BB^{†}$, and $E_{B}$ yields

(9.16) $$r\left(F_{A}BB^{†}-(F_{A}BB^{†})BB^{†}F_{A}(F_{A}BB^{†})\right)=r[F_{A},\;B]-r(F_{A})-r(B)+r(F_{A}B),$$

(9.17) $$r\left(A^{†}AE_{B}-(A^{†}AE_{B})E_{B}A^{†}A(A^{†}AE_{B})\right)=r[A,\;E_{B}]-r(A)-r(E_{B})+r(AE_{B}),$$

(9.18) $$r\left(F_{A}E_{B}-(F_{A}E_{B})E_{B}F_{A}(F_{A}E_{B})\right)=r[F_{A},\;E_{B}]-r(F_{A})-r(E_{B})+r(F_{A}AE_{B}).$$

Setting all sides of (9.16)–(9.18) equal to zero leads to the equivalences of 〈84〉–〈86〉 and 〈117〉–〈119〉.

Applying (4.4) to $ABB^{†}F_{A}$ and $E_{B}A^{†}AB$ and simplifying by Lemma 4.3(c) and (d) yield

(9.19) $$r(ABB^{†}F_{A})=r\left[\begin{array}{c} ABB^{†}\\ A \end{array}\right]-r(A)=r\left[\begin{array}{c} ABB^{†}\\ A-ABB^{†} \end{array}\right]-r(A)=r(ABB^{†})+r(A-ABB^{†})-r(A)=r(N)-r(A)-r(B)+r(AB),$$

(9.20) $$r(E_{B}A^{†}AB)=r[B,\;A^{†}AB]-r(B)=r[B-A^{†}AB,\;A^{†}AB]-r(B)=r(B-A^{†}AB)+r(A^{†}AB)-r(B)=r(N)-r(A)-r(B)+r(AB).$$

Setting all sides of (9.19) and (9.20) equal to zero leads to the equivalence of 〈89〉 and 〈116〉. Replacing $A^{†}A$ and $BB^{†}$ with $F_{A}$ and $E_{B}$ in 〈89〉 respectively leads to the equivalences of 〈90〉 and 〈117〉 and 〈118〉.

Applying (4.27) to $A^{†}ABB^{†}-BB^{†}A^{†}A$ and simplifying yield

(9.21) $$r\left(A^{†}ABB^{†}-BB^{†}A^{†}A\right)=r\left(F_{A}BB^{†}-BB^{†}F_{A}\right)=r\left(A^{†}AE_{B}-E_{B}A^{†}A\right)=r\left(F_{A}E_{B}-E_{B}F_{A}\right)=2r[A^{†}A,\;BB^{†}]-2r(A^{†}A)-2r(BB^{†})+2r(A^{†}ABB^{†})=2r(N)-2r(A)-2r(B)+2r(AB).$$

Setting both sides of (9.21) equal to zero leads to the equivalence of 〈91〉 and 〈116〉.

Applying (4.21) to ${\left(A^{†}ABB^{†}\right)}^{2}-A^{†}ABB^{†}$, ${\left(BB^{†}A^{†}A\right)}^{2}-BB^{†}A^{†}A$, ${\left(ABB^{†}A^{†}\right)}^{2}-ABB^{†}A^{†}$, ${\left(B^{†}A^{†}AB\right)}^{2}-B^{†}A^{†}AB$, and simplifying by (9.8)–(9.10) yield

(9.22) $$r\left({\left(A^{†}ABB^{†}\right)}^{2}-A^{†}ABB^{†}\right)=r(I_{n}-A^{†}ABB^{†})+r(A^{†}ABB^{†})-n=r(N)-r(A)-r(B)+r(AB),$$

(9.23) $$r\left({\left(BB^{†}A^{†}A\right)}^{2}-BB^{†}A^{†}A\right)=r(I_{n}-BB^{†}A^{†}A)+r(BB^{†}A^{†}A)-n=r(N)-r(A)-r(B)+r(AB),$$

(9.24) $$r\left({\left(ABB^{†}A^{†}\right)}^{2}-ABB^{†}A^{†}\right)=r(I_{m}-ABB^{†}A^{†})+r(ABB^{†}A^{†})-m=r(N)-r(A)-r(B)+r(AB),$$

(9.25) $$r\left({\left(B^{†}A^{†}AB\right)}^{2}-B^{†}A^{†}AB\right)=r(I_{p}-B^{†}A^{†}AB)+r(B^{†}A^{†}AB)-p=r(N)-r(A)-r(B)+r(AB).$$

Setting all sides of (9.22)–(9.25) equal to zero leads to the equivalences of 〈92〉, 〈93〉, and 〈116〉. Replacing $A^{†}A$ with $F_{A}$ in 〈92〉 leads to the equivalence of 〈94〉 and 〈117〉. Replacing $BB^{†}$ with $E_{B}$ in 〈92〉 leads to the equivalence of 〈95〉 and 〈118〉. Replacing $A^{†}A$ and $BB^{†}$ with $F_{A}$ and $E_{B}$ in 〈92〉 leads to the equivalence of 〈96〉 and 〈119〉. Replacing $A^{†}A$ and $BB^{†}$ with $F_{A}$ and $E_{B}$ in 〈93〉 respectively leads to the equivalences of 〈97〉 with 〈117〉 and 〈118〉.

Applying (4.16) to ${\left(A^{†}ABB^{†}\right)}^{†}-BB^{†}A^{†}A$ and simplifying by (4.21) and (9.8), we obtain

(9.26) $$r\left({\left(A^{†}ABB^{†}\right)}^{†}-BB^{†}A^{†}A\right)=r\left({\left(A^{†}ABB^{†}\right)}^{†}-{\left(A^{†}ABB^{†}\right)}^{⁎}\right)=r\left((A^{†}ABB^{†})-(A^{†}ABB^{†}){\left(A^{†}ABB^{†}\right)}^{⁎}(A^{†}ABB^{†})\right)=r\left((A^{†}ABB^{†})-{\left(A^{†}ABB^{†}\right)}^{2}\right)=r(A^{†}ABB^{†})+r(I_{n}-A^{†}ABB^{†})-n=r(N)+r(AB)-r(A)-r(B).$$

Setting all sides of (9.26) equal to zero leads to the equivalence of 〈98〉 and 〈116〉. Replacing $A^{†}A$ with $F_{A}$ in 〈98〉 leads to the equivalence of 〈99〉 and 〈117〉. Replacing $BB^{†}$ with $E_{B}$ in 〈98〉 leads to the equivalence of 〈100〉 and 〈118〉. Replacing $A^{†}A$ and $BB^{†}$ with $F_{A}$ and $E_{B}$ in 〈98〉 leads to the equivalence of 〈101〉 and 〈119〉.

Applying (4.16) to the difference ${\left(A^{†}A-BB^{†}\right)}^{†}-(A^{†}A-BB^{†})$ and simplifying by (4.22)–(4.25) yield

(9.27) $$r\left({\left(A^{†}A-BB^{†}\right)}^{†}-(A^{†}A-BB^{†})\right)=r\left((A^{†}A-BB^{†})-{\left(A^{†}A-BB^{†}\right)}^{3}\right)=r(A^{†}A-BB^{†})+r(I_{n}-A^{†}A+BB^{†})+r(I_{n}+A^{†}A-BB^{†})-2n=2r[A^{†}A,\;BB^{†}]-r(A^{†}A)-r(BB^{†})+r[I_{n}-A^{†}A,\;BB^{†}]+r[I_{n}-BB^{†},\;A^{†}A]-2n=2r(N)-r(A)-r(B)+n-r(A)+r(AB)+n-r(B)+r(AB)-2n=2r(N)-2r(A)-2r(B)+2r(AB).$$

Setting both sides of (9.27) equal to zero leads to the equivalence of 〈102〉 and 〈116〉. Replacing $A^{†}A$ with $F_{A}$ in (9.27) yields

(9.28) $$r\left({\left(I_{n}-A^{†}A-BB^{†}\right)}^{†}-(I_{n}-A^{†}A-BB^{†})\right)=r[F_{A},\;B]-r(F_{A})-r(B)+r(F_{A}B).$$

Setting both sides of (9.28) equal to zero leads to the equivalence of 〈103〉 and 〈117〉.

It can be deduced from (4.13) that

(9.29) $$r(2I_{m}-ABB^{†}A^{†})=r\left[\begin{array}{cc} B^{⁎}B\; & B^{⁎}A^{†}\\ AB\; & 2I_{m} \end{array}\right]-r(B)=r\left[\begin{array}{cc} B^{⁎}B-B^{⁎}A^{†}AB/2\; & 0\\ 0\; & 2I_{m} \end{array}\right]-r(B)=m+r[B^{⁎}(I_{n}-A^{†}A/2)B)-r(B)=m+r(B)-r(B)=m,$$

(9.30) $$r(2I_{p}-B^{†}A^{†}AB)=r\left[\begin{array}{cc} AA^{⁎}\; & AB\\ B^{†}A^{⁎}\; & 2I_{p} \end{array}\right]-r(A)=r\left[\begin{array}{cc} AA^{⁎}-ABB^{†}A^{⁎}/2\; & 0\\ 0\; & 2I_{p} \end{array}\right]-r(A)=p+r\left(A(I_{p}-BB^{†}/2)A^{⁎}\right)-r(A)=p+r(A)-r(A)=p,$$

(9.31) $$r(2I_{n}-A^{†}ABB^{†})=r(2I_{n}-BB^{†}A^{†}A)=r\left[\begin{array}{cc} B^{⁎}B\; & B^{⁎}A^{†}A\\ B\; & 2I_{n} \end{array}\right]-r(B)=r\left[\begin{array}{cc} B^{⁎}B-B^{⁎}A^{†}AB/2\; & 0\\ 0\; & 2I_{n} \end{array}\right]-r(B)=r\left(B^{⁎}(I_{n}-A^{†}A/2)B\right)+n-r(B)=r(B)+n-r(B)=n.$$

By (4.16), (9.8)–(9.10), and (9.29)–(9.31), the following formulas

(9.32) $$r\left({\left(I_{m}-ABB^{†}A^{†}\right)}^{†}-(I_{m}-ABB^{†}A^{†})\right)=r\left((I_{m}-ABB^{†}A^{†})-{\left(I_{m}-ABB^{†}A^{†}\right)}^{3}\right)=r(I_{m}-ABB^{†}A^{†})+r(2I_{m}-ABB^{†}A^{†})+r(ABB^{†}A^{†})-2m=r(N)-r(A)-r(B)+2m+r(AB)-2m=r(N)-r(A)-r(B)+r(AB),$$

(9.33) $$r\left({\left(I_{p}-B^{†}A^{†}AB\right)}^{†}-(I_{p}-B^{†}A^{†}AB)\right)=r\left((I_{p}-B^{†}A^{†}AB)-{\left(I_{p}-B^{†}A^{†}AB\right)}^{3}\right)=r(I_{p}-B^{†}A^{†}AB)+r(2I_{p}-B^{†}A^{†}AB)+r(B^{†}A^{†}AB)-2p=r(N)-r(A)-r(B)+2p+r(AB)-2p=r(N)-r(A)-r(B)+r(AB),$$

(9.34) $$r\left({\left(I_{n}-A^{†}ABB^{†}\right)}^{†}-(I_{n}-A^{†}ABB^{†})\right)=r\left((I_{n}-A^{†}ABB^{†})-{\left(I_{n}-A^{†}ABB^{†}\right)}^{3}\right)=r(I_{n}-A^{†}ABB^{†})+r(2I_{n}-A^{†}ABB^{†})+r(A^{†}ABB^{†})-2n=r(N)-r(A)-r(B)+2n+r(AB)-2n=r(N)-r(A)-r(B)+r(AB)$$

hold. Setting all sides of (9.32)–(9.34) equal to zero leads to the equivalences of 〈104〉, 〈105〉, and 〈116〉. Replacing $A^{†}A$ with $F_{A}$ and $BB^{†}$ with $E_{B}$ in 〈104〉 and 〈105〉 respectively leads to the equivalences of 〈106〉–〈109〉 and 〈117〉–〈119〉.

The following rank equalities

$$r\left(NN^{†}-A^{†}A-BB^{†}+A^{†}ABB^{†}\right)=r\left(NN^{†}-A^{†}A-BB^{†}+BB^{†}A^{†}A\right)=r(N)-r(A)-r(B)+r(AB)$$

were proved in [110]. Setting the three sides of these two identities equal to zero leads to the equivalence of 〈110〉 and 〈116〉. Replacing $A^{⁎}$ and *B* with $F_{A}$ and $E_{B}$ in 〈110〉 respectively leads to the equivalences of 〈111〉–〈113〉 and 〈117〉–〈119〉.

It follows from (4.4) and (4.5) that

$$r(A^{†}A-A^{†}ABB^{†})=r(E_{B}A^{⁎})=r(N)-r(B),r(BB^{†}-A^{†}ABB^{†})=r(F_{A}B)=r(N)-r(A).$$

Thus the two rank equalities in 〈126〉 are equivalent to 〈116〉. Replacing $A^{†}A$ and $BB^{†}$ with $F_{A}$ and $E_{B}$ in 〈126〉 respectively leads to the equivalences of 〈127〉–〈129〉 and 〈117〉–〈119〉.

The equivalences of 〈120〉–〈125〉 and 〈130〉–〈137〉 respectively follow from Lemma 4.3(f). The equivalences of 〈130〉–〈137〉 and 〈138〉–〈145〉 are obvious.

By (4.11),

$$\dim\left(R(A^{⁎})\cap R(B)\right)=r(A)+r(B)-r[A^{⁎},\;B],\dim\left(R(F_{A})\cap R(B)\right)=r(F_{A})+r(B)-r[F_{A},\;B],\dim\left(R(A^{⁎})\cap R(E_{B})\right)=r(A)+r(E_{B})-r[A^{⁎},\;E_{B}],\dim\left(R(F_{A})\cap R(E_{B})\right)=r(F_{A})+r(E_{B})-r[F_{A},\;E_{B}].$$

Hence the equivalences of 〈146〉 and 〈116〉–〈119〉 follow.

Applying Lemma 4.3(a) and (b) to 〈89〉 leads to the equivalence of 〈89〉 and 〈149〉. Replacing $A^{†}A$ and $BB^{†}$ with $F_{A}$ and $E_{B}$ in 〈149〉 respectively leads to the equivalences of 〈148〉–〈150〉 and 〈117〉–〈119〉.

It follows first from 〈147〉 that

(9.35) $$R(A^{†}ABB^{†})\subseteq R(A^{†}A)\cap R(BB^{†})$$

and/or

(9.36) $$R(BB^{†}A^{†}A)\subseteq R(A^{†}A)\cap R(BB^{†}),$$

and from 〈116〉 that

(9.37) $$\dim\left(R(A^{†}A)\cap R(BB^{†})\right)=r(A^{†}A)+r(BB^{†})-r[A^{†}A,\;BB^{†}]=r(A^{†}ABB^{†})=r(BB^{†}A^{†}A).$$

Applying (8.10) to (9.35), (9.36), and (9.37) leads to the range identities in 〈151〉. Conversely, 〈151〉 obviously implies 〈147〉. Replacing $A^{†}A$ and $BB^{†}$ with $F_{A}$ and $E_{B}$ in 〈151〉 respectively leads to the equivalences of 〈152〉–〈154〉 and 〈117〉–〈119〉.

By (4.28),

(9.38) $$r(A^{†}ABB^{†}-BB^{†}A^{†}A)=2r[A^{†}ABB^{†},\;BB^{†}A^{†}A]-2r(BB^{†}A^{†}A).$$

Setting both sides of (9.38) equal to zero leads to the equivalence of the first range equality in 〈91〉 and 〈155〉. Replacing $A^{†}A$ and $BB^{†}$ with $F_{A}$ and $E_{B}$ in the first range equality of 〈155〉 respectively leads to the equivalences of the second, third, and fourth range equalities in 〈155〉 and those in 〈117〉–〈119〉.

By (4.11), the dimension of the intersection $R(AB)\cap R(AE_{B})$ is reduced to

(9.39) $$\dim\left(R(AB)\cap R(AE_{B})\right)=r(AB)+r(AE_{B})-r[AB,\;AE_{B}]=r(AB)+r(N)-r(B)-r[AB,\;A]=r(N)-r(A)-r(B)+r(AB).$$

Setting both sides of (9.39) equal to zero leads to the equivalence of the first range equality in 〈156〉 and 〈116〉. The equivalences of the other three range equalities in 〈128〉 with 〈116〉–〈119〉 can be shown similarly.

The equivalence of 〈156〉 and 〈157〉 follows from the definition of direct sum of linear subspaces.

By (4.11) and (4.26),

(9.40) $$\dim\left(R(A^{†}A-BB^{†})\cap R(A^{†}A+BB^{†}-I_{n})\right)=r(A^{†}A-BB^{†})+r(A^{†}A+BB^{†}-I_{n})-r[A^{†}A-BB^{†},\;A^{†}A+BB^{†}-I_{n}]=r(A^{†}A-BB^{†})+r(A^{†}A+BB^{†}-I_{n})-r[A^{†}A-BB^{†},\;2A^{†}A-I_{n}]=r(A^{†}A-BB^{†})+r(A^{†}A+BB^{†}-I_{n})-r[A^{†}A-BB^{†},\;I_{n}]=r(A^{†}A-BB^{†})+r(A^{†}A+BB^{†}-I_{n})-n=r(A^{†}ABB^{†}-BB^{†}A^{†}A).$$

Setting all sides of (9.40) equal to zero leads to the equivalence of the first equality in 〈91〉 and 〈158〉.

The equivalences of 〈91〉 and 〈159〉–〈161〉 follow from Lemma 4.14(a)–(e).

Applying (5.2) to 〈147〉–〈150〉 leads to the equivalences of 〈147〉–〈150〉 and 〈162〉–〈165〉. □

The commutativity of the product of two orthogonal projectors is a quite special topic that attracts much attention in matrix theory, and has important applications in many disciplines of mathematics. A rich variety of results regarding the commutativity problem were scattered in the literature (cf. [2], [3], [4], [5], [8], [9], [10], [11], [12], [16], [19], [37], [43], [61], [69], [70], [85], [111]).

Theorem 9.2 **[45]**

*Let* $A\in C^{m\times n}$ *and* $B\in C^{n\times p}$*, and denote* $N=[A^{⁎},\;B]$*. Then the following 29 statements are equivalent:*

$$\;\begin{array}{cccc} \langle 1\rangle \; & \{{\left(AB\right)}^{\left(1\right)}\}⊇\{B^{\left(1,2,4\right)}A^{\left(1,2,3\right)}\}.\; & \;\langle 2\rangle \; & \{{\left(AB\right)}^{\left(1\right)}\}⊇\{B^{\left(1,2,4\right)}A^{\left(1,3\right)}\}.\\ \langle 3\rangle \; & \{{\left(AB\right)}^{\left(1\right)}\}⊇\{B^{\left(1,2,4\right)}A^{\left(1,2\right)}\}.\; & \langle 4\rangle \; & \{{\left(AB\right)}^{\left(1\right)}\}⊇\{B^{\left(1,2,4\right)}A^{\left(1\right)}\}.\\ \langle 5\rangle \; & \{{\left(AB\right)}^{\left(1\right)}\}⊇\{B^{\left(1,4\right)}A^{\left(1,2,3\right)}\}.\; & \langle 6\rangle \; & \{{\left(AB\right)}^{\left(1\right)}\}⊇\{B^{\left(1,4\right)}A^{\left(1,3\right)}\}.\\ \langle 7\rangle \; & \{{\left(AB\right)}^{\left(1\right)}\}⊇\{B^{\left(1,4\right)}A^{\left(1,2\right)}\}.\; & \langle 8\rangle \; & \{{\left(AB\right)}^{\left(1\right)}\}⊇\{B^{\left(1,4\right)}A^{\left(1\right)}\}.\\ \langle 9\rangle \; & \{{\left(AB\right)}^{\left(1\right)}\}⊇\{B^{\left(1,2\right)}A^{\left(1,2,3\right)}\}.\; & \langle 10\rangle \; & \{{\left(AB\right)}^{\left(1\right)}\}⊇\{B^{\left(1,2\right)}A^{\left(1,3\right)}\}.\\ \langle 11\rangle \; & \{{\left(AB\right)}^{\left(1\right)}\}⊇\{B^{\left(1,2\right)}A^{\left(1,2\right)}\}.\; & \langle 12\rangle \; & \{{\left(AB\right)}^{\left(1\right)}\}⊇\{B^{\left(1,2\right)}A^{\left(1\right)}\}.\\ \langle 13\rangle \; & \{{\left(AB\right)}^{\left(1\right)}\}⊇\{B^{\left(1\right)}A^{\left(1,2,3\right)}\}.\; & \langle 14\rangle \; & \{{\left(AB\right)}^{\left(1\right)}\}⊇\{B^{\left(1\right)}A^{\left(1,3\right)}\}.\\ \langle 15\rangle \; & \{{\left(AB\right)}^{\left(1\right)}\}⊇\{B^{\left(1\right)}A^{\left(1,2\right)}\}.\; & \langle 16\rangle \; & \{{\left(AB\right)}^{\left(1\right)}\}⊇\{B^{\left(1\right)}A^{\left(1\right)}\}.\\ \langle 17\rangle \; & \{AB{\left(AB\right)}^{\left(1\right)}\}⊇\{ABB^{\left(1\right)}A^{\left(1,2,3\right)}\}.\; & \langle 18\rangle \; & \{AB{\left(AB\right)}^{\left(1\right)}\}⊇\{ABB^{\left(1\right)}A^{\left(1,3\right)}\}.\\ \langle 19\rangle \; & \{AB{\left(AB\right)}^{\left(1\right)}\}⊇\{ABB^{\left(1\right)}A^{\left(1,2\right)}\}.\; & \langle 20\rangle \; & \{AB{\left(AB\right)}^{\left(1\right)}\}⊇\{ABB^{\left(1\right)}A^{\left(1\right)}\}.\\ \langle 21\rangle \; & \{{\left(AB\right)}^{\left(1\right)}AB\}⊇\{B^{\left(1,2,4\right)}A^{\left(1\right)}AB\}.\; & \;\langle 22\rangle \; & \{{\left(AB\right)}^{\left(1\right)}AB\}⊇\{B^{\left(1,4\right)}A^{\left(1\right)}AB\}.\\ \langle 23\rangle \; & \{{\left(AB\right)}^{\left(1\right)}AB\}⊇\{B^{\left(1,2\right)}A^{\left(1\right)}AB\}.\; & \;\langle 24\rangle \; & \{{\left(AB\right)}^{\left(1\right)}AB\}⊇\{B^{\left(1\right)}A^{\left(1\right)}AB\}.\\ \langle 25\rangle \; & \{B{\left(AB\right)}^{\left(1\right)}A\}⊇\{BB^{\left(1\right)}A^{\left(1\right)}A\}.\;\\ \langle 26\rangle \; & AB=0\;\text{or}\;r(AB)=r(A)+r(B)-n.\;\\ \langle 27\rangle \; & AB=0\;\text{or}\;F_{A}E_{B}=0.\;\\ \langle 28\rangle \; & N(A)⊇R(B)\;\text{or}\;N(A)\subseteq R(B).\;\\ \langle 29\rangle \; & R(A^{⁎})⊇N(B^{⁎})\;\text{or}\;R(A^{⁎})\subseteq N(B^{⁎}).\; \end{array}$$

Proof The equivalences of 〈1〉–〈16〉 and 〈26〉–〈29〉 follow from (8.8). The equivalences of 〈1〉 and 〈17〉–〈25〉 follow from definitions and Lemma 3.1(f). □

The equivalences of Results 〈16〉 and 〈26〉–〈29〉 in Theorem 9.2 were well known (cf. [73], [74], [114] among others). Subsequently, we characterize the set inclusions in (1.10) for {1,2}-, {1,3}-, and {1,4}-generalized inverses of *AB*.

Theorem 9.3 *Let* $A\in C^{m\times n}$ *and* $B\in C^{n\times p}$ *be given, and denote* M=AB*,* $N=[A^{⁎},\;B]$*, and* t=m+p+r(M)−r(A)−r(B)*. Then the following results hold.*

(a) *The following 6 statements are equivalent:*
  $$\;\begin{array}{cccc} \langle 1\rangle \; & \{{\left(AB\right)}^{\left(1,2\right)}\}∋B^{†}A^{†}.\; & \;\langle 2\rangle \; & \{{\left(AB\right)}^{\left(1,2\right)}\}⊇\{B^{†}A^{\left(1,2,4\right)}\}.\\ \langle 3\rangle \; & \{{\left(AB\right)}^{\left(1,2\right)}\}⊇\{B^{\left(1,2,3\right)}A^{†}\}.\; & \;\langle 4\rangle \; & \{{\left(AB\right)}^{\left(1,2\right)}\}⊇\{B^{\left(1,2,3\right)}A^{\left(1,2,4\right)}\}.\\ \langle 5\rangle \; & ABB^{†}A^{†}AB=AB\;\text{and/or}\;B^{†}A^{†}ABB^{†}A^{†}=B^{†}A^{†}.\;\\ \langle 6\rangle \; & r(M)=r(A)+r(B)-r(N).\; \end{array}$$
(b) *The following 9 statements are equivalent:*
  $$\;\begin{array}{cccc} \langle 1\rangle \; & \{{\left(AB\right)}^{\left(1,2\right)}\}⊇\{B^{†}A^{\left(1,2,3\right)}\}.\; & \langle 2\rangle \; & \{{\left(AB\right)}^{\left(1,2\right)}\}⊇\{B^{†}A^{\left(1,2\right)}\}.\\ \langle 3\rangle \; & \{{\left(AB\right)}^{\left(1,2\right)}\}⊇\{B^{\left(1,2,4\right)}A^{†}\}.\; & \langle 4\rangle \; & \{{\left(AB\right)}^{\left(1,2\right)}\}⊇\{B^{\left(1,2,4\right)}A^{\left(1,2,4\right)}\}.\\ \langle 5\rangle \; & \{{\left(AB\right)}^{\left(1,2\right)}\}⊇\{B^{\left(1,2,3\right)}A^{\left(1,2,3\right)}\}.\; & \langle 6\rangle \; & \{{\left(AB\right)}^{\left(1,2\right)}\}⊇\{B^{\left(1,2,3\right)}A^{\left(1,2\right)}\}.\\ \langle 7\rangle \; & \{{\left(AB\right)}^{\left(1,2\right)}\}⊇\{B^{\left(1,2\right)}A^{†}\}.\; & \langle 8\rangle \; & \{{\left(AB\right)}^{\left(1,2\right)}\}⊇\{B^{\left(1,2\right)}A^{\left(1,2,4\right)}\}.\\ \langle 9\rangle \; & R(A^{⁎})\subseteq R(B)\;\text{or}\;R(A^{⁎})⊇R(B).\; \end{array}$$
(c) *The following 9 statements are equivalent:*
  $$\;\begin{array}{cccc} \langle 1\rangle \; & \{{\left(AB\right)}^{\left(1,2\right)}\}⊇\{B^{\left(1,3,4\right)}A^{\left(1,2,3\right)}\}.\; & \;\langle 2\rangle \; & \left\{{\left(AB\right)}^{\left(1,2\right)}\}⊇\{B^{\left(1,3,4\right)}A^{\left(1,2\right)}\right\}\\ \langle 3\rangle \; & \{{\left(AB\right)}^{\left(1,2\right)}\}⊇\{B^{\left(1,4\right)}A^{†}\}.\; & \;\langle 4\rangle \; & \{{\left(AB\right)}^{\left(1,2\right)}\}⊇\{B^{\left(1,4\right)}A^{\left(1,2,4\right)}\}.\\ \langle 5\rangle \; & \{{\left(AB\right)}^{\left(1,2\right)}\}⊇\{B^{\left(1,3\right)}A^{\left(1,2,3\right)}\}.\; & \;\langle 6\rangle \; & \{{\left(AB\right)}^{\left(1,2\right)}\}⊇\{B^{\left(1,3\right)}A^{\left(1,2\right)}\}.\\ \langle 7\rangle \; & \{{\left(AB\right)}^{\left(1,2\right)}\}⊇\{B^{\left(1\right)}A^{†}\}.\; & \;\langle 8\rangle \; & \{{\left(AB\right)}^{\left(1,2\right)}\}⊇\{B^{\left(1\right)}A^{\left(1,2,4\right)}\}.\\ \langle 9\rangle \; & R(A^{⁎})\subseteq R(B)\;\text{or}\;\{R(A^{⁎})⊇R(B)\;\text{and}\;r(B)=p\}.\; \end{array}$$
(d) *The following 9 statements are equivalent:*
  $$\;\begin{array}{cccc} \langle 1\rangle \; & \{{\left(AB\right)}^{\left(1,2\right)}\}⊇\{B^{†}A^{\left(1,3\right)}\}.\; & \;\langle 2\rangle \; & \{{\left(AB\right)}^{\left(1,2\right)}\}⊇\{B^{†}A^{\left(1\right)}\}.\\ \langle 3\rangle \; & \{{\left(AB\right)}^{\left(1,2\right)}\}⊇\{B^{\left(1,2,4\right)}A^{\left(1,3,4\right)}\}.\; & \;\langle 4\rangle \; & \{{\left(AB\right)}^{\left(1,2\right)}\}⊇\{B^{\left(1,2,4\right)}A^{\left(1,4\right)}\}.\\ \langle 5\rangle \; & \{{\left(AB\right)}^{\left(1,2\right)}\}⊇\{B^{\left(1,2,3\right)}A^{\left(1,3\right)}\}.\; & \;\langle 6\rangle \; & \{{\left(AB\right)}^{\left(1,2\right)}\}⊇\{B^{\left(1,2,3\right)}A^{\left(1\right)}\}.\\ \langle 7\rangle \; & \{{\left(AB\right)}^{\left(1,2\right)}\}⊇\{B^{\left(1,2\right)}A^{\left(1,3,4\right)}\}.\; & \;\langle 8\rangle \; & \{{\left(AB\right)}^{\left(1,2\right)}\}⊇\{B^{\left(1,2\right)}A^{\left(1,4\right)}\}.\\ \langle 9\rangle \; & R(A^{⁎})⊇R(B)\;\text{or}\;\{r(A)=m\;\text{and}\;R(A^{⁎})\subseteq R(B)\}.\; \end{array}$$
(e) *The following 5 statements are equivalent:*
  $$\;\begin{array}{cccc} \langle 1\rangle \; & \{{\left(AB\right)}^{\left(1,2\right)}\}⊇\{B^{\left(1,3,4\right)}A^{†}\}.\; & \;\langle 2\rangle \; & \{{\left(AB\right)}^{\left(1,2\right)}\}⊇\{B^{\left(1,3,4\right)}A^{\left(1,2,4\right)}\}.\\ \langle 3\rangle \; & \{{\left(AB\right)}^{\left(1,2\right)}\}⊇\{B^{\left(1,3\right)}A^{†}\}.\; & \;\langle 4\rangle \; & \{{\left(AB\right)}^{\left(1,2\right)}\}⊇\{B^{\left(1,3\right)}A^{\left(1,2,4\right)}\}.\\ \langle 5\rangle \; & R(A^{⁎})\subseteq R(B)\;\text{or}\;\{r(B)=p\;\text{and}\;r(M)=r(A)+r(B)-r(N)\}.\; \end{array}$$
(f) *The following 5 statements are equivalent:*
  $$\;\begin{array}{cccc} \langle 1\rangle \; & \{{\left(AB\right)}^{\left(1,2\right)}\}⊇\{B^{†}A^{\left(1,3,4\right)}\}.\; & \;\langle 2\rangle \; & \{{\left(AB\right)}^{\left(1,2\right)}\}⊇\{B^{†}A^{\left(1,4\right)}\}.\\ \langle 3\rangle \; & \{{\left(AB\right)}^{\left(1,2\right)}\}⊇\{B^{\left(1,2,3\right)}A^{\left(1,3,4\right)}\}.\; & \;\langle 4\rangle \; & \{{\left(AB\right)}^{\left(1,2\right)}\}⊇\{B^{\left(1,2,3\right)}A^{\left(1,4\right)}\}.\\ \langle 5\rangle \; & R(A^{⁎})⊇R(B)\;\text{or}\;\{r(A)=m\;\text{and}\;r(M)=r(A)+r(B)-r(N)\}.\; \end{array}$$
(g) *The following 9 statements are equivalent:*
  $$\;\begin{array}{cccc} \langle 1\rangle \; & \{{\left(AB\right)}^{\left(1,2\right)}\}⊇\{B^{\left(1,3,4\right)}A^{\left(1,3\right)}\}.\; & \;\langle 2\rangle \; & \{{\left(AB\right)}^{\left(1,2\right)}\}⊇\{B^{\left(1,3,4\right)}A^{\left(1\right)}\}.\\ \langle 3\rangle \; & \{{\left(AB\right)}^{\left(1,2\right)}\}⊇\{B^{\left(1,4\right)}A^{\left(1,3,4\right)}\}.\; & \;\langle 4\rangle \; & \{{\left(AB\right)}^{\left(1,2\right)}\}⊇\{B^{\left(1,4\right)}A^{\left(1,4\right)}\}.\\ \langle 5\rangle \; & \{{\left(AB\right)}^{\left(1,2\right)}\}⊇\{B^{\left(1,3\right)}A^{\left(1,3\right)}\}.\; & \;\langle 6\rangle \; & \{{\left(AB\right)}^{\left(1,2\right)}\}⊇\{B^{\left(1,3\right)}A^{\left(1\right)}\}.\\ \langle 7\rangle \; & \{{\left(AB\right)}^{\left(1,2\right)}\}⊇\{B^{\left(1\right)}A^{\left(1,3,4\right)}\}.\; & \;\langle 8\rangle \; & \{{\left(AB\right)}^{\left(1,2\right)}\}⊇\{B^{\left(1\right)}A^{\left(1,4\right)}\}.\\ \langle 9\rangle \; & r(M)=r(A)+r(B)-r(N)=\min\{m,\;n,\;p\}.\; \end{array}$$
(h) *The following 5 statements are equivalent:*
  $$\;\begin{array}{cccc} \langle 1\rangle \; & \{{\left(AB\right)}^{\left(1,2\right)}\}⊇\{B^{\left(1,3,4\right)}A^{\left(1,3,4\right)}\}.\; & \;\langle 2\rangle \; & \{{\left(AB\right)}^{\left(1,2\right)}\}⊇\{B^{\left(1,3,4\right)}A^{\left(1,4\right)}\}.\\ \langle 10\rangle \; & \{{\left(AB\right)}^{\left(1,2\right)}\}⊇\{B^{\left(1,3\right)}A^{\left(1,3,4\right)}\}.\; & \;\langle 4\rangle \; & \{{\left(AB\right)}^{\left(1,2\right)}\}⊇\{B^{\left(1,3\right)}A^{\left(1,4\right)}\}.\\ \langle 5\rangle \; & r(M)=r(A)+r(B)-r(N)=\min\{m,\;n,\;p,\;t\}.\; \end{array}$$
(i) *The following 5 statements are equivalent:*
  $$\;\begin{array}{cccc} \langle 1\rangle \; & \{{\left(AB\right)}^{\left(1,2\right)}\}⊇\{B^{\left(1,2,4\right)}A^{\left(1,2,3\right)}\}.\; & \;\langle 2\rangle \; & \{{\left(AB\right)}^{\left(1,2\right)}\}⊇\{B^{\left(1,2,4\right)}A^{\left(1,2\right)}\}.\\ \langle 3\rangle \; & \{{\left(AB\right)}^{\left(1,2\right)}\}⊇\{B^{\left(1,2\right)}A^{\left(1,2,3\right)}\}.\; & \;\langle 4\rangle \; & \{{\left(AB\right)}^{\left(1,2\right)}\}⊇\{B^{\left(1,2\right)}A^{\left(1,2\right)}\}.\\ \langle 5\rangle \; & A=0\;\text{or}\;B=0\;\text{or}\;r(A)=n\;\text{or}\;r(B)=n.\; \end{array}$$
(j) *The following 5 statements are equivalent:*
  $$\;\begin{array}{cccc} \langle 1\rangle \; & \{{\left(AB\right)}^{\left(1,2\right)}\}⊇\{B^{\left(1,2,4\right)}A^{\left(1,3\right)}\}.\; & \;\langle 2\rangle \; & \{{\left(AB\right)}^{\left(1,2\right)}\}⊇\{B^{\left(1,2,4\right)}A^{\left(1\right)}\}.\\ \langle 3\rangle \; & \{{\left(AB\right)}^{\left(1,2\right)}\}⊇\{B^{\left(1,2\right)}A^{\left(1,3\right)}\}.\; & \;\langle 4\rangle \; & \{{\left(AB\right)}^{\left(1,2\right)}\}⊇\{B^{\left(1,2\right)}A^{\left(1\right)}\}.\\ \langle 5\rangle \; & r(A)=n\;\text{or}\;B=0\;\text{or}\;\{r(A)=m\;\text{and}\;r(B)=n\}.\; \end{array}$$
(k) *The following 5 statements are equivalent:*
  $$\;\begin{array}{cccc} \langle 1\rangle \; & \{{\left(AB\right)}^{\left(1,2\right)}\}⊇\{B^{\left(1,4\right)}A^{\left(1,2,3\right)}\}.\; & \;\langle 2\rangle \; & \{{\left(AB\right)}^{\left(1,2\right)}\}⊇\{B^{\left(1,4\right)}A^{\left(1,2\right)}\}.\\ \langle 3\rangle \; & \{{\left(AB\right)}^{\left(1,2\right)}\}⊇\{B^{\left(1\right)}A^{\left(1,2,3\right)}\}.\; & \;\langle 4\rangle \; & \{{\left(AB\right)}^{\left(1,2\right)}\}⊇\{B^{\left(1\right)}A^{\left(1,2\right)}\}.\\ \langle 5\rangle \; & A=0\;\text{or}\;r(B)=n\;\text{or}\;\{r(A)=n\;\text{and}\;r(B)=p\}.\; \end{array}$$
(l) *The following 5 statements are equivalent:*
  $$\;\begin{array}{cccc} \langle 1\rangle \; & \{{\left(AB\right)}^{\left(1,2\right)}\}⊇\{B^{\left(1,4\right)}A^{\left(1,3\right)}\}.\; & \;\langle 2\rangle \; & \{{\left(AB\right)}^{\left(1,2\right)}\}⊇\{B^{\left(1,4\right)}A^{\left(1\right)}\}.\\ \langle 3\rangle \; & \{{\left(AB\right)}^{\left(1,2\right)}\}⊇\{B^{\left(1\right)}A^{\left(1,3\right)}\}.\; & \;\langle 4\rangle \; & \{{\left(AB\right)}^{\left(1,2\right)}\}⊇\{B^{\left(1\right)}A^{\left(1\right)}\}.\\ \langle 5\rangle \; & \{r(A)=m\;\text{and}\;r(B)=n\}\;\text{or}\;\{r(A)=r(B)=n\}\;\text{or}\;\{r(A)=n\;\text{and}\;r(B)=p\}.\; \end{array}$$

Proof Applying (8.3) to $B^{†}A^{†}ABB^{†}A^{†}-B^{†}A^{†}$ and simplifying by Lemma 8.6 result in

(9.41) $$r(B^{†}A^{†}ABB^{†}A^{†}-B^{†}A^{†})=r[{\left(B^{†}\right)}^{⁎},\;A^{†}]-r(B^{†}A^{†})-r(A^{†})-r(B^{†})=r(N)+r(M)-r(A)-r(B).$$

Setting both sides of (9.41) equal to zero leads to the equivalence of the second rank equality in 〈5〉 and 〈6〉 of (a). All the remaining equivalences in (a)–(l) follow from Lemma 3.3(b), Lemma 8.3, Theorem 9.1, as well as the simplification of the combined conditions. □

Theorem 9.4 *Let* $A\in C^{m\times n}$ *and* $B\in C^{n\times p}$ *be given, and denote* M=AB *and* $N=[A^{⁎},\;B]$*. Then the following results hold.*

(a) *The following 26 statements are equivalent:*
  $$\;\begin{array}{cccc} \langle 1\rangle \; & \{M^{\left(1,3\right)}\}∋B^{†}A^{†}.\; & \;\langle 2\rangle \; & \{M^{\left(1,3\right)}\}⊇\{B^{\left(1,3,4\right)}A^{†}\}.\\ \langle 3\rangle \; & \{M^{\left(1,3\right)}\}⊇\{B^{\left(1,2,3\right)}A^{†}\}.\; & \;\langle 4\rangle \; & \{M^{\left(1,3\right)}\}⊇\{B^{\left(1,3\right)}A^{†}\}.\\ \langle 5\rangle \; & MM^{†}=MB^{†}A^{†}.\; & \;\langle 6\rangle \; & M^{⁎}MB^{†}A^{†}=M^{⁎}.\\ \langle 7\rangle \; & MB^{†}A^{⁎}M=AA^{⁎}M.\; & \;\langle 8\rangle \; & BB^{†}A^{⁎}M=A^{⁎}M.\\ \langle 9\rangle \; & {\left(ABB^{†}\right)}^{†}=BB^{†}A^{†}.\; & \;\langle 10\rangle \; & B^{⁎}{\left(ABB^{†}\right)}^{†}AA^{⁎}=M^{⁎}.\\ \langle 11\rangle \; & {\left(AE_{B}\right)}^{†}=E_{B}A^{†}.\; & \;\langle 12\rangle \; & A{\left(AE_{B}\right)}^{†}=AE_{B}A^{†}.\\ \langle 13\rangle \; & MM^{†}A=MB^{†}.\; & \;\langle 14\rangle \; & A^{⁎}ABB^{†}=BB^{†}A^{⁎}A.\\ \langle 15\rangle \; & r[A^{⁎}M,\;B]=r(B).\; & \;\langle 16\rangle \; & r[A^{⁎}AE_{B},\;E_{B}]=r(E_{B}).\\ \langle 17\rangle \; & R(A^{⁎}M)\subseteq R(B).\; & \;\langle 18\rangle \; & R(A^{⁎}AE_{B})\subseteq N(B^{⁎}).\\ \langle 19\rangle \; & R(A^{⁎}M)=R(A^{⁎})\cap R(B).\; & \;\langle 20\rangle \; & R(A^{⁎}AE_{B})=R(A^{⁎})\cap N(B^{⁎}).\\ \langle 21\rangle \; & R(A^{⁎}ABB^{†})=R(BB^{†}A^{⁎}A).\; & \;\langle 22\rangle \; & R(A^{⁎}AE_{B})=R(E_{B}A^{⁎}A).\\ \langle 23\rangle \; & MB^{†}A^{†}\;\text{is an orthogonal projector}.\;\\ \langle 24\rangle \; & AE_{B}A^{†}\;\text{is an orthogonal projector}.\;\\ \langle 25\rangle \; & MB^{†}A^{†}M=M\;\text{and}\;{\left(MB^{†}A^{†}\right)}^{⁎}=MB^{†}A^{†}.\;\\ \langle 26\rangle \; & r(N)=r(A)+r(B)-r(M)\;\text{and}\;r[AA^{⁎}M,\;M]=r(M).\; \end{array}$$
(b) *The following 6 statements are equivalent:*
  $$\;\begin{array}{cccc} \langle 1\rangle \; & \{M^{\left(1,3\right)}\}⊇\{B^{†}A^{\left(1,3,4\right)}\}.\; & \;\langle 2\rangle \; & \{M^{\left(1,3\right)}\}⊇\{B^{\left(1,3,4\right)}A^{\left(1,3,4\right)}\}.\\ \langle 3\rangle \; & \{M^{\left(1,3\right)}\}⊇\{B^{\left(1,2,3\right)}A^{\left(1,3,4\right)}\}.\; & \;\langle 4\rangle \; & \{M^{\left(1,3\right)}\}⊇\{B^{\left(1,3\right)}A^{\left(1,3,4\right)}\}.\\ \langle 5\rangle \; & M^{⁎}MB^{†}F_{A}UE_{A}=M^{⁎}-M^{⁎}MB^{†}A^{†}\;\text{holds for all}\;\text{U}.\;\\ \langle 6\rangle \; & R(A^{⁎}M)\subseteq R(B).\; \end{array}$$
(c) *The following 6 statements are equivalent:*
  $$\;\begin{array}{cccc} \langle 1\rangle \; & \{M^{\left(1,3\right)}\}⊇\{B^{†}A^{\left(1,2,4\right)}\}.\; & \;\langle 2\rangle \; & \{M^{\left(1,3\right)}\}⊇\{B^{\left(1,3,4\right)}A^{\left(1,2,4\right)}\}.\\ \langle 3\rangle \; & \{M^{\left(1,3\right)}\}⊇\{B^{\left(1,2,3\right)}A^{\left(1,2,4\right)}\}.\; & \;\langle 4\rangle \; & \{M^{\left(1,3\right)}\}⊇\{B^{\left(1,3\right)}A^{\left(1,2,4\right)}\}.\\ \langle 5\rangle \; & M^{⁎}MB^{†}A^{†}AUE_{A}=M^{⁎}-M^{⁎}MB^{†}A^{†}\;\text{holds for all}\;\text{U}.\;\\ \langle 6\rangle \; & M=0\;\text{or}\;\{r(A)=m\;\text{and}\;R(A^{⁎}M)\subseteq R(B)\}.\; \end{array}$$
(d) *The following 6 statements are equivalent:*
  $$\;\begin{array}{cccc} \langle 1\rangle \; & \{M^{\left(1,3\right)}\}⊇\{B^{†}A^{\left(1,2,3\right)}\}.\; & \;\langle 2\rangle \; & \{M^{\left(1,3\right)}\}⊇\{B^{\left(1,3,4\right)}A^{\left(1,2,3\right)}\}.\\ \langle 3\rangle \; & \{M^{\left(1,3\right)}\}⊇\{B^{\left(1,2,3\right)}A^{\left(1,2,3\right)}\}.\; & \;\langle 4\rangle \; & \{M^{\left(1,3\right)}\}⊇\{B^{\left(1,3\right)}A^{\left(1,2,3\right)}\}.\\ \langle 5\rangle \; & M^{⁎}MB^{†}F_{A}UAA^{†}=M^{⁎}-M^{⁎}MB^{†}A^{†}\;\text{holds for all}\;\text{U}.\;\\ \langle 6\rangle \; & R(A^{⁎}M)\subseteq R(B).\; \end{array}$$
(e) *The following 6 statements are equivalent:*
  $$\;\begin{array}{cccc} \langle 1\rangle \; & \{M^{\left(1,3\right)}\}⊇\{B^{†}A^{\left(1,4\right)}\}.\; & \;\langle 2\rangle \; & \{M^{\left(1,3\right)}\}⊇\{B^{\left(1,3,4\right)}A^{\left(1,4\right)}\}.\\ \langle 3\rangle \; & \{M^{\left(1,3\right)}\}⊇\{B^{\left(1,2,3\right)}A^{\left(1,4\right)}\}.\; & \;\langle 4\rangle \; & \{M^{\left(1,3\right)}\}⊇\{B^{\left(1,3\right)}A^{\left(1,4\right)}\}.\\ \langle 5\rangle \; & M^{⁎}MB^{†}UE_{A}=M^{⁎}-M^{⁎}MB^{†}A^{†}\;\text{holds for all}\;\text{U}.\;\\ \langle 6\rangle \; & M=0\;\text{or}\;\{r(A)=m\;\text{and}\;R(A^{⁎}M)\subseteq R(B)\}.\; \end{array}$$
(f) *The following 6 statements are equivalent:*
  $$\;\begin{array}{cccc} \langle 1\rangle \; & \{M^{\left(1,3\right)}\}⊇\{B^{†}A^{\left(1,3\right)}\}.\; & \;\langle 2\rangle \; & \{M^{\left(1,3\right)}\}⊇\{B^{\left(1,3,4\right)}A^{\left(1,3\right)}\}.\\ \langle 3\rangle \; & \{M^{\left(1,3\right)}\}⊇\{B^{\left(1,2,3\right)}A^{\left(1,3\right)}\}.\; & \;\langle 4\rangle \; & \{M^{\left(1,3\right)}\}⊇\{B^{\left(1,3\right)}A^{\left(1,3\right)}\}.\\ \langle 5\rangle \; & M^{⁎}MB^{†}F_{A}U=M^{⁎}-M^{⁎}MB^{†}A^{†}\;\text{holds for all}\;\text{U}.\;\\ \langle 6\rangle \; & R(A^{⁎}M)\subseteq R(B).\; \end{array}$$
(g) *The following 6 statements are equivalent:*
  $$\;\begin{array}{cccc} \langle 1\rangle \; & \{M^{\left(1,3\right)}\}⊇\{B^{†}A^{\left(1,2\right)}\}.\; & \;\langle 2\rangle \; & \{M^{\left(1,3\right)}\}⊇\{B^{\left(1,3,4\right)}A^{\left(1,2\right)}\}.\\ \langle 3\rangle \; & \{M^{\left(1,3\right)}\}⊇\{B^{\left(1,2,3\right)}A^{\left(1,2\right)}\}.\; & \;\langle 4\rangle \; & \{M^{\left(1,3\right)}\}⊇\{B^{\left(1,3\right)}A^{\left(1,2\right)}\}.\\ \langle 5\rangle \; & (M^{⁎}MB^{†}A^{†}+M^{⁎}MB^{†}F_{A}U_{1})(AA^{†}+AU_{2}E_{A})=M^{⁎}\;\text{holds for all }U_{1}\text{ and }U_{2}.\;\\ \langle 6\rangle \; & M=0\;\text{or}\;\{r(A)=m\;\text{and}\;R(A^{⁎}M)\subseteq R(B)\}.\; \end{array}$$
(h) *The following 6 statements are equivalent:*
  $$\;\begin{array}{cccc} \langle 1\rangle \; & \{M^{\left(1,3\right)}\}⊇\{B^{†}A^{\left(1\right)}\}.\; & \;\langle 2\rangle \; & \{M^{\left(1,3\right)}\}⊇\{B^{\left(1,3,4\right)}A^{\left(1\right)}\}.\\ \langle 3\rangle \; & \{M^{\left(1,3\right)}\}⊇\{B^{\left(1,2,3\right)}A^{\left(1\right)}\}.\; & \;\langle 4\rangle \; & \{M^{\left(1,3\right)}\}⊇\{B^{\left(1,3\right)}A^{\left(1\right)}\}.\\ \langle 5\rangle \; & M^{⁎}MB^{†}F_{A}U_{1}+M^{⁎}MB^{†}U_{2}E_{A}=M^{⁎}-M^{⁎}MB^{†}A^{†}\;\text{holds for all }U_{1}\text{ and }U_{2}.\;\\ \langle 6\rangle \; & M=0\;\text{or}\;\{r(A)=m\;\text{and}\;R(A^{⁎}M)\subseteq R(B)\}.\; \end{array}$$
(i) *The following 6 statements are equivalent:*
  $$\;\begin{array}{cccc} \langle 1\rangle \; & \{M^{\left(1,3\right)}\}⊇\{B^{\left(1,2,4\right)}A^{†}\}.\; & \;\langle 2\rangle \; & \{M^{\left(1,3\right)}\}⊇\{B^{\left(1,4\right)}A^{†}\}.\\ \langle 3\rangle \; & \{M^{\left(1,3\right)}\}⊇\{B^{\left(1,2\right)}A^{†}\}.\; & \;\langle 4\rangle \; & \{M^{\left(1,3\right)}\}⊇\{B^{\left(1\right)}A^{†}\}.\\ \langle 5\rangle \; & M^{⁎}MVE_{B}A^{†}=M^{⁎}-M^{⁎}MB^{†}A^{†}\;\text{holds for all}\;\text{V}.\;\\ \langle 6\rangle \; & M=0\;\text{or}\;R(A^{⁎})\subseteq R(B).\; \end{array}$$
(j) *The following 6 statements are equivalent:*
  $$\;\begin{array}{cccc} \langle 1\rangle \; & \{M^{\left(1,3\right)}\}⊇\{B^{\left(1,2,4\right)}A^{\left(1,3,4\right)}\}.\; & \;\langle 2\rangle \; & \{M^{\left(1,3\right)}\}⊇\{B^{\left(1,4\right)}A^{\left(1,3,4\right)}\}.\\ \langle 3\rangle \; & \{M^{\left(1,3\right)}\}⊇\{B^{\left(1,2\right)}A^{\left(1,3,4\right)}\}.\; & \;\langle 4\rangle \; & \{M^{\left(1,3\right)}\}⊇\{B^{\left(1\right)}A^{\left(1,3,4\right)}\}.\\ \langle 5\rangle \; & (M^{⁎}MB^{†}+M^{⁎}MVE_{B})(A^{†}+F_{A}UE_{A})=M^{⁎}\;\text{holds for all}\;\text{U}\;\text{and}\;\text{V}.\;\\ \langle 6\rangle \; & M=0\;\text{or}\;r(B)=n\;\text{or}\;\{r(A)=m\;\text{and}\;R(A^{⁎})\subseteq R(B)\}.\; \end{array}$$
(k) *The following 6 statements are equivalent:*
  $$\;\begin{array}{cccc} \langle 1\rangle \; & \{M^{\left(1,3\right)}\}⊇\{B^{\left(1,2,4\right)}A^{\left(1,2,4\right)}\}.\; & \;\langle 2\rangle \; & \{M^{\left(1,3\right)}\}⊇\{B^{\left(1,4\right)}A^{\left(1,2,4\right)}\}.\\ \langle 3\rangle \; & \{M^{\left(1,3\right)}\}⊇\{B^{\left(1,2\right)}A^{\left(1,2,4\right)}\}.\; & \;\langle 4\rangle \; & \{M^{\left(1,3\right)}\}⊇\{B^{\left(1\right)}A^{\left(1,2,4\right)}\}.\\ \langle 5\rangle \; & (M^{⁎}MB^{†}+M^{⁎}MVE_{B})(A^{†}+A^{†}AUE_{A})=M^{⁎}\;\text{holds for all}\;\text{U}\;\text{and}\;\text{V}.\;\\ \langle 6\rangle \; & M=0\;\text{or}\;\{r(A)=m\;\text{and}\;R(A^{⁎})\subseteq R(B)\}.\; \end{array}$$
(l) *The following 6 statements are equivalent:*
  $$\;\begin{array}{cccc} \langle 1\rangle \; & \{M^{\left(1,3\right)}\}⊇\{B^{\left(1,2,4\right)}A^{\left(1,2,3\right)}\}.\; & \;\langle 2\rangle \; & \{M^{\left(1,3\right)}\}⊇\{B^{\left(1,4\right)}A^{\left(1,2,3\right)}\}.\\ \langle 3\rangle \; & \{M^{\left(1,3\right)}\}⊇\{B^{\left(1,2\right)}A^{\left(1,2,3\right)}\}.\; & \;\langle 4\rangle \; & \{M^{\left(1,3\right)}\}⊇\{B^{\left(1\right)}A^{\left(1,2,3\right)}\}.\\ \langle 5\rangle \; & (M^{⁎}MB^{†}+M^{⁎}MVE_{B})(A^{†}+F_{A}UAA^{†})=M^{⁎}\;\text{holds for all}\;\text{U}\;\text{and}\;\text{V}.\;\\ \langle 6\rangle \; & M=0\;\text{or}\;r(B)=n.\; \end{array}$$
(m) *The following 6 statements are equivalent:*
  $$\;\begin{array}{cccc} \langle 1\rangle \; & \{M^{\left(1,3\right)}\}⊇\{B^{\left(1,2,4\right)}A^{\left(1,4\right)}\}.\; & \;\langle 2\rangle \; & \{M^{\left(1,3\right)}\}⊇\{B^{\left(1,4\right)}A^{\left(1,4\right)}\}.\\ \langle 3\rangle \; & \{M^{\left(1,3\right)}\}⊇\{B^{\left(1,2\right)}A^{\left(1,4\right)}\}.\; & \;\langle 4\rangle \; & \{M^{\left(1,3\right)}\}⊇\{B^{\left(1\right)}A^{\left(1,4\right)}\}.\\ \langle 5\rangle \; & (M^{⁎}MB^{†}+M^{⁎}MVE_{B})(A^{†}+UE_{A})=M^{⁎}\;\text{holds for all}\;\text{U}\;\text{and}\;\text{V}.\;\\ \langle 6\rangle \; & M=0\;\text{or}\;\{r(A)=m\;\text{and}\;R(A^{⁎})\subseteq R(B)\}.\; \end{array}$$
(n) *The following 6 statements are equivalent:*
  $$\;\begin{array}{cccc} \langle 1\rangle \; & \{M^{\left(1,3\right)}\}⊇\{B^{\left(1,2,4\right)}A^{\left(1,3\right)}\}.\; & \;\langle 2\rangle \; & \{M^{\left(1,3\right)}\}⊇\{B^{\left(1,4\right)}A^{\left(1,3\right)}\}.\\ \langle 3\rangle \; & \{M^{\left(1,3\right)}\}⊇\{B^{\left(1,2\right)}A^{\left(1,3\right)}\}.\; & \;\langle 4\rangle \; & \{M^{\left(1,3\right)}\}⊇\{B^{\left(1\right)}A^{\left(1,3\right)}\}.\\ \langle 5\rangle \; & (M^{⁎}MB^{†}+M^{⁎}MVE_{B})(A^{†}+F_{A}U)=M^{⁎}\;\text{holds for all}\;\text{U}\;\text{and}\;\text{V}.\;\\ \langle 6\rangle \; & M=0\;\text{or}\;r(B)=n.\; \end{array}$$
(o) *The following 6 statements are equivalent:*
  $$\;\begin{array}{cccc} \langle 1\rangle \; & \{M^{\left(1,3\right)}\}⊇\{B^{\left(1,2,4\right)}A^{\left(1,2\right)}\}.\; & \;\langle 2\rangle \; & \{M^{\left(1,3\right)}\}⊇\{B^{\left(1,4\right)}A^{\left(1,2\right)}\}.\\ \langle 3\rangle \; & \{M^{\left(1,3\right)}\}⊇\{B^{\left(1,2\right)}A^{\left(1,2\right)}\}.\; & \;\langle 4\rangle \; & \{M^{\left(1,3\right)}\}⊇\{B^{\left(1\right)}A^{\left(1,2\right)}\}.\\ \langle 5\rangle \; & (M^{⁎}MB^{†}+M^{⁎}MVE_{B})(A^{†}+F_{A}U_{1})(AA^{†}+AU_{2}E_{A})=M^{⁎}\text{holds for all }U_{1}\text{, }U_{2}\text{, and}\;\text{V}.\;\\ \langle 6\rangle \; & M=0\;\text{or}\;\{r(A)=m\;\text{and}\;r(B)=n\}.\; \end{array}$$
(p) *The following 6 statements are equivalent:*
  $$\;\begin{array}{cccc} \langle 1\rangle \; & \{M^{\left(1,3\right)}\}⊇\{B^{\left(1,2,4\right)}A^{\left(1\right)}\}.\; & \;\langle 2\rangle \; & \{M^{\left(1,3\right)}\}⊇\{B^{\left(1,4\right)}A^{\left(1\right)}\}.\\ \langle 3\rangle \; & \{M^{\left(1,3\right)}\}⊇\{B^{\left(1,2\right)}A^{\left(1\right)}\}.\; & \;\langle 4\rangle \; & \{M^{\left(1,3\right)}\}⊇\{B^{\left(1\right)}A^{\left(1\right)}\}.\\ \langle 5\rangle \; & (M^{⁎}MB^{†}+M^{⁎}MVE_{B})(A^{†}+F_{A}U_{1}+U_{2}E_{A})=M^{⁎}\;\text{holds for all }U_{1}\text{, }U_{2}\text{, and}\;\text{V}.\;\\ \langle 6\rangle \; & M=0\;\text{or}\;\{r(A)=m\;\text{and}\;r(B)=n\}.\; \end{array}$$

Proof The equivalence of 〈1〉 and 〈25〉 in (a) follows from the definition of {1,3}-generalized inverses of a matrix. The equivalences of 〈1〉–〈6〉 in (a) follows from (3.65).

Applying (4.15) and simplifying yield

(9.42) $$r(MM^{†}-MB^{†}A^{†})=r(M^{⁎}-M^{⁎}MB^{†}A^{†})=r[M^{⁎}AA^{⁎}-M^{⁎}MB^{†}A^{⁎})=r(AA^{⁎}AB-ABB^{†}A^{⁎}AB)=r\left[\begin{array}{cc} B^{⁎}B\; & B^{⁎}A^{⁎}AB\\ AB\; & AA^{⁎}AB \end{array}\right]-r(B)=r\left(\left[\begin{array}{c} B^{⁎}\\ A \end{array}\right][B,\;A^{⁎}AB]\right)-r(B)=r\left(\left[\begin{array}{c} B^{⁎}\\ B^{⁎}A^{⁎}A \end{array}\right][B,\;A^{⁎}AB]\right)-r(B)=r[A^{⁎}AB,\;B]-r(B).$$

Setting all sides of (9.42) equal to zero leads to the equivalences of 〈5〉–〈7〉, 〈15〉, and 〈17〉.

By (4.29),

(9.43) $$r(BB^{†}A^{⁎}M-A^{⁎}M)=r(E_{B}A^{⁎}M)=r[A^{⁎}AB,\;B]-r(B).$$

Setting all sides of (9.43) equal to zero leads to the equivalence of 〈8〉 and 〈15〉.

By (4.4),

(9.44) $$r[A^{⁎}AE_{B},\;E_{B}]-r(E_{B})=r(BB^{†}A^{⁎}AE_{B})=r(B^{⁎}A^{⁎}AE_{B})=r[A^{⁎}AB,\;B^{⁎}]-r(B).$$

Setting all sides of (9.44) equal to zero leads to the equivalences of 〈15〉, 〈16〉, and 〈18〉.

It follows from $t_{5}\geq t_{6}$ in (4.32) that

(9.45) $$r[A^{⁎}AB,\;B]-r(B)⩾r(M)+r(M)-r(A)-r(B)⩾0.$$

Also by (8.11),

(9.46) $$R(A^{⁎}AB)\subseteq R(B)\Rightarrow R(AA^{⁎}AB)\subseteq R(AB).$$

Combining (9.45) and (9.46) yields

(9.47) $$r[A^{⁎}AB,\;B]=r(B)\Rightarrow r(M)=r(A)+r(B)-r(M)\;\text{and}\;r[AA^{⁎}AB,\;AB]=r(M).$$

Hence 〈28〉 implies 〈15〉. Conversely, substituting the two rank equalities in 〈28〉 into both sides of $t_{4}\geq t_{5}$ in (4.32) yields $r[A^{⁎}AB,\;B]⩽r(B)$, which in fact implies $r[A^{⁎}AB,\;B]=r(B)$. So that 〈15〉 is equivalent to 〈26〉.

Applying (4.13) to ${\left(ABB^{†}\right)}^{†}-BB^{†}A^{†}$ and simplifying yield

(9.48) $$r\left({\left(ABB^{†}\right)}^{†}-BB^{†}A^{†}\right)=r\left(B^{⁎}{\left(ABB^{†}\right)}^{†}AA^{⁎}-B^{⁎}A^{⁎}\right)=r\left[\begin{array}{cc} {\left(ABB^{†}\right)}^{⁎}(ABB^{†}){\left(ABB^{†}\right)}^{⁎} & {\left(ABB^{†}\right)}^{⁎}AA^{⁎}\\ B^{⁎}{\left(ABB^{†}\right)}^{⁎} & B^{⁎}A^{⁎} \end{array}\right]-r(ABB^{†})=r\left[\begin{array}{cc} B^{⁎}A^{⁎}ABB^{†}A^{⁎} & B^{⁎}A^{⁎}AA^{⁎}\\ B^{⁎}A^{⁎} & B^{⁎}A^{⁎} \end{array}\right]-r(M)=r\left[\begin{array}{cc} 0 & B^{⁎}A^{⁎}AA^{⁎}-B^{⁎}A^{⁎}ABB^{†}A^{⁎}\\ B^{⁎}A^{⁎} & 0 \end{array}\right]-r(M)=r(AA^{⁎}AB-ABB^{†}A^{⁎}AB)=r[A^{⁎}AB,\;B]-r(B)\;\;\text{(by (9.42))}.$$

Setting all sides of (9.48) equal to zero leads to the equivalences of 〈9〉, 〈10〉, and 〈15〉.

Replacing $BB^{†}$ with $E_{B}$ in (9.48) and applying (9.44) yield

(9.49) $$r\left({\left(AE_{B}\right)}^{†}-E_{B}A^{†}\right)=r[A^{⁎}AE_{B},\;E_{B}]-r(E_{B})=r[A^{⁎}AB,\;B^{⁎}]-r(B).$$

Setting all sides of (9.49) equal to zero leads to the equivalence of 〈11〉 and 〈15〉.

By (9.42) and (9.44),

(9.50) $$r\left(A{\left(AE_{B}\right)}^{†}-AE_{B}A^{†}\right)=r\left((AE_{B}){\left(AE_{B}\right)}^{†}-(AE_{B}){\left(E_{B}\right)}^{†}A^{†}\right)=r[A^{⁎}AE_{B},\;E_{B}]-r(E_{B})=r[A^{⁎}AB,\;B^{⁎}]-r(B).$$

Setting all sides of (9.50) equal to zero leads to the equivalence of 〈12〉 and 〈15〉.

By (4.4),

(9.51) $$r(MM^{†}A-MB^{†})=r(M^{⁎}A-M^{⁎}ABB^{†})=r(E_{B}A^{⁎}M)=r[A^{⁎}M,\;B]-r(B).$$

Setting both sides of (9.51) equal to zero leads to the equivalence of 〈13〉 and 〈15〉.

By (4.29),

(9.52) $$r(A^{⁎}ABB^{†}-BB^{†}A^{⁎}A)=2r[A^{⁎}ABB^{†},\;BB^{†}]-2r(BB^{†})=2r[A^{⁎}AB,\;B]-2r(B).$$

Setting all sides of (9.52) equal to zero leads to the equivalence of 〈14〉 and 〈15〉.

It follows from 〈17〉 that

(9.53) $$R(A^{⁎}AB)\subseteq R(A^{⁎})\cap R(B),$$

and from (4.11) and 〈28〉 that

(9.54) $$\dim[R(A^{⁎})\cap R(B)]=r(A)+r(B)-r[A^{⁎},\;B]=r(M).$$

Applying (8.10) to (9.53) and (9.54) leads to the range identities in 〈19〉. Conversely, 〈19〉 obviously implies 〈17〉. The equivalence of 〈18〉 and 〈20〉 can be shown similarly.

Replacing $BB^{†}$ with $E_{B}$ in (9.55) and applying (9.44) yield

$$R(A^{⁎}AE_{B})=R(E_{B}A^{⁎}A)\Leftrightarrow r[A^{⁎}AE_{B},\;E_{B}]=r(E_{B}),$$

establishing the equivalence of 〈16〉 and 〈22〉.

Since $MM^{†}$ is both idempotent and Hermitian, 〈5〉 implies 〈23〉. Conversely, the first equality in 〈23〉 is equivalent to $MB^{†}A^{†}M=M$ by Theorem 9.1〈48〉 and 〈93〉. This equality together with the second equality in 〈23〉 implies 〈1〉.

Replacing $BB^{†}$ with $E_{B}$ in 〈23〉 leads to the equivalence of 〈16〉 and 〈24〉.

Applying Lemma 8.6 and (4.4) to $\left[A^{⁎}ABB^{†},\;BB^{†}A^{⁎}A\right]$ yields

$$r[A^{⁎}ABB^{†},\;BB^{†}A^{⁎}A]=r[A^{⁎}AB,\;BB^{†}A^{⁎}]=r[A^{⁎}AB-BB^{†}A^{⁎}AB,\;BB^{†}A^{⁎}]=r[(I_{n}-BB^{†})A^{⁎}AB,\;BB^{†}A^{⁎}]=r\left((I_{n}-BB^{†})A^{⁎}AB\right)+r(BB^{†}A^{⁎})=r[A^{⁎}AB,\;B]+r(M)-r(B).$$

Hence

(9.55) $$R(A^{⁎}ABB^{†})=R(BB^{†}A^{⁎}A)\Leftrightarrow r[A^{⁎}ABB^{†},\;BB^{†}A^{⁎}A]=r(A^{⁎}ABB^{†})=r(BB^{†}A^{⁎}A)=r(M)\Leftrightarrow r[A^{⁎}AB,\;B]=r(B),$$

establishing the equivalence of 〈15〉 and 〈21〉. Replacing $BB^{†}$ with $E_{B}$ in 〈21〉 leads to the equivalence of 〈22〉 and 〈16〉.

The equivalences of 〈1〉–〈5〉 in (b) follow from (3.152). By Lemma 5.2, the matrix equation in 〈5〉 holds for all *U* if and only if

(9.56) $$[M^{⁎}MB^{†}A^{†}-M^{⁎},\;M^{⁎}MB^{†}F_{A}]=0\;\;\mathrm{or}\;\;\left[\begin{array}{c} M^{⁎}MB^{†}A^{†}-M^{⁎}\\ E_{A} \end{array}\right]=0,$$

where by (4.5),

(9.57) $$r[M^{⁎}MB^{†}A^{†}-M^{⁎},\;M^{⁎}MB^{†}F_{A}]=r\left[\begin{array}{cc} M^{⁎}MB^{†}A^{†}-M^{⁎} & M^{⁎}MB^{†}\\ 0 & A \end{array}\right]-r(A)=r\left[\begin{array}{cc} -B^{⁎}A^{⁎} & M^{⁎}MB^{†}\\ -AA^{†} & A \end{array}\right]-r(A)=r\left[\begin{array}{cc} B^{⁎}A^{⁎}A & M^{⁎}MB^{†}\\ A & A \end{array}\right]-r(A)=r\left[\begin{array}{cc} 0 & M^{⁎}MB^{†}-B^{⁎}A^{⁎}A\\ A & 0 \end{array}\right]-r(A)=r(BB^{†}A^{⁎}M-A^{⁎}M)=r[B,\;A^{⁎}M]-r(B)\;\;\;\text{(by (4.4))},$$

and

(9.58) $$r\left[\begin{array}{c} M^{⁎}MB^{†}A^{†}-M^{⁎}\\ E_{A} \end{array}\right]=r(M^{⁎}MB^{†}A^{†}-M^{⁎})+r(E_{A})\;\;\;\text{(by Lemma 4.3(d))}=m-r(A)+r[B,\;A^{⁎}M]-r(B).$$

Combining (9.56) with (9.57) and (9.58) leads to

(9.59) $$[M^{⁎}MB^{†}F_{A},\;M^{⁎}MB^{†}A^{†}-M^{⁎}]=0\Leftrightarrow M^{⁎}MB^{†}A^{†}-M^{⁎}=0\Leftrightarrow R(A^{⁎}M)\subseteq R(B),$$

(9.60) $$\left[\begin{array}{c} M^{⁎}MB^{†}A^{†}-M^{⁎}\\ E_{A} \end{array}\right]=0\Leftrightarrow r(A)=m\;\mathrm{and}\;R(A^{⁎}M)\subseteq R(B).$$

Combining (9.56)–(9.60) leads to the equivalence of 〈5〉 and 〈6〉 in (b).

The equivalences of 〈1〉–〈5〉 in (c) follow from (3.154). By Lemma 5.2, the matrix equation in 〈5〉 holds for all *U* if and only if

(9.61) $$M=0\;\;\mathrm{or}\;\;\left[\begin{array}{c} M^{⁎}MB^{†}A^{†}-M^{⁎}\\ E_{A} \end{array}\right]=0.$$

In this case, combining (9.60) with (9.61) leads to the equivalence of 〈5〉 and 〈6〉 in (c).

The equivalences of 〈1〉–〈5〉 in (d) follow from (3.155). By Lemma 5.2, the matrix equation in 〈5〉 holds for all *U* if and only if

(9.62) $$M=0\;\;\mathrm{or}\;\;\left[\begin{array}{c} M^{⁎}MB^{†}A^{†}-M^{⁎}\\ AA^{†} \end{array}\right]=0,$$

where the first equality in (9.62) is equivalent to (9.59). Thus 〈5〉 and 〈6〉 in (d) are equivalent.

The equivalences of 〈1〉–〈5〉 in (e) follow from (3.156). By Lemma 5.2, the matrix equation in 〈5〉 holds for all *U* if and only if

(9.63) $$[M^{⁎}MB^{†}A^{†}-M^{⁎},\;M^{⁎}MB^{†}]=0\;\mathrm{or}\;\left[\begin{array}{c} M^{⁎}MB^{†}A^{†}-M^{⁎}\\ E_{A} \end{array}\right]=0,$$

where the rank of the first block matrix in (9.63) is

(9.64) $$r[M^{⁎}MB^{†}A^{†}-M^{⁎},\;M^{⁎}MB^{†}]=r[-M^{⁎},\;M^{⁎}MB^{†}]=r[M^{⁎},\;0]=r(M),$$

the second equality in (9.63) is equivalent to (9.60). Combining (9.63) with (9.60) and (9.64) leads to the equivalence of 〈5〉 and 〈6〉 in (e).

The equivalences of 〈1〉–〈5〉 in (f) follow from (3.157). By Lemma 5.2, the matrix equation in 〈5〉 holds for all *U* if and only if $[M^{⁎}MB^{†}A^{†}-M^{⁎},\;M^{⁎}MB^{†}F_{A}]=0$, which is equivalent to (9.59). Thus 〈5〉 and 〈6〉 in (f) are equivalent.

The equivalences of 〈1〉–〈5〉 in (g) follow from (3.158). By Lemma 5.4(e), the matrix equation in 〈5〉 holds for all $U_{1}$ and $U_{2}$ if and only if

(9.65) $$M=0\;\;\mathrm{or}\;\;\left[\begin{array}{cc} M^{⁎}MB^{†}A^{†}-M^{⁎} & M^{⁎}MB^{†}F_{A}\\ E_{A} & 0 \end{array}\right]=0,$$

where by Lemma 4.3(d) and (9.57),

(9.66) $$r\left[\begin{array}{cc} M^{⁎}MB^{†}A^{†}-M^{⁎} & M^{⁎}MB^{†}F_{A}\\ E_{A} & 0 \end{array}\right]=r[M^{⁎}MB^{†}A^{†}-M^{⁎},\;M^{⁎}MB^{†}F_{A}]+r(E_{A})=m-r(A)+r[B,\;A^{⁎}M]-r(B).$$

Hence

(9.67) $$\left[\begin{array}{cc} M^{⁎}MB^{†}A^{†}-M^{⁎} & M^{⁎}MB^{†}F_{A}\\ E_{A} & 0 \end{array}\right]=0\Leftrightarrow r(A)=m\;\mathrm{and}\;R(A^{⁎}M)\subseteq R(B),$$

establishing the equivalence of 〈5〉 and 〈6〉 in (g).

The equivalences of 〈1〉–〈5〉 in (h) follow from (3.159). By Lemma 5.3(b), the matrix equation in 〈5〉 holds for all $U_{1}$ and $U_{2}$ if and only if M=0 or

$$\left[\begin{array}{cc} M^{⁎}MB^{†}A^{†}-M^{⁎} & M^{⁎}MB^{†}F_{A}\\ E_{A} & 0 \end{array}\right]=0,$$

which is equivalent to 〈6〉 in (h) by (9.67).

The equivalences of 〈1〉–〈5〉 in (i) follow from (3.160). By Lemma 5.2, the matrix equation in 〈5〉 holds for all *V* if and only if

(9.68) $$M=0\;\;\mathrm{or}\;\;\left[\begin{array}{c} M^{⁎}MB^{†}A^{†}-M^{⁎}\\ E_{B}A^{†} \end{array}\right]=0,$$

where

$$r\left[\begin{array}{c} M^{⁎}MB^{†}A^{†}-M^{⁎}\\ E_{B}A^{†} \end{array}\right]=r\left[\begin{array}{cc} M^{⁎}MB^{†}A^{†}-M^{⁎} & 0\\ A^{†} & B \end{array}\right]-r(B)=r\left[\begin{array}{cc} -M^{⁎} & -M^{⁎}M\\ A^{†} & B \end{array}\right]-r(B)=r\left[\begin{array}{cc} {\left(AB\right)}^{⁎}AA^{⁎} & {\left(AB\right)}^{⁎}AB\\ A^{⁎} & B \end{array}\right]-r(B)=r\left[\begin{array}{cc} 0 & 0\\ A^{⁎} & B \end{array}\right]-r(B)=r[A^{⁎},\;B]-r(B).$$

Hence

(9.69) $$\left[\begin{array}{c} M^{⁎}MB^{†}A^{†}-M^{⁎}\\ E_{B}A^{†} \end{array}\right]=0\Leftrightarrow r[A^{⁎},\;B]=r(B)\Leftrightarrow R(A^{⁎}M)\subseteq R(B).$$

Combining (9.68) with (9.69) leads to the equivalence of 〈5〉 and 〈6〉 in (i).

The equivalences of 〈1〉–〈5〉 in (j) follow from (3.161). By Lemma 5.4(a), the matrix equation in 〈5〉 holds for all *U* and *V* if and only if

(9.70) $$M=0\;\mathrm{or}\;\left[\begin{array}{cc} M^{⁎}MB^{†}A^{†}-M^{⁎} & M^{⁎}MB^{†}F_{A}\\ E_{B}A^{†} & F_{B}F_{A} \end{array}\right]=0\;\mathrm{or}\;\left[\begin{array}{c} M^{⁎}MB^{†}A^{†}-M^{⁎}\\ E_{B}A^{†}\\ E_{A} \end{array}\right]=0,$$

where the ranks of the two block matrices in (9.70) are

(9.71) $$r\left[\begin{array}{cc} M^{⁎}MB^{†}A^{†}-M^{⁎} & M^{⁎}MB^{†}F_{A}\\ E_{B}A^{†} & E_{B}F_{A} \end{array}\right]=r\left[\begin{array}{cc} M^{⁎}MB^{†}A^{†}-M^{⁎} & M^{⁎}ABB^{†}(I_{n}-A^{†}A)\\ E_{B}A^{†} & E_{B}(I_{n}-A^{†}A) \end{array}\right]=r\left[\begin{array}{cc} M^{⁎}ABB^{†}A^{†}-M^{⁎} & -M^{⁎}AE_{B}\\ E_{B}A^{†} & E_{B} \end{array}\right]=r\left[\begin{array}{cc} 0 & 0\\ 0 & E_{B} \end{array}\right]=n-r(B),$$

(9.72) $$r\left[\begin{array}{c} M^{⁎}MB^{†}A^{†}-M^{⁎}\\ E_{B}A^{†}\\ E_{A} \end{array}\right]=r\left[\begin{array}{c} M^{⁎}ABB^{†}A^{†}-M^{⁎}\\ E_{B}A^{†} \end{array}\right]+r(E_{A})=r\left[\begin{array}{c} 0\\ E_{B}A^{†} \end{array}\right]+r(E_{A})=r[A^{⁎},\;B]-r(B)+m-r(A).$$

Combining (9.70) with (9.71) and (9.72) yields

(9.73) $$\left[\begin{array}{cc} M^{⁎}MB^{†}A^{†}-M^{⁎} & M^{⁎}MB^{†}F_{A}\\ E_{B}A^{†} & E_{B}F_{A} \end{array}\right]=0\Leftrightarrow r(B)=n,$$

(9.74) $$\left[\begin{array}{c} M^{⁎}MB^{†}A^{†}-M^{⁎}\\ E_{B}A^{†}\\ E_{A} \end{array}\right]=0\Leftrightarrow r(A)=m\;\mathrm{and}\;R(A^{⁎})\subseteq R(B),$$

establishing the equivalence of 〈5〉 and 〈6〉 in (j).

The two groups of equivalent facts in 〈1〉–〈5〉 of (k) and (m) follow from (3.162) and (3.164), respectively. By Lemma 5.4(b), the two matrix equations in 〈5〉 of (k) and (m) hold for all *U* and *V* if and only if M=0 or $\left[\begin{array}{c} M^{⁎}MB^{†}A^{†}-M^{⁎}\\ E_{B}A^{†}\\ E_{A} \end{array}\right]=0$, which, by (9.74), is equivalent to 〈6〉 in (k) and (m), respectively.

The two groups of equivalent facts in 〈1〉–〈5〉 of (l) and (n) follow from (3.163) and (3.165), respectively. By Lemma 5.4(b), the two matrix equations in 〈5〉 hold for all *U* and *V* if and only if M=0 or $\left[\begin{array}{cc} M^{⁎}MB^{†}A^{†}-M^{⁎} & M^{⁎}MB^{†}F_{A}\\ E_{B}A^{†} & F_{B}F_{A} \end{array}\right]=0$, which, by (9.73), is equivalent to 〈6〉 in (l) and (n), respectively.

The equivalences of 〈1〉–〈5〉 in (o) follow from (3.166). By Lemma 5.5(b), the matrix equation in 〈5〉 holds for all $U_{1}$, $U_{2}$, and *V* if and only if

(9.75) $$M=0\;\;\mathrm{or}\;\;\left[\begin{array}{cc} M^{⁎}MB^{†}A^{†}-M^{⁎} & M^{⁎}MB^{†}F_{A}\\ E_{B}A^{†} & E_{B}F_{A}\\ E_{A} & 0 \end{array}\right]=0,$$

where by Lemma 4.3(d) and (9.71),

(9.76) $$r\left[\begin{array}{cc} M^{⁎}MB^{†}A^{†}-M^{⁎} & M^{⁎}MB^{†}F_{A}\\ E_{B}A^{†} & E_{B}F_{A}\\ E_{A} & 0 \end{array}\right]=r\left[\begin{array}{cc} M^{⁎}MB^{†}A^{†}-M^{⁎} & M^{⁎}MB^{†}F_{A}\\ E_{B}A^{†} & E_{B}F_{A} \end{array}\right]+r(E_{A})=m-r(A)+n-r(B).$$

Hence

(9.77) $$\left[\begin{array}{cc} M^{⁎}MB^{†}A^{†}-M^{⁎} & M^{⁎}MB^{†}F_{A}\\ E_{B}A^{†} & E_{B}F_{A}\\ E_{A} & 0 \end{array}\right]=0\Leftrightarrow r(A)=m\;\mathrm{and}\;r(B)=n,$$

establishing the equivalence of 〈5〉 and 〈6〉 in (o).

The equivalences of 〈1〉–〈5〉 in (p) follow from (3.166). By Lemma 5.5(b), the matrix equation in 〈5〉 holds for all $U_{1}$, $U_{2}$, and *V* if and only if (9.75) holds, which is equivalent to 〈6〉 in (p) by (9.77). □

The following theorem can be established by a similar approach, and the details are therefore omitted.

Theorem 9.5 *Let* $A\in C^{m\times n}$ *and* $B\in C^{n\times p}$ *be given, and denote* M=AB *and* $N=[A^{⁎},\;B]$*. Then the following results hold.*

(a) *The following 26 statements are equivalent:*
  $$\;\begin{array}{cccc} \langle 1\rangle \; & \{M^{\left(1,4\right)}\}⊇\{B^{†}A^{†}\}.\; & \;\langle 2\rangle \; & \{M^{\left(1,4\right)}\}⊇\{B^{†}A^{\left(1,3,4\right)}\}.\\ \langle 3\rangle \; & \{M^{\left(1,4\right)}\}⊇\{B^{†}A^{\left(1,2,4\right)}\}.\; & \;\langle 4\rangle \; & \{M^{\left(1,4\right)}\}⊇\{B^{†}A^{\left(1,4\right)}\}.\\ \langle 5\rangle \; & M^{†}M=B^{†}A^{†}M.\; & \;\langle 6\rangle \; & B^{†}A^{†}MM^{⁎}=M^{⁎}.\\ \langle 7\rangle \; & B^{⁎}A^{†}ABM^{⁎}=B^{⁎}BM^{⁎}.\; & \;\langle 8\rangle \; & A^{†}ABM^{⁎}=BM^{⁎}.\\ \langle 9\rangle \; & {\left(A^{†}AB\right)}^{†}=B^{†}A^{†}A.\; & \;\langle 10\rangle \; & B^{⁎}B{\left(A^{†}AB\right)}^{†}A^{⁎}=M^{⁎}.\\ \langle 11\rangle \; & {\left(F_{A}B\right)}^{†}=B^{†}F_{A}.\; & \;\langle 12\rangle \; & {\left(F_{A}B\right)}^{†}B=B^{†}F_{A}B.\\ \langle 13\rangle \; & BM^{†}M=A^{†}M.\; & \;\langle 14\rangle \; & A^{†}ABB^{⁎}=BB^{⁎}A^{†}A.\\ \langle 15\rangle \; & r[BM^{⁎},\;A^{⁎}]=r(A).\; & \;\langle 16\rangle \; & r[BB^{⁎}F_{A},\;F_{A}]=r(F_{A}).\\ \langle 17\rangle \; & R(BM^{⁎})\subseteq R(A^{⁎}).\; & \;\langle 18\rangle \; & R(BB^{⁎}F_{A})\subseteq N(A).\\ \langle 19\rangle \; & R(BM^{⁎})=R(B)\cap R(A^{⁎}).\; & \;\langle 20\rangle \; & R(BB^{⁎}F_{A})=R(B)\cap N(A).\\ \langle 21\rangle \; & R(A^{†}ABB^{⁎})=R(BB^{⁎}A^{†}A).\; & \;\langle 22\rangle \; & R(F_{A}BB^{⁎})=R(BB^{⁎}F_{A}).\\ \langle 23\rangle \; & B^{†}A^{†}M\;is\;an\;orthogonal\;projector.\;\\ \langle 24\rangle \; & B^{†}F_{A}B\;is\;an\;orthogonal\;projector.\;\\ \langle 25\rangle \; & MB^{†}A^{†}M=M\;\text{and}\;{\left(B^{†}A^{†}M\right)}^{⁎}=B^{†}A^{†}M.\;\\ \langle 26\rangle \; & r(N)=r(A)+r(B)-r(M)\;\text{and}\;r[B^{⁎}BM^{⁎},\;M^{⁎}]=r(M).\; \end{array}$$
(b) *The following 6 statements are equivalent:*
  $$\;\begin{array}{cccc} \langle 1\rangle \; & \{M^{\left(1,4\right)}\}⊇\{B^{\left(1,3,4\right)}A^{†}\}.\; & \;\langle 2\rangle \; & \{M^{\left(1,4\right)}\}⊇\{B^{\left(1,3,4\right)}A^{\left(1,3,4\right)}\}.\\ \langle 3\rangle \; & \{M^{\left(1,4\right)}\}⊇\{B^{\left(1,3,4\right)}A^{\left(1,2,4\right)}\}.\; & \;\langle 4\rangle \; & \{M^{\left(1,4\right)}\}⊇\{B^{\left(1,3,4\right)}A^{\left(1,4\right)}\}.\\ \langle 5\rangle \; & F_{B}VE_{B}A^{†}MM^{⁎}=M^{⁎}-B^{†}A^{†}MM^{⁎}\;\text{holds for all}\;\text{V}.\;\\ \langle 6\rangle \; & R(BM^{⁎})\subseteq R(A^{⁎}).\; \end{array}$$
(c) *The following 6 statements are equivalent:*
  $$\;\begin{array}{cccc} \langle 1\rangle \; & \{M^{\left(1,4\right)}\}⊇\{B^{\left(1,2,4\right)}A^{†}\}.\; & \;\langle 2\rangle \; & \{M^{\left(1,4\right)}\}⊇\{B^{\left(1,2,4\right)}A^{\left(1,3,4\right)}\}.\\ \langle 3\rangle \; & \{M^{\left(1,4\right)}\}⊇\{B^{\left(1,2,4\right)}A^{\left(1,2,4\right)}\}.\; & \;\langle 4\rangle \; & \{M^{\left(1,4\right)}\}⊇\{B^{\left(1,2,4\right)}A^{\left(1,4\right)}\}.\\ \langle 5\rangle \; & B^{†}BVE_{B}A^{†}MM^{⁎}=M^{⁎}-B^{†}A^{†}MM^{⁎}\;\text{holds for all}\;\text{V}.\;\\ \langle 6\rangle \; & R(BM^{⁎})\subseteq R(A^{⁎}).\; \end{array}$$
(d) *The following 6 statements are equivalent:*
  $$\;\begin{array}{cccc} \langle 1\rangle \; & \{M^{\left(1,4\right)}\}⊇\{B^{\left(1,2,3\right)}A^{†}\}.\; & \;\langle 2\rangle \; & \{M^{\left(1,4\right)}\}⊇\{B^{\left(1,2,3\right)}A^{\left(1,3,4\right)}\}.\\ \langle 3\rangle \; & \{M^{\left(1,4\right)}\}⊇\{B^{\left(1,2,3\right)}A^{\left(1,2,4\right)}\}.\; & \;\langle 4\rangle \; & \{M^{\left(1,4\right)}\}⊇\{B^{\left(1,2,3\right)}A^{\left(1,4\right)}\}.\\ \langle 5\rangle \; & F_{B}VBB^{†}A^{†}MM^{⁎}=M^{⁎}-B^{†}A^{†}MM^{⁎}\;\text{holds for all}\;\text{V}.\;\\ \langle 6\rangle \; & M=0\;\text{or}\;\{r(B)=p\;\text{and}\;R(BM^{⁎})\subseteq R(A^{⁎})\}.\; \end{array}$$
(e) *The following 6 statements are equivalent:*
  $$\;\begin{array}{cccc} \langle 1\rangle \; & \{M^{\left(1,4\right)}\}⊇\{B^{\left(1,4\right)}A^{†}\}.\; & \;\langle 2\rangle \; & \{M^{\left(1,4\right)}\}⊇\{B^{\left(1,4\right)}A^{\left(1,3,4\right)}\}.\\ \langle 3\rangle \; & \{M^{\left(1,4\right)}\}⊇\{B^{\left(1,4\right)}A^{\left(1,2,4\right)}\}.\; & \;\langle 4\rangle \; & \{M^{\left(1,4\right)}\}⊇\{B^{\left(1,4\right)}A^{\left(1,4\right)}\}.\\ \langle 5\rangle \; & VE_{B}A^{†}MM^{⁎}=M^{⁎}-B^{†}A^{†}MM^{⁎}\;\text{holds for all}\;\text{V}.\;\\ \langle 6\rangle \; & R(BM^{⁎})\subseteq R(A^{⁎}).\; \end{array}$$
(f) *The following 6 statements are equivalent:*
  $$\;\begin{array}{cccc} \langle 1\rangle \; & \{M^{\left(1,4\right)}\}⊇\{B^{\left(1,3\right)}A^{†}\}.\; & \;\langle 2\rangle \; & \{M^{\left(1,4\right)}\}⊇\{B^{\left(1,3\right)}A^{\left(1,3,4\right)}\}.\\ \langle 3\rangle \; & \{M^{\left(1,4\right)}\}⊇\{B^{\left(1,3\right)}A^{\left(1,2,4\right)}\}.\; & \;\langle 4\rangle \; & \{M^{\left(1,4\right)}\}⊇\{B^{\left(1,3\right)}A^{\left(1,4\right)}\}.\\ \langle 5\rangle \; & F_{B}VA^{†}MM^{⁎}=M^{⁎}-B^{†}A^{†}MM^{⁎}\;\text{holds for all}\;\text{V}.\;\\ \langle 6\rangle \; & M=0\;\text{or}\;\{r(B)=p\;\text{and}\;R(BM^{⁎})\subseteq R(A^{⁎})\}.\; \end{array}$$
(g) *The following 6 statements are equivalent:*
  $$\;\begin{array}{cccc} \langle 1\rangle \; & \{M^{\left(1,4\right)}\}⊇\{B^{\left(1,2\right)}A^{†}\}.\; & \;\langle 2\rangle \; & \{M^{\left(1,4\right)}\}⊇\{B^{\left(1,2\right)}A^{\left(1,3,4\right)}\}.\\ \langle 3\rangle \; & \{M^{\left(1,4\right)}\}⊇\{B^{\left(1,2\right)}A^{\left(1,2,4\right)}\}.\; & \;\langle 4\rangle \; & \{M^{\left(1,4\right)}\}⊇\{B^{\left(1,2\right)}A^{\left(1,4\right)}\}.\\ \langle 5\rangle \; & (B^{†}+F_{B}V_{1})(BB^{†}A^{†}MM^{⁎}+BV_{2}E_{B}A^{†}MM^{⁎})=M^{⁎}\;\text{holds for all }V_{1}\text{ and }V_{2}.\;\\ \langle 6\rangle \; & M=0\;\text{or}\;\{r(B)=p\;\text{and}\;R(BM^{⁎})\subseteq R(A^{⁎})\}.\; \end{array}$$
(h) *The following 6 statements are equivalent:*
  $$\;\begin{array}{cccc} \langle 1\rangle \; & \{M^{\left(1,4\right)}\}⊇\{B^{\left(1\right)}A^{†}\}.\; & \;\langle 2\rangle \; & \{M^{\left(1,4\right)}\}⊇\{B^{\left(1\right)}A^{\left(1,3,4\right)}\}.\\ \langle 3\rangle \; & \{M^{\left(1,4\right)}\}⊇\{B^{\left(1\right)}A^{\left(1,2,4\right)}\}.\; & \;\langle 4\rangle \; & \{M^{\left(1,4\right)}\}⊇\{B^{\left(1\right)}A^{\left(1,4\right)}\}.\\ \langle 5\rangle \; & F_{B}V_{1}A^{†}MM^{⁎}+V_{2}E_{B}A^{†}MM^{⁎}=M^{⁎}-B^{†}A^{†}MM^{⁎}\;\text{holds for all }V_{1}\text{ and }V_{2}.\;\\ \langle 6\rangle \; & M=0\;\text{or}\;\{r(B)=p\;\text{and}\;R(BM^{⁎})\subseteq R(A^{⁎})\}.\; \end{array}$$
(i) *The following 6 statements are equivalent:*
  $$\;\begin{array}{cccc} \langle 1\rangle \; & \{M^{\left(1,4\right)}\}⊇\{B^{†}A^{\left(1,2,3\right)}\}.\; & \;\langle 2\rangle \; & \{M^{\left(1,4\right)}\}⊇\{B^{†}A^{\left(1,3\right)}\}.\\ \langle 3\rangle \; & \{M^{\left(1,4\right)}\}⊇\{B^{†}A^{\left(1,2\right)}\}.\; & \;\langle 4\rangle \; & \{M^{\left(1,4\right)}\}⊇\{B^{†}A^{\left(1\right)}\}.\\ \langle 5\rangle \; & B^{†}F_{A}UMM^{⁎}=M^{⁎}-B^{†}A^{†}MM^{⁎}\;\text{holds for all}\;\text{U}.\;\\ \langle 6\rangle \; & M=0\;\text{or}\;R(A^{⁎})⊇R(B).\; \end{array}$$
(j) *The following 6 statements are equivalent:*
  $$\;\begin{array}{cccc} \langle 1\rangle \; & \{M^{\left(1,4\right)}\}⊇\{B^{\left(1,3,4\right)}A^{\left(1,2,3\right)}\}.\; & \;\langle 2\rangle \; & \{M^{\left(1,4\right)}\}⊇\{B^{\left(1,3,4\right)}A^{\left(1,3\right)}\}.\\ \langle 3\rangle \; & \{M^{\left(1,4\right)}\}⊇\{B^{\left(1,3,4\right)}A^{\left(1,2\right)}\}.\; & \;\langle 4\rangle \; & \{M^{\left(1,4\right)}\}⊇\{B^{\left(1,3,4\right)}A^{\left(1\right)}\}.\\ \langle 5\rangle \; & (B^{†}+F_{B}VE_{B})(A^{†}MM^{⁎}+F_{A}UMM^{⁎})=M^{⁎}\;\text{holds for all}\;\text{U}\;\text{and}\;\text{V}.\;\\ \langle 6\rangle \; & M=0\;\text{or}\;r(A)=n\;\text{or}\;\{r(B)=p\;\text{and}\;R(A^{⁎})⊇R(B)\}.\; \end{array}$$
(k) *The following 6 statements are equivalent:*
  $$\;\begin{array}{cccc} \langle 1\rangle \; & \{M^{\left(1,4\right)}\}⊇\{B^{\left(1,2,3\right)}A^{\left(1,2,3\right)}\}.\; & \;\langle 2\rangle \; & \{M^{\left(1,4\right)}\}⊇\{B^{\left(1,2,3\right)}A^{\left(1,3\right)}\}.\\ \langle 3\rangle \; & \{M^{\left(1,4\right)}\}⊇\{B^{\left(1,2,3\right)}A^{\left(1,2\right)}\}.\; & \;\langle 4\rangle \; & \{M^{\left(1,4\right)}\}⊇\{B^{\left(1,2,3\right)}A^{\left(1\right)}\}.\\ \langle 5\rangle \; & (B^{†}+F_{B}VBB^{†})(A^{†}MM^{⁎}+F_{A}UMM^{⁎})=M^{⁎}\;\text{holds for all}\;\text{U}\;\text{and}\;\text{V}.\;\\ \langle 6\rangle \; & M=0\;\text{or}\;\{r(B)=p\;\text{and}\;R(A^{⁎})⊇R(B)\}.\; \end{array}$$
(l) *The following 6 statements are equivalent:*
  $$\;\begin{array}{cccc} \langle 1\rangle \; & \{M^{\left(1,4\right)}\}⊇\{B^{\left(1,2,4\right)}A^{\left(1,2,3\right)}\}.\; & \;\langle 2\rangle \; & \{M^{\left(1,4\right)}\}⊇\{B^{\left(1,2,4\right)}A^{\left(1,3\right)}\}.\\ \langle 3\rangle \; & \{M^{\left(1,4\right)}\}⊇\{B^{\left(1,2,4\right)}A^{\left(1,2\right)}\}.\; & \;\langle 4\rangle \; & \{M^{\left(1,4\right)}\}⊇\{B^{\left(1,2,4\right)}A^{\left(1\right)}\}.\\ \langle 5\rangle \; & (B^{†}+B^{†}BVE_{B})(A^{†}MM^{⁎}+F_{A}UMM^{⁎})=M^{⁎}\;\text{holds for all}\;\text{U}\;\text{and}\;\text{V}.\;\\ \langle 6\rangle \; & M=0\;\text{or}\;r(A)=n.\; \end{array}$$
(m) *The following 6 statements are equivalent:*
  $$\;\begin{array}{cccc} \langle 1\rangle \; & \{M^{\left(1,4\right)}\}⊇\{B^{\left(1,3\right)}A^{\left(1,2,3\right)}\}.\; & \;\langle 2\rangle \; & \{M^{\left(1,4\right)}\}⊇\{B^{\left(1,3\right)}A^{\left(1,3\right)}\}.\\ \langle 3\rangle \; & \{M^{\left(1,4\right)}\}⊇\{B^{\left(1,3\right)}A^{\left(1,2\right)}\}.\; & \;\langle 4\rangle \; & \{M^{\left(1,4\right)}\}⊇\{B^{\left(1,3\right)}A^{\left(1\right)}\}.\\ \langle 5\rangle \; & (B^{†}+F_{B}V)(A^{†}MM^{⁎}+F_{A}UMM^{⁎})=M^{⁎}\;\text{holds for all}\;\text{U}\;\text{and}\;\text{V}.\;\\ \langle 6\rangle \; & M=0\;\text{or}\;\{r(B)=p\;\text{and}\;R(A^{⁎})⊇R(B)\}.\; \end{array}$$
(n) *The following 6 statements are equivalent:*
  $$\;\begin{array}{cccc} \langle 1\rangle \; & \{M^{\left(1,4\right)}\}⊇\{B^{\left(1,4\right)}A^{\left(1,2,3\right)}\}.\; & \;\langle 2\rangle \; & \{M^{\left(1,4\right)}\}⊇\{B^{\left(1,4\right)}A^{\left(1,3\right)}\}.\\ \langle 3\rangle \; & \{M^{\left(1,4\right)}\}⊇\{B^{\left(1,4\right)}A^{\left(1,2\right)}\}.\; & \;\langle 4\rangle \; & \{M^{\left(1,4\right)}\}⊇\{B^{\left(1,4\right)}A^{\left(1\right)}\}.\\ \langle 5\rangle \; & (B^{†}+VE_{B})(A^{†}MM^{⁎}+F_{A}UMM^{⁎})=M^{⁎}\;\text{holds for all}\;\text{U}\;\text{and}\;\text{V}.\;\\ \langle 6\rangle \; & M=0\;\text{or}\;r(A)=n.\; \end{array}$$
(o) *The following 6 statements are equivalent:*
  $$\;\begin{array}{cccc} \langle 1\rangle \; & \{M^{\left(1,4\right)}\}⊇\{B^{\left(1,2\right)}A^{\left(1,2,3\right)}\}.\; & \;\langle 2\rangle \; & \{M^{\left(1,4\right)}\}⊇\{B^{\left(1,2\right)}A^{\left(1,3\right)}\}.\\ \langle 3\rangle \; & \{M^{\left(1,4\right)}\}⊇\{B^{\left(1,2\right)}A^{\left(1,2\right)}\}.\; & \;\langle 4\rangle \; & \{M^{\left(1,4\right)}\}⊇\{B^{\left(1,2\right)}A^{\left(1\right)}\}.\\ \langle 5\rangle \; & (B^{†}+F_{B}V_{1})B(B^{†}+V_{2}E_{B})(A^{†}MM^{⁎}+F_{A}UMM^{⁎})=M^{⁎}\;\text{holds for all}\;\text{U}\text{, }V_{1}\text{, and }V_{2}.\;\\ \langle 6\rangle \; & M=0\;\text{or}\;\{r(A)=n\;\text{and}\;r(B)=p\}.\; \end{array}$$
(p) *The following 6 statements are equivalent:*
  $$\;\begin{array}{cccc} \langle 1\rangle \; & \{M^{\left(1,4\right)}\}⊇\{B^{\left(1\right)}A^{\left(1,2,3\right)}\}.\; & \;\langle 2\rangle \; & \{M^{\left(1,4\right)}\}⊇\{B^{\left(1\right)}A^{\left(1,3\right)}\}.\\ \langle 3\rangle \; & \{M^{\left(1,4\right)}\}⊇\{B^{\left(1\right)}A^{\left(1,2\right)}\}.\; & \;\langle 4\rangle \; & \{M^{\left(1,4\right)}\}⊇\{B^{\left(1\right)}A^{\left(1\right)}\}.\\ \langle 5\rangle \; & (B^{†}+F_{B}V_{1}+V_{2}E_{B})(A^{†}MM^{⁎}+F_{A}UMM^{⁎})=M^{⁎}\;\text{holds for all}\;\text{U}\text{, }V_{1}\text{, and }V_{2}.\;\\ \langle 6\rangle \; & M=0\;\text{or}\;\{r(A)=n\;\text{and}\;r(B)=p\}.\; \end{array}$$

## 10 Set inclusions for {1,2,3}-, {1,2,4}-, and {1,3,4}-generalized inverses of *AB*

As continuation of the preceding work, we obtain the following results regarding the three groups of set inclusions for $\left\{{\left(AB\right)}^{\left(1,2,3\right)}\right\}$, $\left\{{\left(AB\right)}^{\left(1,2,4\right)}\right\}$, $\left\{{\left(AB\right)}^{\left(1,3,4\right)}\right\}$ in (1.10) by applying Lemma 3.2, Lemma 3.3 to Theorem 9.3, Theorem 9.5. We omit their proofs due to space limitation.

Theorem 10.1 *Let* $A\in C^{m\times n}$ *and* $B\in C^{n\times p}$ *be given, and denote* M=AB *and* t=m+p+r(M)−r(A)−r(B)*. Then the following results hold.*

(a) *The following 3 statements are equivalent:*
  〈1〉 $\langle 1\rangle \;\{M^{\left(1,2,3\right)}\}∋B^{†}A^{†}.\;\;\langle 2\rangle \;\{M^{\left(1,2,3\right)}\}⊇\{B^{\left(1,2,3\right)}A^{†}\}.\;\;\langle 3\rangle \;\;R(A^{⁎}M)\subseteq R(B)$*.*
(b) *The following 3 statements are equivalent:*
  〈1〉 $\left\{M^{\left(1,2,3\right)}\}⊇\{B^{†}A^{\left(1,2,4\right)}\}.\;\;\;\;\;\;\;\;\langle 2\rangle \;\{M^{\left(1,2,3\right)}\}⊇\{B^{\left(1,2,3\right)}A^{\left(1,2,4\right)}\right\}$*.*
  〈3〉 $M=0\;\text{or}\;\{r(A)=m\;\text{and}\;R(A^{⁎}M)\subseteq R(B)\}$*.*
(c) *The following 3 statements are equivalent:*
  〈1〉 $\left\{M^{\left(1,2,3\right)}\}⊇\{B^{†}A^{\left(1,2,3\right)}\}.\;\;\;\;\;\;\;\;\langle 2\rangle \;\{M^{\left(1,2,3\right)}\}⊇\{B^{\left(1,2,3\right)}A^{\left(1,2,3\right)}\right\}$*.*
  〈3〉 $R(A^{⁎})\subseteq R(B)\;\text{or}\;R(A^{⁎}M)=R(B)$*.*
(d) *The following 3 statements are equivalent:*
  〈1〉 $\left\{M^{\left(1,2,3\right)}\}⊇\{B^{†}A^{\left(1,4\right)}\}.\;\;\;\;\;\;\;\;\;\;\;\langle 2\rangle \;\{M^{\left(1,2,3\right)}\}⊇\{B^{\left(1,2,3\right)}A^{\left(1,4\right)}\right\}$*.*
  〈3〉 $B=0\;\text{or}\;\{r(A)=m\;\text{and}\;R(A^{⁎}M)\subseteq R(B)\}$*.*
(e) *The following 3 statements are equivalent:*
  〈1〉 $\left\{M^{\left(1,2,3\right)}\}⊇\{B^{†}A^{\left(1,2\right)}\}.\;\;\;\;\;\;\;\;\;\;\;\langle 2\rangle \;\{M^{\left(1,2,3\right)}\}⊇\{B^{\left(1,2,3\right)}A^{\left(1,2\right)}\right\}$*.*
  〈3〉 $A=0\;\text{or}\;B=0\;\text{or}\;\{r(M)=m\;\text{and}\;R(A^{⁎})\subseteq R(B)\}$ *or* $\left\{r(A)=m\;\text{and}\;R(A^{⁎}M)=R(B)\right\}$*.*
(f) *The following 3 statements are equivalent:*
  〈1〉 $\left\{M^{\left(1,2,3\right)}\}⊇\{B^{\left(1,3,4\right)}A^{\left(1,2\right)}\}.\;\;\;\;\;\langle 2\rangle \;\{M^{\left(1,2,3\right)}\}⊇\{B^{\left(1,3\right)}A^{\left(1,2\right)}\right\}$*.*
  〈3〉 A=0 *or* $\left\{r(M)=m\;\text{and}\;R(A^{⁎})\subseteq R(B)\right\}$ *or* $\left\{r(A)=m\;\text{and}\;R(A^{⁎}M)=R(B)\right\}$*.*
(g) *The following 3 statements are equivalent:*
  〈1〉 $\left\{M^{\left(1,2,3\right)}\}⊇\{B^{\left(1,2,4\right)}A^{†}\}.\;\;\;\;\;\;\;\;\;\langle 2\rangle \;\{M^{\left(1,2,3\right)}\}⊇\{B^{\left(1,2\right)}A^{†}\right\}$*.*
  〈3〉 $B=0\;\text{or}\;R(A^{⁎})\subseteq R(B)$*.*
(h) *The following 3 statements are equivalent:*
  〈1〉 $\left\{M^{\left(1,2,3\right)}\}⊇\{B^{\left(1,4\right)}A^{†}\}.\;\;\;\;\;\;\;\;\;\langle 2\rangle \;\{M^{\left(1,2,3\right)}\}⊇\{B^{\left(1\right)}A^{†}\right\}$*.*
  〈3〉 $A=0\;\text{or}\;\{r(M)=p\;\text{and}\;R(A^{⁎})\subseteq R(B)\}$*.*
(i) *The following 3 statements are equivalent:*
  〈1〉 $\left\{M^{\left(1,2,3\right)}\}⊇\{B^{\left(1,2,4\right)}A^{\left(1,2,4\right)}\}.\;\;\;\;\;\langle 2\rangle \;\{M^{\left(1,2,3\right)}\}⊇\{B^{\left(1,2\right)}A^{\left(1,2,4\right)}\right\}$*.*
  〈3〉 $A=0\;\text{or}\;B=0\;\text{or}\;\{r(M)=m\;\text{and}\;R(A^{⁎})\subseteq R(B)\}$*.*
(j) *The following 3 statements are equivalent:*
  〈1〉 $\left\{M^{\left(1,2,3\right)}\}⊇\{B^{\left(1,4\right)}A^{\left(1,2,4\right)}\}.\;\;\;\;\;\;\langle 2\rangle \;\{M^{\left(1,2,3\right)}\}⊇\{B^{\left(1\right)}A^{\left(1,2,4\right)}\right\}$*.*
  〈3〉 $A=0\;\text{or}\;\{r(M)=m\;\text{and}\;R(A^{⁎})\subseteq R(B)\}$ *or* $\left\{r(M)=p\;\text{and}\;R(A^{⁎})\subseteq R(B)\right\}$*.*
(k) *The following 3 statements are equivalent:*
  〈1〉 $\left\{M^{\left(1,2,3\right)}\}⊇\{B^{\left(1,2,4\right)}A^{\left(1,4\right)}\}.\;\;\;\;\;\langle 2\rangle \;\{M^{\left(1,2,3\right)}\}⊇\{B^{\left(1,2\right)}A^{\left(1,4\right)}\right\}$*.*
  〈3〉 $B=0\;\text{or}\;\{r(M)=m\;\text{and}\;R(A^{⁎})\subseteq R(B)\}$*.*
(l) *The following 3 statements are equivalent:*
  〈1〉 $\left\{M^{\left(1,2,3\right)}\}⊇\{B^{\left(1,4\right)}A^{\left(1,4\right)}\}.\;\;\;\;\;\;\;\langle 2\rangle \;\{M^{\left(1,2,3\right)}\}⊇\{B^{\left(1\right)}A^{\left(1,4\right)}\right\}$*.*
  〈3〉 $r(A)=m,\;r(M)=p,\;\text{and}\;R(A^{⁎})\subseteq R(B)$*.*
(m) *The following 3 statements are equivalent:*
  〈1〉 $\left\{M^{\left(1,2,3\right)}\}⊇\{B^{\left(1,3,4\right)}A^{\left(1,2,3\right)}\}.\;\;\;\;\;\langle 2\rangle \;\{M^{\left(1,2,3\right)}\}⊇\{B^{\left(1,3\right)}A^{\left(1,2,3\right)}\right\}$*.*
  〈3〉 $R(A^{⁎})\subseteq R(B)\;\text{or}\;\{r(M)=p\;\text{and}\;R(A^{⁎}M)=R(B)\}$*.*
(n) *The following 3 statements are equivalent:*
  〈1〉 $\left\{M^{\left(1,2,3\right)}\}⊇\{B^{†}A^{\left(1,3\right)}\}.\;\;\;\;\;\;\;\;\langle 2\rangle \;\{M^{\left(1,2,3\right)}\}⊇\{B^{\left(1,2,3\right)}A^{\left(1,3\right)}\right\}$*.*
  〈3〉 $R(A^{⁎}M)=R(B)\;\text{or}\;\{r(A)=m\;\text{and}\;R(A^{⁎})\subseteq R(B)\}$*.*
(o) *The following 3 statements are equivalent:*
  〈1〉 $\left\{M^{\left(1,2,3\right)}\}⊇\{B^{†}A^{\left(1\right)}\}.\;\;\;\;\;\;\;\;\;\;\;\;\;\;\langle 2\rangle \;\{M^{\left(1,2,3\right)}\}⊇\{B^{\left(1,2,3\right)}A^{\left(1\right)}\right\}$*.*
  〈3〉 $\left\{r(M)=m\;\text{and}\;R(A^{⁎}M)\subseteq R(B)\right\}$ *or* $R(A^{⁎}M)=R(B)$*.*
(p) *The following 3 statements are equivalent:*
  〈1〉 $\left\{M^{\left(1,2,3\right)}\}⊇\{B^{\left(1,2,4\right)}A^{\left(1,3,4\right)}\}.\;\;\;\;\;\langle 2\rangle \;\{M^{\left(1,2,3\right)}\}⊇\{B^{\left(1,2\right)}A^{\left(1,3,4\right)}\right\}$*.*
  〈3〉 $B=0\;\text{or}\;r(M)=n\;\text{or}\;\{r(M)=m\;\text{and}\;R(A^{⁎})\subseteq R(B)\}$*.*
(q) *The following 3 statements are equivalent:*
  〈1〉 $\left\{M^{\left(1,2,3\right)}\}⊇\{B^{\left(1,4\right)}A^{\left(1,3,4\right)}\}.\;\;\;\;\;\;\;\langle 2\rangle \;\{M^{\left(1,2,3\right)}\}⊇\{B^{\left(1\right)}A^{\left(1,3,4\right)}\right\}$*.*
  〈3〉 $r(M)=n\;\text{or}\;\{r(A)=m\;\text{and}\;R(A^{⁎})\subseteq R(B)\}$*.*
(r) *The following 3 statements are equivalent:*
  〈1〉 $\left\{M^{\left(1,2,3\right)}\}⊇\{B^{\left(1,3,4\right)}A^{†}\}.\;\;\;\;\;\;\;\;\;\;\langle 2\rangle \;\{M^{\left(1,2,3\right)}\}⊇\{B^{\left(1,3\right)}A^{†}\right\}$*.*
  〈3〉 $R(A^{⁎})\subseteq R(B)\;\text{or}\;\{R(A^{⁎}M)\subseteq R(B)\;\text{and}\;r(B)=p\}$*.*
(s) *The following 3 statements are equivalent:*
  〈1〉 $\left\{M^{\left(1,2,3\right)}\}⊇\{B^{\left(1,3,4\right)}A^{\left(1,2,4\right)}\}.\;\;\;\;\;\langle 2\rangle \;\{M^{\left(1,2,3\right)}\}⊇\{B^{\left(1,3\right)}A^{\left(1,2,4\right)}\right\}$*.*
  〈3〉 $\left\{r(M)=m\;\text{and}\;R(A^{⁎})\subseteq R(B)\right\}$ *or* $\left\{r(A)=m,\;r(B)=p,\;\text{and}\;R(A^{⁎}M)\subseteq R(B)\right\}$*.*
(t) *The following 3 statements are equivalent:*
  〈1〉 $\left\{M^{\left(1,2,3\right)}\}⊇\{B^{†}A^{\left(1,3,4\right)}\}.\;\;\;\;\;\;\;\;\;\langle 2\rangle \;\{M^{\left(1,2,3\right)}\}⊇\{B^{\left(1,2,3\right)}A^{\left(1,3,4\right)}\right\}$*.*
  〈3〉 $R(A^{⁎}M)=R(B)$ *or* $\left\{r(A)=m\;\text{and}\;R(A^{⁎}M)\subseteq R(B)\right\}$*.*
(u) *The following 3 statements are equivalent:*
  〈1〉 $\left\{M^{\left(1,2,3\right)}\}⊇\{B^{\left(1,3,4\right)}A^{\left(1,3\right)}\}.\;\;\;\;\;\;\langle 2\rangle \;\{M^{\left(1,2,3\right)}\}⊇\{B^{\left(1,3\right)}A^{\left(1,3\right)}\right\}$*.*
  〈3〉 $r(M)=\min\{m,\;n,\;p\}\;\text{and}\;R(A^{⁎}M)\subseteq R(B)$*.*
(v) *The following 3 statements are equivalent:*
  〈1〉 $\left\{M^{\left(1,2,3\right)}\}⊇\{B^{\left(1,3,4\right)}A^{\left(1\right)}\}.\;\;\;\;\;\;\;\langle 2\rangle \;\;\{M^{\left(1,2,3\right)}\}⊇\{B^{\left(1,3\right)}A^{\left(1\right)}\right\}$*.*
  〈3〉 $\left\{r(M)=m\;\text{and}\;R(A^{⁎}M)\subseteq R(B)\}\;\text{or}\;\{r(A)=m,\;r(M)=p,\;\text{and}\;R(A^{⁎}M)\subseteq R(B)\right\}$*.*
(w) *The following 3 statements are equivalent:*
  〈1〉 $\left\{M^{\left(1,2,3\right)}\}⊇\{B^{\left(1,3,4\right)}A^{\left(1,3,4\right)}\}.\;\;\;\;\;\langle 2\rangle \;\{M^{\left(1,2,3\right)}\}⊇\{B^{\left(1,3\right)}A^{\left(1,3,4\right)}\right\}$*.*
  〈3〉 $r(M)=\min\{m,\;n,\;p,\;t\}\;\text{and}\;R(A^{⁎}M)\subseteq R(B)$*.*
(x) *The following 3 statements are equivalent:*
  〈1〉 $\left\{M^{\left(1,2,3\right)}\}⊇\{B^{\left(1,3,4\right)}A^{\left(1,4\right)}\}.\;\;\;\;\;\;\langle 2\rangle \;\{M^{\left(1,2,3\right)}\}⊇\{B^{\left(1,3\right)}A^{\left(1,4\right)}\right\}$*.*
  〈3〉 $\left\{r(M)=m\;\text{and}\;R(A^{⁎}M)\subseteq R(B)\right\}$ *or* $\left\{r(M)=p\;\text{and}\;R(A^{⁎}M)\subseteq R(B)\right\}$ *or* $\left\{r(A)=m,\;r(B)=p,\;\text{and}\;R(A^{⁎}M)\subseteq R(B\right)$*.*
(y) *The following 3 statements are equivalent:*
  〈1〉 $\left\{M^{\left(1,2,3\right)}\}⊇\{B^{\left(1,2,4\right)}A^{\left(1,2,3\right)}\}\;\;\;\;\;\langle 2\rangle \;\{M^{\left(1,2,3\right)}\}⊇\{B^{\left(1,2\right)}A^{\left(1,2,3\right)}\right\}$*.*
  〈3〉 A=0orB=0orr(B)=n*.*
(z) *The following 3 statements are equivalent:*
  〈1〉 $\left\{M^{\left(1,2,3\right)}\}⊇\{B^{\left(1,2,4\right)}A^{\left(1,3\right)}\}.\;\;\;\;\;\langle 2\rangle \;\{M^{\left(1,2,3\right)}\}⊇\{B^{\left(1,2\right)}A^{\left(1,3\right)}\right\}$*.*
  〈3〉 B=0 *or* r(M)=n *or* {r(A)=mandr(B)=n}*.*
(a1) *The following 3 statements are equivalent:*
  〈1〉 $\left\{M^{\left(1,2,3\right)}\}⊇\{B^{\left(1,4\right)}A^{\left(1,2,3\right)}\}.\;\;\;\;\;\langle 2\rangle \;\{M^{\left(1,2,3\right)}\}⊇\{B^{\left(1\right)}A^{\left(1,2,3\right)}\right\}$*.*
  〈3〉 A=0 *or* r(B)=n*.*
(b1) *The following 3 statements are equivalent:*
  〈1〉 $\left\{M^{\left(1,2,3\right)}\}⊇\{B^{\left(1,4\right)}A^{\left(1,3\right)}\}.\;\;\;\;\;\;\;\langle 2\rangle \;\{M^{\left(1,2,3\right)}\}⊇\{B^{\left(1\right)}A^{\left(1,3\right)}\right\}$*.*
  〈3〉 r(M)=n *or* {r(A)=mandr(B)=n}*.*
(c1) *The following 3 statements are equivalent:*
  〈1〉 $\left\{M^{\left(1,2,3\right)}\}⊇\{B^{\left(1,2,4\right)}A^{\left(1,2\right)}\}.\;\;\;\;\;\;\langle 2\rangle \;\{M^{\left(1,2,3\right)}\}⊇\{B^{\left(1,2\right)}A^{\left(1,2\right)}\right\}$*.*
  〈3〉 A=0 *or* B=0 *or* {r(A)=m *and* r(B)=n}*.*
(d1) *The following 3 statements are equivalent:*
  〈1〉 $\left\{M^{\left(1,2,3\right)}\}⊇\{B^{\left(1,2,4\right)}A^{\left(1\right)}\}.\;\;\;\;\;\;\;\langle 2\rangle \;\{M^{\left(1,2,3\right)}\}⊇\{B^{\left(1,2\right)}A^{\left(1\right)}\right\}$*.*
  〈3〉 B=0 *or* {r(A)=m *and* r(B)=n}*.*
(e1) *The following 3 statements are equivalent:*
  〈1〉 $\left\{M^{\left(1,2,3\right)}\}⊇\{B^{\left(1,4\right)}A^{\left(1,2\right)}\}.\;\;\;\;\;\;\;\langle 2\rangle \;\{M^{\left(1,2,3\right)}\}⊇\{B^{\left(1\right)}A^{\left(1,2\right)}\right\}$*.*
  〈3〉 A=0 *or* {r(A)=m *and* r(B)=n}*.*
(f1) *The following 3 statements are equivalent:*
  〈1〉 $\left\{M^{\left(1,2,3\right)}\}⊇\{B^{\left(1,4\right)}A^{\left(1\right)}\}.\;\;\;\;\;\;\;\;\langle 2\rangle \;\{M^{\left(1,2,3\right)}\}⊇\{B^{\left(1\right)}A^{\left(1\right)}\right\}$*.*
  〈3〉 r(A)=m *and* r(B)=n*.*

Theorem 10.2 *Let* $A\in C^{m\times n}$ *and* $B\in C^{n\times p}$ *be given, and denote* M=AB *and* t=m+p+r(M)−r(A)−r(B)*. Then the following results hold.*

(a) *The following 3 statements are equivalent:*
  〈1〉 $\left\{M^{\left(1,2,4\right)}\}∋B^{†}A^{†}.\;\;\langle 2\rangle \;\{M^{\left(1,2,4\right)}\}⊇\{A^{†}B^{\left(1,2,4\right)}\}.\;\;\langle 3\rangle \;R(BM^{⁎})\subseteq R(A^{⁎}\right)$*.*
(b) *The following 3 statements are equivalent:*
  〈1〉 $\left\{M^{\left(1,2,4\right)}\}⊇\{B^{\left(1,2,4\right)}A^{†}\}.\;\;\;\;\;\;\;\;\;\;\langle 2\rangle \;\{M^{\left(1,2,4\right)}\}⊇\{B^{\left(1,2,4\right)}A^{\left(1,2,4\right)}\right\}$*.*
  〈3〉 $R(B)\subseteq R(A^{⁎})\;\text{or}\;R(BM^{⁎})=R(A^{⁎})$*.*
(c) *The following 3 statements are equivalent:*
  〈1〉 $\left\{M^{\left(1,2,4\right)}\}⊇\{B^{\left(1,2,3\right)}A^{†}\}.\;\;\;\;\;\;\;\;\;\;\langle 2\rangle \;\{M^{\left(1,2,4\right)}\}⊇\{B^{\left(1,2,3\right)}A^{\left(1,2,4\right)}\right\}$*.*
  〈3〉 $M=0\;\text{or}\;\{r(B)=p\;\text{and}\;R(BM^{⁎})\subseteq R(A^{⁎})\}$*.*
(d) *The following 3 statements are equivalent:*
  〈1〉 $\left\{M^{\left(1,2,4\right)}\}⊇\{B^{\left(1,3\right)}A^{†}\}.\;\;\;\;\;\;\;\;\;\;\;\;\langle 2\rangle \;\{M^{\left(1,2,4\right)}\}⊇\{B^{\left(1,3\right)}A^{\left(1,2,4\right)}\right\}$*.*
  〈3〉 $A=0\;\text{or}\;\{r(B)=p\;\text{and}\;R(BM^{⁎})\subseteq R(A^{⁎})\}$*.*
(e) *The following 3 statements are equivalent:*
  〈1〉 $\left\{M^{\left(1,2,4\right)}\}⊇\{B^{\left(1,2\right)}A^{†}\}.\;\;\;\;\;\;\;\;\;\;\;\;\langle 2\rangle \;\{M^{\left(1,2,4\right)}\}⊇\{B^{\left(1,2\right)}A^{\left(1,2,4\right)}\right\}$*.*
  〈3〉 $A=0\;\text{or}\;B=0\;\text{or}\;\{r(M)=p\;\text{and}\;R(B)\subseteq R(A^{⁎})\}$ *or* $\left\{r(B)=p\;\text{and}\;R(BM^{⁎})=R(A^{⁎})\right\}$*.*
(f) *The following 3 statements are equivalent:*
  〈1〉 $\left\{M^{\left(1,2,4\right)}\}⊇\{B^{\left(1,2\right)}A^{\left(1,3,4\right)}\}.\;\;\;\;\;\;\;\;\langle 2\rangle \;\{M^{\left(1,2,4\right)}\}⊇\{B^{\left(1,2\right)}A^{\left(1,4\right)}\right\}$*.*
  〈3〉 B=0 *or* $\left\{r(M)=p\;\text{and}\;R(B)\subseteq R(A^{⁎})\right\}$ *or* $\left\{r(B)=p\;\text{and}\;R(BM^{⁎})=R(A^{⁎})\right\}$*.*
(g) *The following 3 statements are equivalent:*
  〈1〉 $\left\{M^{\left(1,2,4\right)}\}⊇\{B^{†}A^{\left(1,2,3\right)}\}.\;\;\;\;\;\;\;\;\;\;\;\langle 2\rangle \;\{M^{\left(1,2,4\right)}\}⊇\{B^{†}A^{\left(1,2\right)}\right\}$*.*
  〈3〉 $A=0\;\text{or}\;R(B)\subseteq R(A^{⁎})$*.*
(h) *The following 3 statements are equivalent:*
  〈1〉 $\left\{M^{\left(1,2,4\right)}\}⊇\{B^{†}A^{\left(1,3\right)}\}.\;\;\;\;\;\;\;\;\;\;\;\;\;\langle 2\rangle \;\{M^{\left(1,2,4\right)}\}⊇\{B^{†}A^{\left(1\right)}\right\}$*.*
  〈3〉 $B=0\;\text{or}\;\{r(M)=m\;\text{and}\;R(B)\subseteq R(A^{⁎})\}$*.*
(i) *The following 3 statements are equivalent:*
  〈1〉 $\left\{M^{\left(1,2,4\right)}\}⊇\{B^{\left(1,2,3\right)}A^{\left(1,2,3\right)}\}.\;\;\;\;\;\;\;\langle 2\rangle \;\{M^{\left(1,2,4\right)}\}⊇\{B^{\left(1,2,3\right)}A^{\left(1,2\right)}\right\}$*.*
  〈3〉 $A=0\;\text{or}\;B=0\;\text{or}\;\{r(M)=p\;\text{and}\;R(B)\subseteq R(A^{⁎})\}$*.*
(j) *The following 3 statements are equivalent:*
  〈1〉 $\left\{M^{\left(1,2,4\right)}\}⊇\{B^{\left(1,2,3\right)}A^{\left(1,3\right)}\}.\;\;\;\;\;\;\;\;\langle 2\rangle \;\{M^{\left(1,2,4\right)}\}⊇\{B^{\left(1,2,3\right)}A^{\left(1\right)}\right\}$*.*
  〈3〉 $B=0\;\text{or}\;\{r(M)=p\;\text{and}\;R(B)\subseteq R(A^{⁎})\}$ *or* $\left\{r(M)=m\;\text{and}\;R(B)\subseteq R(A^{⁎})\right\}$*.*
(k) *The following 3 statements are equivalent:*
  〈1〉 $\left\{M^{\left(1,2,4\right)}\}⊇\{B^{\left(1,3\right)}A^{\left(1,2,3\right)}\}.\;\;\;\;\;\;\;\langle 2\rangle \;\{M^{\left(1,2,4\right)}\}⊇\{B^{\left(1,3\right)}A^{\left(1,2\right)}\right\}$*.*
  〈3〉 $A=0\;\text{or}\;\{r(M)=p\;\text{and}\;R(B)\subseteq R(A^{⁎})\}$*.*
(l) *The following 3 statements are equivalent:*
  〈1〉 $\left\{M^{\left(1,2,4\right)}\}⊇\{B^{\left(1,3\right)}A^{\left(1,3\right)}\}.\;\;\;\;\;\;\;\;\langle 2\rangle \;\{M^{\left(1,2,4\right)}\}⊇\{B^{\left(1,3\right)}A^{\left(1\right)}\right\}$*.*
  〈3〉 $r(B)=p,\;r(M)=m,\;\text{and}\;R(B)\subseteq R(A^{⁎})$*.*
(m) *The following 3 statements are equivalent:*
  〈1〉 $\left\{M^{\left(1,2,4\right)}\}⊇\{B^{\left(1,2,4\right)}A^{\left(1,3,4\right)}\}.\;\;\;\;\;\langle 2\rangle \;\{M^{\left(1,2,4\right)}\}⊇\{B^{\left(1,2,4\right)}A^{\left(1,4\right)}\right\}$*.*
  〈3〉 $R(B)\subseteq R(A^{⁎})\;\text{or}\;\{r(M)=m\;\text{and}\;R(BM^{⁎})=R(A^{⁎})\}$*.*
(n) *The following 3 statements are equivalent:*
  〈1〉 $\left\{M^{\left(1,2,4\right)}\}⊇\{B^{\left(1,4\right)}A^{†}\}.\;\;\;\;\;\;\;\;\;\;\;\langle 2\rangle \;\{M^{\left(1,2,4\right)}\}⊇\{B^{\left(1,4\right)}A^{\left(1,2,4\right)}\right\}$*.*
  〈3〉 $R(BM^{⁎})=R(A^{⁎})\;\text{or}\;\{r(B)=p\;\text{and}\;R(B)\subseteq R(A^{⁎})\}$*.*
(o) *The following 3 statements are equivalent:*
  〈1〉 $\left\{M^{\left(1,2,4\right)}\}⊇\{B^{\left(1\right)}A^{†}\}.\;\;\;\;\;\;\;\;\;\;\;\;\;\langle 2\rangle \;\{M^{\left(1,2,4\right)}\}⊇\{B^{\left(1\right)}A^{\left(1,2,4\right)}\right\}$*.*
  〈3〉 $\left\{r(M)=p\;\text{and}\;R(BM^{⁎})\subseteq R(A^{⁎})\right\}$ *or* $R(BM^{⁎})=R(A^{⁎})$*.*
(p) *The following 3 statements are equivalent:*
  〈1〉 $\left\{M^{\left(1,2,4\right)}\}⊇\{B^{\left(1,3,4\right)}A^{\left(1,2,3\right)}\}.\;\;\;\;\;\;\langle 2\rangle \;\{M^{\left(1,2,4\right)}\}⊇\{B^{\left(1,3,4\right)}A^{\left(1,2\right)}\right\}$*.*
  〈3〉 $A=0\;\text{or}\;r(M)=n\;\text{or}\;\{r(M)=p\;\text{and}\;R(B)\subseteq R(A^{⁎})\}$*.*
(q) *The following 3 statements are equivalent:*
  〈1〉 $\left\{M^{\left(1,2,4\right)}\}⊇\{B^{\left(1,3,4\right)}A^{\left(1,3\right)}\}.\;\;\;\;\;\;\;\;\langle 2\rangle \;\{M^{\left(1,2,4\right)}\}⊇\{B^{\left(1,3,4\right)}A^{\left(1\right)}\right\}$*.*
  〈3〉 $r(M)=n\;\text{or}\;\{r(B)=p\;\text{and}\;R(B)\subseteq R(A^{⁎})\}$*.*
(r) *The following 3 statements are equivalent:*
  〈1〉 $\left\{M^{\left(1,2,4\right)}\}⊇\{B^{†}A^{\left(1,3,4\right)}\}.\;\;\;\;\;\;\;\;\;\;\;\langle 2\rangle \;\{M^{\left(1,2,4\right)}\}⊇\{B^{†}A^{\left(1,4\right)}A\right\}$*.*
  〈3〉 $R(B^{†})\subseteq R(A^{⁎})\;\text{or}\;\{R(BM^{⁎})\subseteq R(A^{⁎})\;\text{and}\;r(A)=m\}$*.*
(s) *The following 3 statements are equivalent:*
  〈1〉 $\left\{M^{\left(1,2,4\right)}\}⊇\{B^{\left(1,2,3\right)}A^{\left(1,3,4\right)}\}.\;\;\;\;\;\;\langle 2\rangle \;\{M^{\left(1,2,4\right)}\}⊇\{B^{\left(1,2,3\right)}A^{\left(1,4\right)}\right\}$*.*
  〈3〉 $\left\{r(M)=p\;\text{and}\;R(B)\subseteq R(A^{⁎})\right\}$ *or* $\left\{r(A)=m,\;r(B)=p,\;\text{and}\;R(BM^{⁎})\subseteq R(A^{⁎})\right\}$*.*
(t) *The following 3 statements are equivalent:*
  〈1〉 $\left\{M^{\left(1,2,4\right)}\}⊇\{B^{\left(1,3,4\right)}A^{†}\}.\;\;\;\;\;\;\;\;\;\;\;\langle 2\rangle \;\{M^{\left(1,2,4\right)}\}⊇\{B^{\left(1,3,4\right)}A^{\left(1,2,4\right)}\right\}$*.*
  〈3〉 $R(A^{⁎}M)=R(B)$ *or* $\left\{r(B)=p\;\text{and}\;R(BM^{⁎})\subseteq R(A^{⁎})\right\}$*.*
(u) *The following 3 statements are equivalent:*
  〈1〉 $\left\{M^{\left(1,2,4\right)}\}⊇\{B^{\left(1,4\right)}A^{\left(1,3,4\right)}\}.\;\;\;\;\;\;\langle 2\rangle \;\{M^{\left(1,2,4\right)}\}⊇\{B^{\left(1,4\right)}A^{\left(1,4\right)}\right\}$*.*
  〈3〉 $r(M)=\min\{m,\;n,\;p\}\;\text{and}\;R(BM^{⁎})\subseteq R(A^{⁎})$*.*
(v) *The following 3 statements are equivalent:*
  〈1〉 $\left\{M^{\left(1,2,4\right)}\}⊇\{B^{\left(1\right)}A^{\left(1,3,4\right)}\}.\;\;\;\;\;\;\;\;\langle 2\rangle \;\;\{M^{\left(1,2,4\right)}\}⊇\{B^{\left(1\right)}A^{\left(1,4\right)}\right\}$*.*
  〈3〉 $\left\{r(M)=p\;\text{and}\;R(BM^{⁎})\subseteq R(A^{⁎})\}\;\text{or}\;\{r(B)=p,\;r(M)=m,\;\text{and}\;R(BM^{⁎})\subseteq R(A^{⁎})\right\}$*.*
(w) *The following 3 statements are equivalent:*
  〈1〉 $\left\{M^{\left(1,2,4\right)}\}⊇\{B^{\left(1,3,4\right)}A^{\left(1,3,4\right)}\}.\;\;\;\;\;\langle 2\rangle \;\{M^{\left(1,2,4\right)}\}⊇\{B^{\left(1,3,4\right)}A^{\left(1,4\right)}\right\}$*.*
  〈3〉 $r(M)=\min\{m,\;n,\;p,\;t\}\;\text{and}\;R(BM^{⁎})\subseteq R(A^{⁎})$*.*
(x) *The following 3 statements are equivalent:*
  〈1〉 $\left\{M^{\left(1,2,4\right)}\}⊇\{B^{\left(1,3\right)}A^{\left(1,3,4\right)}\}.\;\;\;\;\;\;\;\langle 2\rangle \;\{M^{\left(1,2,4\right)}\}⊇\{B^{\left(1,3\right)}A^{\left(1,4\right)}\right\}$*.*
  〈3〉 $\left\{r(M)=p\;\text{and}\;R(BM^{⁎})\subseteq R(A^{⁎})\right\}$ *or* $\left\{r(M)=m\;\text{and}\;R(BM^{⁎})\subseteq R(A^{⁎})\right\}$ *or* $\left\{r(A)=m,\;r(B)=p,\;\text{and}\;R(BM^{⁎})\subseteq R(A^{⁎}\right)$*.*
(y) *The following 3 statements are equivalent:*
  〈1〉 $\left\{M^{\left(1,2,4\right)}\}⊇\{B^{\left(1,2,4\right)}A^{\left(1,2,3\right)}\}\;\;\;\;\;\;\langle 2\rangle \;\{M^{\left(1,2,4\right)}\}⊇\{B^{\left(1,2,4\right)}A^{\left(1,2\right)}\right\}$*.*
  〈3〉 A=0orB=0orr(A)=n*.*
(z) *The following 3 statements are equivalent:*
  〈1〉 $\left\{M^{\left(1,2,4\right)}\}⊇\{B^{\left(1,4\right)}A^{\left(1,2,3\right)}\}.\;\;\;\;\;\;\langle 2\rangle \;\{M^{\left(1,2,4\right)}\}⊇\{B^{\left(1,4\right)}A^{\left(1,2\right)}\right\}$*.*
  〈3〉 A=0 *or* r(M)=n *or* {r(A)=nandr(B)=p}*.*
(a1) *The following 3 statements are equivalent:*
  〈1〉 $\left\{M^{\left(1,2,4\right)}\}⊇\{B^{\left(1,2,4\right)}A^{\left(13\right)}\}.\;\;\;\;\;\;\langle 2\rangle \;\{M^{\left(1,2,4\right)}\}⊇\{B^{\left(1,2,4\right)}A^{\left(1\right)}\right\}$*.*
  〈3〉 r(A)=n *or* B=0*.*
(b1) *The following 3 statements are equivalent:*
  〈1〉 $\left\{M^{\left(1,2,4\right)}\}⊇\{B^{\left(1,4\right)}A^{\left(1,3\right)}\}.\;\;\;\;\;\;\;\langle 2\rangle \;\{M^{\left(1,2,4\right)}\}⊇\{B^{\left(1,4\right)}A^{\left(1\right)}\right\}$*.*
  〈3〉 r(M)=n *or* {r(A)=nandr(B)=p}*.*
(c1) *The following 3 statements are equivalent:*
  〈1〉 $\left\{M^{\left(1,2,4\right)}\}⊇\{B^{\left(1,2\right)}A^{\left(1,2,3\right)}\}.\;\;\;\;\;\;\langle 2\rangle \;\{M^{\left(1,2,4\right)}\}⊇\{B^{\left(1,2\right)}A^{\left(1,2\right)}\right\}$*.*
  〈3〉 A=0 *or* B=0 *or* {r(A)=n *and* r(B)=p}*.*
(d1) *The following 3 statements are equivalent:*
  〈1〉 $\left\{M^{\left(1,2,4\right)}\}⊇\{B^{\left(1,\right)}A^{\left(1,2,3\right)}\}.\;\;\;\;\;\;\langle 2\rangle \;\{M^{\left(1,2,4\right)}\}⊇\{B^{\left(1\right)}A^{\left(1,2\right)}\right\}$*.*
  〈3〉 A=0 *or* {r(A)=n *and* r(B)=p}*.*
(e1) *The following 3 statements are equivalent:*
  〈1〉 $\left\{M^{\left(1,2,4\right)}\}⊇\{B^{\left(1,2\right)}A^{\left(1,3\right)}\}.\;\;\;\;\;\;\;\langle 2\rangle \;\{M^{\left(1,2,4\right)}\}⊇\{B^{\left(1,2\right)}A^{\left(1\right)}\right\}$*.*
  〈3〉 B=0 *or* {r(A)=n *and* r(B)=p}*.*
(f1) *The following 3 statements are equivalent:*
  〈1〉 $\left\{M^{\left(1,2,4\right)}\}⊇\{B^{\left(1\right)}A^{\left(1,3\right)}\}.\;\;\;\;\;\;\;\;\langle 2\rangle \;\{M^{\left(1,2,4\right)}\}⊇\{B^{\left(1\right)}A^{\left(1\right)}\right\}$*.*
  〈3〉 r(A)=n *and* r(B)=p*.*

Theorem 10.3 *Let* $A\in C^{m\times n}$ *and* $B\in C^{n\times p}$ *be given, and denote* M=AB*. Then the following results hold.*

(a) *The following 5 statements are equivalent:*
  〈1〉 $\left\{M^{\left(1,3,4\right)}\}∋B^{†}A^{†}.\;\;\;\;\;\;\;\;\;\;\;\;\;\;\;\;\;\langle 2\rangle \;\{M^{\left(1,3,4\right)}\}⊇\{B^{†}A^{\left(1,3,4\right)}\right\}$*.*
  〈3〉 $\left\{M^{\left(1,3,4\right)}\}⊇\{B^{\left(1,3,4\right)}A^{†}\}.\;\;\;\;\;\;\;\;\;\langle 4\rangle \;\{M^{\left(1,3,4\right)}\}⊇\{B^{\left(1,3,4\right)}A^{\left(1,3,4\right)}\right\}$*.*
  〈5〉 $R(A^{⁎}M)\subseteq R(B)\;\text{and}\;R(BM^{⁎})\subseteq R(A^{⁎})$*.*
(b) *The following 3 statements are equivalent:*
  〈1〉 $\left\{M^{\left(1,3,4\right)}\}⊇\{B^{†}A^{\left(1,2,4\right)}\}.\;\;\;\;\;\;\;\;\langle 2\rangle \;\;\{M^{\left(1,3,4\right)}\}⊇\{B^{†}A^{\left(1,4\right)}\right\}$*.*
  〈3〉 M=0 *or* $\left\{r(A)=m,\;R(A^{⁎}M)\subseteq R(B),\;\text{and}\;R(BM^{⁎})\subseteq R(A^{⁎})\right\}$*.*
(c) *The following 3 statements are equivalent:*
  〈1〉 $\left\{M^{\left(1,3,4\right)}\}⊇\{B^{\left(1,3,4\right)}A^{\left(1,2,4\right)}\}.\;\;\;\langle 2\rangle \;\;\{M^{\left(1,3,4\right)}\}⊇\{B^{\left(1,3,4\right)}A^{\left(1,4\right)}\right\}$*.*
  〈3〉 M=0 *or* $\left\{r(A)=m,\;R(A^{⁎}M)\subseteq R(B),\;\text{and}\;R(BM^{⁎})\subseteq R(A^{⁎})\right\}$*.*
(d) *The following 3 statements are equivalent:*
  〈1〉 $\left\{M^{\left(1,3,4\right)}\}⊇\{B^{\left(1,2,4\right)}A^{†}\}.\;\;\;\;\;\;\;\;\langle 2\rangle \;\;\{M^{\left(1,3,4\right)}\}⊇\{B^{\left(1,4\right)}A^{†}\right\}$*.*
  〈3〉 $M=0\;\text{or}\;R(BM^{⁎})=R(A^{⁎})$*.*
(e) *The following 3 statements are equivalent:*
  〈1〉 $\left\{M^{\left(1,3,4\right)}\}⊇\{B^{†}A^{\left(1,2,3\right)}\}.\;\;\;\;\;\;\;\;\langle 2\rangle \;\;\{M^{\left(1,3,4\right)}\}⊇\{B^{†}A^{\left(1,3\right)}\right\}$*.*
  〈3〉 $M=0\;\text{or}\;R(A^{⁎}M)=R(B)$*.*
(f) *The following 3 statements are equivalent:*
  〈1〉 $\left\{M^{\left(1,3,4\right)}\}⊇\{B^{\left(1,2,4\right)}A^{\left(1,3,4\right)}\}.\;\;\;\;\langle 2\rangle \;\;\{M^{\left(1,3,4\right)}\}⊇\{B^{\left(1,4\right)}A^{\left(1,3,4\right)}\right\}$*.*
  〈3〉 M=0 *or* $\left\{r(B)=n\;\text{and}\;R(BM^{⁎})\subseteq R(A^{⁎})\right\}$ *or* $\left\{r(A)=m\;\text{and}\;R(BM^{⁎})=R(A^{⁎})\right\}$*.*
(g) *The following 5 statements are equivalent:*
  〈1〉 $\left\{M^{\left(1,3,4\right)}\}⊇\{B^{\left(1,2,4\right)}A^{\left(1,2,4\right)}\}.\;\;\;\;\langle 2\rangle \;\{M^{\left(1,3,4\right)}\}⊇\{B^{\left(1,2,4\right)}A^{\left(1,4\right)}\right\}$*.*
  〈3〉 $\left\{M^{\left(1,3,4\right)}\}⊇\{B^{\left(1,4\right)}A^{\left(1,2,4\right)}\}.\;\;\;\;\;\;\langle 4\rangle \;\{M^{\left(1,3,4\right)}\}⊇\{B^{\left(1,4\right)}A^{\left(1,4\right)}\right\}$*.*
  〈5〉 $M=0\;\text{or}\;\{r(A)=m\;\text{and}\;R(BM^{⁎})=R(A^{⁎})\}$*.*
(h) *The following 5 statements are equivalent:*
  〈1〉 $\left\{M^{\left(1,3,4\right)}\}⊇\{B^{\left(1,2,3\right)}A^{†}\}.\;\;\;\;\;\;\;\;\;\langle 2\rangle \;\{M^{\left(1,3,4\right)}\}⊇\{B^{\left(1,2,3\right)}A^{\left(1,3,4\right)}\right\}$*.*
  〈3〉 $\left\{M^{\left(1,3,4\right)}\}⊇\{B^{\left(1,3\right)}A^{†}\}.\;\;\;\;\;\;\;\;\;\;\;\langle 4\rangle \;\{M^{\left(1,3,4\right)}\}⊇\{B^{\left(1,3\right)}A^{\left(1,3,4\right)}\right\}$*.*
  〈5〉 $M=0\;\text{or}\;\{r(B)=p,\;R(A^{⁎}M)\subseteq R(B),\;\text{and}\;R(BM^{⁎})\subseteq R(A^{⁎})\}$*.*
(i) *The following 5 statements are equivalent:*
  〈1〉 $\left\{M^{\left(1,3,4\right)}\}⊇\{B^{\left(1,2,3\right)}A^{\left(1,2,4\right)}\}.\;\;\;\;\langle 2\rangle \;\{M^{\left(1,3,4\right)}\}⊇\{B^{\left(1,2,3\right)}A^{\left(1,4\right)}\right\}$*.*
  〈3〉 $\left\{M^{\left(1,3,4\right)}\}⊇\{B^{\left(1,3\right)}A^{\left(1,2,4\right)}\}.\;\;\;\;\;\;\langle 4\rangle \;\{M^{\left(1,3,4\right)}\}⊇\{B^{\left(1,3\right)}A^{\left(1,4\right)}\right\}$*.*
  〈5〉 $M=0\;\text{or}\;\{r(A)=m,\;r(B)=p,\;R(A^{⁎}M)\subseteq R(B),\;\text{and}\;R(BM^{⁎})\subseteq R(A^{⁎})\}$*.*
(j) *The following 3 statements are equivalent:*
  〈1〉 $\left\{M^{\left(1,3,4\right)}\}⊇\{B^{\left(1,2\right)}A^{†}\}.\;\;\;\;\;\;\;\;\;\;\;\langle 2\rangle \;\;\{M^{\left(1,3,4\right)}\}⊇\{B^{\left(1\right)}A^{†}\right\}$*.*
  〈3〉 M=0 *or* $\left\{r(B)=p\;\text{and}\;R(BM^{⁎})=R(A^{⁎})\right\}$*.*
(k) *The following 3 statements are equivalent:*
  〈1〉 $\left\{M^{\left(1,3,4\right)}\}⊇\{B^{\left(1,2\right)}A^{\left(1,3,4\right)}\}.\;\;\;\;\;\langle 2\rangle \;\;\{M^{\left(1,3,4\right)}\}⊇\{B^{\left(1\right)}A^{\left(1,3,4\right)}\right\}$*.*
  〈3〉 M=0 *or* $\left\{r(B)=n=p\;\text{and}\;R(BM^{⁎})\subseteq R(A^{⁎})\right\}$ *or* $\left\{r(A)=m,\;r(B)=p,\;\text{and}\;R(BM^{⁎})=R(A^{⁎})\right\}$*.*
(l) *The following 5 statements are equivalent:*
  〈1〉 $\left\{M^{\left(1,3,4\right)}\}⊇\{B^{\left(1,2\right)}A^{\left(1,2,4\right)}\}.\;\;\;\;\;\langle 2\rangle \;\{M^{\left(1,3,4\right)}\}⊇\{B^{\left(1,2\right)}A^{\left(1,4\right)}\right\}$*.*
  〈3〉 $\left\{M^{\left(1,3,4\right)}\}⊇\{B^{\left(1\right)}A^{\left(1,2,4\right)}\}.\;\;\;\;\;\;\;\langle 4\rangle \;\{M^{\left(1,3,4\right)}\}⊇\{B^{\left(1\right)}A^{\left(1,4\right)}\right\}$*.*
  〈5〉 $M=0\;\text{or}\;\{r(A)=m,\;r(B)=p,\;\text{and}\;R(BM^{⁎})=R(A^{⁎})\}$*.*
(m) *The following 5 statements are equivalent:*
  〈1〉 $\left\{M^{\left(1,3,4\right)}\}⊇\{B^{\left(1,2,3\right)}A^{\left(1,2\right)}\}.\;\;\;\;\langle 2\rangle \;\{M^{\left(1,3,4\right)}\}⊇\{B^{\left(1,2,3\right)}A^{\left(1\right)}\right\}$*.*
  〈3〉 $\left\{M^{\left(1,3,4\right)}\}⊇\{B^{\left(1,3\right)}A^{\left(1,2\right)}\}.\;\;\;\;\;\;\langle 3\rangle \;\{M^{\left(1,3,4\right)}\}⊇\{B^{\left(1,3\right)}A^{\left(1\right)}\right\}$*.*
  〈5〉 $M=0\;\text{or}\;\{r(A)=m,\;r(B)=p,\;\text{and}\;R(A^{⁎}M)=R(B)\}$*.*
(n) *The following 3 statements are equivalent:*
  〈1〉 $\left\{M^{\left(1,3,4\right)}\}⊇\{B^{†}A^{\left(1,2\right)}\}.\;\;\;\;\;\;\;\;\;\;\langle 2\rangle \;\{M^{\left(1,3,4\right)}\}⊇\{B^{†}A^{\left(1\right)}\right\}$*.*
  〈3〉 $M=0\;\text{or}\;\{r(A)=m\;\text{and}\;R(A^{⁎}M)=R(B)$*.*
(o) *The following 3 statements are equivalent:*
  〈1〉 $\left\{M^{\left(1,3,4\right)}\}⊇\{B^{\left(1,3,4\right)}A^{\left(1,2,3\right)}\}.\;\;\;\;\;\;\langle 2\rangle \;\{M^{\left(1,3,4\right)}\}⊇\{B^{\left(1,3,4\right)}A^{\left(1,3\right)}\right\}$*.*
  〈3〉 $M=0\;\text{or}\;\{r(A)=n\;\text{and}\;R(A^{⁎}M)\subseteq R(B)\}$ *or* $\left\{r(B)=p\;\text{and}\;R(A^{⁎}M)=R(B)\right\}$*.*
(p) *The following 3 statements are equivalent:*
  〈1〉 $\left\{M^{\left(1,3,4\right)}\}⊇\{B^{\left(1,3,4\right)}A^{\left(1,2\right)}\}.\;\;\;\;\;\;\;\langle 2\rangle \;\{M^{\left(1,3,4\right)}\}⊇\{B^{\left(1,3,4\right)}A^{\left(1\right)}\right\}$*.*
  〈3〉 $M=0\;\text{or}\;\{r(A)=m=n\;\text{and}\;R(A^{⁎}M)\subseteq R(B)\}\;\text{or}\;\{r(A)=m,\;r(B)=p,\;\text{and}\;R(A^{⁎}M)=R(B)\}$*.*
(q) *The following 5 statements are equivalent:*
  〈1〉 $\left\{M^{\left(1,3,4\right)}\}⊇\{B^{\left(1,2,3\right)}A^{\left(1,2,3\right)}\}.\;\;\;\;\;\;\langle 2\rangle \;\{M^{\left(1,3,4\right)}\}⊇\{B^{\left(1,2,3\right)}A^{\left(1,3\right)}\right\}$*.*
  〈3〉 $\left\{M^{\left(1,3,4\right)}\}⊇\{B^{\left(1,3\right)}A^{\left(1,2,3\right)}\}.\;\;\;\;\;\;\;\;\langle 4\rangle \;\{M^{\left(1,3,4\right)}\}⊇\{B^{\left(1,3\right)}A^{\left(1,3\right)}\right\}$*.*
  〈5〉 $M=0\;\text{or}\;\{r(B)=p\;\text{and}\;R(A^{⁎}M)=R(B)\}$*.*
(r) *The following 5 statements are equivalent:*
  〈1〉 $\left\{M^{\left(1,3,4\right)}\}⊇\{B^{\left(1,2,4\right)}A^{\left(1,2,3\right)}\}.\;\;\;\;\;\langle 2\rangle \;\{M^{\left(1,3,4\right)}\}⊇\{B^{\left(1,2,4\right)}A^{\left(1,3\right)}\right\}$*.*
  〈3〉 $\left\{M^{\left(1,3,4\right)}\}⊇\{B^{\left(1,4\right)}A^{\left(1,2,3\right)}\}.\;\;\;\;\;\;\;\langle 4\rangle \;\{M^{\left(1,3,4\right)}\}⊇\{B^{\left(1,4\right)}A^{\left(1,3\right)}\right\}$*.*
  〈5〉 M=0orr(M)=n*.*
(s) *The following 5 statements are equivalent:*
  〈1〉 $\left\{M^{\left(1,3,4\right)}\}⊇\{B^{\left(1,2,4\right)}A^{\left(1,2\right)}\}.\;\;\;\;\;\;\langle 2\rangle \;\{M^{\left(1,3,4\right)}\}⊇\{B^{\left(1,2,4\right)}A^{\left(1\right)}\right\}$*.*
  〈3〉 $\left\{M^{\left(1,3,4\right)}\}⊇\{B^{\left(1,4\right)}A^{\left(1,2\right)}\}.\;\;\;\;\;\;\;\;\langle 4\rangle \;\{M^{\left(1,3,4\right)}\}⊇\{B^{\left(1,4\right)}A^{\left(1\right)}\right\}$*.*
  〈5〉 M=0orr(M)=m=n*.*
(t) *The following 5 statements are equivalent:*
  〈1〉 $\left\{M^{\left(1,3,4\right)}\}⊇\{B^{\left(1,2\right)}A^{\left(1,2,3\right)}\}.\;\;\;\;\;\;\langle 2\rangle \;\{M^{\left(1,3,4\right)}\}⊇\{B^{\left(1,2\right)}A^{\left(1,3\right)}\right\}$*.*
  〈3〉 $\left\{M^{\left(1,3,4\right)}\}⊇\{B^{\left(1\right)}A^{\left(1,2,3\right)}\}.\;\;\;\;\;\;\;\;\langle 4\rangle \;\{M^{\left(1,3,4\right)}\}⊇\{B^{\left(1\right)}A^{\left(1,3\right)}\right\}$*.*
  〈5〉 M=0orr(M)=n=p*.*
(u) *The following 5 statements are equivalent:*
  〈1〉 $\left\{M^{\left(1,3,4\right)}\}⊇\{B^{\left(1,2\right)}A^{\left(1,2\right)}\}.\;\;\;\;\;\;\;\langle 2\rangle \;\{M^{\left(1,3,4\right)}\}⊇\{B^{\left(1,2\right)}A^{\left(1\right)}\right\}$*.*
  〈3〉 $\left\{M^{\left(1,3,4\right)}\}⊇\{B^{\left(1\right)}A^{\left(1,2\right)}\}.\;\;\;\;\;\;\;\;\;\langle 4\rangle \;\{M^{\left(1,3,4\right)}\}⊇\{B^{\left(1\right)}A^{\left(1\right)}\right\}$*.*
  〈5〉 M=0orr(M)=m=n=p*.*

Since the product $B^{†}A^{†}$ is unique, the last 64 cases in (1.10) can be written as

(10.1) $${\left(AB\right)}^{†}=\{{\left(AB\right)}^{\left(1,2,3,4\right)}\}⊇\{B^{\left(s_{2},\ldots ,t_{2}\right)}A^{\left(s_{1},\ldots ,t_{1}\right)}\}$$

for the eight commonly-used types of generalized inverses of matrices. From (4.100),

(10.2) $${\left(AB\right)}^{†}=\{{\left(AB\right)}^{\left(1,2,3,4\right)}\}⊇\{B^{\left(s_{2},\ldots ,t_{2}\right)}A^{\left(s_{1},\ldots ,t_{1}\right)}\}\Leftrightarrow \;B^{\left(s_{2},\ldots ,t_{2}\right)}A^{\left(s_{1},\ldots ,t_{1}\right)}\;\text{is invariant and}\;{\left(AB\right)}^{†}=B^{†}A^{†}.$$

Consequently combining Theorem 6.1 and a group of necessary and sufficient conditions for ${\left(AB\right)}^{†}=B^{†}A^{†}$ to hold in [34], we obtain the following results.

Theorem 10.4 *Let* $A\in C^{m\times n}$ *and* $B\in C^{n\times p}$ *be given. Then the following results hold.*

〈1〉 [34] ${\left(AB\right)}^{†}=B^{†}A^{†}$ ⇔ $R(A^{⁎}AB)\subseteq R(B)$ *and* $R(BB^{⁎}A^{⁎})\subseteq R(A^{⁎})$*.*
〈2〉 ${\left(AB\right)}^{†}=B^{†}A^{\left(1,3,4\right)}$ *holds for all* $A^{\left(1,3,4\right)}$ ⇔ {r(A)=m*,* $R(A^{⁎}AB)\subseteq R(B)$*, and* $R(BB^{⁎}A^{⁎})\subseteq R(A^{⁎})\}$ *or* $R(A^{⁎}AB)=R(B)$*.*
〈3〉 ${\left(AB\right)}^{†}=B^{†}A^{\left(1,2,4\right)}$ *holds for all* $A^{\left(1,2,4\right)}$ ⇔ AB=0 *or* {r(A)=m*,* $R(A^{⁎}AB)\subseteq R(B)$*, and* $R(BB^{⁎}A^{⁎})\subseteq R(A^{⁎})\}$*.*
〈4〉 ${\left(AB\right)}^{†}=B^{†}A^{\left(1,2,3\right)}$ *holds for all* $A^{\left(1,2,3\right)}$ ⇔ A=0 *or* $R(A^{⁎}AB)=R(B)$*.*
〈5〉 ${\left(AB\right)}^{†}=B^{†}A^{\left(1,4\right)}$ *holds for all* $A^{\left(1,4\right)}$ ⇔ {r(A)=m*,* $R(A^{⁎}AB)\subseteq R(B)$*, and* $R(BB^{⁎}A^{⁎})\subseteq R(A^{⁎})\}$ *or* B=0*.*
〈6〉 ${\left(AB\right)}^{†}=B^{†}A^{\left(1,3\right)}$ *holds for all* $A^{\left(1,3\right)}$ ⇔ $R(A^{⁎}AB)=R(B)$*.*
〈7〉 ${\left(AB\right)}^{†}=B^{†}A^{\left(1,2\right)}$ *holds for all* $A^{\left(1,2\right)}$ ⇔ A=0 *or* B=0 *or* {r(A)=m *and* $R(A^{⁎}AB)=R(B)\}$*.*
〈8〉 ${\left(AB\right)}^{†}=B^{†}A^{\left(1\right)}$ *holds for all* $A^{\left(1\right)}$ ⇔ {r(A)=m *and* $R(A^{⁎}AB)=R(B)\}$ *or* B=0*.*
〈9〉 ${\left(AB\right)}^{†}=B^{\left(1,3,4\right)}A^{†}$ *holds for all* $B^{\left(1,3,4\right)}$ ⇔ {r(B)=p*,* $R(A^{⁎}AB)\subseteq R(B)$*, and* $R(BB^{⁎}A^{⁎})\subseteq R(A^{⁎})\}$ *or* $R(BB^{⁎}A^{⁎})=R(A^{⁎})$*.*
〈10〉 ${\left(AB\right)}^{†}=B^{\left(1,3,4\right)}A^{\left(1,3,4\right)}$ *holds for all* $B^{\left(1,3,4\right)}$ *and* $A^{\left(1,3,4\right)}$ ⇔ r(A)=r(B)=n *or* {r(A)=m*,* r(B)=p*,* $R(A^{⁎}AB)\subseteq R(B)$*, and* $R(BB^{⁎}A^{⁎})\subseteq R(A^{⁎})\}$ *or* {r(A)=m *and* $R(BB^{⁎}A^{⁎})=R(A^{⁎})\}$ *or* {r(B)=p *and* $R(A^{⁎}AB)=R(B)\}$*.*
〈11〉 ${\left(AB\right)}^{†}=B^{\left(1,3,4\right)}A^{\left(1,2,4\right)}$ *holds for all* $B^{\left(1,3,4\right)}$ *and* $A^{\left(1,2,4\right)}$ ⇔ A=0 *or* {AB=0 *and* r(B)=p} *or* {r(A)=m *and* r(B)=p*,* $R(A^{⁎}AB)\subseteq R(B)$*, and* $R(BB^{⁎}A^{⁎})\subseteq R(A^{⁎})\}$ *or* {r(A)=m *and* $R(BB^{⁎}A^{⁎})=R(A^{⁎})\}$*.*
〈12〉 ${\left(AB\right)}^{†}=B^{\left(1,3,4\right)}A^{\left(1,2,3\right)}$ *holds for all* $B^{\left(1,3,4\right)}$ *and* $A^{\left(1,2,3\right)}$ ⇔ A=0 *or* r(A)=r(B)=n *or* {r(B)=p *and* $R(A^{⁎}AB)=R(B)\}$*.*
〈13〉 ${\left(AB\right)}^{†}=B^{\left(1,3,4\right)}A^{\left(1,4\right)}$ *holds for all* $B^{\left(1,3,4\right)}$ *and* $A^{\left(1,4\right)}$ ⇔ {r(A)=m*,* r(B)=p*,* $R(A^{⁎}AB)\subseteq R(B)$*, and* $R(BB^{⁎}A^{⁎})\subseteq R(A^{⁎})\}$ *or* {r(A)=m *and* $R(BB^{⁎}A^{⁎})=R(A^{⁎})\}$*.*
〈14〉 ${\left(AB\right)}^{†}=B^{\left(1,3,4\right)}A^{\left(1,3\right)}$ *holds for all* $B^{\left(1,3,4\right)}$ *and* $A^{\left(1,3\right)}$ ⇔ r(A)=r(B)=n *or* {r(B)=p *and* $R(A^{⁎}AB)=R(B)\}$*.*
〈15〉 ${\left(AB\right)}^{†}=B^{\left(1,3,4\right)}A^{\left(1,2\right)}$ *holds for all* $B^{\left(1,3,4\right)}$ *and* $A^{\left(1,2\right)}$ ⇔ A=0 *or* r(A)=r(B)=m=n *or* {AB=0,r(A)=m,andr(B)=p}*.*
〈16〉 ${\left(AB\right)}^{†}=B^{\left(1,3,4\right)}A^{\left(1\right)}$ *holds for all* $B^{\left(1,3,4\right)}$ *and* $A^{\left(1\right)}$ ⇔ r(A)=r(B)=m=n *or* {r(A)=m*,* r(B)=p*, and* $R(A^{⁎}AB)=R(B)\}$*.*
〈17〉 ${\left(AB\right)}^{†}=B^{\left(1,2,4\right)}A^{†}$ *holds for all* $B^{\left(1,2,4\right)}$ ⇔ B=0 *or* $R(BB^{⁎}A^{⁎})=R(A^{⁎})$*.*
〈18〉 ${\left(AB\right)}^{†}=B^{\left(1,2,4\right)}A^{\left(1,3,4\right)}$ *holds for all* $B^{\left(1,2,4\right)}$ *and* $A^{\left(1,3,4\right)}$ ⇔ B=0 *or* r(A)=r(B)=n *or* {r(A)=m *and* $R(BB^{⁎}A^{⁎})=R(A^{⁎})\}$*.*
〈19〉 ${\left(AB\right)}^{†}=B^{\left(1,2,4\right)}A^{\left(1,2,4\right)}$ *holds for all* $B^{\left(1,2,4\right)}$ *and* $A^{\left(1,2,4\right)}$ ⇔ A=0 *or* B=0 *or* {r(A)=m *and* $R(BB^{⁎}A^{⁎})=R(A^{⁎})\}$*.*
〈20〉 ${\left(AB\right)}^{†}=B^{\left(1,2,4\right)}A^{\left(1,2,3\right)}$ *holds for all* $B^{\left(1,2,4\right)}$ *and* $A^{\left(1,2,3\right)}$ ⇔ A=0 *or* B=0 *or* r(A)=r(B)=n*.*
〈21〉 ${\left(AB\right)}^{†}=B^{\left(1,2,4\right)}A^{\left(1,4\right)}$ *holds for all* $B^{\left(1,2,4\right)}$ *and* $A^{\left(1,4\right)}$ ⇔ B=0 *or* {r(A)=m *and* $R(BB^{⁎}A^{⁎})=R(A^{⁎})\}$*.*
〈22〉 ${\left(AB\right)}^{†}=B^{\left(1,2,4\right)}A^{\left(1,3\right)}$ *holds for all* $B^{\left(1,2,4\right)}$ *and* $A^{\left(1,3\right)}$ ⇔ B=0 *or* r(A)=r(B)=n*.*
〈23〉 ${\left(AB\right)}^{†}=B^{\left(1,2,4\right)}A^{\left(1,2\right)}$ *holds for all* $B^{\left(1,2,4\right)}$ *and* $A^{\left(1,2\right)}$ ⇔ A=0 *or* B=0 *or* r(A)=r(B)=m=n*.*
〈24〉 ${\left(AB\right)}^{†}=B^{\left(1,2,4\right)}A^{\left(1\right)}$ *holds for all* $B^{\left(1,2,4\right)}$ *and* $A^{\left(1\right)}$ ⇔ B=0 *or* r(A)=r(B)=m=n*.*
〈25〉 ${\left(AB\right)}^{†}=B^{\left(1,2,3\right)}A^{†}$ *holds for all* $B^{\left(1,2,3\right)}$ ⇔ A=0 *or* B=0 *or* {(B)=p*,* $R(A^{⁎}AB)\subseteq R(B)$*, and* $R(BB^{⁎}A^{⁎})\subseteq R(A^{⁎})\}$*.*
〈26〉 ${\left(AB\right)}^{†}=B^{\left(1,2,3\right)}A^{\left(1,3,4\right)}$ *holds for all* $B^{\left(1,2,3\right)}$ *and* $A^{\left(1,3,4\right)}$ ⇔ B=0 *or* {AB=0 *and* r(A)=m*,* $R(A^{⁎}AB)\subseteq R(B)$*, and* $R(BB^{⁎}A^{⁎})\subseteq R(A^{⁎})\}$ *or* {r(A)=m *and* r(B)=p*,* $R(A^{⁎}AB)\subseteq R(B)$*, and* $R(BB^{⁎}A^{⁎})\subseteq R(A^{⁎})\}$ *or* {r(B)=p *and* $R(A^{⁎}AB)=R(B)\}$*.*
〈27〉 ${\left(AB\right)}^{†}=B^{\left(1,2,3\right)}A^{\left(1,2,4\right)}$ *holds for all* $B^{\left(1,2,3\right)}$ *and* $A^{\left(1,2,4\right)}$ ⇔ AB=0 *or* {r(A)=m*,* r(B)=p*,* $R(A^{⁎}AB)\subseteq R(B)$*, and* $R(BB^{⁎}A^{⁎})\subseteq R(A^{⁎})\}$*.*
〈28〉 ${\left(AB\right)}^{†}=B^{\left(1,2,3\right)}A^{\left(1,2,3\right)}$ *holds for all* $B^{\left(1,2,3\right)}$ *and* $A^{\left(1,2,3\right)}$ ⇔ A=0 *or* B=0 *or* {r(B)=p *and* $R(A^{⁎}AB)=R(B)\}$*.*
〈29〉 ${\left(AB\right)}^{†}=B^{\left(1,2,3\right)}A^{\left(1,4\right)}$ *holds for all* $B^{\left(1,2,3\right)}$ *and* $A^{\left(1,4\right)}$ ⇔ B=0 *or* {AB=0 *and* r(A)=m} *or* {r(A)=m*,* r(B)=p*,* $R(A^{⁎}AB)\subseteq R(B)$*, and* $R(BB^{⁎}A^{⁎})\subseteq R(A^{⁎})\}$*.*
〈30〉 ${\left(AB\right)}^{†}=B^{\left(1,2,3\right)}A^{\left(1,3\right)}$ *holds for all* $B^{\left(1,2,3\right)}$ *and* $A^{\left(1,3\right)}$ ⇔ B=0 *or* r(B)=p *and* $R(A^{⁎}AB)=R(B)$*.*
〈31〉 ${\left(AB\right)}^{†}=B^{\left(1,2,3\right)}A^{\left(1,2\right)}$ *holds for all* $B^{\left(1,2,3\right)}$ *and* $A^{\left(1,2\right)}$ ⇔ A=0 *or* B=0 *or* {r(A)=m*,* r(B)=p*, and* $R(A^{⁎}AB)=R(B)\}$*.*
〈32〉 ${\left(AB\right)}^{†}=B^{\left(1,2,3\right)}A^{\left(1\right)}$ *holds for all* $B^{\left(1,2,3\right)}$ *and* $A^{\left(1\right)}$ ⇔ B=0 *or* {r(A)=m*,* r(B)=p*, and* $R(A^{⁎}AB)=R(B)\}$*.*
〈33〉 ${\left(AB\right)}^{†}=B^{\left(1,4\right)}A^{†}$ *holds for all* $B^{\left(1,4\right)}$ ⇔ $R(BB^{⁎}A^{⁎})=R(A^{⁎})$*.*
〈34〉 ${\left(AB\right)}^{†}=B^{\left(1,4\right)}A^{\left(1,3,4\right)}$ *holds for all* $B^{\left(1,4\right)}$ *and* $A^{\left(1,3,4\right)}$ ⇔ r(A)=r(B)=n *or* {r(A)=m *and* $R(BB^{⁎}A^{⁎})=R(A^{⁎})\}$*.*
〈35〉 ${\left(AB\right)}^{†}=B^{\left(1,4\right)}A^{\left(1,2,4\right)}$ *holds for all* $B^{\left(1,4\right)}$ *and* $A^{\left(1,2,4\right)}$ ⇔ A=0 {r(A)=m *and* $R(BB^{⁎}A^{⁎})=R(A^{⁎})\}$*.*
〈36〉 ${\left(AB\right)}^{†}=B^{\left(1,4\right)}A^{\left(1,2,3\right)}$ *holds for all* $B^{\left(1,4\right)}$ *and* $A^{\left(1,2,3\right)}$ ⇔ A=0 *or* r(A)=r(B)=n*.*
〈37〉 ${\left(AB\right)}^{†}=B^{\left(1,4\right)}A^{\left(1,4\right)}$ *holds for all* $B^{\left(1,4\right)}$ *and* $A^{\left(1,4\right)}$ ⇔ r(A)=m *and* $R(BB^{⁎}A^{⁎})=R(A^{⁎})$*.*
〈38〉 ${\left(AB\right)}^{†}=B^{\left(1,4\right)}A^{\left(1,3\right)}$ *holds for all* $B^{\left(1,4\right)}$ *and* $A^{\left(1,3\right)}$ ⇔ r(A)=r(B)=n*.*
〈39〉 ${\left(AB\right)}^{†}=B^{\left(1,4\right)}A^{\left(1,2\right)}$ *holds for all* $B^{\left(1,4\right)}$ *and* $A^{\left(1,2\right)}$ ⇔ A=0 *or* r(A)=r(B)=m=n*.*
〈40〉 ${\left(AB\right)}^{†}=B^{\left(1,4\right)}A^{\left(1\right)}$ *holds for all* $B^{\left(1,4\right)}$ *and* $A^{\left(1\right)}$ ⇔ r(A)=r(B)=m=n*.*
〈41〉 ${\left(AB\right)}^{†}=B^{\left(1,3\right)}A^{†}$ *holds for all* $B^{\left(1,3\right)}$ ⇔ r(B)=p*,* $R(A^{⁎}AB)\subseteq R(B)$*, and* $R(BB^{⁎}A^{⁎})\subseteq R(A^{⁎})$*.*
〈42〉 ${\left(AB\right)}^{†}=B^{\left(1,3\right)}A^{\left(1,3,4\right)}$ *holds for all* $B^{\left(1,3\right)}$ *and* $A^{\left(1,3,4\right)}$ ⇔ {r(A)=m*,* r(B)=p*,* $R(A^{⁎}AB)\subseteq R(B)$*, and* $R(BB^{⁎}A^{⁎})\subseteq R(A^{⁎})\}$ *or* {r(B)=p *and* $R(A^{⁎}AB)=R(B)\}$*.*
〈43〉 ${\left(AB\right)}^{†}=B^{\left(1,3\right)}A^{\left(1,2,4\right)}$ *holds for all* $B^{\left(1,3\right)}$ *and* $A^{\left(1,2,4\right)}$ ⇔ A=0 *or* {AB=0 *and* r(B)=p} *or* {r(A)=m*,* r(B)=p*,* $R(A^{⁎}AB)\subseteq R(B)$*, and* $R(BB^{⁎}A^{⁎})\subseteq R(A^{⁎})\}$*.*
〈44〉 ${\left(AB\right)}^{†}=B^{\left(1,3\right)}A^{\left(1,2,3\right)}$ *holds for all* $B^{\left(1,3\right)}$ *and* $A^{\left(1,2,3\right)}$ ⇔ A=0 *or* {r(B)=p *and* $R(A^{⁎}AB)=R(B)\}$*.*
〈45〉 ${\left(AB\right)}^{†}=B^{\left(1,3\right)}A^{\left(1,4\right)}$ *holds for all* $B^{\left(1,3\right)}$ *and* $A^{\left(1,4\right)}$ ⇔ r(A)=m*,* r(B)=p*,* $R(A^{⁎}AB)\subseteq R(B)$*, and* $R(BB^{⁎}A^{⁎})\subseteq R(A^{⁎})$*.*
〈46〉 ${\left(AB\right)}^{†}=B^{\left(1,3\right)}A^{\left(1,3\right)}$ *holds for all* $B^{\left(1,3\right)}$ *and* $A^{\left(1,3\right)}$ ⇔ r(B)=p *and* $R(A^{⁎}AB)=R(B)$*.*
〈47〉 ${\left(AB\right)}^{†}=B^{\left(1,3\right)}A^{\left(1,2\right)}$ *holds for all* $B^{\left(1,3\right)}$ *and* $A^{\left(1,2\right)}$ ⇔ A=0 *or* {r(A)=m*,* r(B)=p*, and* $R(A^{⁎}AB)=R(B)\}$*.*
〈48〉 ${\left(AB\right)}^{†}=B^{\left(1,3\right)}A^{\left(1\right)}$ *holds for all* $B^{\left(1,3\right)}$ *and* $A^{\left(1\right)}$ ⇔ r(A)=m*,* r(B)=p*, and* $R(A^{⁎}AB)=R(B)$*.*
〈49〉 ${\left(AB\right)}^{†}=B^{\left(1,2\right)}A^{†}$ *holds for all* $B^{\left(1,2\right)}$ ⇔ A=0 *or* B=0 *or* {r(B)=p *and* $R(BB^{⁎}A^{⁎})=R(A^{⁎})\}$*.*
〈50〉 ${\left(AB\right)}^{†}=B^{\left(1,2\right)}A^{\left(1,3,4\right)}$ *holds for all* $B^{\left(1,2\right)}$ *and* $A^{\left(1,3,4\right)}$ ⇔ B=0 *or* r(A)=r(B)=n=p *or* {AB=0,r(A)=m,andr(B)=p}*.*
〈51〉 ${\left(AB\right)}^{†}=B^{\left(1,2\right)}A^{\left(1,2,4\right)}$ *holds for all* $B^{\left(1,2\right)}$ *and* $A^{\left(1,2,4\right)}$ ⇔ A=0 *or* B=0 *or* {r(A)=m*,* r(B)=p*, and* $R(BB^{⁎}A^{⁎})=R(A^{⁎})\}$*.*
〈52〉 ${\left(AB\right)}^{†}=B^{\left(1,2\right)}A^{\left(1,2,3\right)}$ *holds for all* $B^{\left(1,2\right)}$ *and* $A^{\left(1,2,3\right)}$ ⇔ A=0 *or* B=0 *or* r(A)=r(B)=n=p*.*
〈53〉 ${\left(AB\right)}^{†}=B^{\left(1,2\right)}A^{\left(1,4\right)}$ *holds for all* $B^{\left(1,2\right)}$ *and* $A^{\left(1,4\right)}$ ⇔ B=0 *or* {r(A)=m*,* r(B)=p*, and* $R(BB^{⁎}A^{⁎})=R(A^{⁎})\}$*.*
〈54〉 ${\left(AB\right)}^{†}=B^{\left(1,2\right)}A^{\left(1,3\right)}$ *holds for all* $B^{\left(1,2\right)}$ *and* $A^{\left(1,3\right)}$ ⇔ B=0 *or* r(A)=r(B)=n=p*.*
〈55〉 ${\left(AB\right)}^{†}=B^{\left(1,2\right)}A^{\left(1,2\right)}$ *holds for all* $B^{\left(1,2\right)}$ *and* $A^{\left(1,2\right)}$ ⇔ A=0 *or* B=0 *or* r(A)=r(B)=m=n=p*.*
〈56〉 ${\left(AB\right)}^{†}=B^{\left(1,2\right)}A^{\left(1\right)}$ *holds for all* $B^{\left(1,2\right)}$ *and* $A^{\left(1\right)}$ ⇔ B=0 *or* r(A)=r(B)=m=n=p*.*
〈57〉 ${\left(AB\right)}^{†}=B^{\left(1\right)}A^{†}$ *holds for all* $B^{\left(1\right)}$ ⇔ r(B)=p *and* $R(BB^{⁎}A^{⁎})=R(A^{⁎})$*.*
〈58〉 ${\left(AB\right)}^{†}=B^{\left(1\right)}A^{\left(1,3,4\right)}$ *holds for all* $B^{\left(1\right)}$ *and* $A^{\left(1,3,4\right)}$ ⇔ r(A)=r(B)=n=p *or* {r(A)=m*,* r(B)=p*, and* $R(BB^{⁎}A^{⁎})=R(A^{⁎})\}$*.*
〈59〉 ${\left(AB\right)}^{†}=B^{\left(1\right)}A^{\left(1,2,4\right)}$ *holds for all* $B^{\left(1\right)}$ *and* $A^{\left(1,2,4\right)}$ ⇔ A=0 *or* {r(A)=m*,* r(B)=p*, and* $R(BB^{⁎}A^{⁎})=R(A^{⁎})\}$*.*
〈60〉 ${\left(AB\right)}^{†}=B^{\left(1\right)}A^{\left(1,2,3\right)}$ *holds for all* $B^{\left(1\right)}$ *and* $A^{\left(1,2,3\right)}$ ⇔ r(A)=r(B)=n=p*.*
〈61〉 ${\left(AB\right)}^{†}=B^{\left(1\right)}A^{\left(1,4\right)}$ *holds for all* $B^{\left(1\right)}$ *and* $A^{\left(1,4\right)}$ ⇔ r(A)=m*,* r(B)=p*, and* $R(BB^{⁎}A^{⁎})=R(A^{⁎})$*.*
〈62〉 ${\left(AB\right)}^{†}=B^{\left(1\right)}A^{\left(1,3\right)}$ *holds for all* $B^{\left(1\right)}$ *and* $A^{\left(1,3\right)}$ ⇔ r(A)=r(B)=n=p*.*
〈63〉 ${\left(AB\right)}^{†}=B^{\left(1\right)}A^{\left(1,2\right)}$ *holds for all* $B^{\left(1\right)}$ *and* $A^{\left(1,2\right)}$ ⇔ A=0 *or* r(A)=r(B)=m=n=p*.*
〈64〉 ${\left(AB\right)}^{†}=B^{\left(1\right)}A^{\left(1\right)}$ *holds for all* $B^{\left(1\right)}$ *and* $A^{\left(1\right)}$ ⇔ r(A)=r(B)=m=n=p*.*

## 11 The reverse order law ${\left(AB\right)}^{†}=B^{†}A^{†}$ and its equivalent equalities and facts

This section is completely devoted to the following famous ROL:

(11.1) $${\left(AB\right)}^{†}=B^{†}A^{†}$$

in the theory of generalized inverses. Since (11.1) is a relatively straightforward generalization of the fundamental matrix identity ${\left(AB\right)}^{-1}=B^{-1}A^{-1}$, it is well endowed with certain nice properties that enable its perspective study to become well developed since the initial work in [34]. There have been plenty of classic and novel discussions and results in the literature concerning the derivations and representations of the ROL and its equivalent assertions due to its vital role in the theory of ROL problems. Eq. (11.1) motivates from time to time some profound considerations and explorations of universal-algebraic methods for dealing with this ROL and other matrix equality problems concerning Moore–Penrose inverses with developments appearing in a diversity of journals.

In this section, we present a series of known and new formulas, results, and facts associated with (11.1), and give detailed derivations of the main part of the issue. In the beginning part, we present a collection of obvious or known facts concerning (11.1) and its equivalent expressions and assertions.

Theorem 11.1 *Let* $A\in C^{m\times n}$ *and* $B\in C^{n\times p}$*. Then the following 40 statements are equivalent:*

〈1〉 ${\left(AB\right)}^{†}=B^{†}A^{†}$*.*
〈2〉 $B{\left(AB\right)}^{†}A=BB^{†}A^{†}A$*.*
〈3〉 $AA^{⁎}{\left(B^{⁎}A^{⁎}\right)}^{†}B^{⁎}B=AB$*.*
〈4〉 ${\left(AB\right)}^{†}=B^{†}A^{†}ABB^{†}A^{†}$*.*
〈5〉 ${\left(AB\right)}^{†}={\left(A^{†}AB\right)}^{†}A^{†}$ *and* ${\left(A^{†}AB\right)}^{†}=B^{†}A^{†}A$*.*
〈6〉 ${\left(AB\right)}^{†}=B^{†}{\left(ABB^{†}\right)}^{†}$ *and* ${\left(ABB^{†}\right)}^{†}=BB^{†}A^{†}$*.*
〈7〉 ${\left(AB\right)}^{†}=B^{†}{\left(A^{†}ABB^{†}\right)}^{†}A^{†}$ *and* ${\left(A^{†}ABB^{†}\right)}^{†}=BB^{†}A^{†}A$*.*
〈8〉 $\{{\left(AB\right)}^{\left(1,3\right)}\}∋B^{†}A^{†}$ *and* $\{{\left(AB\right)}^{\left(1,4\right)}\}∋B^{†}A^{†}$*.*
〈9〉 ${\left(AB\right)}^{†}\in \{B^{\left(1,3\right)}A^{\left(1,4\right)}\}$*.*
〈10〉 $AB{\left(AB\right)}^{†}=ABB^{†}A^{†}={\left(A^{†}\right)}^{⁎}BB^{†}A^{⁎}$ *and* ${\left(AB\right)}^{†}AB=B^{†}A^{†}AB=B^{⁎}A^{†}A{\left(B^{†}\right)}^{⁎}$*.*
〈11〉 ${\left({\left(A^{†}\right)}^{⁎}B\right)}^{†}=B^{†}A^{⁎}$*.*
〈12〉 $A^{†}{\left(B^{⁎}A^{†}\right)}^{†}B^{⁎}=A^{†}ABB^{†}$*.*
〈13〉 $AA^{†}{\left(B^{⁎}A^{†}\right)}^{†}B^{⁎}B=AB$*.*
〈14〉 ${\left({\left(A^{†}\right)}^{⁎}B\right)}^{†}=B^{†}A^{†}ABB^{†}A^{⁎}$*.*
〈15〉 ${\left({\left(A^{†}\right)}^{⁎}B\right)}^{†}={\left(A^{†}AB\right)}^{†}A^{⁎}$ *and* ${\left(A^{†}AB\right)}^{†}=B^{†}A^{†}A$*.*
〈16〉 ${\left({\left(A^{†}\right)}^{⁎}B\right)}^{†}=B^{†}{\left({\left(A^{†}\right)}^{⁎}BB^{†}\right)}^{†}$ *and* ${\left({\left(A^{†}\right)}^{⁎}BB^{†}\right)}^{†}=BB^{†}A^{⁎}$*.*
〈17〉 ${\left({\left(A^{†}\right)}^{⁎}B\right)}^{†}=B^{†}{\left(A^{†}ABB^{†}\right)}^{†}A^{⁎}\;\text{and}\;{\left(A^{†}ABB^{†}\right)}^{†}=BB^{†}A^{†}A$*.*
〈18〉 $\left\{{\left({\left(A^{†}\right)}^{⁎}B\right)}^{\left(1,3\right)}\right\}∋B^{†}A^{⁎}$ *and* $\left\{{\left({\left(A^{†}\right)}^{⁎}B\right)}^{\left(1,4\right)}\right\}∋B^{†}A^{⁎}$*.*
〈19〉 ${\left(B^{⁎}A^{†}\right)}^{†}B^{⁎}A^{†}=ABB^{†}A^{†}={\left(A^{†}\right)}^{⁎}BB^{†}A^{⁎}$ *and* $B^{⁎}A^{†}{\left(B^{⁎}A^{†}\right)}^{†}=B^{†}A^{†}AB=B^{⁎}A^{†}A{\left(B^{†}\right)}^{⁎}$*.*
〈20〉 ${\left(A{\left(B^{†}\right)}^{⁎}\right)}^{†}=B^{⁎}A^{†}$*.*
〈21〉 ${\left(B^{†}\right)}^{⁎}{\left(A{\left(B^{†}\right)}^{⁎}\right)}^{†}A=BB^{†}A^{†}A$*.*
〈22〉 $AA^{⁎}{\left(B^{†}A^{⁎}\right)}^{†}B^{†}B=AB$*.*
〈23〉 ${\left(A{\left(B^{†}\right)}^{⁎}\right)}^{†}=B^{⁎}A^{†}ABB^{†}A^{†}$*.*
〈24〉 ${\left(A{\left(B^{†}\right)}^{⁎}\right)}^{†}={\left(A^{†}A{\left(B^{†}\right)}^{⁎}\right)}^{†}A^{†}$ *and* ${\left(A^{†}A{\left(B^{†}\right)}^{⁎}\right)}^{†}=B^{⁎}A^{†}A$*.*
〈25〉 ${\left(A{\left(B^{†}\right)}^{⁎}\right)}^{†}=B^{⁎}{\left(ABB^{†}\right)}^{†}$ *and* ${\left(ABB^{†}\right)}^{†}=BB^{†}A^{†}$*.*
〈26〉 ${\left(A{\left(B^{†}\right)}^{⁎}\right)}^{†}=B^{⁎}{\left(A^{†}ABB^{†}\right)}^{†}A^{†}\;\text{and}\;{\left(A^{†}ABB^{†}\right)}^{†}=BB^{†}A^{†}A$*.*
〈27〉 $\left\{{\left(A{\left(B^{†}\right)}^{⁎}\right)}^{\left(1,3\right)}\right\}∋B^{⁎}A^{†}$ *and* $\left\{{\left(A{\left(B^{†}\right)}^{⁎}\right)}^{\left(1,4\right)}\right\}∋B^{⁎}A^{†}$*.*
〈28〉 ${\left(B^{†}A^{⁎}\right)}^{†}B^{†}A^{⁎}=ABB^{†}A^{†}={\left(A^{†}\right)}^{⁎}BB^{†}A^{⁎}$ *and* $B^{†}A^{⁎}{\left(B^{†}A^{⁎}\right)}^{†}=B^{†}A^{†}AB=B^{⁎}A^{†}A{\left(B^{†}\right)}^{⁎}$*.*
〈29〉 ${\left(B^{†}A^{†}\right)}^{†}=AB$*.*
〈30〉 $A^{†}{\left(B^{†}A^{†}\right)}^{†}B^{†}=A^{†}ABB^{†}$*.*
〈31〉 ${\left(AA^{⁎}\right)}^{†}{\left(B^{†}A^{†}\right)}^{†}{\left(B^{⁎}B\right)}^{†}={\left(A^{†}\right)}^{⁎}{\left(B^{†}\right)}^{⁎}$*.*
〈32〉 ${\left(B^{†}A^{†}\right)}^{†}=ABB^{†}A^{†}AB$*.*
〈33〉 ${\left(B^{†}A^{†}\right)}^{†}=A{\left(B^{†}A^{†}A\right)}^{†}\;\text{and}\;{\left(B^{†}A^{†}A\right)}^{†}=A^{†}AB$*.*
〈34〉 ${\left(B^{†}A^{†}\right)}^{†}={\left(BB^{†}A^{†}\right)}^{†}B\;\text{and}\;{\left(BB^{†}A^{†}\right)}^{†}=ABB^{†}$*.*
〈35〉 ${\left(B^{†}A^{†}\right)}^{†}=A{\left(BB^{†}A^{†}A\right)}^{†}B\;\text{and}\;{\left(BB^{†}A^{†}A\right)}^{†}=A^{†}ABB^{†}$*.*
〈36〉 $\left\{{\left(B^{†}A^{†}\right)}^{\left(1,3\right)}\right\}∋AB$ *and* $\left\{{\left(B^{†}A^{†}\right)}^{\left(1,4\right)}\right\}∋AB$*.*
〈37〉 $B^{†}A^{†}{\left(B^{†}A^{†}\right)}^{†}=B^{†}A^{†}AB=B^{⁎}A^{†}A{\left(B^{†}\right)}^{⁎}$ *and* ${\left(B^{†}A^{†}\right)}^{†}B^{†}A^{†}=ABB^{†}A^{†}={\left(A^{†}\right)}^{⁎}BB^{†}A^{⁎}$*.*
〈38〉 ${\left(AB\right)}^{†}=B^{⁎}{\left(BB^{⁎}\right)}^{k-1}{\left({\left(A^{⁎}A\right)}^{k}{\left(BB^{⁎}\right)}^{k}\right)}^{†}{\left(A^{⁎}A\right)}^{k-1}A^{⁎}$ *and* ${\left({\left(A^{⁎}A\right)}^{k}{\left(BB^{⁎}\right)}^{k}\right)}^{†}={\left({\left(BB^{⁎}\right)}^{k}\right)}^{†}{\left({\left(A^{⁎}A\right)}^{k}\right)}^{†}$ *for any integer* k≥1*.*
〈39〉 ${\left(AB\right)}^{†}={\left(BB^{⁎}\right)}^{k}{\left(A{\left(A^{⁎}A\right)}^{k}{\left(BB^{⁎}\right)}^{k}B\right)}^{†}{\left(AA^{⁎}\right)}^{k}$ *and* ${\left(A{\left(A^{⁎}A\right)}^{k}{\left(BB^{⁎}\right)}^{k}B\right)}^{†}={\left({\left(BB^{⁎}\right)}^{k}B\right)}^{†}{\left(A{\left(A^{⁎}A\right)}^{k}\right)}^{†}$ *for any integer* k≥1*.*
〈40〉 $r\left[\begin{array}{cc} AB{\left(AB\right)}^{⁎}AB & ABB^{⁎}B\\ AA^{⁎}AB & AB \end{array}\right]=r(AB)$*.*

Proof Pre- and post-multiplying (11.1) with *B* and *A* lead to 〈2〉. Pre- and post-multiplying (11.1) with $B^{⁎}B$ and $AA^{⁎}$ and taking conjugate transpose lead to 〈3〉. Conversely, pre- and post-multiplying 〈2〉 with $B^{†}$ and $A^{†}$, and pre- and post-multiplying 〈3〉 with ${\left(AA^{⁎}\right)}^{†}$ and ${\left(B^{⁎}B\right)}^{†}$ and taking conjugate transpose lead to 〈1〉.

Eq. (11.1) implies ${\left(AB\right)}^{†}={\left(AB\right)}^{†}AB{\left(AB\right)}^{†}=B^{†}A^{†}ABB^{†}A^{†}$, i.e., 〈1〉 implies 〈4〉. Conversely, pre-multiplying the equality in 〈4〉 with *AB* yields $AB{\left(AB\right)}^{†}=ABB^{†}A^{†}ABB^{†}A^{†}={\left(ABB^{†}A^{†}\right)}^{2}$, where $AB{\left(AB\right)}^{†}$ is idempotent. Hence ${\left(ABB^{†}A^{†}\right)}^{2}=ABB^{†}A^{†}$, which is equivalent to $B^{†}A^{†}ABB^{†}A^{†}=B^{†}A^{†}$ by Theorem 9.1〈88〉 and 〈93〉. Thus, 〈4〉 implies 〈1〉.

If 〈1〉 holds, then it is easy to verify the following four equalities

$$A^{†}AB(B^{†}A^{†}A)A^{†}AB=A^{†}ABB^{†}A^{†}AB=A^{†}AB{\left(AB\right)}^{†}AB=A^{†}AB,(B^{†}A^{†}A)A^{†}AB(B^{†}A^{†}A)=B^{†}A^{†}ABB^{†}A^{†}A={\left(AB\right)}^{†}AB{\left(AB\right)}^{†}A={\left(AB\right)}^{†}A=B^{†}A^{†}A,{\left(A^{†}AB(B^{†}A^{†}A)\right)}^{⁎}={\left(A^{†}ABB^{†}A^{†}A\right)}^{⁎}=A^{†}ABB^{†}A^{†}A=A^{†}AB(B^{†}A^{†}A),{\left((B^{†}A^{†}A)A^{†}AB\right)}^{⁎}={\left(B^{†}A^{†}AB\right)}^{⁎}={\left({\left(AB\right)}^{†}AB\right)}^{⁎}={\left(AB\right)}^{†}AB=(B^{†}A^{†}A)A^{†}AB$$

hold, which by definition mean that

(11.2) $${\left(A^{†}AB\right)}^{†}=B^{†}A^{†}A\;\;\mathrm{or equivalently}\;\;A^{†}AB={\left(B^{†}A^{†}A\right)}^{†}$$

holds. Subsequently, substituting it into (11.1) yields

(11.3) $${\left(AB\right)}^{†}=B^{†}A^{†}=(B^{†}A^{†}A)A^{†}={\left(A^{†}AB\right)}^{†}A^{†}.$$

Combining (11.2) and (11.3) leads to 〈5〉. Conversely, merging the two equalities in 〈5〉 and simplifying lead to

$${\left(AB\right)}^{†}={\left(A^{†}AB\right)}^{†}A^{†}=B^{†}A^{†}AA^{†}=B^{†}A^{†},$$

establishing 〈1〉. The equivalences of 〈1〉 and 〈6〉 can be shown by a similar approach.

If 〈1〉 holds, then it is easy to verify the following four equalities

$$A^{†}ABB^{†}(BB^{†}A^{†}A)A^{†}ABB^{†}=A^{†}ABB^{†}A^{†}ABB^{†}=A^{†}AB{\left(AB\right)}^{†}ABB^{†}=A^{†}ABB^{†},BB^{†}A^{†}AA^{†}ABB^{†}BB^{†}A^{†}A=BB^{†}A^{†}ABB^{†}A^{†}A=B{\left(AB\right)}^{†}A=BB^{†}A^{†}A,{\left(A^{†}ABB^{†}(BB^{†}A^{†}A)\right)}^{⁎}={\left(A^{†}ABB^{†}A^{†}A\right)}^{⁎}=A^{†}ABB^{†}A^{†}A=A^{†}ABB^{†}(BB^{†}A^{†}A),{\left((BB^{†}A^{†}A)A^{†}ABB^{†}\right)}^{⁎}={\left(BB^{†}A^{†}ABB^{†}\right)}^{⁎}=BB^{†}A^{†}ABB^{†}=(BB^{†}A^{†}A)A^{†}ABB^{†}$$

hold, which by definition mean that

(11.4) $${\left(A^{†}ABB^{†}\right)}^{†}=BB^{†}A^{†}A\;\;\mathrm{or equivalently}\;\;A^{†}ABB^{†}={\left(BB^{†}A^{†}A\right)}^{†}$$

holds. Subsequently, substituting it into (11.1) yields

(11.5) $${\left(AB\right)}^{†}=B^{†}A^{†}=B^{†}A^{†}ABB^{†}A^{†}=B^{†}(A^{†}ABB^{†})A^{†}=B^{†}{\left(BB^{†}A^{†}A\right)}^{†}A^{†}.$$

Combining (11.4) and (11.5) leads to 〈7〉. Conversely, merging the two equalities in 〈7〉 and simplifying lead to

$${\left(AB\right)}^{†}=B^{†}{\left(A^{†}ABB^{†}\right)}^{†}A^{†}=B^{†}BB^{†}A^{†}AA^{†}=B^{†}A^{†},$$

establishing 〈1〉.

The equivalence of 〈1〉 and 〈8〉 follows from (3.70) and $r(AB)=r(B^{†}A^{†})$. The equivalence of 〈8〉 and 〈10〉 follows from (3.65) and (3.66).

Result 〈1〉 obviously implies 〈9〉. Conversely, pre- and post-multiplying $B^{†}B$ and $AA^{†}$ to both sides of $M^{†}=B^{\left(1,3\right)}A^{\left(1,4\right)}$ leads to

$$M^{†}=B^{†}BM^{†}AA^{†}=B^{†}BB^{\left(1,3\right)}A^{\left(1,4\right)}AA^{†}=B^{†}BB^{†}A^{†}AA^{†}=B^{†}A^{†}.$$

Hence 〈9〉 implies 〈1〉.

If 〈1〉 holds, then it is easy to verify from 〈1〉–〈8〉 and 〈10〉 the following four groups of equalities

$${\left(A^{†}\right)}^{⁎}BB^{†}A^{⁎}{\left(A^{†}\right)}^{⁎}B={\left(A^{†}\right)}^{⁎}(BB^{†}A^{†}A)B={\left(A^{†}\right)}^{⁎}(A^{†}ABB^{†})AB={\left(A^{†}\right)}^{⁎}B,B^{†}A^{⁎}{\left(A^{†}\right)}^{⁎}BB^{†}A^{⁎}=B^{†}(A^{†}ABB^{†})A^{⁎}=B^{†}(BB^{†}A^{†}A)A^{⁎}=B^{†}A^{⁎},{\left({\left(A^{†}\right)}^{⁎}BB^{†}A^{⁎}\right)}^{⁎}=ABB^{†}A^{†}={\left(ABB^{†}A^{†}\right)}^{⁎}={\left(A^{†}\right)}^{⁎}BB^{†}A^{⁎},{\left(B^{†}A^{⁎}{\left(A^{†}\right)}^{⁎}B\right)}^{⁎}=B^{⁎}A^{†}A{\left(B^{†}\right)}^{⁎}=B^{†}A^{†}AB=B^{†}A^{⁎}{\left(A^{†}\right)}^{⁎}B$$

hold, which by definition mean that 〈11〉 holds. The equivalences of 〈12〉–〈19〉 can be established by replacing *A* with ${\left(A^{†}\right)}^{⁎}$ in 〈1〉–〈8〉 and 〈10〉.

The equivalences of 〈1〉 and 〈20〉–〈37〉 can be established by a similar way.

If 〈1〉 holds, then it is easy to verify the following four equalities

$${\left(A^{⁎}A\right)}^{k}{\left(BB^{⁎}\right)}^{k}{\left({\left(BB^{⁎}\right)}^{k}\right)}^{†}{\left({\left(A^{⁎}A\right)}^{k}\right)}^{†}{\left(A^{⁎}A\right)}^{k}{\left(BB^{⁎}\right)}^{k}={\left(A^{⁎}A\right)}^{k}BB^{†}A^{†}A{\left(BB^{⁎}\right)}^{k}={\left(A^{⁎}A\right)}^{k-1}A^{⁎}(ABB^{†}A^{†}AB)B^{⁎}{\left(BB^{⁎}\right)}^{k-1}={\left(A^{⁎}A\right)}^{k-1}A^{⁎}ABB^{⁎}{\left(BB^{⁎}\right)}^{k-1}={\left(A^{⁎}A\right)}^{k}{\left(BB^{⁎}\right)}^{k},{\left({\left(BB^{⁎}\right)}^{k}\right)}^{†}{\left({\left(A^{⁎}A\right)}^{k}\right)}^{†}{\left(A^{⁎}A\right)}^{k}{\left(BB^{⁎}\right)}^{k}{\left({\left(BB^{⁎}\right)}^{k}\right)}^{†}{\left({\left(A^{⁎}A\right)}^{k}\right)}^{†}={\left({\left(BB^{⁎}\right)}^{k}\right)}^{†}A^{†}ABB^{†}{\left({\left(A^{⁎}A\right)}^{k}\right)}^{†}={\left({\left(BB^{⁎}\right)}^{k-1}\right)}^{†}{\left(B^{†}\right)}^{⁎}B^{†}A^{†}ABB^{†}A^{†}{\left(A^{†}\right)}^{⁎}{\left({\left(A^{⁎}A\right)}^{k-1}\right)}^{†}={\left({\left(BB^{⁎}\right)}^{k-1}\right)}^{†}{\left(BB^{⁎}\right)}^{†}{\left(A^{⁎}A\right)}^{†}{\left({\left(A^{⁎}A\right)}^{k-1}\right)}^{†}={\left({\left(BB^{⁎}\right)}^{k}\right)}^{†}{\left({\left(A^{⁎}A\right)}^{k}\right)}^{†},$$

$${\left({\left(A^{⁎}A\right)}^{k}{\left(BB^{⁎}\right)}^{k}{\left({\left(BB^{⁎}\right)}^{k}\right)}^{†}{\left({\left(A^{⁎}A\right)}^{k}\right)}^{†}\right)}^{⁎}={\left({\left(A^{⁎}A\right)}^{k}\right)}^{†}{\left(BB^{⁎}\right)}^{k}{\left({\left(BB^{⁎}\right)}^{k}\right)}^{†}{\left(A^{⁎}A\right)}^{k}={\left({\left(A^{⁎}A\right)}^{k}\right)}^{†}BB^{†}{\left(A^{⁎}A\right)}^{k}={\left({\left(A^{⁎}A\right)}^{k}\right)}^{†}{\left(A^{⁎}A\right)}^{k}BB^{†},=A^{†}ABB^{†}=BB^{†}A^{†}A={\left(A^{⁎}A\right)}^{k}{\left(BB^{⁎}\right)}^{k}{\left({\left(BB^{⁎}\right)}^{k}\right)}^{†}{\left({\left(A^{⁎}A\right)}^{k}\right)}^{†},{\left({\left({\left(BB^{⁎}\right)}^{k}\right)}^{†}{\left({\left(A^{⁎}A\right)}^{k}\right)}^{†}{\left(A^{⁎}A\right)}^{k}{\left(BB^{⁎}\right)}^{k}\right)}^{⁎}={\left(BB^{⁎}\right)}^{k}{\left({\left(A^{⁎}A\right)}^{k}\right)}^{†}{\left(A^{⁎}A\right)}^{k}{\left({\left(BB^{⁎}\right)}^{k}\right)}^{†}={\left(BB^{⁎}\right)}^{k}A^{†}A{\left({\left(BB^{⁎}\right)}^{k}\right)}^{†}=A^{†}A{\left(BB^{⁎}\right)}^{k}{\left({\left(BB^{⁎}\right)}^{k}\right)}^{†}=A^{†}ABB^{†}=BB^{†}A^{†}A={\left({\left(BB^{⁎}\right)}^{k}\right)}^{†}{\left({\left(A^{⁎}A\right)}^{k}\right)}^{†}{\left(A^{⁎}A\right)}^{k}{\left(BB^{⁎}\right)}^{k}$$

hold, which by definition mean that the second equality in 〈39〉 holds. Subsequently, substituting the second equality into (11.1) yields

$${\left(AB\right)}^{†}=B^{†}A^{†}=B^{⁎}{\left(BB^{⁎}\right)}^{†}{\left(A^{⁎}A\right)}^{†}A^{⁎}=B^{⁎}{\left(BB^{⁎}\right)}^{k-1}{\left({\left(BB^{⁎}\right)}^{k}\right)}^{†}{\left({\left(A^{⁎}A\right)}^{k}\right)}^{†}{\left(A^{⁎}A\right)}^{k-1}A^{⁎}=B^{⁎}{\left(BB^{⁎}\right)}^{k-1}{\left({\left(A^{⁎}A\right)}^{k}{\left(BB^{⁎}\right)}^{k}\right)}^{†}{\left(A^{⁎}A\right)}^{k-1}A^{⁎},$$

establishing first equality in 〈38〉. Conversely, merging the two equalities in 〈38〉 and simplifying lead to 〈1〉. The equivalence of 〈1〉 to 〈39〉 can be shown by a similar way.

Finally, applying (4.13) to the difference of $AA^{⁎}{\left(B^{⁎}A^{⁎}\right)}^{†}B^{⁎}B-AB$ gives

$$r\left(AB-AA^{⁎}{\left(B^{⁎}A^{⁎}\right)}^{†}B^{⁎}B\right)=r\left[\begin{array}{cc} AB{\left(AB\right)}^{⁎}AB & ABB^{⁎}B\\ AA^{⁎}AB & AB \end{array}\right]-r(AB).$$

Setting both sides of this rank equality equal to zero leads to the equivalence of 〈1〉 and 〈40〉. □

The family of results in Theorem 11.1 demonstrate that the ROL in (11.1) is equivalent to many other matrix equalities, matrix set inclusions, as well as a rank equality that is composed of *A*, *B*, *AB*, and their Moore–Penrose inverses, including the three pairs of nested equalities in 〈5〉, 〈6〉, and 〈7〉. These equivalent assertions link many equalities and facts together, so that people can touch the ROL from different aspects. Moreover, for each of these matrix equalities and matrix set inclusions, we can separately derive conditions under which the equality or matrix set inclusion holds, so that we are able to obtain various equivalent facts for (11.1) to hold by merging the separate conditions obtained. In fact, we have obtained in Theorem 9.1〈98〉 that the second equality in Theorem 11.1〈7〉 and other 164 groups of facts are equivalent.

To further characterize the role of the first equality in Theorem 11.1〈7〉, we present in the following three theorems a rich variety of algebraic equalities and facts that are equivalent to the equality.

Lemma 11.2 *Let* $A\in C^{m\times n}$ *and* $B\in C^{n\times p}$*. Then the following 57 statements are equivalent:*

〈1〉 $\left\{{\left(AB\right)}^{\left(1,2,3\right)}\right\}∋{\left(A^{†}AB\right)}^{†}A^{†}$*.*
〈2〉 $\left\{{\left(AB\right)}^{\left(1,2,3\right)}\right\}∋{\left(A^{⁎}AB\right)}^{†}A^{⁎}$*.*
〈3〉 $\left\{{\left(A^{†}AB\right)}^{\left(1,2,3\right)}\right\}∋{\left(AB\right)}^{†}A$*.*
〈4〉 $\left\{{\left(A^{⁎}AB\right)}^{\left(1,2,3\right)}\right\}∋{\left(AB\right)}^{†}{\left(A^{†}\right)}^{⁎}$*.*
〈5〉 $\left\{{\left(AB\right)}^{\left(1,2,3\right)}\right\}∋B^{†}{\left(A^{†}ABB^{†}\right)}^{†}A^{†}$*.*
〈6〉 $\left\{{\left(AB\right)}^{\left(1,2,3\right)}\right\}∋B^{⁎}{\left(A^{⁎}ABB^{⁎}\right)}^{†}A^{⁎}$*.*
〈7〉 $\left\{{\left(A^{†}ABB^{†}\right)}^{\left(1,2,3\right)}\right\}∋B{\left(AB\right)}^{†}A$*.*
〈8〉 $\left\{{\left(A^{⁎}ABB^{⁎}\right)}^{\left(1,2,3\right)}\right\}∋{\left(B^{†}\right)}^{⁎}{\left(AB\right)}^{†}{\left(A^{†}\right)}^{⁎}$*.*
〈9〉 ${\left(AB\right)}^{†}={\left(A^{†}AB\right)}^{†}A^{†}$*.*
〈10〉 ${\left(A^{†}AB\right)}^{†}={\left(AB\right)}^{†}A$*.*
〈11〉 ${\left(AB\right)}^{†}={\left(A^{⁎}AB\right)}^{†}A^{⁎}$*.*
〈12〉 ${\left(A^{⁎}AB\right)}^{†}={\left(AB\right)}^{†}{\left(A^{†}\right)}^{⁎}$*.*
〈13〉 $B^{†}A^{†}AB{\left(AB\right)}^{†}=B^{†}A^{†}$*.*
〈14〉 ${\left(B^{†}A^{†}\right)}^{†}B^{†}A^{†}AB=AB$*.*
〈15〉 $AB{\left(AB\right)}^{†}={\left(B^{†}A^{†}\right)}^{†}B^{†}A^{†}$*.*
〈16〉 $AB{\left(A^{†}AB\right)}^{†}A^{†}$ *is an orthogonal projector.*
〈17〉 $AB{\left(A^{⁎}AB\right)}^{†}A^{⁎}$ *is an orthogonal projector.*
〈18〉 $A^{†}(AB){\left(AB\right)}^{†}A$ *is an orthogonal projector.*
〈19〉 $A{\left(A^{†}ABB^{†}\right)}^{†}A^{†}$ *is an orthogonal projector.*
〈20〉 $ABB^{⁎}{\left(A^{⁎}ABB^{⁎}\right)}^{†}A^{⁎}$ *is an orthogonal projector.*
〈21〉 $AB{\left(AB\right)}^{†}$ *and* $AA^{⁎}$ *commute.*
〈22〉 $AB{\left(A^{†}AB\right)}^{†}A^{†}$ *and* $AA^{⁎}$ *commute.*
〈23〉 $AB{\left(A^{⁎}AB\right)}^{†}A^{⁎}$ *and* $AA^{⁎}$ *commute.*
〈24〉 $A^{†}(AB){\left(AB\right)}^{†}A$ *and* $A^{⁎}A$ *commute.*
〈25〉 $ABB^{†}A^{†}$ *is EP.*
〈26〉 $R(AB)=R({\left(B^{†}A^{†}\right)}^{⁎})$*.*
〈27〉 $R(AB)=R\left({\left(A^{†}\right)}^{⁎}{\left(B^{⁎}A^{†}A\right)}^{†}\right)$*.*
〈28〉 $R(AB)=R\left(A{\left(B^{⁎}A^{⁎}A\right)}^{†}\right)$*.*
〈29〉 $R(AB)=R\left({\left(A^{†}\right)}^{⁎}{\left(BB^{†}A^{†}A\right)}^{†}{\left(B^{†}\right)}^{⁎}\right)$*.*
〈30〉 $R(AB)=R\left(A{\left(BB^{⁎}A^{⁎}A\right)}^{†}B\right)$*.*
〈31〉 $\left\{{\left({\left(A^{†}\right)}^{⁎}B\right)}^{\left(1,2,3\right)}\right\}∋{\left(A^{†}AB\right)}^{†}A^{⁎}$*.*
〈32〉 $\left\{{\left({\left(A^{†}\right)}^{⁎}B\right)}^{\left(1,2,3\right)}\right\}∋{\left({\left(A^{⁎}A\right)}^{†}B\right)}^{†}A^{†}$*.*
〈33〉 $\left\{{\left(A^{†}AB\right)}^{\left(1,2,3\right)}\right\}∋{\left({\left(A^{†}\right)}^{⁎}B\right)}^{†}{\left(A^{†}\right)}^{⁎}$*.*
〈34〉 $\left\{{\left({\left(A^{⁎}A\right)}^{†}B\right)}^{\left(1,2,3\right)}\right\}∋{\left({\left(A^{†}\right)}^{⁎}B\right)}^{†}A$*.*
〈35〉 $\left\{{\left({\left(A^{†}\right)}^{⁎}B\right)}^{\left(1,2,3\right)}\right\}∋B^{†}{\left(A^{†}ABB^{†}\right)}^{†}A^{⁎}$*.*
〈36〉 $\left\{{\left({\left(A^{†}\right)}^{⁎}B\right)}^{\left(1,2,3\right)}\right\}∋B^{⁎}{\left({\left(A^{⁎}A\right)}^{†}BB^{⁎}\right)}^{†}A^{†}$*.*
〈37〉 $\left\{{\left(A^{†}ABB^{†}\right)}^{\left(1,2,3\right)}\right\}∋B{\left({\left(A^{†}\right)}^{⁎}B\right)}^{†}{\left(A^{†}\right)}^{⁎}$*.*
〈38〉 $\left\{{\left({\left(A^{⁎}A\right)}^{†}BB^{⁎}\right)}^{\left(1,2,3\right)}\right\}∋{\left(B^{†}\right)}^{⁎}\left({\left(A^{†}\right)}^{⁎}B\right)A$*.*
〈39〉 ${\left({\left(A^{†}\right)}^{⁎}B\right)}^{†}={\left(A^{†}AB\right)}^{†}A^{⁎}$*.*
〈40〉 ${\left({\left(A^{†}\right)}^{⁎}B\right)}^{†}={\left({\left(A^{⁎}A\right)}^{†}B\right)}^{†}A^{†}$*.*
〈41〉 ${\left(A^{†}AB\right)}^{†}={\left({\left(A^{†}\right)}^{⁎}B\right)}^{†}{\left(A^{†}\right)}^{⁎}$*.*
〈42〉 ${\left({\left(A^{⁎}A\right)}^{†}B\right)}^{†}={\left({\left(A^{†}\right)}^{⁎}B\right)}^{†}A$*.*
〈43〉 $A{\left(B^{†}A^{†}A\right)}^{†}B^{†}A^{†}$ *is an orthogonal projector.*
〈44〉 ${\left(A^{†}\right)}^{⁎}{\left(B^{†}{\left(A^{⁎}A\right)}^{†}\right)}^{†}B^{†}A^{†}$ *is an orthogonal projector.*
〈45〉 $A^{†}{\left(B^{†}A^{†}\right)}^{†}(B^{†}A^{†})A$ *is an orthogonal projector.*
〈46〉 $A{\left(BB^{†}A^{†}A\right)}^{†}A^{†}$ *is an orthogonal projector.*
〈47〉 ${\left(A^{†}\right)}^{⁎}{\left(BB^{⁎}\right)}^{†}{\left({\left(A^{⁎}A\right)}^{†}{\left(BB^{⁎}\right)}^{†}\right)}^{†}A^{†}$ *is an orthogonal projector.*
〈48〉 ${\left(B^{†}A^{†}\right)}^{†}(B^{†}A^{†})$ *and* ${\left(AA^{⁎}\right)}^{†}$ *commute.*
〈49〉 $A{\left(B^{†}A^{†}A\right)}^{†}B^{†}A^{†}$ *and* ${\left(AA^{⁎}\right)}^{†}$ *commute.*
〈50〉 ${\left(A^{†}\right)}^{⁎}{\left(B^{†}{\left(A^{⁎}A\right)}^{†}\right)}^{†}B^{†}A^{†}$ *and* ${\left(AA^{⁎}\right)}^{†}$ *commute.*
〈51〉 $A^{†}{\left(B^{†}A^{†}\right)}^{†}(B^{†}A^{†})A$ *and* ${\left(A^{⁎}A\right)}^{†}$ *commute.*
〈52〉 $R\left({\left(B^{†}A^{†}\right)}^{†}\right)=R\left(A{\left(B^{†}A^{†}A\right)}^{†}\right)$*.*
〈53〉 $R\left({\left(B^{†}A^{†}\right)}^{†}\right)=R\left({\left(A^{†}\right)}^{⁎}{\left(B^{†}{\left(A^{⁎}A\right)}^{†}\right)}^{†}\right)$*.*
〈54〉 $R\left({\left(B^{†}A^{†}\right)}^{†}\right)=R\left(A{\left(BB^{†}A^{†}A\right)}^{†}B\right)$*.*
〈55〉 $R\left({\left(B^{†}A^{†}\right)}^{†}\right)=R\left({\left(A^{†}\right)}^{⁎}{\left({\left(BB^{⁎}\right)}^{†}{\left(A^{⁎}A\right)}^{†}\right)}^{†}{\left(B^{†}\right)}^{⁎}\right)$*.*
〈56〉 $R(AA^{⁎}AB)=R(AB)$*.*
〈57〉 $r[AA^{⁎}AB,\;AB]=r(AB)$*.*

Proof It is obvious by definition that

$$AB{\left(AB\right)}^{†},\;\;(A^{†}AB){\left(A^{†}AB\right)}^{†},\;\;(A^{⁎}AB){\left(A^{⁎}AB\right)}^{†},\;\;(A^{†}ABB^{†}){\left(A^{†}ABB^{†}\right)}^{†},\;\;(A^{⁎}ABB^{⁎}){\left(A^{⁎}ABB^{⁎}\right)}^{†}$$

are idempotent matrices, and it is easy to verify by definition that

$$AB{\left(A^{†}AB\right)}^{†}A^{†},\;\;AB{\left(A^{⁎}AB\right)}^{†}A^{⁎},\;\;A^{†}AB{\left(AB\right)}^{†}A,\;\;(A^{⁎}AB){\left(AB\right)}^{†}{\left(A^{†}\right)}^{⁎},\;\;ABB^{†}{\left(A^{†}ABB^{†}\right)}^{†}A^{†},\;\;\;\;\;\;\;\;\;\;ABB^{⁎}{\left(A^{⁎}ABB^{⁎}\right)}^{†}A^{⁎},\;\;(A^{†}ABB^{†})B{\left(AB\right)}^{†}A,\;\;(A^{⁎}ABB^{⁎}){\left(B^{†}\right)}^{⁎}{\left(AB\right)}^{†}{\left(A^{†}\right)}^{⁎}$$

are idempotent matrices. Thus we can obtain by (4.23) the following rank equalities

(11.6) $$r\left(AB{\left(A^{†}AB\right)}^{†}A^{†}-AB{\left(AB\right)}^{†}\right)\;\;=r\left[\begin{array}{c} AB{\left(A^{†}AB\right)}^{†}A^{†}\\ AB{\left(AB\right)}^{†} \end{array}\right]+r[AB{\left(A^{†}AB\right)}^{†}A^{†},\;AB{\left(AB\right)}^{†}]-r(AB{\left(A^{†}AB\right)}^{†}A^{†})-r(AB{\left(AB\right)}^{†})\;\;=r[AA^{⁎}AB,\;AB]-r(AB),$$

(11.7) $$r\left(AB{\left(A^{⁎}AB\right)}^{†}A^{⁎}-AB{\left(AB\right)}^{†}\right)\;\;=r\left[\begin{array}{c} AB{\left(A^{⁎}AB\right)}^{†}A^{⁎}\\ AB{\left(AB\right)}^{†} \end{array}\right]+r[AB{\left(A^{⁎}AB\right)}^{†}A^{⁎},\;AB{\left(AB\right)}^{†}]-r(AB{\left(A^{⁎}AB\right)}^{†}A^{⁎})-r(AB{\left(AB\right)}^{†})\;\;=r[AA^{⁎}AB,\;AB]-r(AB),$$

(11.8) $$r\left(A^{†}AB{\left(AB\right)}^{†}A-(A^{†}AB){\left(A^{†}AB\right)}^{†}\right)\;\;=r\left[\begin{array}{c} A^{†}AB{\left(AB\right)}^{†}A\\ (A^{†}AB){\left(A^{†}AB\right)}^{†} \end{array}\right]+r[A^{†}AB{\left(AB\right)}^{†}A,\;(A^{†}AB){\left(A^{†}AB\right)}^{†}]-r(A^{†}AB{\left(AB\right)}^{†}A)-r((A^{†}AB){\left(A^{†}AB\right)}^{†})\;\;=r[AA^{⁎}AB,\;AB]-r(AB),$$

(11.9) $$r\left((A^{⁎}AB){\left(AB\right)}^{†}{\left(A^{†}\right)}^{⁎}-(A^{⁎}AB){\left(A^{⁎}AB\right)}^{†}\right)\;\;=r\left[\begin{array}{c} (A^{⁎}AB){\left(AB\right)}^{†}{\left(A^{†}\right)}^{⁎}\\ (A^{⁎}AB){\left(A^{⁎}AB\right)}^{†} \end{array}\right]+r[(A^{⁎}AB){\left(AB\right)}^{†}{\left(A^{†}\right)}^{⁎},\;(A^{⁎}AB){\left(A^{⁎}AB\right)}^{†}]-r((A^{⁎}AB){\left(AB\right)}^{†}{\left(A^{†}\right)}^{⁎})-r((A^{⁎}AB){\left(A^{⁎}AB\right)}^{†})\;\;=r[AA^{⁎}AB,\;AB]-r(AB),$$

(11.10) $$r\left(ABB^{†}{\left(A^{†}ABB^{†}\right)}^{†}A^{†}-AB{\left(AB\right)}^{†}\right)\;\;=r\left[\begin{array}{c} ABB^{†}{\left(A^{†}ABB^{†}\right)}^{†}A^{†}\\ AB{\left(AB\right)}^{†} \end{array}\right]+r[ABB^{†}{\left(A^{†}ABB^{†}\right)}^{†}A^{†},\;AB{\left(AB\right)}^{†}]-r(ABB^{†}{\left(A^{†}ABB^{†}\right)}^{†}A^{†})-r(AB{\left(AB\right)}^{†})\;\;=r[AA^{⁎}AB,\;AB]-r(AB),$$

(11.11) $$r\left(ABB^{⁎}{\left(A^{⁎}ABB^{⁎}\right)}^{†}A^{⁎}-AB{\left(AB\right)}^{†}\right)\;\;=r\left[\begin{array}{c} ABB^{⁎}{\left(A^{⁎}ABB^{⁎}\right)}^{†}A^{⁎}\\ AB{\left(AB\right)}^{†} \end{array}\right]+r[ABB^{⁎}{\left(A^{⁎}ABB^{⁎}\right)}^{†}A^{⁎},\;AB{\left(AB\right)}^{†}]-r(ABB^{⁎}{\left(A^{⁎}ABB^{⁎}\right)}^{†}A^{⁎})-r(AB{\left(AB\right)}^{†})\;\;=r[AA^{⁎}AB,\;AB]-r(AB),$$

(11.12) $$r\left(A^{†}ABB^{†}B{\left(AB\right)}^{†}A-(A^{†}ABB^{†}){\left(A^{†}ABB^{†}\right)}^{†}\right)\;\;=r\left[\begin{array}{c} A^{†}ABB^{†}B{\left(AB\right)}^{†}A\\ (A^{†}ABB^{†}){\left(A^{†}ABB^{†}\right)}^{†} \end{array}\right]+r[A^{†}ABB^{†}B{\left(AB\right)}^{†}A,\;(A^{†}ABB^{†}){\left(A^{†}ABB^{†}\right)}^{†}]-r(A^{†}ABB^{†}B{\left(AB\right)}^{†}A)-r((A^{†}ABB^{†}){\left(A^{†}ABB^{†}\right)}^{†})\;\;=r[AA^{⁎}AB,\;AB]-r(AB),$$

(11.13) $$r\left(A^{⁎}ABB^{⁎}{\left(B^{†}\right)}^{⁎}{\left(AB\right)}^{†}{\left(A^{†}\right)}^{⁎}-(A^{⁎}ABB^{⁎}){\left(A^{⁎}ABB^{⁎}\right)}^{†}\right)\;\;=r\left[\begin{array}{c} A^{⁎}ABB^{⁎}{\left(B^{†}\right)}^{⁎}{\left(AB\right)}^{†}{\left(A^{†}\right)}^{⁎}\\ (A^{⁎}ABB^{⁎}){\left(A^{⁎}ABB^{⁎}\right)}^{†} \end{array}\right]+r[A^{⁎}ABB^{⁎}{\left(B^{†}\right)}^{⁎}{\left(AB\right)}^{†}{\left(A^{†}\right)}^{⁎},\;(A^{⁎}ABB^{⁎}){\left(A^{⁎}ABB^{⁎}\right)}^{†}]-r(A^{⁎}ABB^{⁎}{\left(B^{†}\right)}^{⁎}{\left(AB\right)}^{†}{\left(A^{†}\right)}^{⁎})-r((A^{⁎}ABB^{⁎}){\left(A^{⁎}ABB^{⁎}\right)}^{†})\;\;=r[AA^{⁎}AB,\;AB]-r(AB).$$

Setting both sides of (11.6)–(11.13) equal to zero leads to the equivalences of 〈1〉–〈8〉, 〈56〉, and 〈57〉.

It is easy to verify that ${\left(AB\right)}^{†}$, ${\left(A^{†}AB\right)}^{†}A^{†}$, and ${\left(A^{⁎}AB\right)}^{†}A^{⁎}$ are {2}-inverses of *AB*. Then it follows from (4.23) that

(11.14) $$r\left({\left(AB\right)}^{†}-{\left(A^{†}AB\right)}^{†}A^{†}\right)=r\left({\left(AB\right)}^{†}A-{\left(A^{†}AB\right)}^{†}\right)=r\left[\begin{array}{c} {\left(AB\right)}^{†}\\ {\left(A^{†}AB\right)}^{†}A^{†} \end{array}\right]+r[{\left(AB\right)}^{†},\;{\left(A^{†}AB\right)}^{†}A^{†}]-r({\left(AB\right)}^{†})-r\left({\left(A^{†}AB\right)}^{†}A^{†}\right)=r\left[\begin{array}{c} {\left(AB\right)}^{⁎}\\ {\left(A^{†}AB\right)}^{⁎}A^{†} \end{array}\right]+r[{\left(AB\right)}^{⁎},\;{\left(A^{†}AB\right)}^{⁎}]-2r(AB)=r\left[\begin{array}{c} {\left(AB\right)}^{⁎}AA^{⁎}\\ {\left(AB\right)}^{⁎} \end{array}\right]-r(AB)=r[AA^{⁎}AB,\;AB]-r(AB),$$

and

(11.15) $$r\left({\left(AB\right)}^{†}-{\left(A^{⁎}AB\right)}^{†}A^{⁎}\right)=r\left({\left(AB\right)}^{†}{\left(A^{†}\right)}^{⁎}-{\left(A^{⁎}AB\right)}^{†}\right)=r\left[\begin{array}{c} {\left(AB\right)}^{†}\\ {\left(A^{⁎}AB\right)}^{†}A^{⁎} \end{array}\right]+r\left[{\left(AB\right)}^{†},\;{\left(A^{⁎}AB\right)}^{†}A^{⁎}\right]-r({\left(AB\right)}^{†})-r\left({\left(A^{⁎}AB\right)}^{†}A^{⁎}\right)=r\left[\begin{array}{c} {\left(AB\right)}^{⁎}\\ {\left(A^{⁎}AB\right)}^{⁎}A^{⁎} \end{array}\right]+r[{\left(AB\right)}^{⁎},\;{\left(A^{⁎}AB\right)}^{⁎}]-2r(AB)=r\left[\begin{array}{c} {\left(AB\right)}^{⁎}\\ {\left(AA^{⁎}AB\right)}^{⁎} \end{array}\right]-r(AB)=r[AA^{⁎}AB,\;AB]-r(AB).$$

Setting all sides of (11.14) and (11.15) equal to zero leads to the equivalences of 〈9〉–〈12〉, and 〈57〉.

By (4.4), (4.5), (4.23), and Lemma 8.6,

(11.16) $$r\left(B^{†}A^{†}AB{\left(AB\right)}^{†}-B^{†}A^{†}\right)=r[{\left(B^{†}A^{†}\right)}^{⁎},\;AB]-r\left({\left(B^{†}A^{†}\right)}^{⁎}\right)=r[AA^{⁎}AB,\;AB]-r(AB),$$

(11.17) $$r\left({\left(B^{†}A^{†}\right)}^{†}B^{†}A^{†}AB-AB\right)=r[{\left(B^{†}A^{†}\right)}^{⁎},\;AB]-r\left({\left(B^{†}A^{†}\right)}^{⁎}\right)=r[AA^{⁎}AB,\;AB]-r(AB),$$

(11.18) $$r\left(AB{\left(AB\right)}^{†}-{\left(B^{†}A^{†}\right)}^{†}B^{†}A^{†}\right)=r\left[\begin{array}{c} (AB{\left(AB\right)}^{†}\\ {\left(B^{†}A^{†}\right)}^{†}B^{†}A^{†} \end{array}\right]+r\left[(AB{\left(AB\right)}^{†},\;{\left(B^{†}A^{†}\right)}^{†}B^{†}A^{†}\right]-r\left(AB{\left(AB\right)}^{†}\right)-r\left({\left(B^{†}A^{†}\right)}^{†}B^{†}A^{†}\right)=2r[AA^{⁎}AB,\;AB]-2r(AB).$$

Setting all sides of (11.16)–(11.18) equal to zero leads to the equivalences of 〈13〉–〈15〉 and 〈57〉.

It is easy to verify by definition that $AB{\left(A^{†}AB\right)}^{†}A^{†}$, $AB{\left(A^{⁎}AB\right)}^{†}A^{⁎}$, and $A^{†}AB{\left(AB\right)}^{†}A$ are idempotent. Then by (4.23) and Lemma 8.6,

(11.19) $$r\left({\left(AB{\left(A^{†}AB\right)}^{†}A^{†}\right)}^{⁎}-AB{\left(A^{†}AB\right)}^{†}A^{†}\right)=2r\left[{\left(AB{\left(A^{†}AB\right)}^{†}A^{†}\right)}^{⁎},\;AB{\left(A^{†}AB\right)}^{†}A^{†}\right]-2r\left(AB{\left(A^{†}AB\right)}^{†}A^{†}\right)=2r\left[{\left(A^{†}\right)}^{⁎}{\left(B^{⁎}A^{†}A\right)}^{†}B^{⁎}A^{⁎},\;AB\right]-2r(AB)=2r\left[{\left(A^{†}\right)}^{⁎}{\left(B^{⁎}A^{†}A\right)}^{⁎},\;AB\right]-2r(AB)=2r\left[A^{†}B,\;AB\right]-2r(AB)=2r[AA^{⁎}AB,\;AB]-2r(AB),$$

(11.20) $$r\left({\left(AB{\left(A^{⁎}AB\right)}^{†}A^{⁎}\right)}^{⁎}-AB{\left(A^{⁎}AB\right)}^{†}A^{⁎}\right)=2r\left[{\left(AB{\left(A^{⁎}AB\right)}^{†}A^{⁎}\right)}^{⁎},\;AB{\left(A^{⁎}AB\right)}^{†}A^{⁎}\right]-2r\left(AB{\left(A^{⁎}AB\right)}^{†}A^{⁎}\right)=2r\left[A{\left(B^{⁎}A^{⁎}A\right)}^{†}B^{⁎}A^{⁎},\;AB\right]-2r(AB)=2r\left(A{\left(B^{⁎}A^{⁎}A\right)}^{⁎},\;AB\right)-2r(AB)=2r[AA^{⁎}AB,\;AB]-2r(AB),$$

(11.21) $$r\left({\left(A^{†}AB{\left(AB\right)}^{†}A\right)}^{⁎}-A^{†}AB{\left(AB\right)}^{†}A\right)=r\left(A^{⁎}AB{\left(AB\right)}^{†}{\left(A^{†}\right)}^{⁎}-A^{†}AB{\left(AB\right)}^{†}A\right)=r\left(AA^{⁎}(AB){\left(AB\right)}^{†}-(AB){\left(AB\right)}^{†}AA^{⁎}\right)=2r\left[A^{⁎}AB{\left(AB\right)}^{†}{\left(A^{†}\right)}^{⁎},\;A^{†}AB{\left(AB\right)}^{†}A\right]-2r\left(A^{†}AB{\left(AB\right)}^{†}A\right)=2r\left[AA^{⁎}AB{\left(AB\right)}^{†},\;AB{\left(AB\right)}^{†}\right]-2r(AB)=2r[AA^{⁎}AB,\;AB]-2r(AB).$$

Setting all sides of (11.19), (11.20), and (11.21) equal to zero and combining them with the idempotency of the matrices lead to the equivalences of 〈16〉–〈18〉, 〈21〉, and 〈57〉.

The equivalence of 〈16〉 and 〈19〉 follows $A{\left(A^{†}ABB^{†}\right)}^{†}=AB{\left(A^{†}AB\right)}^{†}$.

The equivalence of 〈17〉 and 〈20〉 follows $ABB^{⁎}{\left(A^{⁎}ABB^{⁎}\right)}^{†}A^{⁎}=AB{\left(A^{⁎}AB\right)}^{†}A^{⁎}$.

By the idempotency of $AB{\left(A^{†}AB\right)}^{†}A^{†}$, $AB{\left(A^{⁎}AB\right)}^{†}A^{⁎}$, and $A^{†}AB{\left(AB\right)}^{†}A$, and (4.23) and Lemma 8.6,

(11.22) $$r\left(AB{\left(A^{†}AB\right)}^{†}A^{†}AA^{⁎}-AA^{⁎}AB{\left(A^{†}AB\right)}^{†}A^{†}AA^{⁎}\right)=r\left[\begin{array}{c} AB{\left(A^{†}AB\right)}^{†}A^{†}AA^{⁎}\\ {\left(A^{†}AB\right)}^{†}A^{†}AA^{⁎} \end{array}\right]+r[AA^{⁎}AB{\left(A^{†}AB\right)}^{†}A^{†}AA^{⁎},\;AB{\left(A^{†}AB\right)}^{†}A^{†}AA^{⁎}]-2r(AB{\left(A^{†}AB\right)}^{†}A^{†})=2r[AA^{⁎}AB,\;AB]-2r(AB),$$

(11.23) $$r\left(AB{\left(A^{†}AB\right)}^{†}A^{†}AA^{⁎}-AA^{⁎}AB{\left(A^{†}AB\right)}^{†}A^{†}AA^{⁎}\right)=r\left[\begin{array}{c} AB{\left(A^{⁎}AB\right)}^{†}A^{⁎}AA^{⁎}\\ {\left(A^{⁎}AB\right)}^{†}A^{⁎}AA^{⁎} \end{array}\right]+r[AA^{⁎}AB{\left(A^{⁎}AB\right)}^{†}A^{⁎}AA^{⁎},\;AB{\left(A^{⁎}AB\right)}^{†}A^{⁎}AA^{⁎}]-2r(AB{\left(A^{⁎}AB\right)}^{†}A^{⁎})=2r[AA^{⁎}AB,\;AB]-2r(AB),$$

(11.24) $$r\left(A^{†}(AB){\left(AB\right)}^{†}AA^{⁎}A-A^{⁎}AA^{†}(AB){\left(AB\right)}^{†}A\right)=r\left[\begin{array}{c} A^{†}(AB){\left(AB\right)}^{†}AA^{⁎}A\\ A^{†}(AB){\left(AB\right)}^{†}A \end{array}\right]+r[A^{⁎}AA^{†}(AB){\left(AB\right)}^{†}A,\;A^{†}(AB){\left(AB\right)}^{†}A]-2r(A^{†}(AB){\left(AB\right)}^{†}A)=2r[AA^{⁎}AB,\;AB]-2r(AB).$$

Setting all sides of (11.22), (11.23), and (11.24) equal to zero leads to the equivalences of 〈22〉–〈24〉 and 〈57〉.

By Lemma 8.6,

$$r\left[ABB^{†}A^{†},\;{\left(ABB^{†}A^{†}\right)}^{⁎}\right]=r\left[AB,\;{\left(A^{†}\right)}^{⁎}B\right]=r[AA^{⁎}AB,\;AB].$$

Hence

$$R\left(ABB^{†}A^{†}\right)=R\left({\left(ABB^{†}A^{†}\right)}^{⁎}\right)\Leftrightarrow r[AA^{⁎}AB,\;AB]=r(AB),$$

establishing the equivalence of 〈25〉 and 〈57〉.

By Lemma 8.6,

$$r\left[AB,\;{\left(A^{†}\right)}^{⁎}{\left(B^{†}\right)}^{⁎}\right]=r[AA^{⁎}AB,\;AB],r\left[AB,\;{\left(A^{†}\right)}^{⁎}{\left(B^{⁎}A^{†}A\right)}^{†}\right]=r[AA^{⁎}AB,\;AB],r\left[AB,\;A{\left(B^{⁎}A^{⁎}A\right)}^{†}\right]=r[AA^{⁎}AB,\;AB],r\left[AB,\;{\left(A^{†}\right)}^{⁎}{\left(BB^{†}A^{†}A\right)}^{†}{\left(B^{†}\right)}^{⁎}\right]=r[AA^{⁎}AB,\;AB],r\left[AB,\;A{\left(BB^{⁎}A^{⁎}A\right)}^{†}B\right]=r[AA^{⁎}AB,\;AB],r\left[{\left(B^{⁎}A^{†}A\right)}^{†},\;A^{⁎}{\left(B^{⁎}A^{⁎}\right)}^{†}\right]=r[AA^{⁎}AB,\;AB],r\left[{\left(B^{⁎}A^{⁎}A\right)}^{†},\;A^{†}{\left(B^{⁎}A^{⁎}\right)}^{†}\right]=r[AA^{⁎}AB,\;AB].$$

Hence

$$R(AB)=R\left({\left(A^{†}\right)}^{⁎}{\left(B^{†}\right)}^{⁎}\right)\Leftrightarrow r[AA^{⁎}AB,\;AB]=r(AB),R(AB)=R\left({\left(A^{†}\right)}^{⁎}{\left(B^{⁎}A^{†}A\right)}^{†}\right)\Leftrightarrow r[AA^{⁎}AB,\;AB]=r(AB),R(AB)=R\left(A{\left(B^{⁎}A^{⁎}A\right)}^{†}\right)\Leftrightarrow r[AA^{⁎}AB,\;AB]=r(AB),R(AB)=R\left({\left(A^{†}\right)}^{⁎}{\left(BB^{†}A^{†}A\right)}^{†}{\left(B^{†}\right)}^{⁎}\right)\Leftrightarrow r[AA^{⁎}AB,\;AB]=r(AB),R(AB)=R\left(A{\left(BB^{⁎}A^{⁎}A\right)}^{†}B\right)\Leftrightarrow r[AA^{⁎}AB,\;AB]=r(AB),$$

establishing the equivalences of 〈26〉–〈30〉, and 〈57〉.

Replacing *A* with ${\left(A^{†}\right)}^{⁎}$ and/or *B* with ${\left(B^{†}\right)}^{⁎}$ in 〈1〉–〈30〉 leads to 〈31〉–〈59〉, respectively. Also replacing *A* with ${\left(A^{†}\right)}^{⁎}$ in 〈57〉 leads to

(11.25) $$r[{\left(A^{†}\right)}^{⁎}A^{†}{\left(A^{†}\right)}^{⁎}B,\;{\left(A^{†}\right)}^{⁎}B]=r\left({\left(A^{†}\right)}^{⁎}B\right),$$

where by Lemma 8.6, $r[{\left(A^{†}\right)}^{⁎}A^{†}{\left(A^{†}\right)}^{⁎}B,\;{\left(A^{†}\right)}^{⁎}B]=r[AA^{⁎}AB,\;AB]$ and $r\left({\left(A^{†}\right)}^{⁎}B\right)=r(AB)$. Thus (11.25) is equivalent to 〈57〉. This fact implies that 〈30〉–〈55〉 are equivalent to 〈57〉. □

The following results can be shown by a similar approach.

Lemma 11.3 *Let* $A\in C^{m\times n}$ *and* $B\in C^{n\times p}$*. Then the following 57 statements are equivalent:*

〈1〉 $\left\{{\left(AB\right)}^{\left(1,2,4\right)}\right\}∋B^{†}{\left(ABB^{†}\right)}^{†}$*.*
〈2〉 $\left\{{\left(AB\right)}^{\left(1,2,4\right)}\right\}∋B^{⁎}{\left(ABB^{⁎}\right)}^{†}$*.*
〈3〉 $\left\{{\left(ABB^{†}\right)}^{\left(1,2,4\right)}\right\}∋B{\left(AB\right)}^{†}$*.*
〈4〉 $\left\{{\left(ABB^{⁎}\right)}^{\left(1,4\right)}\right\}∋{\left(B^{†}\right)}^{⁎}{\left(AB\right)}^{†}$*.*
〈5〉 $\left\{{\left(AB\right)}^{\left(1,2,4\right)}\right\}∋B^{†}{\left(A^{†}ABB^{†}\right)}^{†}A^{†}$*.*
〈6〉 $\left\{{\left(AB\right)}^{\left(1,2,4\right)}\right\}∋B^{⁎}{\left(A^{⁎}ABB^{⁎}\right)}^{†}A^{⁎}$*.*
〈7〉 $\left\{{\left(A^{†}ABB^{†}\right)}^{\left(1,2,4\right)}\right\}∋B{\left(AB\right)}^{†}A$*.*
〈8〉 $\left\{{\left(A^{⁎}ABB^{⁎}\right)}^{\left(1,2,4\right)}\right\}∋{\left(B^{†}\right)}^{⁎}{\left(AB\right)}^{†}{\left(A^{†}\right)}^{⁎}$*.*
〈9〉 ${\left(AB\right)}^{†}=B^{†}{\left(ABB^{†}\right)}^{†}$*.*
〈10〉 ${\left(ABB^{†}\right)}^{†}=B{\left(AB\right)}^{†}$*.*
〈11〉 ${\left(AB\right)}^{†}=B^{⁎}{\left(ABB^{⁎}\right)}^{†}$*.*
〈12〉 ${\left(ABB^{⁎}\right)}^{†}={\left(B^{†}\right)}^{⁎}{\left(AB\right)}^{†}$*.*
〈13〉 ${\left(AB\right)}^{†}ABB^{†}A^{†}=B^{†}A^{†}$*.*
〈14〉 $ABB^{†}A^{†}{\left(B^{†}A^{†}\right)}^{†}=AB$*.*
〈15〉 ${\left(AB\right)}^{†}AB=B^{†}A^{†}{\left(B^{†}A^{†}\right)}^{†}$*.*
〈16〉 $B^{†}{\left(ABB^{†}\right)}^{†}AB$ *is an orthogonal projector.*
〈17〉 $B^{⁎}{\left(ABB^{⁎}\right)}^{†}AB$ *is an orthogonal projector.*
〈18〉 $B{\left(AB\right)}^{†}(AB)B^{†}$ *is an orthogonal projector.*
〈19〉 $B^{†}{\left(A^{†}ABB^{†}\right)}^{†}B$ *is an orthogonal projector.*
〈20〉 $B^{⁎}{\left(A^{⁎}ABB^{⁎}\right)}^{†}A^{⁎}AB$ *is an orthogonal projector.*
〈21〉 ${\left(AB\right)}^{†}AB$ *and* $B^{⁎}B$ *commute.*
〈22〉 $B^{†}{\left(ABB^{†}\right)}^{†}AB$ *and* $B^{⁎}B$ *commute.*
〈23〉 $B^{⁎}{\left(ABB^{⁎}\right)}^{†}AB$ *and* $B^{⁎}B$ *commute.*
〈24〉 $B{\left(AB\right)}^{†}(AB)B^{†}$ *and* $BB^{⁎}$ *commute.*
〈25〉 $B^{†}A^{†}AB$ *is EP.*
〈26〉 $R\left({\left(AB\right)}^{†}\right)=R(B^{†}A^{†})$*.*
〈27〉 $R\left({\left(AB\right)}^{†}\right)=R\left(B^{†}{\left(ABB^{†}\right)}^{†}\right)$*.*
〈28〉 $R\left({\left(AB\right)}^{†}\right)=R\left(B^{⁎}{\left(ABB^{⁎}\right)}^{†}\right)$*.*
〈29〉 $R\left({\left(AB\right)}^{†}\right)=R\left(B^{†}{\left(A^{†}ABB^{†}\right)}^{†}A^{†}\right)$*.*
〈30〉 $R\left({\left(AB\right)}^{†}\right)=R\left(B^{⁎}{\left(A^{⁎}ABB^{⁎}\right)}^{†}A^{⁎}\right)$*.*
〈31〉 $\left\{{\left(A{\left(B^{†}\right)}^{⁎}\right)}^{\left(1,2,4\right)}\right\}∋B^{⁎}{\left(ABB^{†}\right)}^{†}$*.*
〈32〉 $\left\{{\left[A(B^{†})\right]}^{\left(1,2,4\right)}\right\}∋B^{†}{\left(A{\left(BB^{⁎}\right)}^{†}\right)}^{†}$*.*
〈33〉 $\left\{{\left(ABB^{†}\right)}^{\left(1,2,4\right)}\right\}∋B^{†}{\left(A{\left(B^{†}\right)}^{⁎}\right)}^{†}$*.*
〈34〉 $\left\{{\left(A{\left(BB^{⁎}\right)}^{†}\right)}^{\left(1,4\right)}\right\}∋B{\left(A{\left(B^{†}\right)}^{⁎}\right)}^{†}$*.*
〈35〉 $\left\{{\left(A{\left(B^{†}\right)}^{⁎}\right)}^{\left(1,2,4\right)}\right\}∋B^{⁎}{\left(A^{†}ABB^{†}\right)}^{†}A^{†}$*.*
〈36〉 $\left\{{\left(A{\left(B^{†}\right)}^{⁎}\right)}^{\left(1,2,4\right)}\right\}∋B^{†}{\left(A^{⁎}A{\left(BB^{⁎}\right)}^{†}\right)}^{†}A^{⁎}$*.*
〈37〉 ${\left\{{\left(A^{†}ABB^{†}\right)}^{\left(1,2,4\right)}\right\}∋B^{†})}^{⁎}{\left(A{\left(B^{†}\right)}^{⁎}\right)}^{†}A$*.*
〈38〉 $\left\{{\left(A^{⁎}A{\left(BB^{⁎}\right)}^{†}\right)}^{\left(1,2,4\right)}\right\}∋B{\left(A{\left(B^{†}\right)}^{⁎}\right)}^{†}{\left(A^{†}\right)}^{⁎}$*.*
〈39〉 ${\left(A{\left(B^{†}\right)}^{⁎}\right)}^{†}=B^{⁎}{\left(ABB^{†}\right)}^{†}$*.*
〈40〉 $\left(A{\left(B^{†}\right)}^{⁎}\right)=B^{†}{\left(A{\left(BB^{⁎}\right)}^{†}\right)}^{†}$*.*
〈41〉 ${\left(ABB^{†}\right)}^{†}={\left(B^{†}\right)}^{⁎}{\left(A{\left(B^{†}\right)}^{⁎}\right)}^{†}$*.*
〈42〉 ${\left(A{\left(B^{†}\right)}^{⁎}\right)}^{†}=B{\left(A{\left(BB^{⁎}\right)}^{†}\right)}^{†}$*.*
〈43〉 $B^{†}A^{†}{\left(BB^{†}A^{†}\right)}^{†}B$ *is an orthogonal projector.*
〈44〉 $B^{†}A^{†}{\left({\left(BB^{⁎}\right)}^{†}A^{†}\right)}^{†}{\left(B^{†}\right)}^{⁎}$ *is orthogonal projector.*
〈45〉 $B(B^{†}A^{†}){\left(B^{†}A^{†}\right)}^{†}B^{†}$ *is orthogonal projector.*
〈46〉 $B^{†}{\left(BB^{†}A^{†}A\right)}^{†}B$ *is an orthogonal projector.*
〈47〉 $B^{†}{\left({\left(A^{⁎}A\right)}^{†}{\left(BB^{⁎}\right)}^{†}\right)}^{†}{\left(A^{⁎}A\right)}^{†}{\left(B^{†}\right)}^{⁎}$ *is orthogonal projector.*
〈48〉 $(B^{†}A^{†}){\left(B^{†}A^{†}\right)}^{†}$ *and* ${\left(B^{⁎}B\right)}^{†}$ *commute.*
〈49〉 $B^{†}A^{†}{\left(BB^{†}A^{†}\right)}^{†}B$ *and* ${\left(B^{⁎}B\right)}^{†}$ *commute.*
〈50〉 $B^{†}A^{†}{\left({\left(BB^{⁎}\right)}^{†}A^{†}\right)}^{†}{\left(B^{†}\right)}^{⁎}$ *and* ${\left(B^{⁎}B\right)}^{†}$ *commute.*
〈51〉 $B(B^{†}A^{†}){\left(B^{†}A^{†}\right)}^{†}B^{†}$ *and* ${\left(BB^{⁎}\right)}^{†}$ *commute.*
〈52〉 $R\left({\left({\left(A^{†}\right)}^{⁎}{\left(B^{†}\right)}^{⁎}\right)}^{†}\right)=R\left(B^{⁎}{\left({\left(A^{†}\right)}^{⁎}BB^{†}\right)}^{†}\right)$*.*
〈53〉 $R\left({\left({\left(A^{†}\right)}^{⁎}{\left(B^{†}\right)}^{⁎}\right)}^{†}\right)=R\left(B^{†}{\left({\left(A^{†}\right)}^{⁎}{\left(BB^{⁎}\right)}^{†}\right)}^{†}\right)$*.*
〈54〉 $R\left({\left({\left(A^{†}\right)}^{⁎}{\left(B^{†}\right)}^{⁎}\right)}^{†}\right)=R\left(B^{⁎}{\left(A^{†}ABB^{†}\right)}^{†}A^{⁎}\right)$*.*
〈55〉 $R\left({\left({\left(A^{†}\right)}^{⁎}{\left(B^{†}\right)}^{⁎}\right)}^{†}\right)=R\left(B^{†}{\left({\left(A^{⁎}A\right)}^{†}{\left(BB^{⁎}\right)}^{†}\right)}^{†}\right)$*.*
〈56〉 $R\left(B^{⁎}B{\left(AB\right)}^{⁎}\right)=R\left({\left(AB\right)}^{⁎}\right)$*.*
〈57〉 $r[B^{⁎}B{\left(AB\right)}^{⁎},\;{\left(AB\right)}^{⁎}]=r(AB)$*.*

Combining the above two lemmas, we obtain the following principle results in this section.

Theorem 11.4 *Let* $A\in C^{m\times n}$ *and* $B\in C^{n\times p}$*. Then the following 132 statements are equivalent:*

〈1〉 ${\left(AB\right)}^{†}=B^{†}{\left(A^{†}ABB^{†}\right)}^{†}A^{†}$*.*
〈2〉 ${\left(A^{†}ABB^{†}\right)}^{†}=B{\left(AB\right)}^{†}A$*.*
〈3〉 $\left\{{\left(AB\right)}^{\left(1,2,3\right)}\right\}∋{\left(A^{†}AB\right)}^{†}A^{†}$ *and* $\left\{{\left(AB\right)}^{\left(1,2,4\right)}\right\}∋B^{†}{\left(ABB^{†}\right)}^{†}$*.*
〈4〉 $\left\{{\left(AB\right)}^{\left(1,2,3\right)}\right\}∋{\left(A^{⁎}AB\right)}^{†}A^{⁎}$*, and* $\left\{{\left(AB\right)}^{\left(1,2,4\right)}\right\}∋B^{⁎}{\left(ABB^{⁎}\right)}^{†}$*.*
〈5〉 $\left\{{\left(A^{†}AB\right)}^{\left(1,2,3\right)}\right\}∋{\left(AB\right)}^{†}A$ *and* $\left\{{\left(ABB^{†}\right)}^{\left(1,2,4\right)}\right\}∋B{\left(AB\right)}^{†}$*.*
〈6〉 $\left\{{\left(A^{⁎}AB\right)}^{\left(1,2,3\right)}\right\}∋{\left(AB\right)}^{†}{\left(A^{†}\right)}^{⁎}$ *and* $\left\{{\left(ABB^{⁎}\right)}^{\left(1,4\right)}\right\}∋{\left(B^{†}\right)}^{⁎}{\left(AB\right)}^{†}$*.*
〈7〉 ${\left(AB\right)}^{†}={\left(A^{†}AB\right)}^{†}A^{†}$ *and* ${\left(AB\right)}^{†}=B^{†}{\left(ABB^{†}\right)}^{†}$*.*
〈8〉 ${\left(A^{†}AB\right)}^{†}={\left(AB\right)}^{†}A$ *and* ${\left(ABB^{†}\right)}^{†}=B{\left(AB\right)}^{†}$*.*
〈9〉 $AB{\left(AB\right)}^{†}A=AB{\left(A^{†}AB\right)}^{†}$ *and* $B{\left(AB\right)}^{†}AB={\left(ABB^{†}\right)}^{†}AB$*.*
〈10〉 $AB{\left(AB\right)}^{†}=A{\left(A^{†}ABB^{†}\right)}^{†}A^{†}$ *and* ${\left(AB\right)}^{†}AB=B^{†}{\left(A^{†}ABB^{†}\right)}^{†}B$*.*
〈11〉 $AB{\left(AB\right)}^{†}A=A{\left(A^{†}ABB^{†}\right)}^{†}$ *and* $B{\left(AB\right)}^{†}AB={\left(A^{†}ABB^{†}\right)}^{†}B$*.*
〈12〉 ${\left(ABB^{†}\right)}^{†}={\left(A^{†}ABB^{†}\right)}^{†}A^{†}$ *and* ${\left(A^{†}AB\right)}^{†}=B^{†}{\left(A^{†}ABB^{†}\right)}^{†}$*.*
〈13〉 ${\left(A^{†}ABB^{†}\right)}^{†}={\left(ABB^{†}\right)}^{†}A$ *and* ${\left(A^{†}ABB^{†}\right)}^{†}=B{\left(A^{†}AB\right)}^{†}$*.*
〈14〉 ${\left(A^{†}AB\right)}^{†}A^{†}=B^{†}{\left(ABB^{†}\right)}^{†}$*.*
〈15〉 ${\left(ABB^{†}\right)}^{†}A=B{\left(A^{†}AB\right)}^{†}$*.*
〈16〉 ${\left(B^{†}A^{†}\right)}^{†}=A{\left(B^{†}A^{†}A\right)}^{†}$ *and* ${\left(B^{†}A^{†}\right)}^{†}={\left(BB^{†}A^{†}\right)}^{†}B$*.*
〈17〉 ${\left(B^{†}A^{†}A\right)}^{†}=A^{†}{\left(B^{†}A^{†}\right)}^{†}$ *and* ${\left(BB^{†}A^{†}\right)}^{†}={\left(B^{†}A^{†}\right)}^{†}B^{†}$*.*
〈18〉 $B^{†}A^{†}{\left(B^{†}A^{†}\right)}^{†}=B^{†}{\left(B^{†}A^{†}A\right)}^{†}$ *and* ${\left(B^{†}A^{†}\right)}^{†}B^{†}A^{†}={\left(BB^{†}A^{†}\right)}^{†}A^{†}$*.*
〈19〉 ${\left(B^{†}A^{†}\right)}^{†}=A{\left(BB^{†}A^{†}A\right)}^{†}B$*.*
〈20〉 ${\left(BB^{†}A^{†}A\right)}^{†}=A^{†}{\left(B^{†}A^{†}\right)}^{†}B^{†}$*.*
〈21〉 $B^{†}A^{†}{\left(B^{†}A^{†}\right)}^{†}=B^{†}{\left(BB^{†}A^{†}A\right)}^{†}B$ *and* ${\left(B^{†}A^{†}\right)}^{†}B^{†}A^{†}=A{\left(BB^{†}A^{†}A\right)}^{†}A^{†}$*.*
〈22〉 $B^{†}A^{†}{\left(B^{†}A^{†}\right)}^{†}B^{†}=B^{†}{\left(BB^{†}A^{†}A\right)}^{†}$ *and* $A^{†}{\left(B^{†}A^{†}\right)}^{†}B^{†}A^{†}={\left(BB^{†}A^{†}A\right)}^{†}A^{†}$*.*
〈23〉 $A{\left(B^{†}A^{†}A\right)}^{†}={\left(BB^{†}A^{†}\right)}^{†}B$*.*
〈24〉 $A^{†}{\left(BB^{†}A^{†}\right)}^{†}={\left(B^{†}A^{†}A\right)}^{†}B^{†}$*.*
〈25〉 ${\left(B^{†}A^{†}A\right)}^{†}={\left(BB^{†}A^{†}A\right)}^{†}B$ *and* ${\left(BB^{†}A^{†}\right)}^{†}=A{\left(BB^{†}A^{†}A\right)}^{†}$*.*
〈26〉 ${\left(BB^{†}A^{†}A\right)}^{†}={\left(B^{†}A^{†}A\right)}^{†}B^{†}$ *and* ${\left(BB^{†}A^{†}A\right)}^{†}=A^{†}{\left(BB^{†}A^{†}\right)}^{†}$*.*
〈27〉 $B^{†}A^{†}AB{\left(AB\right)}^{†}=B^{†}A^{†}$ *and* ${\left(AB\right)}^{†}ABB^{†}A^{†}=B^{†}A^{†}$*.*
〈28〉 ${\left(B^{†}A^{†}\right)}^{†}B^{†}A^{†}AB=AB$ *and* $ABB^{†}A^{†}{\left(B^{†}A^{†}\right)}^{†}=AB$*.*
〈29〉 $AB{\left(AB\right)}^{†}={\left(B^{†}A^{†}\right)}^{†}B^{†}A^{†}$ *and* ${\left(AB\right)}^{†}AB=B^{†}A^{†}{\left(B^{†}A^{†}\right)}^{†}$*.*
〈30〉 ${\left({\left(A^{†}\right)}^{⁎}B\right)}^{†}={\left(A^{†}AB\right)}^{†}A^{⁎}$ *and* ${\left(A{\left(B^{†}\right)}^{⁎}\right)}^{†}=B^{⁎}{\left(ABB^{†}\right)}^{†}$*.*
〈31〉 ${\left(A^{†}AB\right)}^{†}={\left({\left(A^{†}\right)}^{⁎}B\right)}^{†}{\left(A^{†}\right)}^{⁎}$ *and* ${\left(ABB^{†}\right)}^{†}={\left(B^{†}\right)}^{⁎}{\left(A{\left(B^{†}\right)}^{⁎}\right)}^{†}$*.*
〈32〉 $A{\left(B^{†}\right)}^{⁎}{\left(A{\left(B^{†}\right)}^{⁎}\right)}^{†}=A{\left(ABB^{†}\right)}^{†}$ *and* ${\left({\left(A^{†}\right)}^{⁎}B\right)}^{†}{\left(A^{†}\right)}^{⁎}B={\left(A^{†}AB\right)}^{†}B$*.*
〈33〉 ${\left({\left(A^{†}\right)}^{⁎}BB^{†}\right)}^{†}={\left(A^{†}ABB^{†}\right)}^{†}A^{⁎}$ *and* ${\left(A^{†}A{\left(B^{†}\right)}^{⁎}\right)}^{†}=B^{⁎}{\left(A^{†}ABB^{†}\right)}^{†}$*.*
〈34〉 ${\left(A^{†}ABB^{†}\right)}^{†}={\left({\left(A^{†}\right)}^{⁎}BB^{†}\right)}^{†}{\left(A^{†}\right)}^{⁎}$ *and* ${\left(A^{†}ABB^{†}\right)}^{†}={\left(B^{†}\right)}^{⁎}{\left(A^{†}A{\left(B^{†}\right)}^{⁎}\right)}^{†}$*.*
〈35〉 ${\left({\left(A^{†}\right)}^{⁎}BB^{†}\right)}^{†}{\left(A^{†}\right)}^{⁎}={\left(B^{†}\right)}^{⁎}{\left(A^{†}A{\left(B^{†}\right)}^{⁎}\right)}^{†}$*.*
〈36〉 $B^{⁎}{\left({\left(A^{†}\right)}^{⁎}BB^{†}\right)}^{†}={\left(A^{†}A{\left(B^{†}\right)}^{⁎}\right)}^{†}A^{⁎}$*.*
〈37〉 ${\left(AB\right)}^{†}=B^{⁎}{\left(A^{⁎}ABB^{⁎}\right)}^{†}A^{⁎}$*.*
〈38〉 ${\left(A^{⁎}ABB^{⁎}\right)}^{†}={\left(B^{†}\right)}^{⁎}{\left(AB\right)}^{†}{\left(A^{†}\right)}^{⁎}$*.*
〈39〉 ${\left(AB\right)}^{†}={\left(A^{⁎}AB\right)}^{†}A^{⁎}$ *and* ${\left(AB\right)}^{†}=B^{⁎}{\left(ABB^{⁎}\right)}^{†}$*.*
〈40〉 ${\left(A^{⁎}AB\right)}^{†}={\left(AB\right)}^{†}{\left(A^{†}\right)}^{⁎}$ *and* ${\left(ABB^{⁎}\right)}^{†}={\left(A^{†}\right)}^{⁎}{\left(AB\right)}^{†}$*.*
〈41〉 ${\left({\left(A^{†}\right)}^{⁎}B\right)}^{†}={\left({\left(A^{⁎}A\right)}^{†}B\right)}^{†}A^{†}$ *and* ${\left(A{\left(B^{†}\right)}^{⁎}\right)}^{†}=B^{†}{\left(A{\left(BB^{⁎}\right)}^{†}\right)}^{†}$*.*
〈42〉 ${\left({\left(A^{⁎}A\right)}^{†}B\right)}^{†}={\left({\left(A^{†}\right)}^{⁎}B\right)}^{†}A$ *and* ${\left(A{\left(BB^{⁎}\right)}^{†}\right)}^{†}=B{\left(A{\left(B^{†}\right)}^{⁎}\right)}^{†}$*.*
〈43〉 ${\left({\left(A^{†}\right)}^{⁎}{\left(B^{†}\right)}^{⁎}\right)}^{†}={\left({\left(A^{⁎}A\right)}^{†}B\right)}^{†}A^{†}$ *and* ${\left({\left(A^{†}\right)}^{⁎}{\left(B^{†}\right)}^{⁎}\right)}^{†}=B^{†}{\left({\left(A^{†}\right)}^{⁎}{\left(BB^{⁎}\right)}^{†}\right)}^{†}$*.*
〈44〉 ${\left({\left(A^{⁎}A\right)}^{†}B\right)}^{†}={\left({\left(A^{†}\right)}^{⁎}{\left(B^{†}\right)}^{⁎}\right)}^{†}A$ *and* ${\left({\left(A^{†}\right)}^{⁎}{\left(BB^{⁎}\right)}^{†}\right)}^{†}=B{\left({\left(A^{†}\right)}^{⁎}{\left(B^{†}\right)}^{⁎}\right)}^{†}$*.*
〈45〉 ${\left({\left(A^{†}\right)}^{⁎}{\left(B^{†}\right)}^{⁎}\right)}^{†}=B^{†}{\left({\left(A^{⁎}A\right)}^{†}{\left(BB^{⁎}\right)}^{†}\right)}^{†}A^{†}$*.*
〈46〉 ${\left({\left(A^{⁎}A\right)}^{†}{\left(BB^{⁎}\right)}^{†}\right)}^{†}=B{\left({\left(A^{†}\right)}^{⁎}{\left(B^{†}\right)}^{⁎}\right)}^{†}A$*.*
〈47〉 ${\left(A^{⁎}AB\right)}^{†}=B^{⁎}{\left(A^{⁎}ABB^{⁎}\right)}^{†}$ *and* ${\left(ABB^{⁎}\right)}^{†}={\left(A^{⁎}ABB^{⁎}\right)}^{†}A^{⁎}$*.*
〈48〉 ${\left(A^{⁎}ABB^{⁎}\right)}^{†}={\left(ABB^{⁎}\right)}^{†}{\left(A^{†}\right)}^{⁎}$ *and* ${\left(A^{⁎}ABB^{⁎}\right)}^{†}={\left(B^{†}\right)}^{⁎}{\left(A^{⁎}AB\right)}^{†}$*.*
〈49〉 ${\left(A^{⁎}AB\right)}^{†}A^{⁎}=B^{⁎}{\left(ABB^{⁎}\right)}^{†}$*.*
〈50〉 ${\left(ABB^{⁎}\right)}^{†}{\left(A^{†}\right)}^{⁎}={\left(B^{†}\right)}^{⁎}{\left(A^{⁎}AB\right)}^{†}$*.*
〈51〉 ${\left({\left(A^{⁎}A\right)}^{†}B\right)}^{†}=B^{⁎}{\left({\left(A^{⁎}A\right)}^{†}BB^{⁎}\right)}^{†}$ *and* ${\left(A{\left(BB^{⁎}\right)}^{†}\right)}^{†}={\left(A^{⁎}A{\left(BB^{⁎}\right)}^{†}\right)}^{†}A^{⁎}$*.*
〈52〉 ${\left({\left(A^{⁎}A\right)}^{†}BB^{⁎}\right)}^{†}={\left(B^{†}\right)}^{⁎}{\left({\left(A^{⁎}A\right)}^{†}B\right)}^{†}$ *and* ${\left(A^{⁎}A{\left(BB^{⁎}\right)}^{†}\right)}^{†}={\left(A{\left(BB^{⁎}\right)}^{†}\right)}^{†}{\left(A^{†}\right)}^{⁎}$*.*
〈53〉 ${\left({\left(A^{†}\right)}^{⁎}{\left(BB^{⁎}\right)}^{†}\right)}^{†}A=B{\left({\left(A^{⁎}A\right)}^{†}{\left(B^{†}\right)}^{⁎}\right)}^{†}$*.*
〈54〉 ${\left({\left(A^{⁎}A\right)}^{†}{\left(B^{†}\right)}^{⁎}\right)}^{†}A^{†}=B^{†}{\left({\left(A^{†}\right)}^{⁎}{\left(BB^{⁎}\right)}^{†}\right)}^{†}$*.*
〈55〉 ${\left(A^{⁎}AB\right)}^{†}={\left(AA^{⁎}AB\right)}^{†}A$ *and* ${\left(ABB^{⁎}\right)}^{†}=B{\left(ABB^{⁎}B\right)}^{†}$*.*
〈56〉 ${\left(AA^{⁎}AB\right)}^{†}={\left(A^{⁎}AB\right)}^{†}A^{†}$ *and* ${\left(ABB^{⁎}B\right)}^{†}=B^{†}{\left(ABB^{⁎}\right)}^{†}$*.*
〈57〉 ${\left({\left(A^{⁎}A\right)}^{†}B\right)}^{†}={\left({\left(A^{⁎}AA^{⁎}\right)}^{†}B\right)}^{†}{\left(A^{†}\right)}^{⁎}$ *and* ${\left(A{\left(BB^{⁎}\right)}^{†}\right)}^{†}={\left(B^{†}\right)}^{⁎}{\left(A{\left(B^{⁎}BB^{⁎}\right)}^{†}\right)}^{†}$*.*
〈58〉 ${\left({\left(A^{⁎}A\right)}^{†}B\right)}^{†}A^{⁎}={\left({\left(A^{⁎}AA^{⁎}\right)}^{†}B\right)}^{†}$ *and* $B^{⁎}{\left(A{\left(BB^{⁎}\right)}^{†}\right)}^{†}={\left(A{\left(B^{⁎}BB^{⁎}\right)}^{†}\right)}^{†}$*.*
〈59〉 ${\left({\left(A^{⁎}A\right)}^{†}{\left(B^{†}\right)}^{⁎}\right)}^{†}={\left({\left(A^{⁎}AA^{⁎}\right)}^{†}{\left(B^{†}\right)}^{⁎}\right)}^{†}{\left(A^{†}\right)}^{⁎}$ *and* ${\left({\left(A^{†}\right)}^{⁎}{\left(BB^{⁎}\right)}^{†}\right)}^{†}={\left(B^{†}\right)}^{⁎}{\left({\left(A^{†}\right)}^{⁎}{\left(B^{⁎}BB^{⁎}\right)}^{†}\right)}^{†}$*.*
〈60〉 ${\left({\left(A^{⁎}AA^{⁎}\right)}^{†}{\left(B^{†}\right)}^{⁎}\right)}^{†}={\left({\left(A^{⁎}A\right)}^{†}{\left(B^{†}\right)}^{⁎}\right)}^{†}A^{⁎}$ *and* ${\left({\left(A^{†}\right)}^{⁎}{\left(B^{⁎}BB^{⁎}\right)}^{†}\right)}^{†}=B^{⁎}{\left({\left(A^{†}\right)}^{⁎}{\left(BB^{⁎}\right)}^{†}\right)}^{†}$*.*
〈61〉 ${\left(A^{⁎}ABB^{⁎}\right)}^{†}=B{\left(A^{⁎}ABB^{⁎}B\right)}^{†}$ *and* ${\left(A^{⁎}ABB^{⁎}\right)}^{†}={\left(AA^{⁎}ABB^{⁎}\right)}^{†}A$*.*
〈62〉 ${\left(A^{⁎}ABB^{⁎}B\right)}^{†}=B^{†}{\left(A^{⁎}ABB^{⁎}\right)}^{†}$ *and* ${\left(AA^{⁎}ABB^{⁎}\right)}^{†}={\left(A^{⁎}ABB^{⁎}\right)}^{†}A^{†}$*.*
〈63〉 ${\left({\left(A^{⁎}A\right)}^{†}BB^{⁎}\right)}^{†}=B{\left({\left(A^{⁎}A\right)}^{†}BB^{⁎}B\right)}^{†}$ *and* ${\left({\left(A^{⁎}A\right)}^{†}BB^{⁎}\right)}^{†}={\left({\left(A^{⁎}AA^{⁎}\right)}^{†}BB^{⁎}\right)}^{†}{\left(A^{†}\right)}^{⁎}$*.*
〈64〉 ${\left({\left(A^{⁎}A\right)}^{†}BB^{⁎}B\right)}^{†}=B^{†}{\left({\left(A^{⁎}A\right)}^{†}BB^{⁎}\right)}^{†}$ *and* ${\left({\left(A^{⁎}AA^{⁎}\right)}^{†}BB^{⁎}\right)}^{†}={\left({\left(A^{⁎}A\right)}^{†}BB^{⁎}\right)}^{†}A^{⁎}$*.*
〈65〉 ${\left(A^{⁎}A{\left(BB^{⁎}\right)}^{†}\right)}^{†}={\left(B^{†}\right)}^{⁎}{\left(A^{⁎}A{\left(B^{⁎}BB^{⁎}\right)}^{†}\right)}^{†}$ *and* ${\left(A^{⁎}A{\left(BB^{⁎}\right)}^{†}\right)}^{†}={\left(AA^{⁎}A{\left(BB^{⁎}\right)}^{†}\right)}^{†}A$*.*
〈66〉 ${\left(A^{⁎}A{\left(B^{⁎}BB^{⁎}\right)}^{†}\right)}^{†}=B^{⁎}{\left(A^{⁎}A{\left(BB^{⁎}\right)}^{†}\right)}^{†}$ *and* ${\left(AA^{⁎}A{\left(BB^{⁎}\right)}^{†}\right)}^{†}={\left(A^{⁎}A{\left(BB^{⁎}\right)}^{†}\right)}^{†}A^{†}$*.*
〈67〉 ${\left({\left(A^{⁎}A\right)}^{†}{\left(BB^{⁎}\right)}^{†}\right)}^{†}={\left(B^{†}\right)}^{⁎}{\left({\left(A^{⁎}A\right)}^{†}{\left(B^{⁎}BB^{⁎}\right)}^{†}\right)}^{†}$ *and* ${\left({\left(A^{⁎}A\right)}^{†}{\left(BB^{⁎}\right)}^{†}\right)}^{†}={\left({\left(A^{⁎}AA^{⁎}\right)}^{†}{\left(BB^{⁎}\right)}^{†}\right)}^{†}{\left(A^{†}\right)}^{⁎}$*.*
〈68〉 ${\left({\left(A^{⁎}A\right)}^{†}{\left(B^{⁎}BB^{⁎}\right)}^{†}\right)}^{†}=B^{⁎}{\left({\left(A^{⁎}A\right)}^{†}{\left(BB^{⁎}\right)}^{†}\right)}^{†}$ *and* ${\left({\left(A^{⁎}AA^{⁎}\right)}^{†}{\left(BB^{⁎}\right)}^{†}\right)}^{†}={\left({\left(A^{⁎}A\right)}^{†}{\left(BB^{⁎}\right)}^{†}\right)}^{†}A^{⁎}$*.*
〈69〉 $B{\left(A^{⁎}ABB^{⁎}B\right)}^{†}={\left(AA^{⁎}ABB^{⁎}\right)}^{†}A$*.*
〈70〉 ${\left(A^{⁎}ABB^{⁎}B\right)}^{†}A^{†}=B^{†}{\left(AA^{⁎}ABB^{⁎}\right)}^{†}$*.*
〈71〉 $B{\left({\left(A^{⁎}A\right)}^{†}BB^{⁎}B\right)}^{†}={\left({\left(A^{⁎}AA^{⁎}\right)}^{†}BB^{⁎}\right)}^{†}{\left(A^{†}\right)}^{⁎}$*.*
〈72〉 ${\left({\left(A^{⁎}A\right)}^{†}BB^{⁎}B\right)}^{†}A^{⁎}=B^{†}{\left({\left(A^{⁎}AA^{⁎}\right)}^{†}BB^{⁎}\right)}^{†}$*.*
〈73〉 ${\left(B^{†}\right)}^{⁎}{\left(A^{⁎}A{\left(B^{⁎}BB^{⁎}\right)}^{†}\right)}^{†}={\left(AA^{⁎}A{\left(BB^{⁎}\right)}^{†}\right)}^{†}A$*.*
〈74〉 ${\left(A^{⁎}A{\left(B^{⁎}BB^{⁎}\right)}^{†}\right)}^{†}A^{†}=B^{⁎}{\left(AA^{⁎}A{\left(BB^{⁎}\right)}^{†}\right)}^{†}$*.*
〈75〉 ${\left({\left(BB^{⁎}B\right)}^{†}{\left(A^{⁎}A\right)}^{†}\right)}^{†}B^{†}=A^{†}{\left({\left(BB^{⁎}\right)}^{†}{\left(AA^{⁎}A\right)}^{†}\right)}^{†}$*.*
〈76〉 $A{\left({\left(BB^{⁎}B\right)}^{†}{\left(A^{⁎}A\right)}^{†}\right)}^{†}={\left({\left(BB^{⁎}\right)}^{†}{\left(AA^{⁎}A\right)}^{†}\right)}^{†}B$*.*
〈77〉 ${\left(A^{⁎}ABB^{⁎}\right)}^{†}=B{\left(AA^{⁎}ABB^{⁎}B\right)}^{†}A$*.*
〈78〉 ${\left(AA^{⁎}ABB^{⁎}B\right)}^{†}=B^{†}{\left(A^{⁎}ABB^{⁎}\right)}^{†}A^{†}$*.*
〈79〉 ${\left({\left(A^{⁎}A\right)}^{†}BB^{⁎}\right)}^{†}=B{\left({\left(A^{⁎}AA^{⁎}\right)}^{†}BB^{⁎}B\right)}^{†}{\left(A^{†}\right)}^{⁎}$*.*
〈80〉 ${\left({\left(A^{⁎}AA^{⁎}\right)}^{†}BB^{⁎}B\right)}^{†}=B^{†}{\left({\left(A^{⁎}A\right)}^{†}BB^{⁎}\right)}^{†}A^{⁎}$*.*
〈81〉 ${\left(A^{⁎}A{\left(BB^{⁎}\right)}^{†}\right)}^{†}={\left(B^{†}\right)}^{⁎}{\left(AA^{⁎}A{\left(B^{⁎}BB^{⁎}\right)}^{†}\right)}^{†}A$*.*
〈82〉 ${\left(AA^{⁎}A{\left(B^{⁎}BB^{⁎}\right)}^{†}\right)}^{†}=B^{⁎}{\left(A^{⁎}A{\left(BB^{⁎}\right)}^{†}\right)}^{†}{\left(A^{†}\right)}^{⁎}$*.*
〈83〉 ${\left({\left(A^{⁎}A\right)}^{†}{\left(BB^{⁎}\right)}^{†}\right)}^{†}={\left(B^{†}\right)}^{⁎}{\left({\left(A^{⁎}AA^{⁎}\right)}^{†}{\left(B^{⁎}BB^{⁎}\right)}^{†}\right)}^{†}{\left(A^{†}\right)}^{⁎}$*.*
〈84〉 ${\left({\left(A^{⁎}AA^{⁎}\right)}^{†}{\left(B^{⁎}BB^{⁎}\right)}^{†}\right)}^{†}=B^{⁎}{\left({\left(A^{⁎}A\right)}^{†}{\left(BB^{⁎}\right)}^{†}\right)}^{†}A^{⁎}$*.*
〈85〉 ${\left(AA^{⁎}ABB^{⁎}B\right)}^{†}={\left({\left(A^{⁎}A\right)}^{2}BB^{⁎}B\right)}^{†}A^{⁎}$ *and* ${\left(AA^{⁎}ABB^{⁎}B\right)}^{†}=B^{⁎}{\left(AA^{⁎}A{\left(BB^{⁎}\right)}^{2}\right)}^{†}$*.*
〈86〉 ${\left({\left(A^{⁎}A\right)}^{2}BB^{⁎}B\right)}^{†}={\left(AA^{⁎}ABB^{⁎}B\right)}^{†}{\left(A^{†}\right)}^{⁎}$ *and* ${\left(AA^{⁎}A{\left(BB^{⁎}\right)}^{2}\right)}^{†}={\left(B^{†}\right)}^{⁎}{\left(AA^{⁎}ABB^{⁎}B\right)}^{†}$*.*
〈87〉 ${\left({\left(A^{⁎}A\right)}^{2}BB^{⁎}B\right)}^{†}A^{⁎}=B^{⁎}{\left(AA^{⁎}A{\left(BB^{⁎}\right)}^{2}\right)}^{†}$*.*
〈88〉 ${\left(AA^{⁎}ABB^{⁎}B\right)}^{†}{\left(A^{†}\right)}^{⁎}={\left(B^{†}\right)}^{⁎}{\left(AA^{⁎}ABB^{⁎}B\right)}^{†}$*.*
〈89〉 ${\left(AA^{⁎}ABB^{⁎}B\right)}^{†}=B^{⁎}{\left({\left(A^{⁎}A\right)}^{2}{\left(BB^{⁎}\right)}^{2}\right)}^{†}A^{⁎}$*.*
〈90〉 ${\left({\left(A^{⁎}A\right)}^{2}{\left(BB^{⁎}\right)}^{2}\right)}^{†}={\left(B^{†}\right)}^{⁎}{\left(AA^{⁎}ABB^{⁎}B\right)}^{†}{\left(A^{†}\right)}^{⁎}$*.*
〈91〉 ${\left(A^{⁎}AB\right)}^{†}=B^{†}{\left(A^{⁎}ABB^{†}\right)}^{†}$ *and* ${\left(ABB^{⁎}\right)}^{†}={\left(A^{†}ABB^{⁎}\right)}^{†}A^{†}$*.*
〈92〉 ${\left(A^{⁎}ABB^{†}\right)}^{†}=B{\left(A^{⁎}AB\right)}^{†}$ *and* ${\left(A^{†}ABB^{⁎}\right)}^{†}={\left(ABB^{⁎}\right)}^{†}A$*.*
〈93〉 ${\left(B^{†}{\left(A^{⁎}A\right)}^{†}\right)}^{†}={\left({\left(BB^{⁎}\right)}^{†}{\left(A^{⁎}A\right)}^{†}\right)}^{†}{\left(B^{†}\right)}^{⁎}$ *and* ${\left({\left(BB^{⁎}\right)}^{†}A^{†}\right)}^{†}={\left(A^{†}\right)}^{⁎}{\left({\left(BB^{⁎}\right)}^{†}{\left(A^{⁎}A\right)}^{†}\right)}^{†}$*.*
〈94〉 ${\left({\left(A^{⁎}A\right)}^{†}{\left(B^{†}\right)}^{⁎}\right)}^{†}=B^{†}{\left({\left(A^{⁎}A\right)}^{†}{\left(BB^{⁎}\right)}^{†}\right)}^{†}$ *and* ${\left({\left(A^{†}\right)}^{⁎}{\left(BB^{⁎}\right)}^{†}\right)}^{†}={\left({\left(A^{⁎}A\right)}^{†}{\left(BB^{⁎}\right)}^{†}\right)}^{†}A^{†}$*.*
〈95〉 ${\left({\left(A^{⁎}A\right)}^{†}{\left(BB^{⁎}\right)}^{†}\right)}^{†}=B{\left({\left(A^{⁎}A\right)}^{†}{\left(B^{†}\right)}^{⁎}\right)}^{†}$ *and* ${\left({\left(A^{⁎}A\right)}^{†}{\left(BB^{⁎}\right)}^{†}\right)}^{†}={\left({\left(A^{†}\right)}^{⁎}{\left(BB^{⁎}\right)}^{†}\right)}^{†}A$*.*
〈96〉 ${\left(AB\right)}^{†}=B^{†}A^{†}-B^{†}{\left(E_{B}F_{A}\right)}^{†}A^{†}$*.*
〈97〉 ${\left({\left(A^{†}\right)}^{⁎}B\right)}^{†}=B^{†}A^{⁎}-B^{†}{\left(E_{B}F_{A}\right)}^{†}A^{⁎}$*.*
〈98〉 ${\left(A{\left(B^{†}\right)}^{⁎}\right)}^{†}=B^{⁎}A^{†}-B^{⁎}{\left(E_{B}F_{A}\right)}^{†}A^{†}$*.*
〈99〉 ${\left(B^{†}A^{†}\right)}^{†}=AB-A{\left(F_{A}E_{B}\right)}^{†}B$*.*
〈100〉 $AB{\left(AB\right)}^{†}=ABB^{†}A^{†}-ABB^{†}{\left(E_{B}F_{A}\right)}^{†}A^{†}$ *and* ${\left(AB\right)}^{†}AB=B^{†}A^{†}AB-B^{†}{\left(E_{B}F_{A}\right)}^{†}A^{†}AB$*.*
〈101〉 ${\left(A^{†}\right)}^{⁎}B{\left({\left(A^{†}\right)}^{⁎}B\right)}^{†}={\left(A^{†}\right)}^{⁎}BB^{†}A^{⁎}-{\left(A^{†}\right)}^{⁎}BB^{†}{\left(E_{B}F_{A}\right)}^{†}A^{⁎}$ *and* ${\left(A{\left(B^{†}\right)}^{⁎}\right)}^{†}A{\left(B^{†}\right)}^{⁎}=B^{⁎}A^{†}A{\left(B^{†}\right)}^{⁎}-B^{⁎}{\left(E_{B}F_{A}\right)}^{†}A^{†}A{\left(B^{†}\right)}^{⁎}$*.*
〈102〉 $B^{†}A^{†}{\left(B^{†}A^{†}\right)}^{†}=B^{†}A^{†}AB-B^{†}A^{†}A{\left(F_{A}E_{B}\right)}^{†}B$ *and* ${\left(B^{†}A^{†}\right)}^{†}B^{†}A^{†}=ABB^{†}A^{†}-A{\left(F_{A}E_{B}\right)}^{†}BB^{†}A^{†}$*.*
〈103〉 $AB{\left(A^{†}AB\right)}^{†}A^{†}$ *and* $B^{†}{\left(ABB^{†}\right)}^{†}AB$ *are orthogonal projectors.*
〈104〉 $AB{\left(A^{⁎}AB\right)}^{†}A^{⁎}$ *and* $B^{⁎}{\left(ABB^{⁎}\right)}^{†}AB$ *are orthogonal projectors.*
〈105〉 $A^{†}(AB){\left(AB\right)}^{†}A$ *and* $B{\left(AB\right)}^{†}(AB)B^{†}$ *are orthogonal projectors.*
〈106〉 $A{\left(A^{†}ABB^{†}\right)}^{†}A^{†}$ *and* $B^{†}{\left(A^{†}ABB^{†}\right)}^{†}B$ *are orthogonal projectors.*
〈107〉 $ABB^{⁎}{\left(A^{⁎}ABB^{⁎}\right)}^{†}A^{⁎}$ *and* $B^{⁎}{\left(A^{⁎}ABB^{⁎}\right)}^{†}A^{⁎}AB$ *are orthogonal projectors.*
〈108〉 $A{\left(BB^{†}A^{†}A\right)}^{†}A^{†}$ *and* $B^{†}{\left(BB^{†}A^{†}A\right)}^{†}B$ *are orthogonal projectors.*
〈109〉 ${\left(A^{†}\right)}^{⁎}{\left(BB^{⁎}\right)}^{†}{\left({\left(A^{⁎}A\right)}^{†}{\left(BB^{⁎}\right)}^{†}\right)}^{†}A^{†}$ *and* $B^{†}{\left({\left(A^{⁎}A\right)}^{†}{\left(BB^{⁎}\right)}^{†}\right)}^{†}{\left(A^{⁎}A\right)}^{†}{\left(B^{†}\right)}^{⁎}$ *are orthogonal projectors.*
〈110〉 $A{\left(B^{†}A^{†}A\right)}^{†}B^{†}A^{†}$ *and* $B^{†}A^{†}{\left(BB^{†}A^{†}\right)}^{†}B$ *are orthogonal projectors.*
〈111〉 ${\left(A^{†}\right)}^{⁎}{\left(B^{†}{\left(A^{⁎}A\right)}^{†}\right)}^{†}B^{†}A^{†}$ *and* $B^{†}A^{†}{\left({\left(BB^{⁎}\right)}^{†}A^{†}\right)}^{†}{\left(B^{†}\right)}^{⁎}$ *are orthogonal projectors.*
〈112〉 $A^{†}{\left(B^{†}A^{†}\right)}^{†}(B^{†}A^{†})A$ *and* $B(B^{†}A^{†}){\left(B^{†}A^{†}\right)}^{†}B^{†}$ *are orthogonal projectors.*
〈113〉 $AB{\left(AB\right)}^{†}$ *and* $AA^{⁎}$ *commute, and* ${\left(AB\right)}^{†}AB$ *and* $B^{⁎}B$ *commute.*
〈114〉 $AB{\left(A^{†}AB\right)}^{†}A^{†}$ *and* $AA^{⁎}$ *commute, and* $B^{†}{\left(ABB^{†}\right)}^{†}AB$ *and* $B^{⁎}B$ *commute.*
〈115〉 $AB{\left(A^{⁎}AB\right)}^{†}A^{⁎}$ *and* $AA^{⁎}$ *commute, and* $B^{⁎}{\left(ABB^{⁎}\right)}^{†}AB$ *and* $B^{⁎}B$ *commute.*
〈116〉 $A^{†}(AB){\left(AB\right)}^{†}A$ *and* $A^{⁎}A$ *commute, and* $B{\left(AB\right)}^{†}(AB)B^{†}$ *and* $BB^{⁎}$ *commute.*
〈117〉 ${\left(B^{†}A^{†}\right)}^{†}(B^{†}A^{†})$ *and* ${\left(AA^{⁎}\right)}^{†}$ *commute, and* $(B^{†}A^{†}){\left(B^{†}A^{†}\right)}^{†}$ *and* ${\left(B^{⁎}B\right)}^{†}$ *commute.*
〈118〉 $A{\left(B^{†}A^{†}A\right)}^{†}B^{†}A^{†}$ *and* ${\left(AA^{⁎}\right)}^{†}$ *commute, and* $B^{†}A^{†}{\left(BB^{†}A^{†}\right)}^{†}B$ *and* ${\left(B^{⁎}B\right)}^{†}$ *commute.*
〈119〉 ${\left(A^{†}\right)}^{⁎}{\left(B^{†}{\left(A^{⁎}A\right)}^{†}\right)}^{†}B^{†}A^{†}$ *and* ${\left(AA^{⁎}\right)}^{†}$ *commute, and* $B^{†}A^{†}{\left({\left(BB^{⁎}\right)}^{†}A^{†}\right)}^{†}{\left(B^{†}\right)}^{⁎}$ *and* ${\left(B^{⁎}B\right)}^{†}$ *commute.*
〈120〉 $A^{†}{\left(B^{†}A^{†}\right)}^{†}(B^{†}A^{†})A$ *and* ${\left(A^{⁎}A\right)}^{†}$ *commute, and* $B(B^{†}A^{†}){\left(B^{†}A^{†}\right)}^{†}B^{†}$ *and* ${\left(BB^{⁎}\right)}^{†}$ *commute.*
〈121〉 $ABB^{†}A^{†}$ *and* $B^{†}A^{†}AB$ *are EP.*
〈122〉 $R\left({\left(AB\right)}^{†}\right)=R(B^{†}A^{†})$ *and* $R(AB)=R\left({\left(A^{†}\right)}^{⁎}{\left(B^{†}\right)}^{⁎}\right)$*.*
〈123〉 $R\left({\left(AB\right)}^{†}\right)=R\left(B^{†}{\left(ABB^{†}\right)}^{†}\right)$ *and* $R(AB)=R\left({\left(A^{†}\right)}^{⁎}{\left(B^{⁎}A^{†}A\right)}^{†}\right)$*.*
〈124〉 $R\left({\left(AB\right)}^{†}\right)=R\left(B^{⁎}{\left(ABB^{⁎}\right)}^{†}\right)$ *and* $R(AB)=R\left(A{\left(B^{⁎}A^{⁎}A\right)}^{†}\right)$*.*
〈125〉 $R\left({\left(AB\right)}^{†}\right)=R\left(B^{†}{\left(A^{†}ABB^{†}\right)}^{†}A^{†}\right)$ *and* $R(AB)=R\left({\left(A^{†}\right)}^{⁎}{\left(BB^{†}A^{†}A\right)}^{†}{\left(B^{†}\right)}^{⁎}\right)$*.*
〈126〉 $R\left({\left(AB\right)}^{†}\right)=R\left(B^{⁎}{\left(A^{⁎}ABB^{⁎}\right)}^{†}A^{⁎}\right)$ *and* $R(AB)=R\left(A{\left(BB^{⁎}A^{⁎}A\right)}^{†}B\right)$*.*
〈127〉 $R\left({\left(A^{⁎}ABB^{⁎}\right)}^{†}\right)=R\left(B{\left(AA^{⁎}ABB^{⁎}B\right)}^{†}A\right)$ *and* $R\left({\left(BB^{⁎}A^{⁎}A\right)}^{†}\right)=R\left(A^{⁎}{\left(B^{⁎}BB^{⁎}A^{⁎}AA^{⁎}\right)}^{†}B^{⁎}\right)$*.*
〈128〉 $R\left({\left(AA^{⁎}ABB^{⁎}B\right)}^{†}\right)=R\left(B^{†}{\left(A^{⁎}ABB^{⁎}\right)}^{†}A^{†}\right)$ *and* $R\left({\left(B^{⁎}BB^{⁎}A^{⁎}AA^{⁎}\right)}^{†}\right)=R\left({\left(A^{†}\right)}^{⁎}{\left(BB^{⁎}A^{⁎}A\right)}^{†}{\left(B^{†}\right)}^{⁎}\right)$*.*
〈129〉 $R\left({\left(AA^{⁎}ABB^{⁎}B\right)}^{†}\right)=R\left(B^{⁎}{\left({\left(A^{⁎}A\right)}^{2}{\left(BB^{⁎}\right)}^{2}\right)}^{†}A^{⁎}\right)$ *and* $R\left({\left(B^{⁎}BB^{⁎}A^{⁎}AA^{⁎}\right)}^{†}\right)=R\left(A{\left({\left(BB^{⁎}\right)}^{2}{\left(A^{⁎}A\right)}^{2}\right)}^{†}B\right)$*.*
〈130〉 $R\left({\left({\left(A^{⁎}A\right)}^{2}{\left(BB^{⁎}\right)}^{2}\right)}^{†}\right)=R\left({\left(B^{†}\right)}^{⁎}{\left(AA^{⁎}ABB^{⁎}B\right)}^{†}{\left(A^{†}\right)}^{⁎}\right)$ *and* $R\left({\left({\left(BB^{⁎}\right)}^{2}{\left(A^{⁎}A\right)}^{2}\right)}^{†}\right)=R\left(A^{†}{\left(B^{⁎}BB^{⁎}A^{⁎}AA^{⁎}\right)}^{†}B^{†}\right)$*.*
〈131〉 $R(AA^{⁎}AB)=R(AB)$ *and* $R\left(B^{⁎}B{\left(AB\right)}^{⁎}\right)=R\left({\left(AB\right)}^{⁎}\right)$*.*
〈132〉 $r[AA^{⁎}AB,\;AB]=r[{\left(AB\right)}^{⁎},\;B^{⁎}B{\left(AB\right)}^{⁎}]=r(AB)$*.*

Proof We only give proofs of some results in the list to illustrate the usefulness of the MRM and BMM in the establishment of the theorem. It is easy to verify that ${\left(AB\right)}^{†}$ and $B^{†}{\left(A^{†}ABB^{†}\right)}^{†}A^{†}$ are {2}-inverses of *AB*. Then by (4.23) and Lemma 8.6,

(11.26) $$r\left({\left(AB\right)}^{†}-B^{†}{\left(A^{†}ABB^{†}\right)}^{†}A^{†}\right)=r\left(B{\left(AB\right)}^{†}A-{\left(A^{†}ABB^{†}\right)}^{†}\right)=r\left[\begin{array}{c} {\left(AB\right)}^{†}\\ B^{†}{\left(A^{†}ABB^{†}\right)}^{†}A^{†} \end{array}\right]+r[{\left(AB\right)}^{†},\;B^{†}{\left(A^{†}ABB^{†}\right)}^{†}A^{†}]-r({\left(AB\right)}^{†})-r(B^{†}{\left(A^{†}ABB^{†}\right)}^{†}A^{†})=r\left[\begin{array}{c} {\left(AB\right)}^{⁎}\\ B^{⁎}A^{†} \end{array}\right]+r[B^{⁎}A^{⁎},\;B^{†}A^{⁎}]-2r(AB)=r\left[\begin{array}{c} AB\\ ABB^{⁎}B \end{array}\right]+r[AB,\;AA^{⁎}AB]-2r(AB).$$

Setting all sides of (11.26) equal to zero leads to the equivalences of 〈1〉, 〈2〉, 〈131〉, and 〈132〉.

Since ${\left(A^{†}AB\right)}^{†}A^{†}$ and $B^{†}{\left(ABB^{†}\right)}^{†}$ are {2}-inverses of *AB*, we find by (4.23) and Lemma 8.6 that

(11.27) $$r\left({\left(A^{†}AB\right)}^{†}A^{†}-B^{†}{\left(ABB^{†}\right)}^{†}\right)=r\left(B{\left(A^{†}AB\right)}^{†}-{\left(ABB^{†}\right)}^{†}A\right)=r\left[\begin{array}{c} {\left(A^{†}AB\right)}^{†}A^{†}\\ B^{†}{\left(ABB^{†}\right)}^{†} \end{array}\right]+r[{\left(A^{†}AB\right)}^{†}A^{†},\;B^{†}{\left(ABB^{†}\right)}^{†}]-r({\left(A^{†}AB\right)}^{†}A^{†})-r(B^{†}{\left(ABB^{†}\right)}^{†})=r\left[\begin{array}{c} B^{⁎}A^{†}\\ B^{†}A^{⁎} \end{array}\right]+r[B^{⁎}A^{†},\;B^{†}A^{⁎}]-2r(AB)=r\left[\begin{array}{c} AB\\ ABB^{⁎}B \end{array}\right]+r[AB,\;AA^{⁎}AB]-2r(AB).$$

Setting all sides of (11.27) equal to zero leads to the equivalences of 〈14〉, 〈15〉, and 〈132〉.

Let $T=E_{B}F_{A}$. Then by (4.13),

(11.28) $$r\left({\left(AB\right)}^{†}-B^{†}A^{†}+B^{†}T^{†}A^{†}\right)=r\left[\begin{array}{cc} T^{⁎}TT^{⁎} & T^{⁎}A^{†}\\ B^{†}T^{⁎} & B^{†}A^{†}-{\left(AB\right)}^{†} \end{array}\right]-r(T),$$

where the rank of *T* by (4.17) is

(11.29) $$r(T)=\left[\begin{array}{cc} I_{n} & B\\ A & 0 \end{array}\right]-r(A)-r(B)=n+r(AB)-r(A)-r(B).$$

Further by (4.6),

(11.30) $$r\left[\begin{array}{cc} T^{⁎}TT^{⁎} & T^{⁎}A^{†}\\ B^{†}T^{⁎} & B^{†}A^{†}-{\left(AB\right)}^{†} \end{array}\right]=r\left[\begin{array}{cc} \left(I_{n}-A^{†}A)T(I_{n}-BB^{†}\right) & (I_{n}-A^{†}A)(I_{n}-BB^{†})A^{†}\\ B^{†}(I_{n}-A^{†}A)(I_{n}-BB^{†}) & B^{†}A^{†}-{\left(AB\right)}^{†} \end{array}\right]=r\left[\begin{array}{ccc} T & (I_{n}-BB^{†})A^{†} & A^{†}\\ B^{†}(I_{n}-A^{†}A) & B^{†}A^{†}-{\left(AB\right)}^{†} & 0\\ B^{†} & 0 & 0 \end{array}\right]-r(A)-r(B)=r\left[\begin{array}{ccc} I_{n}+BB^{†}A^{†}A & -BB^{†}A^{†} & A^{†}\\ -B^{†}A^{†}A & B^{†}A^{†}-{\left(AB\right)}^{†} & 0\\ B^{†} & 0 & 0 \end{array}\right]-r(A)-r(B)=r\left[\begin{array}{ccc} I_{n} & -BB^{†}A^{†} & A^{†}\\ -{\left(AB\right)}^{†}A & B^{†}A^{†}-{\left(AB\right)}^{†} & 0\\ B^{†} & 0 & 0 \end{array}\right]-r(A)-r(B)=r\left[\begin{array}{ccc} I_{n} & 0 & 0\\ 0 & B^{†}A^{†}-{\left(AB\right)}^{†}-{\left(AB\right)}^{†}ABB^{†}A^{†} & {\left(AB\right)}^{†}\\ 0 & B^{†}A^{†} & -B^{†}A^{†} \end{array}\right]-r(A)-r(B)=r\left[\begin{array}{cc} \left(I_{n}-{\left(AB\right)}^{†}AB\right)B^{†}A^{†} & {\left(AB\right)}^{†}\\ 0 & -B^{†}A^{†} \end{array}\right]+n-r(A)-r(B)=r\left(B^{†}A^{†}-{\left(AB\right)}^{†}ABB^{†}A^{†}\right)+r\left[\begin{array}{c} {\left(AB\right)}^{†}\\ B^{†}A^{†} \end{array}\right]+n-r(A)-r(B),$$

where by (4.4) and Lemma 8.6,

(11.31) $$\;r\left(B^{†}A^{†}-{\left(AB\right)}^{†}ABB^{†}A^{†}\right)=r[{\left(AB\right)}^{†},\;B^{†}A^{†}]-r\left({\left(AB\right)}^{†}\right)=r[{\left(AB\right)}^{⁎},\;B^{†}A^{⁎}]-r(AB)=r[B^{⁎}B{\left(AB\right)}^{⁎},\;B^{⁎}A^{⁎}]-r(AB)=r\left[\begin{array}{c} ABB^{⁎}B\\ AB \end{array}\right]-r(AB),$$

and

(11.32) $$r\left[\begin{array}{c} {\left(AB\right)}^{†}\\ B^{†}A^{†} \end{array}\right]=r\left[{\left({\left(AB\right)}^{†}\right)}^{⁎},\;{\left(A^{†}\right)}^{⁎}{\left(B^{†}\right)}^{⁎}\right]=r\left[AB,\;{\left(A^{†}\right)}^{⁎}B\right]=r[AA^{⁎}AB,\;AB].$$

Substituting (11.29)–(11.32) into (11.28) yields

(11.33) $$r\left({\left(AB\right)}^{†}-B^{†}A^{†}+B^{†}T^{†}A^{†}\right)=r\left[\begin{array}{c} AB\\ ABB^{⁎}B \end{array}\right]+r[AB,\;AA^{⁎}AB]-2r(AB).$$

Setting both sides of (11.33) equal to zero leads to the equivalence of 〈96〉 and 〈132〉. In addition, we can obtain the following two rank equalities

$$r\left(AB{\left(AB\right)}^{†}-ABB^{†}A^{†}+ABB^{†}T^{†}A^{†}\right)=r[AB,\;AA^{⁎}AB]-r(AB),\;\;\;r\left({\left(AB\right)}^{†}AB-B^{†}A^{†}AB+B^{†}T^{†}A^{†}AB\right)=r\left[\begin{array}{c} AB\\ ABB^{⁎}B \end{array}\right]-r(AB)$$

by the same way. Setting both sides of these two equalities equal to zero leads to the equivalence of 〈100〉 and 〈132〉.

The equivalences of the remaining results follow from the combination of Lemma 11.2, Lemma 11.3, as well as algebraic calculations and derivations by the MRM. The details of the proofs are omitted due to space limitation. □

Theorem 11.4〈1〉 was proposed and studied in [44], and was re-approached in [88] by the MRM and BMM. Observe that the two nested equalities in Theorem 11.1〈7〉 occur in Theorem 9.1〈98〉 and Theorem 11.4〈1〉, both of which reveal a deep and fundamental connection between a single matrix equality in (11.1) and many other matrix equalities and facts. As a result of this observation, we immediately obtain by combining Theorem 9.1, Theorem 11.4 a noticeable conclusion that there are 165×132=21,780 groups of necessary and sufficient conditions for the ROL in (11.1) to hold. On the other hand, combining Theorem 11.1〈8〉 with Theorems 9.4(a), (b), (d), and (f) and 9.5(a), (b), (c), and (e) will separately lead to $38^{2}=1,444$ groups of necessary and sufficient conditions for the ROL in (11.1) to hold. Moreover, it has been known in matrix algebra that an EP matrix can be characterized by more than 200 equivalent statements (cf. [96, Theorem 8.1]). Hence each of the two EP conditions in 〈21〉 of Theorems 9.4(a) and 9.5(a) possesses more than 200 equivalent statements, which, as a consequence, will distinctively produce more than 40,000 groups of necessary and sufficient conditions for (11.1) to hold. All these record growths of the equivalent results and facts demonstrates that the ROL in (11.1) links a wide range of matrix equalities, matrix set inclusions, rank equalities, and range equalities for two matrices and their algebraic operations together, so that they provide comprehensive insights and perspectives into the profound performance of the ROL in (11.1), and make us to believe that the fundamental ROL is one of the most valuable and fruitful objects of study in the theory of generalized inverses.

## 12 Concluding remarks

As a summary, we conclude that this expository article studies primarily the relationships between ${\left(AB\right)}^{\left(i,\ldots ,j\right)}$ and $B^{\left(s_{2},\ldots ,t_{2}\right)}A^{\left(s_{1},\ldots ,t_{1}\right)}$ for the eight commonly-used types of generalized inverses of *A*, *B*, and *AB* by means of the three conventional yet distinguished methods in matrix theory—the matrix rank method, the block matrix method, and the matrix equation method, which brings together many conclusions on the matrix set inclusions in (1.10) in one organized, comprehensive, and self-contained place and provides a valuable source of reference. The whole work has contributed to our present understanding of many formulas, results, and facts in the theory of generalized inverses of matrices, and it is believed that the contributions in this paper will have a profound impact on the development of matrix equality theory, and many more profound and fruitful investigations can be conducted on various equality problems of matrix-valued functions and generalized inverses of matrix products. In particular, it is now an easy and routine work to give a group of satisfactory solutions to the statistical inference problems concerning OLSEs under linear regression models that are proposed and formulated in Section 2 as essential applications of the ROLs problems solved in this paper.

Furthermore, the present author remarks that although investigations on ROLs have evolved enough up to now, the formulas, results, and facts collected and proved, as well as the methods and techniques used and developed in this paper will have certain new impacts on the development of matrix equality theory of generalized inverses. The author hopes that the whole work in this paper can be utilized as a general guide to prompt many more consecutive approaches to matrix equalities composed of various complicated matrix operations and generalized inverses. Here we mention some groups of problems in this area for ongoing consideration.

(I) Apart from (1.8), generalized inverses of *AB* can be written as various mixed ROLs, such as,
  $${\left(AB\right)}^{\left(i,\ldots ,j\right)}=B^{\left(i_{1},\ldots ,j_{1}\right)}XA^{\left(i_{2},\ldots ,j_{2}\right)},\;\;\;\;{\left(AB\right)}^{\left(i,\ldots ,j\right)}=B^{\left(i_{1},\ldots ,j_{1}\right)}A^{\left(i_{2},\ldots ,j_{2}\right)}+Y$$
  for certain matrices *X* and *Y* composed of *A*, *B*, and their generalized inverses. Hence there are enormous concrete equalities for generalized inverses of *AB* that can be formulated. Several special cases of these mixed-type ROLs for Moore–Penrose inverses were proposed and approached (cf. [27], [28], [33], [44], [86], [88], [91], [98], [99]). In the following, we provide a sequence of acknowledged equalities (cf. [99]) for the Moore–Penrose inverse of the matrix product *AB*:
  $${\left(AB\right)}^{†}={\left(A^{†}AB\right)}^{†}A^{†},{\left(AB\right)}^{†}=B^{†}{\left(ABB^{†}\right)}^{†},{\left(AB\right)}^{†}={\left(A^{†}AB\right)}^{†}{\left(ABB^{†}\right)}^{†},{\left(AB\right)}^{†}=B^{†}{\left(ABB^{†}\right)}^{†}AB{\left(A^{†}AB\right)}^{†}A^{†},{\left(AB\right)}^{†}={\left(A^{⁎}AB\right)}^{†}A^{⁎},{\left(AB\right)}^{†}=B^{⁎}{\left(ABB^{⁎}\right)}^{†},{\left(AB\right)}^{†}=B^{⁎}{\left(ABB^{⁎}\right)}^{†}AB{\left(A^{⁎}AB\right)}^{†}A^{⁎},{\left(AB\right)}^{†}={\left({\left(A^{†}\right)}^{⁎}B\right)}^{†}{\left(AA^{⁎}\right)}^{†},{\left(AB\right)}^{†}={\left(B^{⁎}B\right)}^{†}{\left(A{\left(B^{†}\right)}^{⁎}\right)}^{†},$$
  $${\left(AB\right)}^{†}={\left({\left(A^{†}\right)}^{⁎}B\right)}^{†}{\left(A^{†}\right)}^{⁎}{\left(B^{†}\right)}^{⁎}{\left(A{\left(B^{†}\right)}^{⁎}\right)}^{†},{\left(AB\right)}^{†}={\left(B^{⁎}B\right)}^{†}{\left(A{\left(B^{†}\right)}^{⁎}\right)}^{†}AB{\left({\left(A^{†}\right)}^{⁎}B\right)}^{†}{\left(AA^{⁎}\right)}^{†},{\left(AB\right)}^{†}={\left(AA^{⁎}AB\right)}^{†}AA^{⁎},{\left(AB\right)}^{†}=B^{⁎}B{\left(ABB^{⁎}B\right)}^{†},{\left(AB\right)}^{†}=B^{⁎}B{\left(ABB^{⁎}B\right)}^{†}AB{\left(AA^{⁎}AB\right)}^{†}AA^{⁎},{\left(AB\right)}^{†}=B^{†}{\left(A^{†}ABB^{†}\right)}^{†}A^{†},{\left(AB\right)}^{†}=B^{⁎}{\left(A^{⁎}ABB^{⁎}\right)}^{†}A^{⁎},{\left(AB\right)}^{†}={\left(B^{⁎}B\right)}^{†}{\left({\left(A^{†}\right)}^{⁎}{\left(B^{†}\right)}^{⁎}\right)}^{†}{\left(AA^{⁎}\right)}^{†},{\left(AB\right)}^{†}=B^{⁎}B{\left(AA^{⁎}ABB^{⁎}B\right)}^{†}AA^{⁎},{\left(AB\right)}^{†}={\left(BB^{⁎}B\right)}^{†}{\left({\left(A^{⁎}A\right)}^{†}{\left(BB^{⁎}\right)}^{†}\right)}^{†}{\left(AA^{⁎}A\right)}^{†},{\left(AB\right)}^{†}=B^{⁎}BB^{⁎}{\left({\left(A^{⁎}A\right)}^{2}{\left(BB^{⁎}\right)}^{2}\right)}^{†}A^{⁎}AA^{⁎},{\left(AB\right)}^{†}={\left({\left(B^{⁎}B\right)}^{†}\right)}^{2}{\left({\left(A^{⁎}AA^{⁎}\right)}^{†}{\left(B^{⁎}BB^{⁎}\right)}^{†}\right)}^{†}{\left({\left(AA^{⁎}\right)}^{†}\right)}^{2},{\left(AB\right)}^{†}={\left(B^{⁎}B\right)}^{2}{\left({\left(AA^{⁎}\right)}^{2}AB{\left(B^{⁎}B\right)}^{2}\right)}^{†}{\left(AA^{⁎}\right)}^{2},{\left(AB\right)}^{†}=B^{†}A^{†}-B^{†}{\left((I_{n}-BB^{†})(I_{n}-A^{†}A)\right)}^{†}A^{†}.$$
  These equalities have certain essential links with ${\left(AB\right)}^{†}=B^{†}A^{†}$, as demonstrated in the preceding sections. Apart from the ROL problems for the Moore–Penrose inverse of the two-term matrix product *AB*, people also proposed and studied ROLs for the Moore–Penrose of a triple matrix product *ABC*, where $A\in C^{m\times n}$, $B\in C^{n\times p}$, and $C\in C^{p\times q}$. Some initial work on the standard ROL: ${\left(ABC\right)}^{†}=C^{†}B^{†}A^{†}$ was presented in [41], and a sequence of known and new mixed-type ROLs for the Moore–Penrose of *ABC* were collected and formulated in the author's recent paper [99] as follows:
  $${\left(ABC\right)}^{†}={\left(BC\right)}^{†}B{\left(AB\right)}^{†},{\left(ABC\right)}^{†}=C^{†}{\left(BCC^{†}\right)}^{†}B{\left(A^{†}AB\right)}^{†}A^{†},{\left(ABC\right)}^{†}={\left(B^{†}BC\right)}^{†}{\left(BCC^{†}\right)}^{†}B{\left(A^{†}AB\right)}^{†}{\left(ABB^{†}\right)}^{†},{\left(ABC\right)}^{†}={\left(B^{†}BC\right)}^{†}B^{†}{\left(ABB^{†}\right)}^{†},{\left(ABC\right)}^{†}=C^{†}{\left(B^{†}BCC^{†}\right)}^{†}B^{†}{\left(A^{†}ABB^{†}\right)}^{†}A^{†},{\left(ABC\right)}^{†}=C^{†}{\left(BCC^{†}\right)}^{†}BC{\left(B^{†}BC\right)}^{†}B^{†}{\left(ABB^{†}\right)}^{†}AB{\left(A^{†}AB\right)}^{†}A^{†},{\left(ABC\right)}^{†}=C^{⁎}{\left(BCC^{⁎}\right)}^{†}B{\left(A^{⁎}AB\right)}^{†}A^{⁎},{\left(ABC\right)}^{†}={\left(B^{⁎}BC\right)}^{†}B^{⁎}BB^{⁎}{\left(ABB^{⁎}\right)}^{†},{\left(ABC\right)}^{†}=C^{⁎}{\left(B^{⁎}BCC^{⁎}\right)}^{†}B^{⁎}BB^{⁎}{\left(A^{⁎}ABB^{⁎}\right)}^{†}A^{⁎},{\left(ABC\right)}^{†}=C^{†}{\left(AB\right)}^{†}ABC{\left(BC\right)}^{†}A^{†},{\left(ABC\right)}^{†}=C^{†}B^{†}{\left(ABB^{†}\right)}^{†}ABC{\left(B^{†}BC\right)}^{†}B^{†}A^{†},{\left(ABC\right)}^{†}=C^{†}B^{†}{\left(A^{†}ABB^{†}\right)}^{†}B{\left(B^{†}BCC^{†}\right)}^{†}B^{†}A^{†},{\left(ABC\right)}^{†}={\left(BC\right)}^{†}A^{†}ABCC^{†}{\left(AB\right)}^{†},{\left(ABC\right)}^{†}=C^{†}{\left(BCC^{†}\right)}^{†}A^{†}ABCC^{†}{\left(A^{†}AB\right)}^{†}A^{†},{\left(ABC\right)}^{†}=C^{⁎}{\left(BCC^{⁎}\right)}^{†}A^{†}ABCC^{†}{\left(A^{⁎}AB\right)}^{†}A^{⁎},{\left(ABC\right)}^{†}={\left(A^{†}ABC\right)}^{†}A^{†},{\left(ABC\right)}^{†}=C^{†}{\left(ABCC^{†}\right)}^{†},{\left(ABC\right)}^{†}={\left(A^{†}ABC\right)}^{†}B{\left(ABCC^{†}\right)}^{†},{\left(ABC\right)}^{†}=C^{†}{\left(ABCC^{†}\right)}^{†}ABC{\left(A^{†}ABC\right)}^{†}A^{†},{\left(ABC\right)}^{†}={\left(A^{⁎}ABC\right)}^{†}A^{⁎},{\left(ABC\right)}^{†}=C^{⁎}{\left(ABCC^{⁎}\right)}^{†},{\left(ABC\right)}^{†}=C^{⁎}{\left(ABCC^{⁎}\right)}^{†}ABC{\left(A^{⁎}ABC\right)}^{†}A^{⁎},{\left(ABC\right)}^{†}={\left({\left(A^{†}\right)}^{⁎}BC\right)}^{†}{\left(AA^{⁎}\right)}^{†},{\left(ABC\right)}^{†}={\left(C^{⁎}C\right)}^{†}{\left(AB{\left(C^{†}\right)}^{⁎}\right)}^{†},{\left(ABC\right)}^{†}={\left(C^{⁎}C\right)}^{†}{\left(AB{\left(C^{†}\right)}^{⁎}\right)}^{†}ABC{\left({\left(A^{†}\right)}^{⁎}BC\right)}^{†}{\left(AA^{⁎}\right)}^{†},{\left(ABC\right)}^{†}={\left(AA^{⁎}ABC\right)}^{†}AA^{⁎},$$
  $${\left(ABC\right)}^{†}=C^{⁎}C{\left(ABCC^{⁎}C\right)}^{†},{\left(ABC\right)}^{†}=C^{⁎}C{\left(ABCC^{⁎}C\right)}^{†}ABC{\left(AA^{⁎}ABC\right)}^{†}AA^{⁎},{\left(ABC\right)}^{†}={\left({\left(AB\right)}^{†}ABC\right)}^{†}{\left(AB\right)}^{†},{\left(ABC\right)}^{†}={\left(BC\right)}^{†}{\left(ABC{\left(BC\right)}^{†}\right)}^{†},{\left(ABC\right)}^{†}={\left(BC\right)}^{†}{\left(ABC{\left(BC\right)}^{†}\right)}^{†}ABC{\left({\left(AB\right)}^{†}ABC\right)}^{†}{\left(AB\right)}^{†},{\left(ABC\right)}^{†}={\left(BC\right)}^{†}{\left({\left(AB\right)}^{†}ABC{\left(BC\right)}^{†}\right)}^{†}{\left(AB\right)}^{†},{\left(ABC\right)}^{†}={\left({\left(AB\right)}^{⁎}ABC\right)}^{†}{\left(AB\right)}^{⁎},{\left(ABC\right)}^{†}={\left(BC\right)}^{⁎}{\left(ABC{\left(BC\right)}^{⁎}\right)}^{†},{\left(ABC\right)}^{†}={\left(BC\right)}^{⁎}{\left(ABC{\left(BC\right)}^{⁎}\right)}^{†}ABC{\left({\left(AB\right)}^{⁎}ABC\right)}^{†}{\left(AB\right)}^{⁎},{\left(ABC\right)}^{†}={\left(BC\right)}^{⁎}{\left({\left(AB\right)}^{⁎}ABC{\left(BC\right)}^{⁎}\right)}^{†}{\left(AB\right)}^{⁎},{\left(ABC\right)}^{†}={\left({\left({\left(AB\right)}^{†}\right)}^{⁎}C\right)}^{†}{\left((AB){\left(AB\right)}^{⁎}\right)}^{†},{\left(ABC\right)}^{†}={\left({\left(BC\right)}^{⁎}(BC)\right)}^{†}{\left(A{\left({\left(BC\right)}^{†}\right)}^{⁎}\right)}^{†},{\left(ABC\right)}^{†}={\left({\left(ABB^{†}\right)}^{†}ABC\right)}^{†}{\left(ABB^{†}\right)}^{†},{\left(ABC\right)}^{†}={\left(B^{†}BC\right)}^{†}{\left(ABC{\left(B^{†}BC\right)}^{†}\right)}^{†},{\left(ABC\right)}^{†}={\left(B^{†}BC\right)}^{†}{\left(ABC{\left(B^{†}BC\right)}^{†}\right)}^{†}ABC{\left({\left(ABB^{†}\right)}^{†}ABC\right)}^{†}{\left(ABB^{†}\right)}^{†},{\left(ABC\right)}^{†}={\left({\left(ABB^{⁎}\right)}^{†}ABC\right)}^{†}{\left(ABB^{⁎}\right)}^{†},{\left(ABC\right)}^{†}={\left(B^{⁎}BC\right)}^{†}{\left(ABC{\left(B^{⁎}BC\right)}^{†}\right)}^{†},{\left(ABC\right)}^{†}={\left(B^{⁎}BC\right)}^{†}{\left(ABC{\left(B^{⁎}BC\right)}^{†}\right)}^{†}ABC{\left({\left(ABB^{⁎}\right)}^{†}ABC\right)}^{†}{\left(ABB^{⁎}\right)}^{†},{\left(ABC\right)}^{†}={\left(B^{†}{\left(ABB^{†}\right)}^{†}ABC\right)}^{†}B^{†}{\left(ABB^{†}\right)}^{†},{\left(ABC\right)}^{†}={\left(B^{†}BC\right)}^{†}B^{†}{\left(ABC{\left(B^{†}BC\right)}^{†}B^{†}\right)}^{†},{\left(ABC\right)}^{†}={\left(B^{†}BC\right)}^{†}B^{†}{\left(ABC{\left(B^{†}BC\right)}^{†}B^{†}\right)}^{†}ABC{\left(B^{†}{\left(ABB^{†}\right)}^{†}ABC\right)}^{†}B^{†}{\left(ABB^{†}\right)}^{†},{\left(ABC\right)}^{†}={\left(B^{⁎}{\left(ABB^{⁎}\right)}^{†}ABC\right)}^{†}B^{⁎}{\left(ABB^{⁎}\right)}^{†},{\left(ABC\right)}^{†}={\left(B^{⁎}BC\right)}^{†}B^{⁎}{\left(ABC{\left(B^{⁎}BC\right)}^{†}B^{⁎}\right)}^{†},{\left(ABC\right)}^{†}={\left(B^{⁎}BC\right)}^{†}B^{⁎}{\left(ABC{\left(B^{⁎}BC\right)}^{†}B^{⁎}\right)}^{†}ABC{\left(B^{⁎}{\left(ABB^{⁎}\right)}^{†}ABC\right)}^{†}B^{⁎}{\left(ABB^{⁎}\right)}^{†},{\left(ABC\right)}^{†}={\left({\left({\left(AB\right)}^{†}\right)}^{⁎}C\right)}^{†}{\left({\left(AB\right)}^{†}\right)}^{⁎}B^{†}{\left({\left(BC\right)}^{†}\right)}^{⁎}{\left(A{\left({\left(BC\right)}^{†}\right)}^{⁎}\right)}^{†},{\left(ABC\right)}^{†}={\left({\left(BC\right)}^{⁎}(BC)\right)}^{†}{\left(A{\left({\left(BC\right)}^{†}\right)}^{⁎}\right)}^{†}ABC{\left({\left({\left(AB\right)}^{†}\right)}^{⁎}C\right)}^{†}{\left((AB){\left(AB\right)}^{⁎}\right)}^{†},{\left(ABC\right)}^{†}={\left({\left(A{\left(B^{†}\right)}^{⁎}\right)}^{†}ABC\right)}^{†}{\left(A{\left(B^{†}\right)}^{⁎}\right)}^{†},{\left(ABC\right)}^{†}={\left({\left(B^{†}\right)}^{⁎}C\right)}^{†}{\left(ABC{\left({\left(B^{†}\right)}^{⁎}C\right)}^{†}\right)}^{†},{\left(ABC\right)}^{†}={\left({\left(A{\left(B^{†}\right)}^{⁎}\right)}^{†}ABC\right)}^{†}{\left(A{\left(B^{†}\right)}^{⁎}\right)}^{†}ABC{\left({\left(B^{†}\right)}^{⁎}C\right)}^{†}{\left(ABC{\left({\left(B^{†}\right)}^{⁎}C\right)}^{†}\right)}^{†},{\left(ABC\right)}^{†}={\left({\left(B^{⁎}B\right)}^{†}{\left(A{\left(B^{†}\right)}^{⁎}\right)}^{†}ABC\right)}^{†}{\left(B^{⁎}B\right)}^{†}{\left(A{\left(B^{†}\right)}^{⁎}\right)}^{†},{\left(ABC\right)}^{†}={\left({\left(B^{†}\right)}^{⁎}C\right)}^{†}{\left(BB^{⁎}\right)}^{†}{\left(ABC{\left({\left(B^{†}\right)}^{⁎}C\right)}^{†}{\left(BB^{⁎}\right)}^{†}\right)}^{†},{\left(ABC\right)}^{†}={\left({\left(B^{†}\right)}^{⁎}C\right)}^{†}{\left(BB^{⁎}\right)}^{†}{\left({\left(B^{⁎}B\right)}^{†}{\left(A{\left(B^{†}\right)}^{⁎}\right)}^{†}ABC{\left({\left(B^{†}\right)}^{⁎}C\right)}^{†}{\left(BB^{⁎}\right)}^{†}\right)}^{†}{\left(B^{⁎}B\right)}^{†}{\left(A{\left(B^{†}\right)}^{⁎}\right)}^{†},{\left(ABC\right)}^{†}=C^{†}{\left(A^{†}ABCC^{†}\right)}^{†}A^{†},{\left(ABC\right)}^{†}=C^{⁎}{\left(A^{⁎}ABCC^{⁎}\right)}^{†}A^{⁎},{\left(ABC\right)}^{†}={\left(C^{⁎}C\right)}^{†}{\left({\left(A^{†}\right)}^{⁎}B{\left(C^{†}\right)}^{⁎}\right)}^{†}{\left(AA^{⁎}\right)}^{†},{\left(ABC\right)}^{†}={\left(C^{⁎}C\right)}^{†}{\left(B{\left(C^{†}\right)}^{⁎}\right)}^{†}B{\left({\left(A^{†}\right)}^{⁎}B\right)}^{†}{\left(AA^{⁎}\right)}^{†},{\left(ABC\right)}^{†}=C^{⁎}C{\left(AA^{⁎}ABCC^{⁎}C\right)}^{†}AA^{⁎},{\left(ABC\right)}^{†}=C^{⁎}CC^{⁎}{\left({\left(A^{⁎}A\right)}^{2}B{\left(CC^{⁎}\right)}^{2}\right)}^{†}A^{⁎}AA^{⁎},{\left(ABC\right)}^{†}=C^{†}{\left(BCC^{†}\right)}^{†}{\left({\left(A^{†}AB\right)}^{†}A^{†}ABCC^{†}{\left(BCC^{†}\right)}^{†}\right)}^{†}{\left(A^{†}AB\right)}^{†}A^{†},{\left(ABC\right)}^{†}=C^{⁎}{\left(BCC^{⁎}\right)}^{†}{\left({\left(A^{⁎}AB\right)}^{†}A^{⁎}ABCC^{⁎}{\left(BCC^{⁎}\right)}^{†}\right)}^{†}{\left(A^{⁎}AB\right)}^{†}A^{⁎},{\left(ABC\right)}^{†}={\left(B^{†}BC\right)}^{†}B^{†}{\left(B^{†}{\left(ABB^{†}\right)}^{†}ABC{\left(B^{†}BC\right)}^{†}B^{†}\right)}^{†}B^{†}{\left(ABB^{†}\right)}^{†},{\left(ABC\right)}^{†}={\left(B^{⁎}BC\right)}^{†}B^{⁎}{\left(B^{⁎}{\left(ABB^{⁎}\right)}^{†}ABC{\left(B^{⁎}BC\right)}^{†}B^{⁎}\right)}^{†}B^{⁎}{\left(ABB^{⁎}\right)}^{†},{\left(ABC\right)}^{†}=C^{†}{\left(B{\left(AB\right)}^{†}ABC{\left(BC\right)}^{†}B\right)}^{†}A^{†},{\left(ABC\right)}^{†}={\left(BC\right)}^{†}B{\left(B{\left(AB\right)}^{†}ABC{\left(BC\right)}^{†}B\right)}^{†}B{\left(AB\right)}^{†},{\left(ABC\right)}^{†}={\left(B{\left(AB\right)}^{†}ABC\right)}^{†}B{\left(AB\right)}^{†}ABC{\left(BC\right)}^{†}B{\left(ABC{\left(BC\right)}^{†}B\right)}^{†},$$
  $${\left(ABC\right)}^{†}={\left({\left(ABB^{†}\right)}^{†}ABC\right)}^{†}B{\left(ABC{\left(B^{†}BC\right)}^{†}\right)}^{†},{\left(ABC\right)}^{†}={\left({\left(A^{†}\right)}^{⁎}BC\right)}^{†}{\left(A^{†}\right)}^{⁎}B{\left(C^{†}\right)}^{⁎}{\left(AB{\left(C^{†}\right)}^{⁎}\right)}^{†},{\left(ABC\right)}^{†}={\left({\left(A{\left(B^{†}\right)}^{⁎}\right)}^{†}ABC\right)}^{†}B^{⁎}BB^{⁎}{\left(ABC{\left({\left(B^{†}\right)}^{⁎}C\right)}^{†}\right)}^{†},{\left(ABC\right)}^{†}={\left(C{\left(BC\right)}^{†}BC\right)}^{†}B^{†}{\left(AB{\left(AB\right)}^{†}A\right)}^{†},{\left(ABC\right)}^{†}={\left({\left(AB\right)}^{†}ABC\right)}^{†}B^{†}{\left(ABC{\left(BC\right)}^{†}\right)}^{†},{\left(ABC\right)}^{†}={\left(BC\right)}^{†}BCC^{†}B^{†}A^{†}AB{\left(AB\right)}^{†},{\left(ABC\right)}^{†}={\left(C^{⁎}C\right)}^{†}{\left({\left(C^{†}B^{†}\right)}^{†}\right)}^{⁎}{\left(B^{⁎}BB^{⁎}\right)}^{†}{\left({\left(B^{†}A^{†}\right)}^{†}\right)}^{⁎}{\left(AA^{⁎}\right)}^{†},{\left(ABC\right)}^{†}={\left({\left(B^{†}\right)}^{⁎}C\right)}^{†}{\left(B^{⁎}BB^{⁎}\right)}^{†}{\left(A{\left(B^{†}\right)}^{⁎}\right)}^{†},{\left(ABC\right)}^{†}={\left({\left(BC\right)}^{⁎}(BC)\right)}^{†}{\left({\left({\left(AB\right)}^{†}\right)}^{⁎}B^{†}{\left({\left(BC\right)}^{†}\right)}^{⁎}\right)}^{†}{\left((AB){\left(AB\right)}^{⁎}\right)}^{†},{\left(ABC\right)}^{†}=(C^{†}-C^{†}T_{2}^{†})B^{†}(A^{†}-T_{1}^{†}A^{†}),{\left(ABC\right)}^{†}={\left(BC\right)}^{†}B{\left(AB\right)}^{†}-{\left(BC\right)}^{†}B{\left((I_{n}-P_{BC})B(I_{p}-P_{{\left(AB\right)}^{⁎}})\right)}^{†}B{\left(AB\right)}^{†},{\left(ABC\right)}^{†}={\left(B^{†}BC\right)}^{†}B^{†}{\left(ABB^{†}\right)}^{†}-{\left(B^{†}BC\right)}^{†}B^{†}{\left((I_{p}-P_{B^{†}BC})B^{†}(I_{n}-P_{{\left(ABB^{†}\right)}^{⁎}})\right)}^{†}B^{†}{\left(ABB^{†}\right)}^{†},$$
  where $T_{1}=(I_{n}-BB^{†})(I_{n}-A^{†}A)$, $T_{2}=(I_{p}-CC^{†})(I_{p}-B^{†}B)$, $P_{BC}=(BC){\left(BC\right)}^{†}$, $P_{{\left(AB\right)}^{⁎}}={\left(AB\right)}^{†}(AB)$, $P_{B^{†}BC}=(B^{†}BC){\left(B^{†}BC\right)}^{†}$, and $P_{{\left(ABB^{†}\right)}^{⁎}}={\left(ABB^{†}\right)}^{†}(ABB^{†})$.
  Most of the above mixed-type ROLs for the Moore–Penrose inverses and their variations have not been touched in the theory of generalized inverses, so that the approaches to these matrix equalities seem challenging, but the author claims that all these ROL problems can be characterized by the MRM and BMM, as demonstrated in the preceding sections, and the whole work will produce tremendous amount of results and facts on matrix equalities composed of generalized inverses, which will definitely improve our capacity of dealing with complicated matrix operations of matrices and their generalized inverses.
(II) As a direct extension of the Moore–Penrose inverse, the weighted Moore–Penrose inverse of a matrix $A\in C^{m\times n}$ with respect to two Hermitian positive semi-definite matrices $M\in C^{m\times m}$ and $N\in C^{n\times n}$ is defined to be the solution *X* satisfying the following four equations (1) MAXA=MA, (2) NXAX=NX, (3) ${\left(MAX\right)}^{⁎}=MAX$, (4) ${\left(NXA\right)}^{⁎}=NXA$ (cf. page 118, Exercise 33 in [17]). A matrix *X* is called a weighted {i,…,j}-generalized inverse of *A*, denoted by $A_{M,N}^{\left(i,\ldots ,j\right)}$, if it satisfies the above *i*th, …,jth equations. The collection of all weighted {i,…,j}-generalized inverses of *A* is denoted by $\left\{A_{M,N}^{\left(i,\ldots ,j\right)}\right\}$. There are also 15 types of weighted {i,…,j}-generalized inverses of *A* according to the above definition. In this situation, it would be of interest to consider the extensions of the preceding results to various ROLs for weighted generalized inverses of a matrix product.
(III) Similar to generalized inverses of matrices, algebraists have defined Moore–Penrose inverses of elements in other general algebraic frameworks, such as, semigroups, rings, operator algebras, Hilbert $C^{⁎}$-modules (cf. [18], [20], [22], [29], [32], [39], [44], [47], [60], [72], [125]). Without loss of generality, assume that the algebraic system is assumed to be a $C^{⁎}$-algebra A. An operator a∈A is said to be regular (in the sense of von Neumann) if there exists an x∈A for which aba=a; any such *x* is called an inner inverse of *a*. For an operator a∈A, the four Penrose equations are defined to be (1) axa=a, (2) xax=x, (3) ${\left(ax\right)}^{⁎}=ax$, (4) ${\left(xa\right)}^{⁎}=xa$. If the four equations have an common solution *x*, then it is unique, and is called the Moore–Penrose inverse of *a* and denoted by $x=a^{†}$ (cf. [20], [22]). It is well known that *a* is regular ⇔ aA is closed ⇔ $a^{†}$ exists (cf. [39]). Thus it would be of interest to extend most of the preceding formulas, results, and facts concerning ROL problems to generalized inverses of products of operators in $C^{⁎}$-algebras, as well as other algebraic systems. The author, however, remarks that principle conclusions obtained concerning a ROL for generalized inverses of products of elements in other algebraic systems should be consistent in nature with these on the same ROL for generalized inverses of products of complex matrices because the conventional operation rules of the additions, multiplications, and ⁎-involutions, as well as, the definitions of the Moore–Penrose inverses in these algebraic systems are symbolically the same.

## Declarations

### Funding statement

This research did not receive any specific grant from funding agencies in the public, commercial, or not-for-profit sectors.

### Competing interest statement

The author declares no conflict of interest.

### Additional information

No additional information is available for this paper.
